# First-in-Class Colchicine-Based Visible Light Photoswitchable Microtubule Dynamics Disrupting Agent

**DOI:** 10.3390/cells12141866

**Published:** 2023-07-16

**Authors:** Filip Borys, Piotr Tobiasz, Hanna Fabczak, Ewa Joachimiak, Hanna Krawczyk

**Affiliations:** 1 Department of Organic Chemistry, Faculty of Chemistry, Warsaw University of Technology, Noakowskiego 3 Street, 00-664 Warsaw, Poland; piotr.tobiasz@pw.edu.pl; 2 Laboratory of Cytoskeleton and Cilia Biology, Nencki Institute of Experimental Biology, Polish Academy of Sciences, 3 Pasteur Street, 02-093 Warsaw, Poland; h.fabczak@nencki.edu.pl (H.F.); e.joachimiak@nencki.edu.pl (E.J.)

**Keywords:** photopharmacology, colchicine, tubulin, photoswitches

## Abstract

Compounds that disrupt microtubule dynamics, such as colchicine, paclitaxel, or Vinca alkaloids, have been broadly used in biological studies and have found application in clinical anticancer medications. However, their main disadvantage is the lack of specificity towards cancerous cells, leading to severe side effects. In this paper, we report the first synthesis of 12 new visible light photoswitchable colchicine-based microtubule inhibitors **AzoCols**. Among the obtained compounds, two photoswitches showed light-dependent cytotoxicity in cancerous cell lines (HCT116 and MCF-7). The most promising compound displayed a nearly twofold increase in potency. Moreover, dissimilar inhibition of purified tubulin polymerisation in cell-free assay and light-dependent disruption of microtubule organisation visualised by immunofluorescence imaging sheds light on the mechanism of action as microtubule photoswitchable destabilisers. The presented results provide a foundation towards the synthesis and development of a novel class of photoswitchable colchicine-based microtubule polymerisation inhibitors.

## 1. Introduction

**Figure 1:**
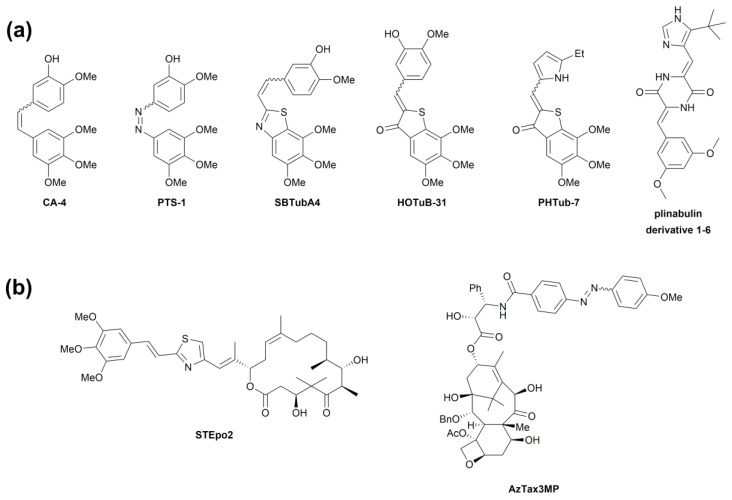
Structures of (**a**) combretastatin A-4 **CA4** and known photoswitchable microtubule destabilisers and (**b**) stabilisers.

Photopharmacology is an emerging method based on incorporation of photoswitchable component—molecular switches into the skeleton of a parent compound with expected biological activity. The goal of photopharmacology is to reduce the effects of drug substances apart from the cellular target and severe systemic/environmental side effects by establishing an external and selective means of controlling the activity of these compounds with time and spatial precision. It involves the design, synthesis, research, and application of drugs in the form of photochromic molecular switches which can be regulated by light [1,2,3,4]. Although photopharmacology is a relatively new technique that has not yet found clinical application, recent years have abounded with outstanding research towards the development of novel photoresponsive bioactive compounds including G protein-coupled receptors (GPCRs) agonists [5,6,7], ion channels activity modulators [8,9,10], and enzyme inhibitors [11]. Azobenzenes are photoswitches that can have many applications in photopharmacology. They can be switched between the (*E*) and (*Z*) configurations by light [12,13]. Due to their small size, high quantum efficiency, high extinction coefficients, low photobleaching factor, and easy synthesis, they are perfect structural elements for creating complex optical tools—they require low-intensity light and, because of their stability, they can be switched many times in a large number of cycles [14,15,16]. For example, the action of photopharmaceuticals containing skeletal fragments of azobenzene as a functionalizing unit allows for control of biological functions with precision in space and time [17,18,19,20]. Classical azobenzenes also have disadvantages that limit their practical use in biological sciences. The first is the necessity to use destructive and harmful-to-cells UV light necessary to induce (*E*) → (*Z*) isomerisation by excitation of π → π*. The second is incomplete reverse (*Z*) → (*E*) photoisomerisation, caused by radiation with the maximum absorption in the visible region, in which the n → π* bands of (*E*) and (*Z*) isomers overlap, which makes it impossible to selectively analyse each of the geometric isomers and select excitation. Therefore, azobenzenes should be modified by introducing various substituents, e.g., halogen, alkoxyl, etc. [21,22,23]. Implementation of ortho-fluorine atoms renders the separation of the n → π* absorption bands in the UV-VIS spectrum possible. This enables selective addressing of each geometric isomer and its selective activation [24,25,26,27]. Microtubules (MT) are vital cytoskeletal constituent present in eukaryotic cells which are involved in many cellular processes. MT are hollow cylindrical polymers composed of αβ-tubulin heterodimers noncovalently bounded longitudinally and laterally. Their ability to rapidly reorganise from growing (by incorporating new αβ-tubulin subunits at the (+) end of MT) to shrinking (by removal of αβ-tubulin subunits) and vice versa, known as “dynamic instability”, is crucial for MT bioactivity and can be modified by small molecules known as microtubule-targeting agents (MTAs). These compounds can be divided into two main groups, depending on their influence on polymerised tubulin mass at high concentrations, namely microtubule-stabilising agents (e.g., taxanes and epothilones) and microtubule-destabilising agents (e.g., vinca alkaloids, maytansines, and colchicine derivatives) [28,29]. In turn, when low concentrations are applied, both classes suppress microtubule dynamics [30,31,32]. Importantly, microtubule-targeting agents disrupt formation and proper activity of mitotic spindle, leading to impaired chromosome segregation during mitosis and, consequently, cell death [33]. Over the past few decades, hundreds of MTAs have been synthesised and evaluated for their bioactivity [34,35,36]. However, there are only a handful of classes of photoswitchable microtubule-targeting agents (PMTAs) that are currently known [19]. Studies from three independent groups have described the first potent photoswitchable analogues of combretastatin A-4 **CA4**, namely photostatins **PTS-1**, in which the isosteric nitrogen–nitrogen double bond replaces the carbon–carbon double bond of **CA4** (Figure 1a) [37,38,39]. Since then, other photoswitchable microtubule-destabilising agents based on **CA4** analogues have been developed, e.g., hemithioindigos **HOTub-31**, **PHTub-7** [40,41,42], spiropyrans [43,44], and styrylbenzothiazoles (**SBTub-A4**) [45,46] (Figure 1a). Recently, photoswitchable plinabulin-based microtubule inhibitors have been developed [47]. Despite extensive research towards novel PMTAs, no colchicine-based photoswitchable microtubule-destabilising agents have yet been described. In contrast, only two classes of photoswitchable microtubule-stabilising agents have been published: paclitaxel-based [48] and epothilone-based photoswitchable microtubule stabilisers [49] (Figure 1b). Each set of compounds has its own disadvantages and advantages. In cases where UV light is used to induce photoisomerization in a biological context, several inherent limitations have to be taken into account. UV light has low tissue penetration ability [50], and hard UV light might lead to DNA mutations; thus, it is toxic to normal cells. Furthermore, high-energy UV light can cause irreversible photolysis (e.g., photooxidation, photoisomerisation, free radical formation) and thus cannot be applied in some pharmacophore structures. It was shown that exposure to UV light irradiation of colchicine causes the formation of β-lumicolchicine, γ-lumicolchicine, α-lumicolchicine, and loss of bioactivity [51,52].

In this study, our goal was to develop novel visible light photoswitchable colchicine-based microtubule disrupting agents and assess their antiproliferative activity against selected tumorous cell lines.

## 2. Materials and Methods

### 2.1. Synthesis

Compound characterisation and copies of NMR spectra are provided in the Supporting Information.

(*R*)-N-deacetyl colchicine was synthesised according to a protocol previously published [53].

General procedure A: the aqueous solution (35 mL) of oxone^®^ (3.5 mmol) was added, dropwise, to the solution of aniline derivative **1a**–**d** (1 mmol) in dichloromethane (20 mL). The reaction mixture was vigorous stirred at room temperature. After disappearance of the starting material (analysed by TLC), the reaction was quenched by addition of NaHCO_3_. After separation, the aqueous phase was extracted twice with DCM. The combined organic layers were dried over MgSO_4_ and concentrated in a vacuum. The residue containing nitrosoarene **2a**–**d** was dissolved in acetic acid (50 mL) and appropriate isomer of aminobenzoic acid (1 mmol) was added. The reaction was stirred at room temperature for 24 h and then poured into water. The crude product was collected by filtration and recrystallized from ethyl acetate to afford the analytically pure product. For soluble products, the solvent was evaporated, and the residue was subjected to column chromatography with 1% of acetic acid in DCM used as eluent.

General procedure B: a solution of appropriate azobenzene *m*-, *p*-**3a**–**d** obtained from procedure A (0.3 mmol) in DMF (5 mL) was added to (*R*)-N-deacetyl colchicine (0.15 mmol), HATU (0.15 mmol), and DIPEA (0.9 mmol) under argon atmosphere. The mixture was stirred at room temperature for 4 h and then diluted with ice-cooled water and extracted with ethyl acetate (2 × 40 mL). Combined organic layers were washed with brine dried over MgSO_4_ and evaporated under reduced pressure. The residue was purified by silica gel column chromatography (DCM/MeOH 9:1).

General procedure C: thionyl chloride (5.0 mmol) was added to a solution of 2-aminobenzoic acid (1.05 mmol) in toluene (5 mL), and the mixture was refluxed for 4 h. Next, the solvent was evaporated under reduced pressure to obtain the crude acid chloride as yellow oil, which was used immediately in the next step without any purification. Et_3_N (1.05 mmol) was added to a solution of (*R*)-N-deacetyl colchicine (1.0 mmol) in DCM (10 mL) at 0 °C and stirred for 15 min. The solution of acid chloride in methylene chloride (5 mL) was added dropwise to the latter mixture at 0 °C and stirred overnight. Thereafter, the solvent was removed under reduced pressure and the residue was purified by column chromatography (ethyl acetate/acetone 4:1) to afford intermediate **S1**. In subsequent reactions, nitrosobenzenes **2a**–**d** (obtained as in general procedure A, 0.2 mmol) was dissolved in acetic acid (5 mL) and intermediate **S1** (0.15 mmol) was added in DCM (5 mL). The reaction was stirred at room temperature for 24 h and then the solvent was evaporated. The residue was dissolved in ethyl acetate (10 mL), washed with NaHCO_3_ (2 × 2 mL) water (2 mL), dried over MgSO_4_, and concentrated in a vacuum. The product was purified by silica gel column chromatography (DCM/MeOH 95:5).

### 2.2. Tubulin Polymerisation Assay

Tubulin from porcine brain was purified according to a protocol published previously [54]. The tubulin polymerisation reaction was conducted at 3.5 mg/mL tubulin, in a tubulin polymerisation buffer (80 mM piperazine-N,N′-bis(2-ethanesulfonic acid) (PIPES) pH = 6.9; 0.5 mM EGTA; 2 mM MgCl_2_), in a 96-well plate (100 μL), in a EnSpire^®^ multimode plate reader (PerkinElmer, Turku, Finland) with temperature maintained at 37 °C. Tubulin was initially preincubated for 30 min at room temperature with (*Z*) enriched isomer (green light pre-illuminated) or with all (*E*) isomer (thermally adapted in dark) of *o*-**AzoCol26DF** (10 µM) in buffer with 1% DMSO, without GTP. A sample with 1% DMSO alone was used as a control. GTP was added to the concentration 1 mM, and the change in absorbance at 340 nm was monitored at 15 s intervals for 20 min. A solution of colchicine (5 μM) or cosolvent (DMSO) was used as a control.

### 2.3. Cell Culturing

Cells were cultured in a humidified incubator at 37 °C under 5% CO_2_. Human breast adenocarcinoma (MCF-7) cells were maintained in phenol red-free Dulbecco’s Modified Eagle’s Medium (Thermo Fisher Scientific, Waltham, MA, USA, 11054020) supplemented with 2 mM L-glutamine, and human colorectal carcinoma (HCT116) cells were maintained in the same medium but supplemented with 4.5 g/L glucose and 4 mM L-glutamine. All culture media were supplemented with 1% Penicillin-Streptomycin (Sigma-Aldrich, Burlington, MA, USA, P4333) and 10% fetal bovine serum (Gibco, Billings, MT, USA, 10270-106). Cells were sub-cultured at approximately 70–90% confluency to maintain the culture in the logarithmic growth phase.

### 2.4. MTT Cytotoxicity Assay with Green and Blue Light Irradiation

Cells were seeded in a 96-well plate at the density of 7 × 10^3^ cells per well and allowed to grow for 24 h. Afterwards, the medium was aspirated, and a fresh medium was added (200 μL) with serial dilutions of tested compounds or DMSO at corresponding concentrations as a control. Following 48 h of incubation under 500 ms pulsed green or blue light irradiation every 15 s, the medium was replaced with a medium (100 μL) containing MTT (3-(4,5-dimethylthiazol-2-yl)-2,5-diphenyl-2H-tetrazolium bromide, 0.5 mg/mL) and incubated at 37 °C for 4 h. The formed formazan was dissolved in DMSO (100 μL) and incubated at 37 °C for 10 min. The absorbance was measured at 540 nm. After blank subtraction, the half maximal effective concentration (IC_50_) was calculated by GraphPad Prism software version 7 (GraphPad Software Inc., San Diego, CA, USA). Each independent experiment was performed in triplicate.

### 2.5. Immunofluorescence

Cells were seeded on coverslips on a 24-well plate at a density of 5 × 10^4^ cells per well and allowed to grow overnight. Next, the medium was replaced with a medium containing the *o*-**AzoCol26DF** or the colchicine (without illumination) or DMSO as control and incubated for 24 h under 500 ms pulsed green or blue light irradiation every 15 s. Then, cells were fixed and permeabilised with 100% methanol at −20 °C for 15 min and subsequently washed three times with PBS at room temperature. After 1 h blocking with 3% BSA/PBS at 4 °C, slides were incubated with anti-α tubulin 12G10 antibody (Developmental Studies Hybridoma Bank, University of Iowa, Iowa City, IA, USA) (diluted 1:300 in 3% BSA/PBS) and anti-acetylated α- tubulin antibody (Cell Signalling, Danvers, MA, USA) (diluted 1:1000 in 3% BSA/PBS) overnight at 4 °C. After 3 × 10 min washing with PBS, slides were incubated with AlexaFluor555-conjugated anti-rabbit and AlexaFluor488-conjugated anti-mouse secondary antibodies (diluted 1:400 in 3% BSA/PBS) (Thermo Fisher Scientific, Waltham, MA, USA, A31570) and with DAPI (50 ng/mL) for 1 h at room temperature. After washing (3 × 10 min with PBS), slides were mounted in Fluoromount-G (Southern Biotech., Birmingham, AL, USA). Images were recorded using Leica TCS SP8 (Leica Microsystems, Wetzlar, Germany) confocal microscope and analysed using ImageJ 1.53t software. 

### 2.6. Cell Cycle Stages Analysis

Approximately 10^6^ cells were plated and treated with DMSO, colchicine, or *o*-**AzoCol26DF**. Next, the medium was replaced with a medium containing solution of the *o*-**AzoCol26DF** or solution of the colchicine (without illumination) or DMSO as control and incubated for 24 h under 500 ms pulsed green or blue light irradiation every 15 s, as described above. After 24 h, cells were harvested, fixed with 70% ethanol, and stained with 50 µg/mL propidium iodide with presence of 50 µg/mL RNAse A. Stained cells were immediately analysed in Cytometer BD FACSCalibur (BD, Franklin Lakes, NJ, USA).

## 3. Results and Discussion

### 3.1. The Computational Study

**Figure 2:**
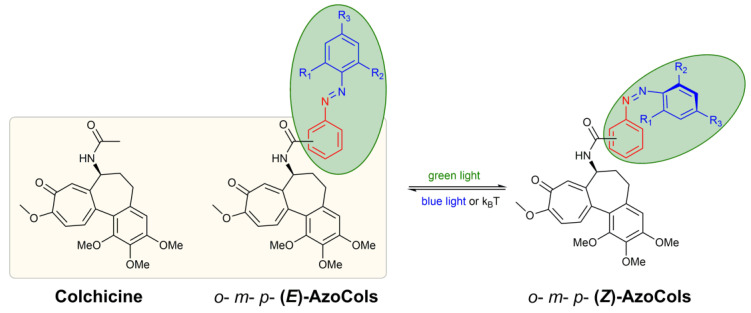
Structure of colchicine and rationale design of azobenzamides-colchicines **AzoCols**.

As mentioned in the introduction, the implementation of fluorine atoms makes it possible to separate the n → π* absorption bands in the UV-VIS spectrum and to separate them from the π → π* band. This is why we first computed and analysed the geometry proposed by using compounds, *o*-, *m*-, *p*-**3a**–**d** for (*E*) and (*Z*) isomers, using the density functional theory (DFT). In the calculations, the B3LYP functional, 6-31G*, and basis set was employed and the continuum model (PCM; Gaussian 03W, see Supporting Information) was used to simulate the effects of the solvent, DMSO [55,56]. This method successfully reproduces the relative energies of the isomers of many azobenzene derivatives, including bridged azobenzenes [57,58]. The SCF energy for the (*E*) and (*Z*) isomers of *o*-, *m*-, *p*-**3a**–**d** is presented in Table S1 in the Supporting Information. For all compounds, the (*E*) isomer has a lower energy (mostly 55.8–59.8 kJ/mol). The smallest differences were observed for (*E*)*-p*-**3d**/(*Z*)-*p*-**3d** and (*E*)*-m*-**3d**/(*Z*)-*m*-**3d** at 43.38 kJ/mol and 45.98 kJ/mol, respectively. Moreover, for compounds (*E*)*-o*-**3b**/(*Z*)-*o*-**3b** and (*E*)*-o*-**3a**/(*Z*)-*o*-**3a**, due to the large spherical hindrance, differences are the largest at 81.08 kJ/mol and 80.61 kJ/mol, respectively. In the case of (*E*)*-o*-**3d**/(*Z*)-*o*-**3d** and (*E*)*-o*-**3c**/(*Z*)-*o*-**3c**, the hydrogen bonds between fluor of first ring and hydrogen of second ring can be observed, such bonds reducing the energy difference between the (*E*) and (*Z*) isomers (56.13 kJ/mol and 56.78 kJ/mol, respectively, see Supporting Information). The geometry of the respective photoswitch has a strong influence on the n → π* excitation energies. To determine whether the conformational changes of the photoswitch structures provide shifts in the excitation energies, we calculated the energy of (*E*) and (*Z*) orbitals HOMO and LUMO for switches *o*-, *m*-, *p*-**3a**–**d** (Table S2). It is known from the framework of MO theory that the lowest excited states of azobenzenes can be quite well described using singly excited n → π* and π → π* configurations [59]. The separation of n and π orbitals by symmetry is easy for the planar (*E*) isomers, and the relevant orbitals, i.e., π, n, and π*, are readily recognised in MO calculations also for the differently shaped (*Z*) isomer. Analysing the obtained data, we can observe that, for all compounds, the π* orbital level is much higher in the (*Z*) isomer relative to the (*E*) isomer. This relates to the fact that in the (*Z*) isomers the π-electron delocalisation is reduced due to the large dihedral angles about the N−C single bonds. The n-orbital energy level in the (*Z*) isomer is also much higher than in the *E*-isomer. This effect is connected with the linearity of the (*E*) isomer and the interaction of the lone pair orbitals on the two neighboring N atoms through bonds. In the case of the nonlinear (*Z*) isomer, the lone pair orbitals interact much more strongly through space. As described by Hecht [24,26], the repulsive interaction of the nitrogen lone pairs increases the n-level in azobenzenes and the introduction of a fluorine atom (σ-electron withdrawing groups) to aromatic ring; especially in ortho position, it should lower the n-orbital energy. It is worth noting that the n → π* excitation energies are very similar for both (*E*) and (*Z*) isomers. This conclusion is consistent with the results of Ali et al. and Hecht et al. obtained for other fluorinated compounds [24,59]. The energy differences of the (*E*) and (*Z*) orbitals HOMO and LUMO are small, ranging from 3.597 eV to 3.935 eV. The information obtained theoretically was verified by synthesis of *o*-, *m*-, *p*-**3a**–**d** compounds (Figure 2) and by measuring their UV-Vis spectra (see Supporting Information).

### 3.2. Design and Synthesis of Azocolchicines

**Scheme 1:**
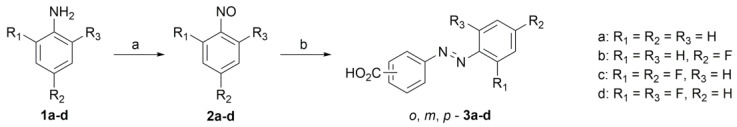
Synthetic route towards azobenzenes *o*-, *m*-, *p*-**3a**–**d**. Reagents and conditions: (a) potassium peroxymonosulfate, DCM/H_2_O (1:1 *v*:*v*); (b) *ortho*-, *meta*-, or *para*- isomer of aminobenzoic acid, AcOH, RT, 24 h.

**Scheme 2:**
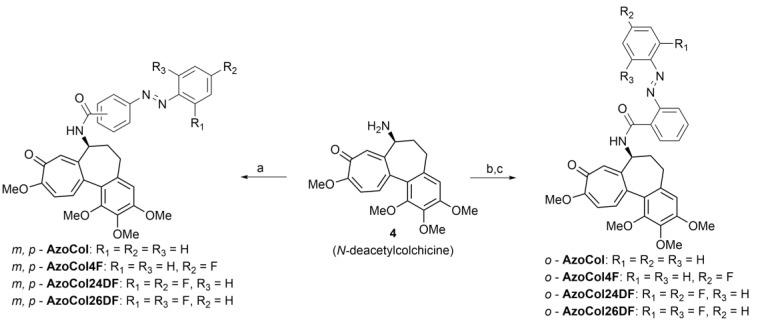
Synthesis strategy towards photoswitchable azobenzamides-colchicines *o*-, *m*-, *p*-**AzoCols**. Reagents and conditions: (a) *m*-, *p*-**3a**–**d**, HATU, DIPEA, DMF, RT, Ar, 4 h; (b) 2-aminobenzoic acid chloride, NEt_3_, DCM, 0 °C to RT, 12; (c) **2a**–**d**, AcOH/DCM (1:1 *v*:*v*), 24 h.

Our synthetic strategy towards photoswitchable azobenzamides-colchicnes **AzoCols** is based on the replacement of the acetoamide group of a well-known and potent microtubule disrupting agent, colchicine, with an azobenzene unit (Figure 2). Previous data suggest that substitutions at this position are well tolerated and do not lead to loss of bioactivity [60,61]. Hence, we anticipated that this approach would maintain the antiproliferative activity and simultaneously allow for precise spatiotemporal control of its activity with light irradiation. Recently, we explored the various synthetic methods to obtain azo compounds. Utilising the optimised conditions, we focused on a one-step method with oxone synthesis [62]. We started from aniline derivatives substituted with a fluorine atom at various positions **1a**–**d** and oxidised them to corresponding nitroso compounds **2a**–**d** by reaction with potassium peroxymonosulfate in biphasic dichloromethane/water solution. Obtained nitroso derivatives were used in Baeyer−Mills reactions with *ortho*-, *meta*-, and *para*- aminobenzoic acid affording azobenzenes *o*-, *m*-, *p*-**3a**–**d** (Scheme 1) [14]. A one-pot condensation reaction between *N*-deacetycolchicine **4** and *meta*- or *para*-**3a**–**d** isomers allowed for a straightforward synthesis of photoswitchable azobenzamides-colchicines *m*-, *p*-**AzoCols**. Unfortunately, reactions with *ortho*-**3a**–**d** resulted in a complicated, inseparable products mixture. Thus, we decided to react **4** with 2-aminobenzoyl chloride in the presence of triethylamine and, in the following step, with nitrosobenzene **2a**–**d**. This synthetic route resulted in the desired target compounds *ortho*-**AzoCols** (Scheme 2).

### 3.3. Photochemical Characterisation

**Figure 3:**
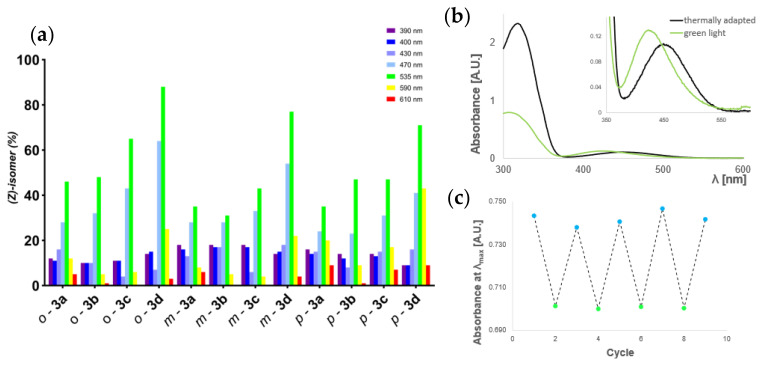
(**a**) Photostationary state (PSS) compositions for azobenzenes *o*-, *m*-, *p*-**3a**–**d** determined by ^1^H or ^19^F NMR analysis (c ≈ 10 mM in DMSO-*d*_6_); (**b**) representative UV-Vis absorption spectra of thermally adapted and green light (535 nm) irradiated 500 μM *o*-**3d** in DMSO; (**c**) representative multiple photoswitching rounds of 50 μM *o*-**3d** in DMSO by pulsed green (535 nm) and blue (430 nm) light illumination.

Photochemical properties of **AzoCols** photoswitches are attributed to azobenzene **3a**–**d** moiety incorporated into the parent pharmacophore structure; therefore, we evaluated the photochemical properties of obtained azobenzenes by NMR spectroscopy and UV-Vis spectrophotometry. As a solvent, we chose DMSO due to its ability to dissolve polar and nonpolar molecules, which is crucial for the analytical methods used in this study. Additionally, its intermediate polarity allows for good approximation of organic and aqueous solvents. Furthermore, in the context of photopharmacology, DMSO is used for stock solution preparation which is illuminated and then diluted into aqueous systems for biological activity assessment [63,64,65]. The photoisomerisation of azobenzenes **3a**–**d** is not altered upon condensation with colchicine (see Supporting Information Figures S2 and S3). We assumed that ultraviolet light would cause photocatalyzed degradation of azobenzamides-colchicines **AzoCols**. Indeed, irradiation of *p*-**AzoCol4F** with UV light (365 nm) lead to the formation of a complex mixture of products (see Supporting Information Figure S4). This result confirms that ultraviolet light is incompatible with photoswitchable ligands based on the colchicine structure. Therefore, we determined the distribution of (*E*) and (*Z*) isomers for azobenzenes *o*-, *m*-, *p*-**3a**–**d** at the photostationary state (PSS) under constants illumination with selected wavelengths of the visible spectrum (390–610 nm) by ^1^H or ^19^F NMR analysis (see Supporting Information Figures S6–S17). The obtained results are summarised in Figure 3a. The green light (505–535 nm) induced (*E*) → (*Z*) photoconversions, affording the highest PSS ratios. On the other hand, blue light (390–430 nm) induced reverse (*Z*) → (*E*) photoconversion, affording low PSS compositions. The lowest PSS percentages were obtained for compounds without a fluorine substituent, i.e., *o*-, *m*-, *p*-**3a** (46, 35, and 35%, respectively), while introducing a fluorine atom at 4- and 2,4-positions in compounds *o*-, *m*-, *p*-**3b**–**c** caused only a slight increase in PSS ratios. The azobenzenes bearing an ortho-fluorine substituent, i.e., *o*-, *m*-, *p*-**3d**, displayed the highest PSS compositions (88, 77, and 71%, respectively). After selection of optimal wavelengths, we acquired UV-Vis absorption spectra for azobenzenes *o*-, *m*-, *p*-**3a**–**d** in dimethyl sulfoxide at 500 μM concentrations for visualisation and analysis of weak n–π* bands. Spectroscopic data are summarised in Table 1. In general, the strong π–π* transition band was observed at 315–330 nm and the weaker n–π* band at approximately 445 nm for (*E*) isomers. After illumination with green light (causing photoconversion to the (*Z*) isomer), a decrease in π–π* band intensity and an increase in n–π* bands were observed. Most importantly, for compounds *o*-, *m*-, *p*-**3d**, due to the introduction of an *ortho*-fluoro substituent, causing stabilisation of nonbonding electron pairs of the azo-bond, significant separation of the n → π* transition band of the (*E*) and (*Z*) isomers (around 30 nm) was observed (Figure 3b), allowing for selective addressing of both isomers with visible light. Multiple cycles of photoreversible switching under alternating green and blue light irradiation without noticeable photobleaching or degradation confirmed repeatable and robust photochromic conversion of the obtained azobenzenes (Figure 3c). Moreover, we checked photochemical stability of *o*-**AzoCol26DF** and corresponding azobenzene *o*-**3d** in cell growth media at conditions similar to photopharmacological assays (high glucose medium supplemented with 4 mM L-glutamine, 500 ms pulses of green or blue light every 15 s for 48 h, 10% of DMSO). No changes in spectrum indicating degradation were observed. The only change in spectrum was attributed to (*E*) → (*Z*) photoconversions (see Supporting Information Figure S5). All photoswitches displayed substantially slower spontaneous (*Z*) → (*E*) relaxation (the half-life at 37 °C varied from 5 h to >48 h) than the biological assays timescale. Stability in organic and aqueous media, near-ideal photochemical characteristics, and bidirectional photoswitching showed that the obtained molecular switches can be used in photopharmacological assays or in vivo.

### 3.4. Photocontrollable in Cellulo Studies

Colchicine is a potent microtubule polymerisation inhibitor leading to mitotic arrests and, as a consequence, cell death [66]. We expected photoswitchable azobenzamides-colchicnes **AzoCols** to show similar antiproliferative activity. Therefore, we decided to evaluate in cellulo cytotoxicity for twelve obtained colchicine analogues at the most favourable illuminating conditions (430 nm for predominantly (*E*) and 535 nm for predominantly (*Z*) isomer, respectively). For irradiation of cell cultures, we used self-built arrays of 24 low-power light-emitting diodes (LED) controlled by the Adurino board (see Supporting Information). Such an automated system allowed for precise pulsed illumination (500 ms pulses of light every 15 s in our experiments) during long-term assays. Most importantly, it has been proven to be compatible with cell culturing conditions [37,40,42,45,46]. We first screened for bioactivity on an MCF-7 cell line as a model for human breast adenocarcinoma (N = 1). For lead compound exhibiting light-dependent cytotoxicity, we further expanded our research to include a HCT116 cell line (human colorectal carcinoma) and a HKE293 cell line (human embryonic kidney). The obtained results are summarised in Table 2. Notably, all **AzoCols** showed potent antiproliferative activity (IC_50_ ranging from 28 to 187 nM), clearly demonstrating that incorporation of azobenzene moiety neither suppress binding to tubulin nor disrupt permeation through the cytoplasmic membrane. The *meta*-**AzoCols** set of compounds displayed equipotent bioactivity under illumination with green or blue light. In contrast, throughout *para*- and *ortho*- isomers, *p*-**AzoCol24DF** and *o*-**AzoCol26DF** showed dissimilar cytotoxicity, dependent on the irradiation conditions. However, for *o*-**AzoCol** and *o*-**AzoCol4F**, we obtained somewhat inconsistent results. In the predominantly (*Z*) isomer state (green light illumination), *p*-**AzoCol24DF** and *o*-**AzoCol26DF** showed higher potency than in the predominantly (*E*) isomer state (blue light illumination). The most promising compound, *o*-**AzoCol26DF**, displayed c.a. a double potency shift on HCT116 cells upon illumination with green light.

### 3.5. o-**AzoCol26DF** Disrupt Tubulin Polymerisation and Cellular Microtubule Organisation in Light-Dependent Manner

**Figure 4:**
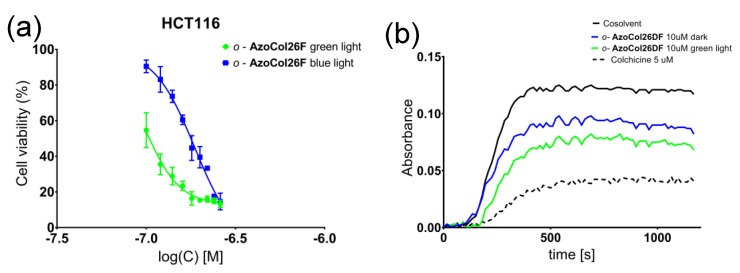
(**a**) Cell viability curves for *o*-**AzoCol26DF** under green (535 nm) or blue (430 nm) light irradiation displayed approximately a double shift in potency on HCT116 cells (MTT assay, N = 2); (**b**) light-dependent influence of *o*-**AzoCol26DF** (10 μM) on tubulin polymerisation in cell-free assays. Solution of colchicine (5 μM) or cosolvent (DMSO) used as a control (curves represent mean from two replicates).

**Figure 5:**
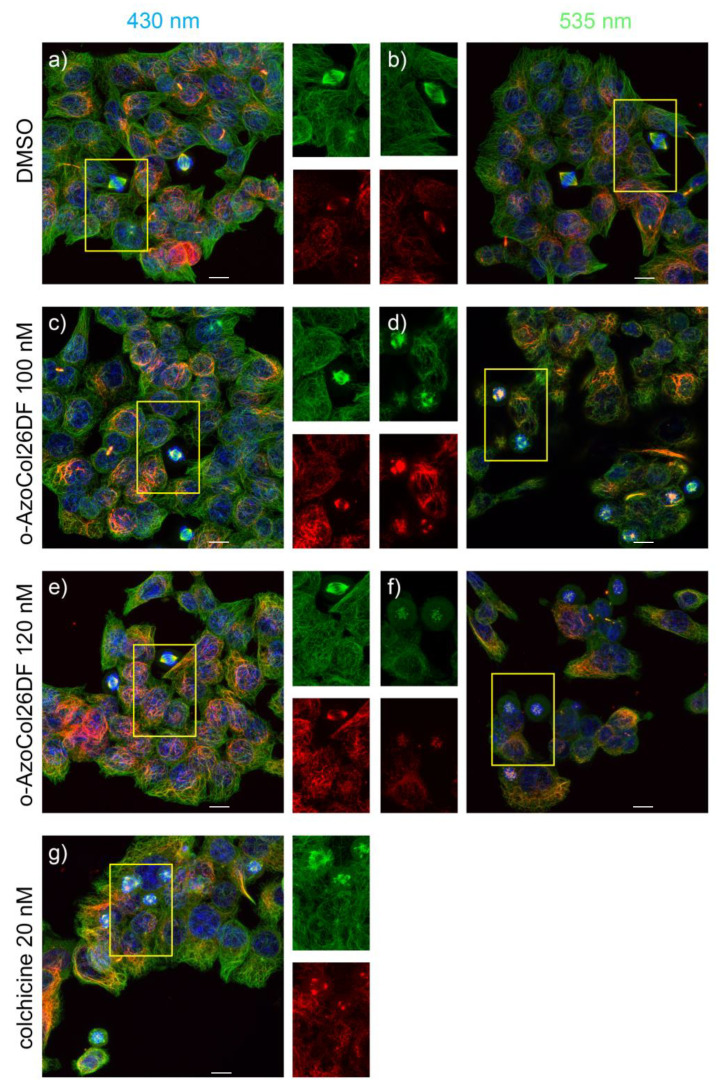
Analysis of HTC116 cells upon treatment with DMSO, *o*-**AzoCol26DF,** or colchicine. Cells were stained with 12G10 monoclonal antibody against total α-tubulin (green) and with anti-acetylated α-tubulin antibody (red). Nuclei stained with DAPI (blue). Insets show magnification of the mitotic spindle structure. Bars in all subfigures = 10 μm. Control cells (DMSO) illuminated with blue (**a**) or green (**b**) light, respectively. Cells show dense microtubular cytoskeleton and normal cell shape. (**c**) Cells incubated with 100 nM *o*-**AzoCol26DF** illuminated with blue light. Notice that microtubular cytoskeleton resembles control. (**d**) Cells incubated with 100 nM *o*-**AzoCol26DF** illuminated with green light. Mitotic spindles frequently are aberrant, showing multipolarity and misarrangement. (**e**) Cells incubated with 120 nM *o*-**AzoCol26DF** illuminated with blue light. (**f**) Cells incubated with 120 nM *o*-**AzoCol26DF** illuminated with green light. Many mitotic cells with disarranged and/or nearly completely depolymerized mitotic spindle are present. (**g**) Cells incubated with 20 nM colchicine. The arrangement of mitotic spindles is similar as in cell treated with 100 nM *o*-**AzoCol26DF** illuminated with green light.

To further explore the molecular mechanism of *o*-**AzoCols** light-dependent cellular activity, we examined the influence of *o*-**AzoCol26DF** on tubulin polymerisation in cell-free assays with purified tubulin. In the mainly (*Z*) isomer state (green light), 10 μM solution of *o*-**AzoCol26DF** resulted in ca. 40% inhibition of polymerisation over control (referenced to DMSO as 0%), while the thermally adapted state yielded only a 30% polymerisation inhibition. In comparison, 5 μM solution of colchicine caused ca. 70% of tubulin polymerisation inhibition (Figure 4b). It is worth noticing that this is a highly nonlinear assay in an environment far from cellular conditions. Thus, these results cannot be used for evaluating potencies, but rather to shed light on the mechanism of action as microtubule destabilisers, as the parent colchicine is. To verify the effect of *o*-**AzoCol26DF** on the microtubular cytoskeleton in vivo, we incubated HTC116 cells for 24 h with either DMSO (control) or *o*-**AzoCol26DF**. During the incubation, all cell samples were illuminated with green or blue light as described in the Material and Methods section. As an additional positive control, we used cells treated with 20 nm colchicine without light illumination. In both control samples, non-dividing cells had a dense, well-expanded network of interphase microtubules, while dividing cells formed a bipolar mitotic spindle (Figure 5a,b). A similar cell phenotype was observed in *o*-**AzoCol26DF**-treated, blue-light-illuminated cells (Figure 5c). In cells treated with 100 nM *o*-**AzoCol26DF** and activated by the green light, the interphase microtubular cytoskeleton was only slightly less prominent with respect to the control. However, we observed more dividing cells, and the mitotic spindle structure was frequently abnormal, with more than two spindle poles or misaligned microtubules (Figure 5d). This phenotype was even more pronounced in 120 nM *o*-**AzoCol26DF**-treated and green-light-illuminated cells (Figure 5f). Importantly, similar changes were observed in cells treated with 20 nM colchicine (Figure 5g).

**Figure 6:**
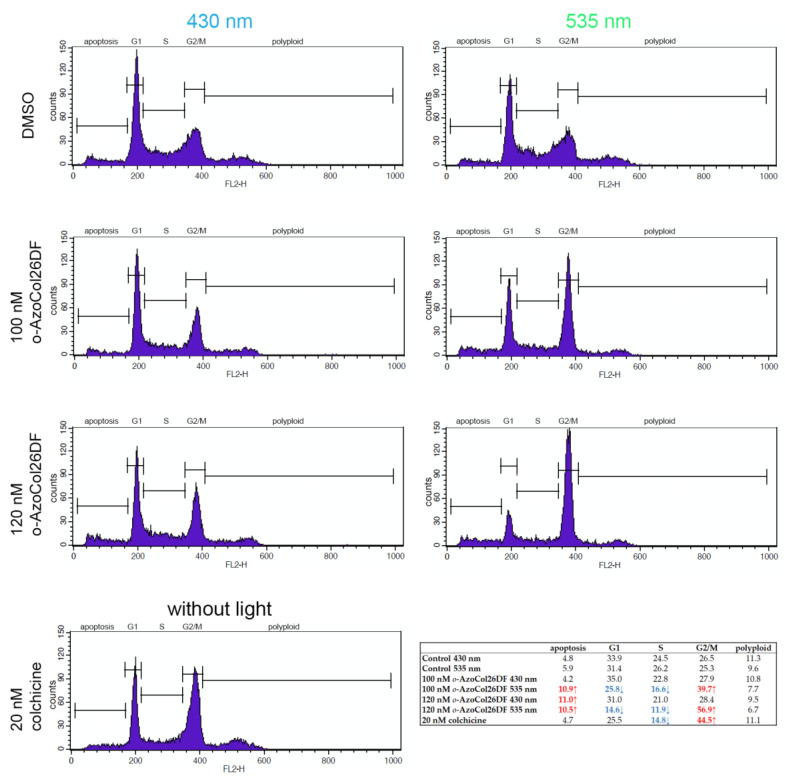
Analysis of cell cycle stages of HTC116 cells upon treatment with DMSO, *o*-**AzoCol26DF**, or colchicine and blue or green light exposure. Graphs show representative flow cytometry analysis for each sample. In the table, the mean of at least two experiments for each sample are included. The increase with respect to the control is marked by the red color and upwards arrow, while the decrease is marked by the blue color and downwards arrow.

To verify these observations, we determined the distribution of cell cycle stages using FACS analysis (Figure 6). Under the control treatment, within the HTC116 samples, ~31–34% cells were in G1 phase, 22–26% in S phase, and 25–26% in G2/M phases. We also observed some polyploid and apoptotic cells (Figure 6). In 100 nM *o*-**AzoCol26DF**-treated and blue-light-illuminated cells, the distribution of cell cycle stages was similar to that in control cells; however, we observed a higher number of apoptotic cells. In contrast, in green-light-illuminated 100 nM *o*-**AzoCol26DF**-treated cells, the number of G2/M cells increased to ~40%, while the number of apoptotic cells was the same as that in blue-light-illuminated cells. Treatment of cells with 20 nM colchicine raised the number of G2/M cells to ~45%, indicating that, likely, 100 nM *o*-**AzoCol26DF** has similar activity to 20 nM colchicine. Importantly, the increase in the concentration of *o*-**AzoCol26DF** to 120 nM elevated the number of G2/M cells to ~60% in green-light-illuminated cells, while in the corresponding blue-light-treated cells G2/M cell numbers remained at 29%.

To summarise, under used conditions, we were able to control the activity of *o*-**AzoCol26DF** and, consequently, disrupt microtubular cytoskeleton leading to mitotic arrest and, eventually, cell death.

## 4. Conclusions

In this study, we successfully designed and synthesised a set of novel photoswitchable colchicine-based microtubule dynamics disrupting agents. The developed photoswitches can be photoisomerised by visible light instead of UV light, which is used in classical photopharmaceutical reagents. This is crucial due to colchicine instability under UV light irradiation. For the lead compound *o***-AzoCol26DF**, we demonstrated light-dependent cytotoxicity in both the HCT116 and the MCF-7 cancerous cell lines. Inhibition of purified tubulin polymerization, as well as disruption of microtubule organization, support proof-of-concept of using **AzoCols** as photoswitchable microtubule dynamics disrupting agents. In summary, we have proven that **AzoCols** provide the basis for further improvement and development of the novel class of photoswitchable colchicine-based microtubule polymerisation inhibitors that could be used in future studies and applications.

## Figures and Tables

**Table 1:** Spectroscopic data for 500 μM solution of *o*-, *m*-, *p*-**3a-d** in DMSO at 25 °C. The photostationary state (PSS) composition was determined by ^19^F or ^1^H NMR (c ≈ 10 mM in DMSO-*d*_6_). Thermal relaxation half-life (t_1/2_) was measured for 100 μM DMSO solutions at 37 °C.

	(*E*)-isomer ^a^		(*Z*)-isomer ^b^				
Compound	λ_max_ (π−π*) [nm]	λ_max_ (n−π*) [nm]	λ_max_ (n−π*) [nm]	Δλ (n−π*) [nm]	t_1/2_[h]	PSS_(*E*)_^c^ (%)	PSS_(*Z*)_^d^ (%)
*o*-**3a**	324	448	440	8	6	95	46
*o*-**3b**	326	445	437	8	5	99	48
*o*-**3c**	330	444	433	11	23	96	66
*o*-**3d**	315	450	420	30	>48	97	88
*m*-**3a**	321	437	429	8	>48	94	35
*m*-**3b**	322	431	423	8	>48	95	32
*m*-**3c**	326	440	423	17	>48	96	46
*m*-**3d**	315	448	417	31	>48	96	77
*p*-**3a**	330	447	440	7	17	91	35
*p*-**3b**	330	446	440	6	16	99	47
*p*-**3c**	334	448	437	11	23	93	47
*p*-**3d**	322	454	421	33	>48	91	71

^a^ Thermally adapted; ^b^ PSS after irradiation with green light (535 nm); ^c^ Percentage of (*E*) isomer at 430 nm PSS; ^d^ Percentage of (*Z*) isomer at 535 nm PSS.

**Table 2:** Light-dependent cytotoxicity of **AzoCols** from MTT assay.

Compound	Cell Line	IC_50_ Blue Light (nM)	IC_50_ Green Light (nM)	IC Ratio
*p*-**AzoCol**	MCF-7 (N = 1)	48 ± 2	44 ± 2	1.1
*p*-**AzoCol4F**	MCF-7 (N = 1)	44 ± 1	43 ± 1	1.0
*p*-**AzoCol24DF**	MCF-7 (N = 3)	50 ± 1	36 ± 1	1.4
*p*-**AzoCol26DF**	MCF-7 (N = 1)	55 ± 1	54 ± 1	1.0
*m*-**AzoCol**	MCF-7 (N = 1)	31 ± 2	27 ± 1	1.1
*m*-**AzoCol4F**	MCF-7 (N = 1)	42 ± 1	40 ± 1	1.1
*m*-**AzoCol24DF**	MCF-7 (N = 1)	49 ± 1	47 ± 1	1.0
*m*-**AzoCol26DF**	MCF-7 (N = 1)	45 ± 2	46 ± 4	1.0
*o*-**AzoCol**	MCF-7 (N = 1)	nc	nc	nc
*o*-**AzoCol4F**	MCF-7 (N = 1)	nc	nc	nc
*o*-**AzoCol24DF**	MCF-7 (N = 1)	183	174	1.1
*o*-**AzoCol26DF**	MCF-7 (N = 3)	184 ± 4	126 ± 2	1.5
*o*-**AzoCol26DF**	HCT116 (N = 2)	187 ± 9	97 ± 2	1.9
*o*-**AzoCol26DF**	HEK293 (N = 3)	>250	>250	-
**Colchicine**	MCF-7	12 ^a^	-	-
**Colchicine**	HCT116	11 ^b^	-	-

^a^ without illumination ref. [67].; ^b^ without illumination ref. [68].; nc—not calculated.

## Data Availability

The data presented in this study are available in Supplementary Materials.

## Supplementary Materials

The following supporting information can be downloaded at: https://www.mdpi.com/article/10.3390/cells12141866/s1, Chemistry: General information and general procedures; Characterization of obtained compounds; NMR & UV-VIS Data; Photostationary State (PSS) Analysis; Photoswitching and Half-Life Determination.; Biological assays; Copies of NMR spectra and Computational calculations.
