# Supplementary material for: First-in-Class Colchicine-Based Visible Light Photoswitchable Microtubule Dynamics Disrupting Agent
Source: Cells. 2023 Jul 16;12(14):1866. doi: 10.3390/cells12141866 (PMC10378023; doi:10.3390/cells12141866)
Supplement: Supplementary file 1 [file cells-12-01866-s001.zip › cells-2439955-supplementary.pdf]

First-in-class colchicine-based visible light photoswitchable microtubule dynamics disrupting agent.

Filip Borys [1,2,*], Piotr Tobiasz [1], Hanna Fabczak [2], Ewa Joachimiak [2] and Hanna Krawczyk [1,*]

[1] Department of Organic Chemistry, Faculty of Chemistry, Warsaw University of Technology, Noakowskiego 3 Street, 00-664 Warsaw, Poland
[2] Nencki Institute of Experimental Biology, Polish Academy of Sciences, 3 Pasteur Street, 02-093 Warsaw, Poland
* Correspondence: filip.borys@pw.edu.pl; hanna.krawczyk@pw.edu.pl

Supporting Information

Table of Contents

1. **Chemistry: General information and general procedures**
2. **Characterization of obtained compounds**
3. **NMR & UV-VIS Data – Photostationary State (PSS) Analysis, Photoswitching and Half-Life Determination**
4. **Biological assays**
5. **Copies of NMR spectra**
6. **Computational calculations**

CHEMISTRY

General Information

Unless otherwise stated, all the chemicals were obtained from commercial supplier and were used without other purification. The solvents were of analytical grade and if needed dried and distilled before use. All of the reactions were monitored by TLC on Merck silica gel plates 60 F254. All compounds were purified by column chromatography on Merck silica gel 60/230– 400 mesh or by flash chromatography. All the spectra were recorded using a Varian VNMRS spectrometer operating at 11.7 T and Varian Mercury VX 9.4 T magnetic field. The residual signals of DMSO-d_6 (2.54 ppm) in the ^{1}H NMR and the DMSO-d_6 signal (39.52 ppm) in the ^{13}C NMR spectra were used as the chemical shift references. In the ^{19}F NMR the CF_3CO_2H signal (-76.55 ppm) was used as the chemical shift references. Coupling constants are reported in Hertz. All the proton spectra were recorded using the standard spectrometer software and parameters. High resolution mass spectrometry (HRMS) spectra were recorded on a QExactive mass spectrometer (Thermo Fisher Scientific). (*R*)-N-deacetyl colchicine was synthesized according to protocol published earlier [3].

General procedure A:

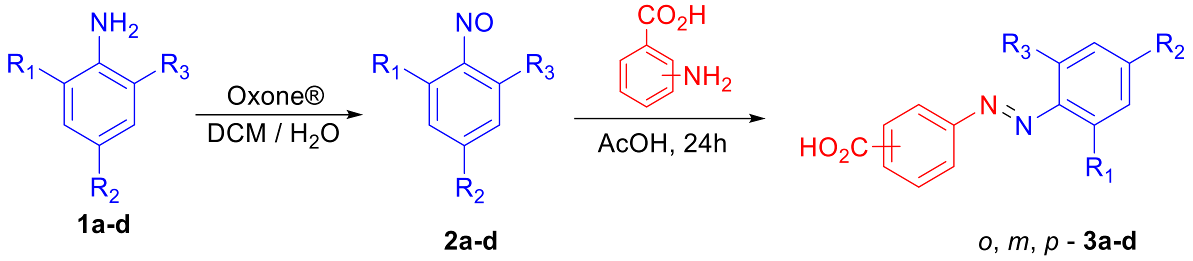

Scheme S1. The synthetic pathway towards compounds *o*-, *m*-, *p*-**3a-d**.

To the solution of aniline derivative **1a-d** (1mmol) in dichloromethane (20 mL) the aqueous solution (35 mL) of oxone® (3.5 mmol) was added dropwise. The reaction mixture was vigorous stirred at room temperature under argon. After disappearance of starting material (analysed by TLC), the reaction was quenched by addition of $NaHCO_3$. After separation, aqueous phase was extracted with DCM twice. The combined organic layers were dried over $MgSO_4$ and concentrated in vacuum. The residue containing nitrosoarene **2a-d** was dissolved in acetic acid (50 mL) and appropriate isomer of aminobenzoic acid (1 mmol) was added. The reaction was stirred at room temperature for 24 h and then poured into water. The crude product was collected by filtration and recrystallized from ethyl acetate to afford the analytically pure product. For soluble products, the solvent was evaporated and the residue was sub*j*ected to column chromatography with 1% of acetic acid in DCM used as eluent.

General procedure B:

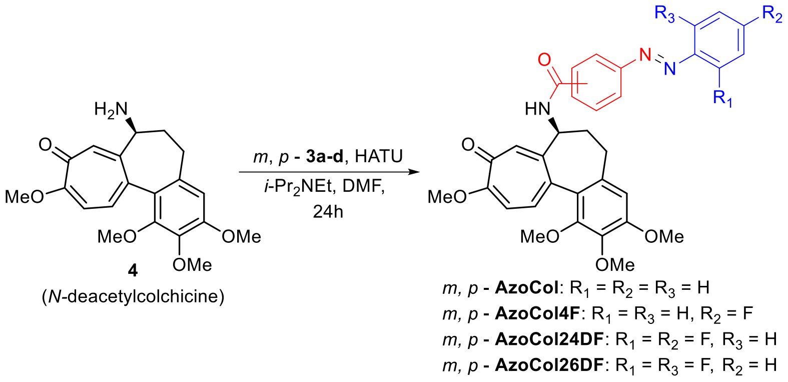

Scheme S2. The synthetic pathway towards compounds *m*-, *p*-**AzoCols**

A solution of appropriate azobenzene *m*-, *p*-**3a-d** obtained from procedure A (0.3 mmol) in DMF (5 mL) was added to (*R*)-N-deacetyl colchicine (0.15 mmol), HATU (0.15 mmol) and DIPEA (0.9 mmol) under argon atmosphere. The mixture was stirred at room temperature for 4 h and then diluted with ice-cooled water and extracted with ethyl acetate (2 × 40 mL). Combined organic layers were washed with brine dried over $MgSO_4$ and evaporated under reduced pressure. The residue was purified by silica gel column chromatography (DCM/MeOH 9:1).

General procedure C:

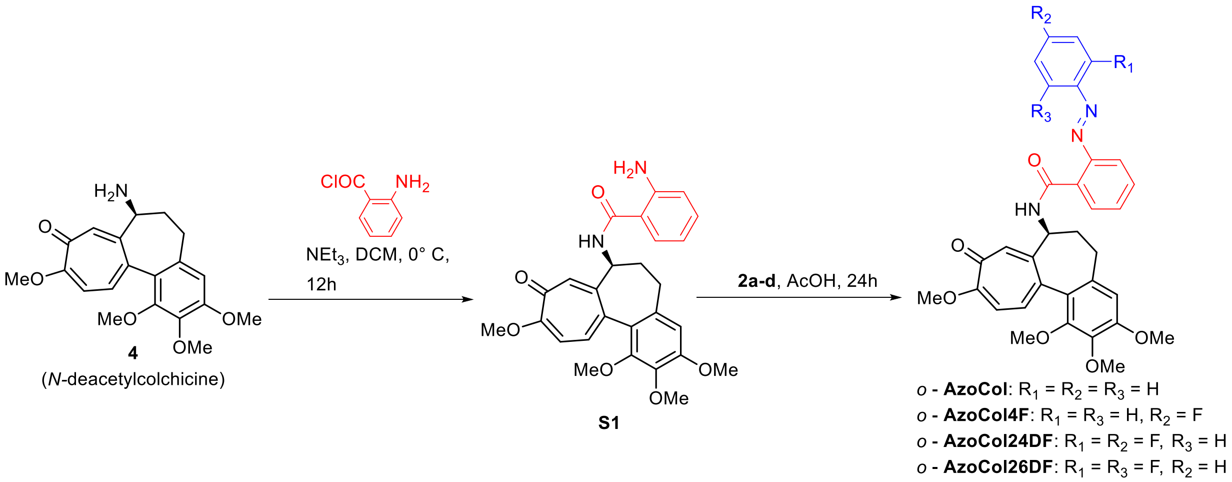

Scheme S3. The synthetic pathway towards compounds *o*-**AzoCols**

To a solution of 2-aminobenzoic acid (1.05 mmol) in toluene (5 mL) thionyl chloride (5.0 mmol) was added and the mixture was refluxed for 4 h. Next, the solvent was evaporated under reduced pressure to obtain the crude acid chloride as yellow oil, which was used immediately in the next step without any purification. To a solution of (*R*)-N-deacetyl colchicine (1.0 mmol) in DCM (10 mL) was added Et_3N (1.05 mmol) at 0°C and stirred for 15 min. To this mixture, the solution of acid chloride in methylene chloride (5 mL) was added dropwise at 0°C and stirred overnight. Thereafter, the solvent was removed under reduced pressure and the residue was purified by column chromatography (ethyl acetate/acetone 4:1) to afforded intermediate **S1**. In subsequent reaction nitrosobenzenes **2a-d** (obtained as in general procedure A, 0.2 mmol) was dissolved in acetic acid (5 mL) and intermediate **S1** (0.15 mmol) was added in DCM (5 mL). Reaction was stirred at room temperature for 24 h and then solvent was evaporated. The residue was dissolved in ethyl acetate (10 mL) and washed with $NaHCO_3$ (2 × 2 mL) water (2 mL) and dried over $MgSO_4$ and concentrated in vacuum. The product was purified by silica gel column chromatography (DCM/MeOH 95:5).

Characterization of obtained compounds:

(*E*)-2-(phenyldiazenyl)benzoic acid (*o*-**3a**)

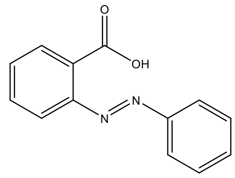

Compound *o*-**3a** was obtained according to general procedure A in 44% yield
TLC: Rf = 0.40 (DCM),
^{1}H NMR (500 MHz, DMSO-d6) δ 13.14 (bs, 1H), 7.87 – 7.84 (m, 2H), 7.80 (dd, *J* = 7.6, 1.4 Hz, 1H), 7.70 – 7.65 (td *J* = 7.5, 1.5 Hz, 1H), 7.65 – 7.54 (m, 5H).
^{13}C NMR (126 MHz, DMSO-d6) δ 168.2 (s), 152.0 (s), 150.8 (s), 131.8 (s), 131.7 (s), 130.4 (s), 130.4 (s), 129.5 (s), 129.2 (s), 122.7 (s), 118.0 (s).
HRMS (HESI): m/z 227.0815 [227.0815 calcd. for $C_{13}H_{11}N_2O_2^+$ (M+H+)].

(E)-2-((4-fluorophenyl)diazenyl)benzoic acid (*o*-**3b**)

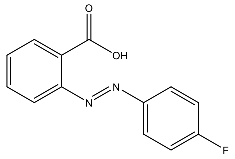

Compound *o*-**3b** was obtained according to general procedure A in 37% yield
TLC: Rf = 0.47 (DCM),
^{1}H NMR (500 MHz, DMSO-d6) δ 13.15 (s, 1H), 7.95 – 7.90 (m, 2H), 7.80 (dd, *J* = 7.6, 1.2 Hz, 1H), 7.70 (td *J* = 7.5, 1.5 Hz, 1H), 7.60 (td, *J* = 7.5, 1.3 Hz, 1H), 7.55 (dd, *J* = 7.9, 1.0 Hz, 1H), 7.49 – 7.43 (m, 2H).
^{13}C NMR (126 MHz, DMSO-d6) δ 168.2 (s), 164.0 (d, *J* = 250.4 Hz), 150.7 (s), 148.8 (d, *J* = 2.8 Hz), 131.7 (s), 130.5 (s), 130.4 (s), 129.3 (s), 125.1 (d, *J* = 9.6 Hz), 118.0 (s), 116.5 (d, *J* = 23.1 Hz).
^{19}F NMR (470 MHz, DMSO-d6) δ -111.9 (tt, *J* = 8.6, 5.3 Hz).
HRMS (HESI): m/z 245.0720 [245.0721 calcd. for $C_{13}H_{11}N_2O_2^+$ (M+H+)].

(*E*)-2-((2,4-difluorophenyl)diazenyl)benzoic acid (*o*-**3c**)

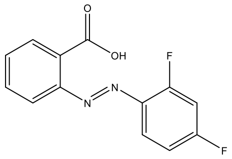

Compound *o*-**3c** was obtained according to general procedure A in 28% yield
TLC: Rf = 0.31 (DCM),
^{1}H NMR (500 MHz, DMSO-d6) δ 13.19 (s, 1H), 7.85 – 7.80 (m, 1H), 7.72 (td, *J* = 8.9, 6.4 Hz, 1H), 7.70 – 7.66 (m, 1H), 7.64 – 7.56 (m, 2H), 7.55 – 7.51 (m, 1H), 7.28 (dddd, *J* = 9.2, 8.2, 2.7, 1.3 Hz, 1H).

^{13}C NMR (126 MHz, DMSO-d6 δ 168.0 (s), 164.4 (dd, *J* = 252.9, 12.0 Hz), 159.9 (dd, *J* = 259.0, 13.1 Hz), 150.9 (s), 137.1 (dd, *J* = 7.1, 3.8 Hz), 131.8 (s), 130.8 (s), 130.6 (s), 129.4 (s), 119.5 (dd, *J* = 10.8, 1.2 Hz), 118.0 (s), 112.7 (dd, *J* = 23.0, 3.7 Hz), 105.7 (dd, *J* = 26.7, 23.8 Hz).
^{19}F NMR (470 MHz, DMSO-d6) δ -107.4 (tdd, *J* = 17.3, 9.2, 6.5 Hz), -123.1 (dt, *J* = 19.6, 9.2 Hz).
HRMS (HESI): m/z 263.0626 [263.0627 calcd. for $C_{13}H_{11}N_2O_2^+$ (M+H+)].
(*E*)-2-((2,6-difluorophenyl)diazenyl)benzoic acid (*o*-**3d**)

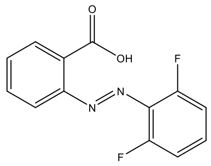

Compound *o*-**3d** was obtained according to general procedure A in 34% yield
TLC: Rf = 0.39 (DCM),
^{1}H NMR (500 MHz, DMSO-d6) δ 13.15 (bs, 1H), 7.80 (dd, *J* = 7.3, 1.7 Hz, 1H), 7.71 – 7.57 (m, 3H), 7.55 – 7.51 (m, 1H), 7.38 – 7.31 (m, 2H).
^{13}C NMR (126 MHz, DMSO-d6) δ 168.0 (s), 154.9 (dd, *J* = 258.5, 4.2 Hz), 151.0 (s), 132.5 (t, *J* = 10.5 Hz), 131.6 (s), 131.4 (s), 130.9 (s), 130.1 (t, *J* = 10.1 Hz), 129.2 (s), 117.8 (s), 113.1 (dd, *J* = 19.7, 3.8 Hz).
^{19}F NMR (470 MHz, DMSO-d6) δ -125.0 (dd, *J* = 9.4, 6.3 Hz).
HRMS (HESI): m/z 263.0625 [263.0627 calcd. for $C_{13}H_{11}N_2O_2^+$ (M+H+)].

(*E*)-3-(phenyldiazenyl)benzoic acid (*m*-**3a**)

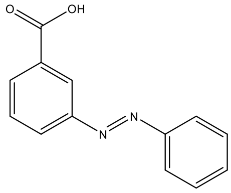

Compound *m*-**3a** was obtained according to general procedure A in 38% yield
TLC: Rf = 0.22 (DCM),
^{1}H NMR (500 MHz, DMSO-d6) δ 13.29 (bs, 1H), 8.38 (t, *J* = 1.6 Hz, 1H), 8.16 (ddd, *J* = 7.9, 2.0, 1.2 Hz, 1H), 8.13 (ddd, *J* = 7.7, 1.6, 1.2 Hz, 1H), 7.98 – 7.90 (m, 2H), 7.75 (t, *J* = 7.8 Hz, 1H), 7.67 – 7.57 (m, 3H).
^{13}C NMR (126 MHz, DMSO-d6) δ 166.6 (s), 151.9 (s), 151.8 (s), 132.1 (s), 132.0 (s), 131.8 (s), 123.0 (s), 129.5 (s), 127.4 (s), 122.7 (s), 122.2 (s).
HRMS (HESI): m/z 227.0813 [227.0815 calcd. for $C_{13}H_{11}N_2O_2^+$ (M+H+)].

(*E*)-3-((4-fluorophenyl)diazenyl)benzoic acid (*m*-**3b**)

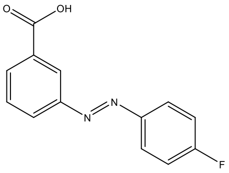

Compound *m*-**3b** was obtained according to general procedure A in 17% yield
TLC: Rf = 0.10 (DCM),
^{1}H NMR (500 MHz, DMSO-d6) δ 13.33 (s, 1H), 8.42 – 8.31 (m, 1H), 8.17 – 8.08 (m, 2H), 8.07 – 7.98 (m, 2H), 7.74 (t, *J* = 7.8 Hz, 1H), 7.50 – 7.40 (m, 2H).
^{13}C NMR (126 MHz, DMSO-d6) δ 166.6 (s), 164.1 (d, *J* = 250.4 Hz), 151.7 (s), 148.6 (d, *J* = 2.9 Hz), 132.2 (s), 131.9 (s), 130.0 (s), 127.3 (s), 125.2 (d, *J* = 9.4 Hz), 122.2 (s), 116.5 (d, *J* = 23.0 Hz).
^{19}F NMR (470 MHz, DMSO-d6) δ -111.7 (tt, *J* = 8.5, 5.4 Hz).

HRMS (HESI): m/z 245.0720 [245.0721 calcd. for $C_{13}H_{10}FN_2O_2^+$ (M+H+)].

(*E*)-3-((2,4-difluorophenyl)diazenyl)benzoic acid (*m*-**3c**)

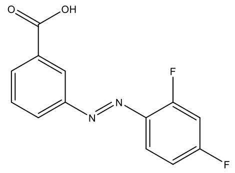

Compound *m*-**3c** was obtained according to general procedure A in 32% yield
TLC: Rf = 0.11 (DCM),
^{1}H NMR (500 MHz, DMSO-d6) δ 13.36 (bs, 1H), 8.36 (t, *J* = 1.7 Hz, 1H), 8.18 – 8.11 (m, 2H), 7.86 (td, *J* = 8.9, 6.4 Hz, 1H), 7.75 (t, *J* = 7.8 Hz, 1H), 7.64 – 7.56 (m, 1H), 7.31 – 7.23 (m, 1H).
^{13}C NMR (101 MHz, DMSO) δ 166.9 (s), 164.7 (dd, *J* = 253.2, 12.1 Hz), 160.2 (dd, *J* = 259.3, 13.1 Hz), 152.1 (s), 137.0 (dd, *J* = 6.7, 3.9 Hz), 132.5 (s), 132.3 (s), 130.2 (s), 127.7 (s), 122.7 (s), 119.4 (d, *J* = 10.8 Hz), 112.7 (dd, *J* = 22.9, 3.1 Hz), 105.7 (t, *J* = 26.0 Hz).
^{19}F NMR (470 MHz, DMSO-d6) δ -107.3 (tdd, *J* = 17.3, 9.3, 6.5 Hz), -122.9 (dt, *J* = 18.9, 9.3 Hz).
HRMS (HESI): m/z 263.0625 [263.0627 calcd. for $C_{13}H_9F_2N_2O_2^+$ (M+H+)].

(*E*)-3-((2,6-difluorophenyl)diazenyl)benzoic acid (*m*-**3d**)

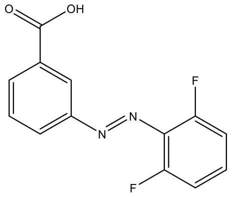

Compound *m*-**3d** was obtained according to general procedure A in 27% yield
TLC: Rf = 0.11 (DCM),
^{1}H NMR (500 MHz, DMSO-d6) δ 13.39 (s, 1H), 8.33 (t, *J* = 1.8 Hz, 1H), 8.20 – 8.16 (m, 1H), 8.14 (ddd, *J* = 7.9, 2.1, 1.2 Hz, 1H), 7.77 (t, *J* = 7.8 Hz, 1H), 7.61 (tt, *J* = 8.5, 6.1 Hz, 1H), 7.40 – 7.32 (m, 2H).
^{13}C NMR (126 MHz, DMSO-d6) δ 167.1 (s), 155.3 (dd, *J* = 258.0, 3.6 Hz), 152.8 (s), 133.2 (s), 132.8 (t, *J* = 10.5 Hz), 132.5 (s), 130.6 (s), 130.5 (t *J* = 10.1 Hz), 127.81 (s), 122.38 (s), 114.43 – 112.33 (dd *J* = 19.8, 3.3 Hz).
^{19}F NMR (470 MHz, DMSO-d6) δ -125.2 (dd, *J* = 9.5, 6.1 Hz).
HRMS (HESI): m/z 263.0625 [263.0627 calcd. for $C_{13}H_9F_2N_2O_2^+$ (M+H+)].

(*E*)-4-(phenyldiazenyl)benzoic acid (*p*-**3a**)

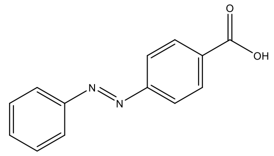

Compound *p*-**3a** was obtained according to general procedure A in 43% yield
TLC: Rf = 0.24 (DCM),
^{1}H NMR (500 MHz, DMSO-d6) δ 13.25 (bs, 1H), 8.15 (d, *J* = 8.7 Hz, 2H), 7.97 (d, *J* = 8.6 Hz, 2H), 7.94 (m, 2H), 7.66 – 7.59 (m, 3H).

^{13}**C NMR** (101 MHz, DMSO) δ 166.8 (s), 154.3 (s), 151.9 (s), 132.9 (s), 132.3 (s), 130.7 (s), 129.6 (s), 122.9 (s), 122.6 (s).
HRMS (HESI): m/z 227.0814 [227.0815 calcd. for $C_{13}H_{11}N_2O_2^+$ (M+H+)].

(*E*)-4-((4-fluorophenyl)diazenyl)benzoic acid (*p*-**3b**)

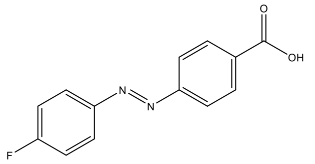

Compound *p*-**3b** was obtained according to general procedure A in 61% yield
TLC: Rf = 0.12 (DCM),
^{1}**H NMR** (400 MHz, DMSO) δ 8.14 (d, *J* = 8.4 Hz, 2H), 8.04 – 7.99 (m, 2H), 7.96 (d, *J* = 8.4 Hz, 2H), 7.50 – 7.43 (m, 2H).
^{13}**C NMR** (101 MHz, DMSO) δ 166.7 (s), 164.3 (d, *J* = 251.1 Hz), 154.1 (s), 148.7 (d, *J* = 2.6 Hz), 132.9 (s), 130.7 (s), 125.3 (d, *J* = 9.3 Hz), 122.6 (s), 116.7 (d, *J* = 23.1 Hz).
^{19}**F NMR** (470 MHz, DMSO-d6) δ -111.28 (tt, *J* = 8.5, 5.3 Hz).
HRMS (HESI): m/z 245.0721 [245.0721 calcd. for $C_{13}H_{10}FN_2O_2^+$ (M+H+)].

(*E*)-4-((2,4-difluorophenyl)diazenyl)benzoic acid (*p*-**3c**)

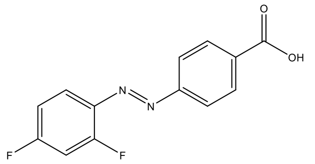

Compound *p*-**3c** was obtained according to general procedure A in 55% yield
TLC: Rf = 0.18 (DCM),
^{1}**H NMR** (400 MHz, DMSO) δ 8.14 (d, *J* = 8.5 Hz, 2H), 7.95 (d, *J* = 8.4 Hz, 2H), 7.85 (td, *J* = 9.0, 6.5 Hz, 1H), 7.65 – 7.57 (m, 1H), 7.31 – 7.23 (m, 1H).
^{13}**C NMR** (101 MHz, DMSO) δ 166.8 (s), 164.8 (dd, *J* = 253.6, 12.1 Hz), 160.3 (dd, *J* = 259.5, 13.2 Hz), 154.4 (s), 137.2 (dd, *J* = 6.7, 3.9 Hz), 133.4 (s), 130.9 (s), 122.9 (s), 119.5 (d, *J* = 10.7 Hz), 112.9 (dd, *J* = 23.0, 3.4 Hz), 105.9 (dd, *J* = 26.7, 23.9 Hz).
^{19}**F NMR** (470 MHz, DMSO-d6) δ -106.8 (tdd, *J* = 17.0, 9.6, 6.5 Hz), -122.5 (dt, *J* = 19.1, 9.6 Hz).
HRMS (HESI): m/z 263.0628 [263.0627 calcd. for $C_{13}H_9F_2N_2O_2^+$ (M+H+)].

(*E*)-4-((2,6-difluorophenyl)diazenyl)benzoic acid (*p*-**3d**)

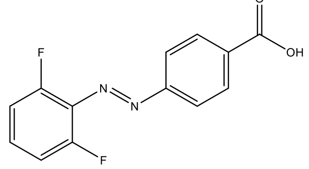

Compound *p*-**3d** was obtained according to general procedure A in 65% yield
TLC: Rf = 0.20(DCM),
^{1}**H NMR** (500 MHz, DMSO-d6) δ 8.16 (d, *J* = 8.6 Hz, 2H), 7.95 (d, *J* = 8.6 Hz, 2H), 7.62 (tt, *J* = 8.4, 6.1 Hz, 1H), 7.43 – 7.33 (m, 2H).

^{13}C NMR (126 MHz, DMSO-d6) δ 166.7 (s), 155.0 (dd, *J* = 258.5, 4.0 Hz), 154.8 (s), 133.8 (s), 132.8 (t, *J* = 10.6 Hz), 130.8 (s), 130.3 (t, *J* = 9.9 Hz), 122.6 (s), 113.2 (dd, *J* = 19.7, 3.6 Hz).
^{19}F NMR (470 MHz, DMSO-d6) δ -124.9 (dd, *J* = 9.5, 6.2 Hz).
HRMS (HESI+): m/z 263.0626 [263.0627 calcd. for $C_{13}H_9F_2N_2O_2$ (M+H$^+$)].

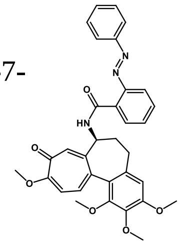

(S,E)-2-(phenyldiazenyl)-N-(1,2,3,10-tetramethoxy-9-oxo-5,6,7,9-tetrahydrobenzo[a]heptalen-7-yl)benzamide (*o*-**AzoCol**)
Compound *o*-**AzoCol** was obtained according to general procedure C in 30% yield

TLC: Rf = 0.64 (DCM/MeOH 9:1),
^{1}H NMR (500 MHz, DMSO-d6) δ 9.18 (d, *J* = 7.3 Hz, 1H), 7.83 – 7.75 (m, 2H), 7.72 – 7.68 (m, 1H), 7.65 – 7.49 (m, 6H), 7.36 (s, 1H), 7.16 (d, *J* = 10.6 Hz, 1H), 7.06 (d, *J* = 11.1 Hz, 1H), 6.82 (s, 1H), 4.61 (dt, *J* = 12.0, 6.9 Hz, 1H), 3.90 (s, 3H), 3.86 (s, 3H), 3.81 (s, 3H), 3.59 (s, 3H), 2.68 – 2.60 (m, 1H), 2.31 – 2.20 (m, 1H), 2.16 – 2.09 (m, 1H), 2.00 – 1.87 (m, 1H)).
^{13}C NMR (126 MHz, DMSO-d6) δ 178.1 (s), 166.6 (s), 163.6 (s), 153.0 (s), 151.9 (s), 150.5 (s), 150.4 (s), 148.8 (s), 140.8 (s), 136.2 (s), 135.1 (s), 134.4 (s), 134.3 (s), 131.7 (s), 131.1 (s), 130.7 (s), 130.4 (s), 129.4 (s), 128.7 (s), 125.5 (s), 122.9 (s), 116.3 (s), 112.0 (s), 107.8 (s), 60.9 (s), 60.7 (s), 56.1 (s), 55.9 (s), 52.2 (s), 35.8 (s), 29.3 (s).
HRMS (HESI): m/z 566.2290 [m/z: 566.2286 calcd. for $C_{33}H_{32}N_3O_6^+$ (M+H+)].

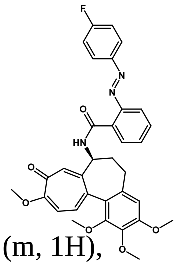

(S,E)-2-((4-fluorophenyl)diazenyl)-N-(1,2,3,10-tetramethoxy-9-oxo-5,6,7,9-tetrahydrobenzo[a]heptalen-7-yl)benzamide *o*-**AzoCol4F**
Compound *o*-**AzoCol4F** was obtained according to general procedure C in 36% yield

TLC: Rf = 0.59 (DCM/MeOH 9:1),
^{1}H NMR (500 MHz, DMSO-d6) δ 9.17 (d, *J* = 7.3 Hz, 1H), 7.90 – 7.81 (m, 2H), 7.71 – 7.67 (m, 1H), 7.65 – 7.58 (m, 3H), 7.37 – 7.32 (m, 1H), 7.17 (d, *J* = 10.6 Hz, 1H), 7.06 (d, *J* = 11.1 Hz, 1H), 6.81 (s, 1H), 4.61 (dt, *J* = 11.9, 6.9 Hz, 1H), 3.91 (s, 3H), 3.86 (s, 3H), 3.81 (s, 3H), 3.60 (s, 3H), 2.63 (dd, *J* = 13.1, 6.0 Hz, 1H), 2.31 – 2.21 (m, 1H), 2.17 – 2.06 (m, 1H), 1.98 – 1.90 (m, 1H).
^{19}F NMR (470 MHz, DMSO-d6) δ -111.99 (tt, *J* = 8.6, 5.3 Hz).
^{13}C NMR 13C NMR (126 MHz, DMSO-d6) δ 178.1 (s), 166.6 (s), 163.9 (d, *J* = 250.4 Hz), 163.6 (s), 153.0 (s), 150.5 (s), 150.5 (s), 148.7 (d, *J* = 2.7 Hz), 148.7 (s), 140.8 (s), 136.4 (s), 135.2 (s), 134.4 (s), 134.3 (s), 131.1 (s), 130.7 (s), 130.4 (s), 128.7 (s), 125.4 (s), 125.3 (d, *J* = 9.3 Hz), 116.3 (d, *J* = 23.0 Hz), 116.1 (s), 112.1 (s), 107.8 (s), 60.9 (s), 60.7 (s), 56.0 (s), 55.9 (s), 52.2 (s), 35.8 (s), 29.3 (s).
HRMS (HESI): m/z 584.2194 [m/z: 584.2191 calcd. for $C_{33}H_{31}FN_3O_6^+$ (M+H+)].

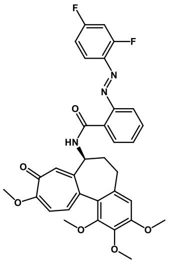

(S,E)-2-((2,4-difluorophenyl)diazenyl)-N-(1,2,3,10-tetramethoxy-9-oxo-5,6,7,9-tetrahydrobenzo[a]heptalen-7-yl)benzamide *o*-**AzoCol24DF**
Compound *o*-**AzoCol24DF** was obtained according to general procedure C in 41% yield

TLC: Rf = 0.60 (DCM/MeOH 9:1),
^{1}H NMR (500 MHz, DMSO-d6) δ 9.16 (d, *J* = 7.3 Hz, 1H), 7.69 – 7.58 (m, 4H), 7.58 – 7.52 (m, 1H), 7.32 (s, 1H), 7.16 (d, *J* = 10.6 Hz, 1H), 7.14 – 7.08 (m, 1H), 7.05 (d, *J* = 11.1 Hz, 1H), 6.81 (s, 1H), 4.61 (dt, *J* = 11.9, 6.9 Hz, 1H), 3.90 (s, 3H), 3.86 (s, 3H), 3.81 (s, 3H), 3.60 (s, 3H), 2.62 (dd, *J* = 13.2, 6.1 Hz, 1H), 2.29 – 2.19 (m, 1H), 2.17 – 2.07 (m, 1H), 1.98 – 1.89 (m, 1H).
^{13}C NMR 178.9 (s), 167.7 (s), 165.0 (dd, *J* = 253.5, 12.1 Hz), 164.2 (s), 160.5 (dd, *J* = 259.3, 13.1 Hz), 153.7 (s), 151.7 (s), 151.0 (s), 149.5 (s), 141.3 (s), 137.6 (dd, *J* = 6.8, 3.8 Hz), 136.7 (s), 136.2 (s),

135.6 (s), 135.0 (s), 132.4 (s), 131.3 (s), 131.0 (s), 129.2 (s), 125.9 (s), 120.2 (d, *J* = 10.6 Hz), 116.9 (s), 113.2 (s), 113.0 (dd, *J* = 22.9, 3.3 Hz), 108.4 (s), 106.0 (dd, *J* = 26.4, 24.0 Hz), 61.6 (s), 61.4 (s), 56.7 (s), 56.5 (s), 53.0 (s), 36.3 (s), 29.7 (s).
^{19}F NMR (470 MHz, DMSO-d6) δ -107.47 – -107.59 (m), -123.04 (dt, *J* = 19.1, 9.3 Hz).
HRMS (HESI): m/z 602.2100 [m/z: 602.2097 calcd. for $C_{33}H_{30}F_2N_3O_6^+$ (M+H+)].

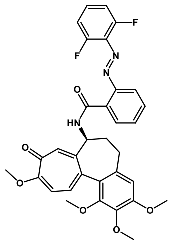

(S,E)-2-((2,6-difluorophenyl)diazenyl)-N-(1,2,3,10-tetramethoxy-9-oxo-5,6,7,9-tetrahydrobenzo[a]heptalen-7-yl)benzamide *o*-**AzoCol26DF**
Compound *o*-**AzoCol26DF** was obtained according to general procedure C in 35% yield

TLC: Rf = 0.61 (DCM/MeOH 9:1),
^{1}H NMR (500 MHz, DMSO-d6) δ 9.10 (d, *J* = 7.4 Hz, 1H), 7.74 – 7.62 (m, 4H), 7.60 (tt, *J* = 8.5, 6.1 Hz, 1H), 7.34 – 7.27 (m, 2H), 7.23 (s, 1H), 7.13 (d, *J* = 10.7 Hz, 1H), 7.01 (d, *J* = 11.1 Hz, 1H), 6.79 (s, 1H), 4.59 (dt, *J* = 11.9, 6.9 Hz, 1H), 3.87 (s, 3H), 3.85 (s, 3H), 3.80 (s, 3H), 3.59 (s, 3H), 2.60 (dd, *J* = 13.2, 6.2 Hz, 1H), 2.25 (td, *J* = 13.2, 7.1 Hz, 1H), 2.12 – 2.00 (m, 1H), 1.94 – 1.85 (m, 1H).
^{13}C NMR (126 MHz, DMSO-d6) δ 177.9 (s), 165.7 (s), 163.5 (s), 155.1 (dd, *J* = 259.1, 4.1 Hz), 153.0 (s), 150.5 (s), 150.1 (s), 149.4 (s), 140.8 (s), 135.5 (s), 135.1 (s), 134.3 (s), 134.2 (s), 132.7 (t, *J* = 10.5 Hz), 132.3 (s), 130.8 (s), 130.6 (s), 130.0 (dd, *J* = 19.6, 9.7 Hz), 129.2 (s), 125.5 (s), 115.7 (s), 113.1 (dd, *J* = 19.8, 3.6 Hz), 112.0 (s), 107.7 (s), 60.9 (s), 60.7 (s), 56.0 (s), 55.9 (s), 52.1 (s), 35.5 (s), 29.2 (s).
^{19}F NMR (470 MHz, DMSO-d6) δ -124.3 (dd, *J* = 9.6, 6.1 Hz).
HRMS (HESI): m/z 602.2100 [602.2097 calcd. for $C_{33}H_{31}FN_3O_6^+$ (M+H+)].

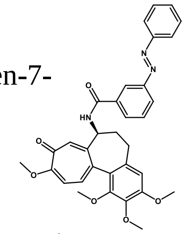

(S,E)-3-(phenyldiazenyl)-N-(1,2,3,10-tetramethoxy-9-oxo-5,6,7,9-tetrahydrobenzo[a]heptalen-7-yl)benzamide *m*-**AzoCol**
Compound *m*-**AzoCol** was obtained according to general procedure B in 53% yield

TLC: Rf = 0.64 (DCM/MeOH 9:1),
^{1}H NMR (500 MHz, DMSO-d6) δ 9.29 (d, *J* = 7.3 Hz, 1H), 8.42 (t, *J* = 1.8 Hz, 1H), 8.09 – 8.04 (m, 2H), 7.96 – 7.91 (m, 2H), 7.72 (t, *J* = 7.8 Hz, 1H), 7.65 – 7.58 (m, 3H), 7.26 (s, 1H), 7.17 (d, *J* = 10.7 Hz, 1H), 7.06 (d, *J* = 11.1 Hz, 1H), 6.81 (s, 1H), 4.63 (dt, *J* = 9.8, 7.8 Hz, 1H), 3.87 (s, 3H), 3.85 (s, 3H), 3.82 (s, 3H), 3.61 (s, 3H), 2.71 – 2.62 (m, 1H), 2.34 – 2.25 (m, 1H), 2.21 – 2.11 (m, 2H).
^{13}C NMR (126 MHz, DMSO-d6) δ 177.9 (s), 165.0 (s), 163.6 (s), 153.0 (s), 151.9 (s), 151.8 (s), 150.7 (s), 150.5 (s), 140.8 (s), 135.2 (s), 135.1 (s), 134.6 (s), 134.3 (s), 131.9 (s), 130.4 (s), 130.3 (s), 129.7 (s), 129.6 (s), 125.5 (s), 125.2 (s), 122.7 (s), 121.5 (s), 112.2 (s), 107.8 (s), 61.0 (s), 60.7 (s), 56.1 (s), 55.9 (s), 52.3 (s), 35.2 (s), 29.3 (s).
HRMS (HESI): m/z 566.2286 [566.2286 calcd. for $C_{33}H_{31}FN_3O_6^+$ (M+H+)].

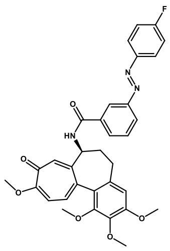

(S,E)-3-((4-fluorophenyl)diazenyl)-N-(1,2,3,10-tetramethoxy-9-oxo-5,6,7,9-tetrahydrobenzo[a]heptalen-7-yl)benzamide *m*-**AzoCol4F**
Compound *m*-**AzoCol4F** was obtained according to general procedure B in 69% yield

TLC: Rf = 0.62 (DCM/MeOH 9:1),
^{1}H NMR (500 MHz, DMSO-d6) δ 9.28 (d, *J* = 7.3 Hz, 1H), 8.40 (t, *J* = 1.8 Hz, 1H), 8.10 – 8.04 (m, 2H), 8.04 – 7.98 (m, 2H), 7.72 (t, *J* = 7.8 Hz, 1H), 7.50 – 7.42 (m, 2H), 7.26 (s, 1H), 7.17 (d, *J* = 10.7 Hz, 1H), 7.06 (d, *J* = 11.2 Hz, 1H), 6.81 (s, 1H), 4.62 (dt, *J* = 9.7, 7.8 Hz, 1H), 3.87 (s, 3H), 3.85 (s, 3H), 3.81 (s, 3H), 3.61 (s, 3H), 2.71 – 2.62 (m, 1H), 2.34 – 2.27 (m, 1H), 2.20 – 2.11 (m, 2H).
^{13}C NMR (126 MHz, DMSO-d6) δ 178.0 (s), 165.0 (s), 164.1 (d, *J* = 250.6 Hz), 163.6 (s), 153.0 (s), 151.7 (s), 150.7 (s), 150.6 (s), 148.7 (d, *J* = 3.0 Hz), 140.8 (s), 135.3 (s), 135.1 (s), 134.6 (s), 134.3 (s),

130.4 (s), 130.3 (s), 129.7 (s), 125.5 (s), 125.2 (s), 125.1 (d, *J* = 9.3 Hz), 121.5 (s), 116.6 (d, *J* = 23.1 Hz), 112.3 (s), 107.9 (s), 61.0 (s), 60.8 (s), 56.1 (s), 55.9 (s), 52.4 (s), 35.3 (s), 29.3 (s).
^{19}F NMR (470 MHz, DMSO-d6) δ -111.8 (tt, *J* = 8.5, 5.3 Hz).
HRMS (HESI): m/z 584.2195 [584.2191 calcd. for $C_{33}H_{31}FN_3O_6^+$ (M+H+)].

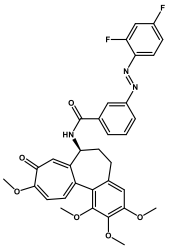

(S,E)-3-((2,4-difluorophenyl)diazenyl)-N-(1,2,3,10-tetramethoxy-9-oxo-5,6,7,9-tetrahydrobenzo[a]heptalen-7-yl)benzamide *m*-**AzoCol24DF**
Compound *m*-**AzoCol24DF** was obtained according to general procedure B in 62% yield

TLC: Rf = 0.57 (DCM/MeOH 9:1),
^{1}H NMR (500 MHz, DMSO-d6) δ 9.29 (d, *J* = 7.3 Hz, 1H), 8.40 (t, *J* = 1.8 Hz, 1H), 8.13 – 8.08 (ddd, *J* = 7.8, 1.6, 1.1 Hz 1H), 8.05 (ddd, *J* = 8.0, 1.9, 1.1 Hz, 1H), 7.85 (td, *J* = 8.9, 6.4 Hz, 1H), 7.73 (t, *J* = 7.8 Hz, 1H), 7.64 – 7.57 (m, 1H), 7.30 – 7.24 (m, 2H), 7.17 (d, *J* = 10.7 Hz, 1H), 7.05 (d, *J* = 11.2 Hz, 1H), 6.81 (s, 1H), 4.66 – 4.59 (m, 1H), 3.87 (s, 3H), 3.85 (s, 3H), 3.82 (s, 3H), 3.61 (s, 3H), 2.71 – 2.62 (m, 1H), 2.35 – 2.25 (m, 1H), 2.20 – 2.12 (m, 2H).
^{13}C NMR (126 MHz, DMSO-d6) δ 178.0 (s), 164.4 (dd, *J* = 253.1, 12.0 Hz), 159.9 (dd, *J* = 259.2, 13.0 Hz), 153.0 (s), 151.9 (s), 150.7 (s), 150.5 (s), 140.8 (s), 136.9 (dd, *J* = 6.9, 4.0 Hz), 135.2 (s), 135.2 (s), 134.6 (s), 134.2 (s), 130.7 (s), 130.4 (s), 125.5 (s), 125.1 (s), 122.0 (s), 119.3 (d, *J* = 11.4 Hz), 112.7 (dd, *J* = 23.1, 3.5 Hz), 112.2 (s), 105.7 (dd, *J* = 26.7, 23.9 Hz), 60.7 (s), 60.7 (s), 56.1 (s), 55.9 (s), 52.3 (s), 35.3 (s), 29.3 (s).
^{19}F NMR (470 MHz, DMSO-d6) δ -107.39 (tdd, *J* = 17.2, 9.0, 6.6 Hz), -122.69 (dt, *J* = 18.8, 9.4 Hz).
HRMS (HESI): m/z 602.2100 [602.2097 calcd. for $C_{33}H_{30}F_2N_3O_6^+$ (M+H+)].

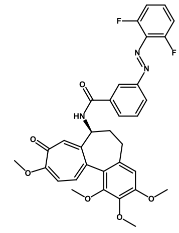

(S,E)-3-((2,6-difluorophenyl)diazenyl)-N-(1,2,3,10-tetramethoxy-9-oxo-5,6,7,9-tetrahydrobenzo[a]heptalen-7-yl)benzamide *m*-**AzoCol26DF**
Compound *m*-**AzoCol26DF** was obtained according to general procedure B in 58% yield

TLC: Rf = 0.63 (DCM/MeOH 9:1),
^{1}H NMR (500 MHz, DMSO-d6) δ 9.32 (d, *J* = 7.3 Hz, 1H), 8.40 (t, *J* = 1.8 Hz, 1H), 8.12 (ddd, *J* = 6.7, 4.5, 3.1 Hz, 1H), 8.03 (ddd, *J* = 8.0, 1.9, 1.1 Hz, 1H), 7.74 (t, *J* = 7.8 Hz, 1H), 7.59 (tt, *J* = 8.5, 6.1 Hz, 1H), 7.39 – 7.32 (m, 2H), 7.25 (s, 1H), 7.17 (d, *J* = 10.7 Hz, 1H), 7.06 (d, *J* = 11.2 Hz, 1H), 6.81 (s, 1H), 4.65 – 4.58 (m, 1H), 3.87 (s, 3H), 3.84 (s, 3H), 3.81 (s, 3H), 3.61 (s, 3H), 2.69 – 2.63 (m, 1H), 2.33 – 2.25 (m, 1H), 2.19 – 2.12 (m, 2H).
^{13}C NMR (126 MHz, DMSO-d6) δ 178.1 (s), 164.9 (s), 163.7 (s), 155.0 (dd, *J* = 257.7, 4.2 Hz), 153.1 (s), 152.5 (s), 150.8 (s), 150.6 (s), 140.9 (s), 135.4 (s), 135.3 (s), 134.7 (s), 134.3 (s), 132.4 (t, *J* = 10.8 Hz), 131.4 (s), 130.4 (s), 130.3 (dd, *J* = 19.1, 8.6 Hz), 123.0 (s), 125.5 (s), 124.9 (s), 121.9 (s), 113.2 (dd, *J* = 19.5, 3.7 Hz), 112.4 (s), 107.9 (s), 61.1 (s), 60.8 (s), 56.2 (s), 56.0 (s), 52.5 (s), 35.3 (s), 29.7 (s), 29.3 (s).
^{19}F NMR (470 MHz, DMSO-d6) δ -125.3 (dd, *J* = 9.4, 6.1 Hz).
HRMS (HESI): m/z 602.2099 [602.2097 calcd. for $C_{33}H_{30}F_2N_3O_6^+$ (M+H+)].

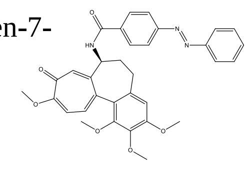

(S,E)-4-(phenyldiazenyl)-N-(1,2,3,10-tetramethoxy-9-oxo-5,6,7,9-tetrahydrobenzo[a]heptalen-7-yl)benzamide *p*-**AzoCol**
Compound *p*-**AzoCol** was obtained according to general procedure B in 45% yield

TLC: Rf = 0.60 (DCM/MeOH 9:1),
^{1}H NMR (500 MHz, DMSO-d6) δ 9.24 (d, *J* = 7.3 Hz, 1H), 8.13 – 8.09 (m, 2H), 7.99 – 7.96 (m, 2H), 7.95 – 7.89 (m, 1H), 7.65 – 7.59 (m, 3H), 7.26 (s, 1H), 7.17 (d, *J* = 10.7 Hz, 1H), 7.06 (d, *J* = 11.1 Hz, 1H), 6.81 (s, 1H), 4.62 (dt, *J* = 10.6, 7.5 Hz, 1H), 3.88 (s, 3H), 3.85 (s, 3H), 3.82 (s, 3H), 3.61 (s, 3H), 2.70 – 2.62 (m, 1H), 2.35 – 2.24 (m, 1H), 2.21 – 2.11 (m, 2H).
^{13}C NMR (126 MHz, DMSO-d6) δ 178.0 (s), 165.0 (s), 163.6 (s), 153.5 (s), 153.0 (s), 151.9 (s), 150.7

(s), 150.5 (s), 140.8 (s), 135.9 (s), 135.2 (s), 134.6 (s), 134.2 (s), 132.0 (s), 130.4 (s), 129.5 (s), 128.8 (s), 125.5 (s), 122.8 (s), 122.4 (s), 112.2 (s), 107.8 (s), 60.9 (s), 60.7 (s), 56.1 (s), 55.9 (s), 52.3 (s), 45.6 (s), 35.3 (s), 29.3 (s), 8.5 (s).
HRMS (HESI): m/z 566.2290 [566.2286 calcd. for $C_{33}H_{32}N_3O_6^+$ (M+H+)].

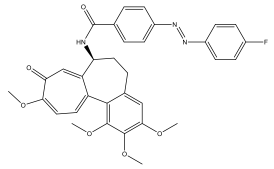

(S,E)-4-((4-fluorophenyl)diazenyl)-N-(1,2,3,10-tetramethoxy-9-oxo-5,6,7,9-tetrahydrobenzo[a]heptalen-7-yl)benzamide *p*-**AzoCol4F**
Compound *p*-**AzoCol4F** was obtained according to general procedure B in 52% yield

TLC: Rf = 0.60 (DCM/MeOH 9:1),
^{1}H NMR (500 MHz, DMSO-d6) δ 9.23 (d, *J* = 7.3 Hz, 1H), 8.12 – 8.08 (m, 2H), 8.03 – 7.98 (m, 2H), 7.98 – 7.94 (m, 2H), 7.48 – 7.42 (m, 2H), 7.26 (s, 1H), 7.17 (d, *J* = 10.7 Hz, 1H), 7.05 (d, *J* = 11.1 Hz, 1H), 6.81 (s, 1H), 4.65 – 4.58 (m, 1H), 3.88 (s, 3H), 3.85 (s, 3H), 3.82 (s, 3H), 3.61 (s, 3H), 2.69 – 2.64 (m, 1H), 2.34 – 2.26 (m, 1H), 2.19 – 2.11 (m, 2H).
^{13}C NMR (126 MHz, DMSO-d6) δ 177.9 (s), 165.0 (s), 164.1 (d, *J* = 250.8 Hz), 163.6 (s), 153.3 (s), 153.0 (s), 150.7 (s), 150.5 (s), 148.7 (d, *J* = 2.8 Hz), 140.8 (s), 136.0 (s), 135.2 (s), 134.6 (s), 134.2 (s), 130.4 (s), 128.8 (s), 125.5 (s), 125.2 (d, *J* = 9.3 Hz), 122.4 (s), 116.6 (d, *J* = 23.0 Hz), 112.2 (s), 107.8 (s), 60.9 (s), 60.7 (s), 56.1 (s), 55.9 (s), 52.3 (s), 35.3 (s), 29.3 (s).
^{19}F NMR (470 MHz, DMSO-d6) δ -111.5 (tt, *J* = 8.5, 5.3 Hz).
HRMS (HESI): m/z 584.2191 [584.2191calcd. for $C_{33}H_{31}FN_3O_6^+$ (M+H+)].

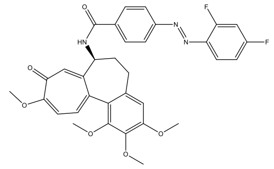

(S,E)-4-((2,4-difluorophenyl)diazenyl)-N-(1,2,3,10-tetramethoxy-9-oxo-5,6,7,9-tetrahydrobenzo[a]heptalen-7-yl)benzamide *p*-**AzoCol24DF**
Compound *p*-**AzoCol24DF** was obtained according to general procedure B in 61% yield

TLC: Rf = 0.59 (DCM/MeOH 9:1),
^{1}H NMR (500 MHz, DMSO-d6) δ 9.24 (d, *J* = 7.3 Hz, 1H), 8.11 (d, *J* = 8.6 Hz, 2H), 7.97 (d, *J* = 8.6 Hz, 2H), 7.86 (td, *J* = 8.9, 6.4 Hz, 1H), 7.64 – 7.57 (m, 1H), 7.30 – 7.23 (m, 2H), 7.17 (d, *J* = 10.7 Hz, 1H), 7.06 (d, *J* = 11.0 Hz, 1H), 6.81 (s, 1H), 4.64 – 4.58 (m, 1H), 3.87 (s, 3H), 3.85 (s, 3H), 3.81 (s, 3H), 3.61 (s, 3H), 2.72 – 2.61 (m, 1H), 2.35 – 2.24 (m, 1H), 2.20 – 2.10 (m, 2H).
^{13}C NMR (126 MHz, DMSO-d6) δ 178.0 (s), 165.0 (s), 164.5 (dd, *J* = 253.9, 11.9 Hz), 163.6 (s), 160.1 (dd, *J* = 259.2, 13.3 Hz), 153.5 (s), 153.0 (s), 150.7 (s), 150.5 (s), 140.8 (s), 137.0 (dd, *J* = 6.9, 4.0 Hz), 136.4 (s), 135.3 (s), 134.6 (s), 134.3 (s), 130.4 (s), 128.9 (s), 125.5 (s), 122.7 (s), 119.3 (d, *J* = 10.6 Hz), 112.7 (dd, *J* = 23.2, 3.5 Hz), 112.3 (s), 107.8 (s), 105.8 (dd, *J* = 26.7, 23.9 Hz), 61.0 (s), 60.8 (s), 56.1 (s), 55.9 (s), 52.4 (s), 35.3 (s), 29.3 (s).
^{19}F NMR (470 MHz, DMSO-d6) δ -107.0 (tdd, *J* = 17.3, 9.0, 6.6 Hz), -122.6 (dt, *J* = 19.6, 9.9 Hz).
HRMS (HESI): m/z 602.2100 [602.2097 calcd. for $C_{33}H_{30}F_2N_3O_6^+$ (M+H+)].

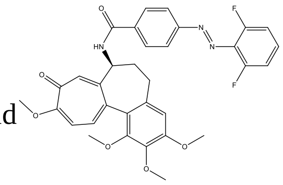

(S,E)-4-((2,6-difluorophenyl)diazenyl)-N-(1,2,3,10-tetramethoxy-9-oxo-5,6,7,9-tetrahydrobenzo[a]heptalen-7-yl)benzamide *p*-**AzoCol26DF**
Compound *p*-**AzoCol26DF** was obtained according to general procedure B in 60% yield

TLC: Rf = 0.65 (DCM/MeOH 9:1),
^{1}H NMR (500 MHz, DMSO-d6) δ 9.28 (d, *J* = 7.2 Hz, 1H), 8.10 (d, *J* = 8.6 Hz, 2H), 7.95 (d, *J* = 8.6 Hz, 2H), 7.59 (tt, *J* = 8.5, 6.1 Hz, 1H), 7.38 – 7.31 (m, 2H), 7.25 (s, 1H), 7.17 (d, *J* = 10.7 Hz, 1H), 7.06 (d, *J* = 11.1 Hz, 1H), 6.80 (s, 1H), 4.63 – 4.56 (m, 1H), 3.87 (s, 3H), 3.84 (s, 3H), 3.80 (s, 3H), 3.60 (s, 3H), 2.69 – 2.63 (m, 1H), 2.32 – 2.24 (m, 1H), 2.18 – 2.11 (m, 2H).
^{13}C NMR (126 MHz, DMSO-d6) δ 178.2 (s), 165.2 (s), 163.8 (s), 155.1 (dd, *J* = 258.0, 4.3 Hz), 154.1 (s), 153.2 (s), 151.0 (s), 150.7 (s), 140.9 (s), 136.9 (s), 135.5 (s), 134.9 (s), 134.4 (s), 132.7 (t, *J* = 11.0 Hz), 130.5 (s), 130.4 (t, *J* = 10.0 Hz), 129.0 (s), 125.6 (s), 122.6 (s), 113.2 (dd, *J* = 19.7, 3.7 Hz),

112.5 (s), 108.0 (s), 61.1 (s), 60.9 (s), 56.2 (s), 56.0 (s), 52.6 (s), 35.4 (s), 29.4 (s).
^{19}F NMR (470 MHz, DMSO-d6) δ -125.3 (dd, *J* = 9.4, 6.1 Hz).
HRMS (HESI): m/z 602.2099 [602.2097 calcd. for $C_{33}H_{30}F_2N_3O_6^+$ (M+H+)].

(S)-2-amino-N-(1,2,3,10-tetramethoxy-9-oxo-5,6,7,9-tetrahydrobenzo[a]heptalen-7-yl)benzamide – Intermidiate **S1**.

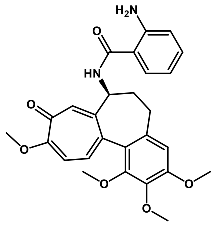

^{1}H NMR (500 MHz, $CDCl_3$) δ 7.57 – 7.50 (m, 2H), 7.30 (d, *J* = 10.7 Hz, 1H), 7.16 (d, *J* = 6.0 Hz, 1H), 7.13 – 7.07 (m, 1H), 6.82 (d, *J* = 11.1 Hz, 1H), 6.59 (dd, *J* = 13.7, 7.3 Hz, 2H), 6.53 (s, 1H), 4.77 (dt, *J* = 12.8, 6.6 Hz, 1H), 3.96 (s, 3H), 3.94 (s, 3H), 3.89 (s, 3H), 3.70 (s, 3H), 2.53 (dd, *J* = 13.4, 6.3 Hz, 1H), 2.42 (td, *J* = 13.1, 6.8 Hz, 1H), 2.32 – 2.22 (m, 1H), 1.98 (dd, *J* = 11.7, 5.3 Hz, 1H).

NMR & UV-VIS Data – Photostationary State (PSS) Analysis, Photoswitching and Half-Life Determination

Illumination setup for light-dependent assays:
Based on previously described data [4-8] for illumination of samples we decided to used self-built arrays of 24 low-power light-emitting diodes (LED) controlled by Adurino board. Such an automated system allowed precise pulsed illumination during long-term assays. Most importantly, it has been proven to be compatible with cell culturing conditions. The proposed system consisted of the Arduino microcomputer to drive an 8-Relay module, which turns on and off sets of LED-arrays to flash with identical timings. The following wavelengths of LEDs were used in this study (in nm): 390, 400, 430, 470, 505, 515, 525, 535, 590, 610 nm. all LEDs were bought from Mouser Electronics, Inc., and the corresponding Mouser part numbers are 749-UV5TZ-390-30, 749-UV5TZ-400-15, 749-5BWC, 630-HLMP-CB3A-UV0DD, 630-HLMP-CE34-Y1CDD, 859-LTL2V3TCYK2, 630-HLMP-CM3A-Z10DD, 859-LTL2V3TGX3KS, 630-HLMP-EL1A-Z1KDD, respectively. Only one wavelength of LED was used per array, and arrays at different wavelengths were kept in separate cardboard boxes. We consider this crucial since with standard, clear well plates, light scattering from one well across the whole plate was often observed to be significant enough to compromise results.

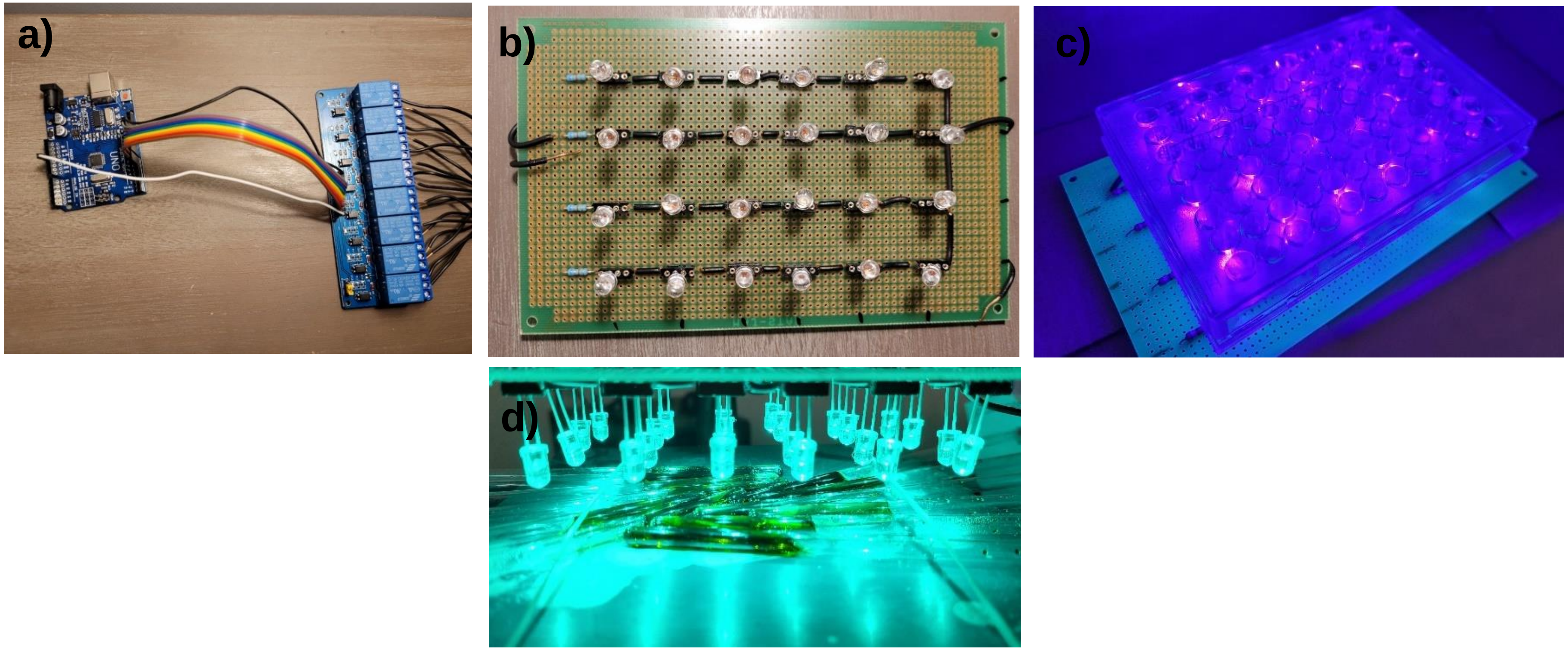

Figure S1. Illumination setup for light-dependent assays used in this study: (**a**) Adurino board connected with 8-Relay module, (**b**) Front of 24 low-power light-emitting diode array, (**c**) Array illuminating 96-well plate, (**d**) Array illuminating NMR samples.

Photostationary State (PSS) Analysis by ^{1}H or ^{19}F NMR spectroscopy:
The PSS composition was determined by measuring 1H or NMR 19F spectra (DMSO-d_6, c ≈ 2 mM) of the respective compounds after irradiation. For thermally adapted experiments samples were kept in the dark at 120 °C overnight to ensure complete relaxation to (*E*) isomer. Next, the probe was illuminated with LEDs of different wavelengths while gradually increasing the irradiation time until no change in the spectrum was detectable.

Photoisomerization studies by UV-VIS spectroscopy:
EnSpire® multimode plate reader (PerkinElmer, Turku, Finland) with the software EnSpire Workstation version 4.10.3005.1440 (PerkinElmer) in absorbance mode was used for UV-VIS spectroscopic measurements. All experiments were done in at least triplicate. Stock solutions of selected compounds were made in DMSO at appropriate concentrations and stirred at 120 °C overnight to ensure complete relaxation to (*E*) isomer. Next 100 µL of stock solution was transferred to 96 – a well plate as quickly as possible and absorbance was read. Afterward, samples were illuminated with green light (λ = 535 nm) by a self-made LED setup. Samples were illuminated with increasing periods of time until no further changes in the spectrum were observed.

Photoswitching exerimetns:
EnSpire® multimode plate reader (PerkinElmer, Turku, Finland) with the software EnSpire Workstation version 4.10.3005.1440 (PerkinElmer) in absorbance mode was used for UV-VIS photoswitching measurements. Samples were prepared in the 24 well plate and illuminated first with green (535 nm) light for 15 minutes. Next well plate was transferred as quickly as possible and absorbance was read. Afterwards, samples were illuminated with blue (430 nm) light for 15 minutes and the well plate was transferred as quickly as possible and absorbance was read. Then the protocol was repeated several times.

Half-Life Determination:
Samples were prepared in the well plate and illuminated with green light (λ = 535 nm) by array of LEDs while gradually increasing the irradiation time until no change in the spectrum was detectable. Then the well plate was placed in EnSpire® multimode plate reader and the absorbance was folowed for 48 hours at 37 °C. The (*Z*) → (*E*) spontaneous relaxation half-life time was caluclated using GraphPad Prism software (GraphPad Software Inc, San Diego, CA, USA).

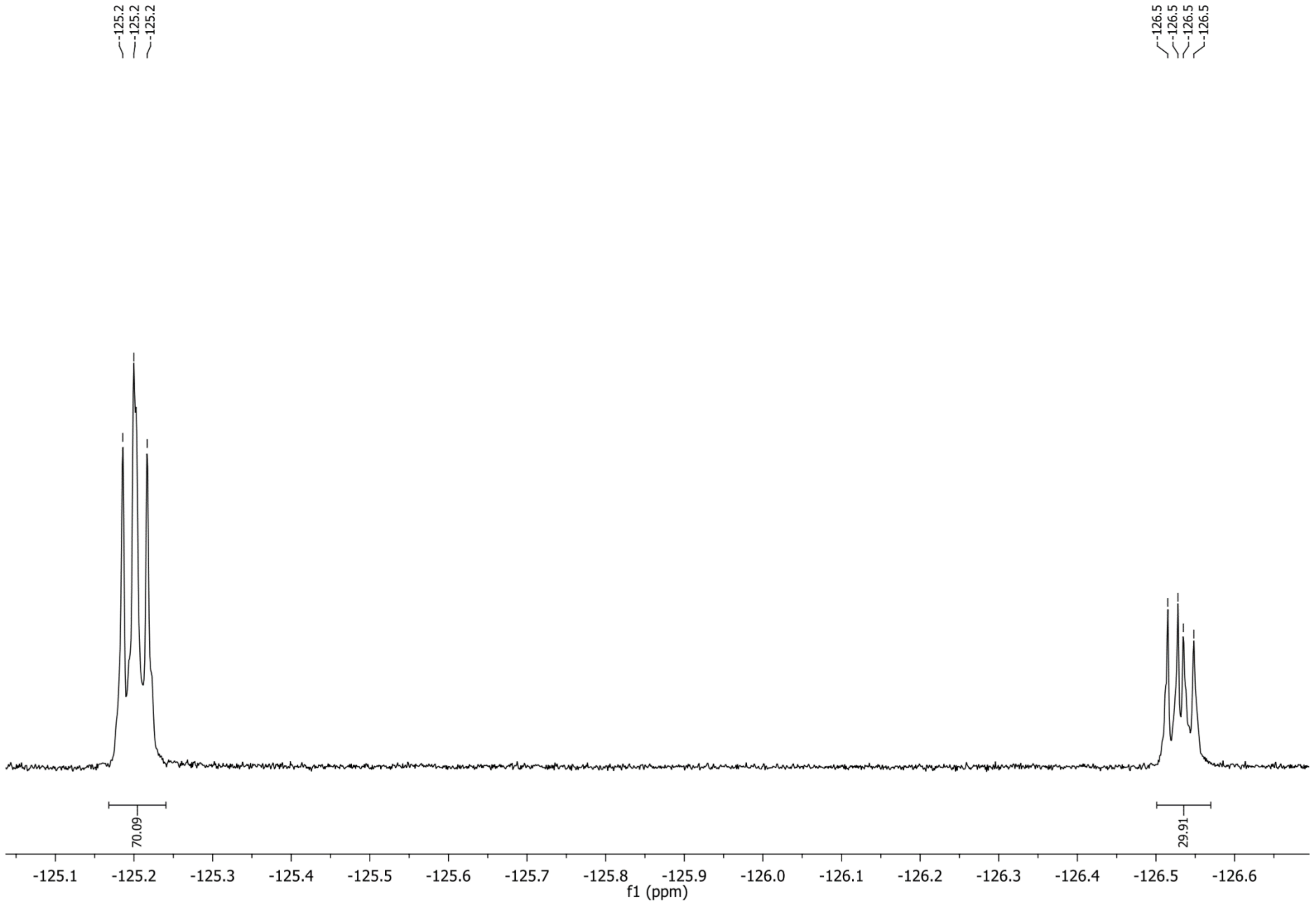

Figure S2. Distribution of (*E*) and (*Z*) isomers for compound *m*-**3d** in DMSO-d_6 (c ≈ 2 mM) after continuous irradiation with green light (λ = 535 nm) for 2h. The sample contained 70 % of (*Z*) isomer.

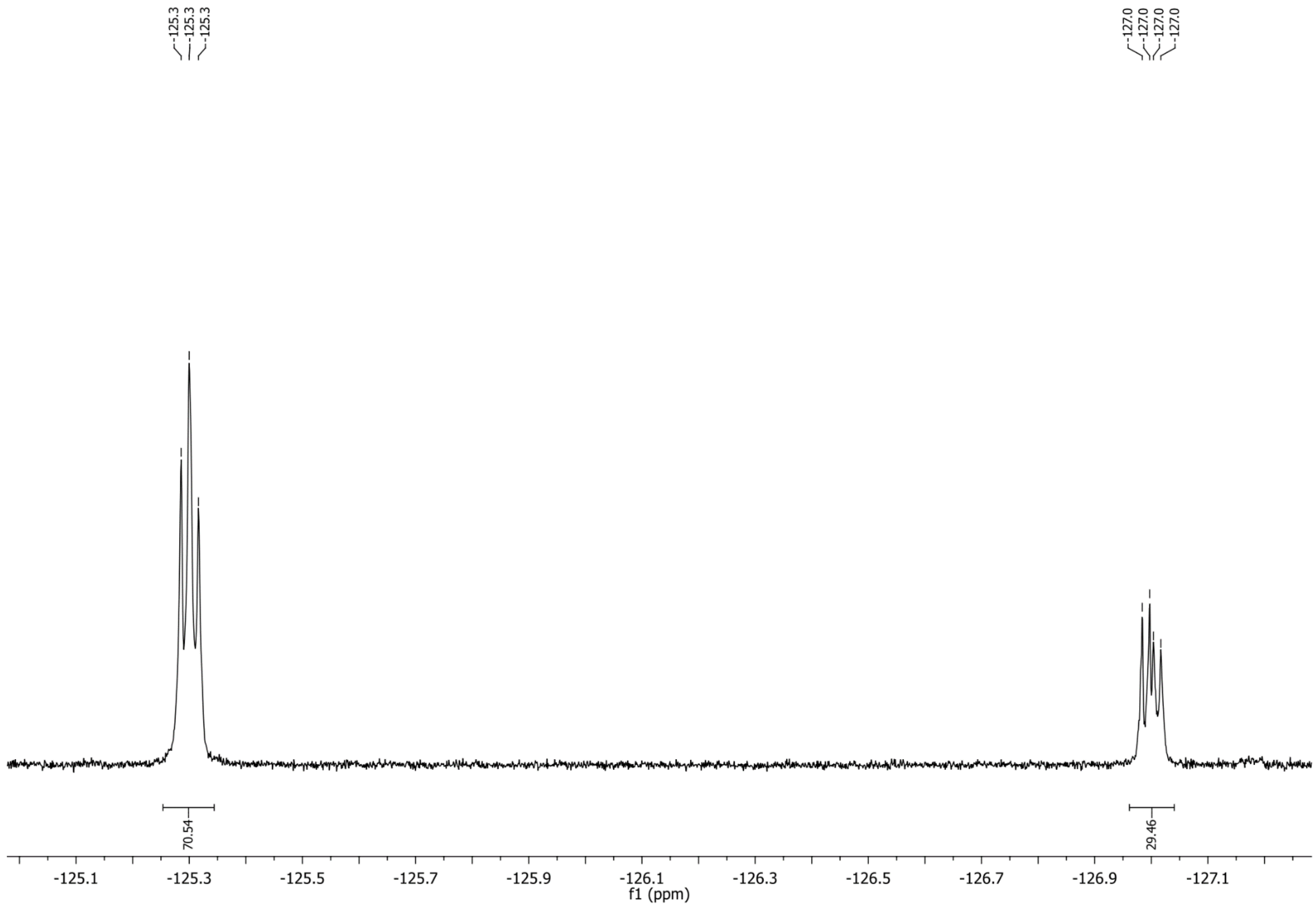

Figure S3. Distribution of (*E*) and (*Z*) isomers for compound *m*-**AzoCol26DF** in DMSO-d_6 (c ≈ 2 mM) after continuous irradiation with green light (λ = 535 nm) for 2h. The sample contained 71 % of (*Z*) isomer.

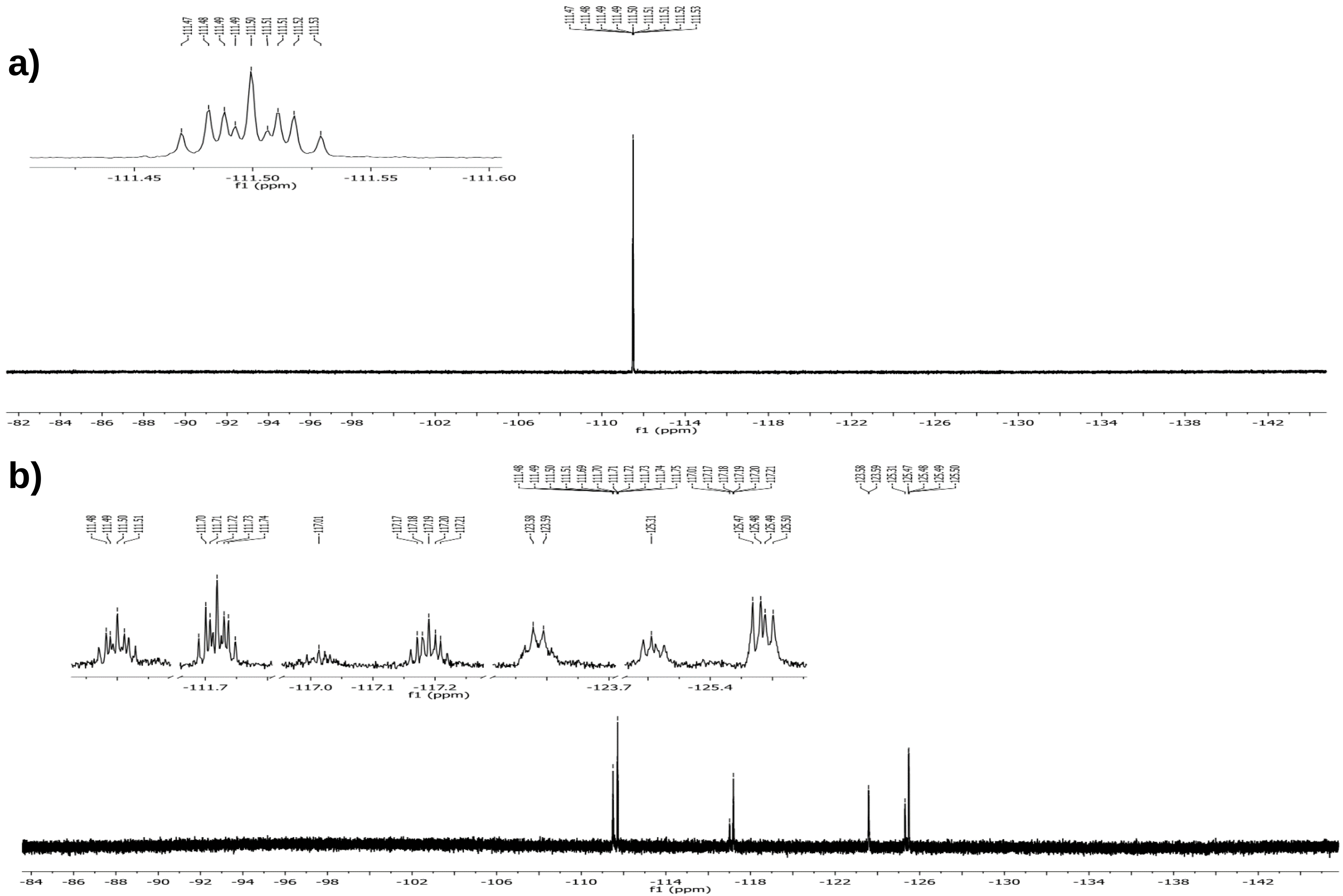

Figure S4. ^{19}F NMR spectra of *p*-**AzoCol4F** in DMSO-d_6 (c ≈ 2 mM) (**a**) before and (**b**) after irradiation with UV light (365 nm) showing formation of new compounds (presumably derivatives of β-lumicolchicine, γ-lumicolchicine and α-lumicolchicine).

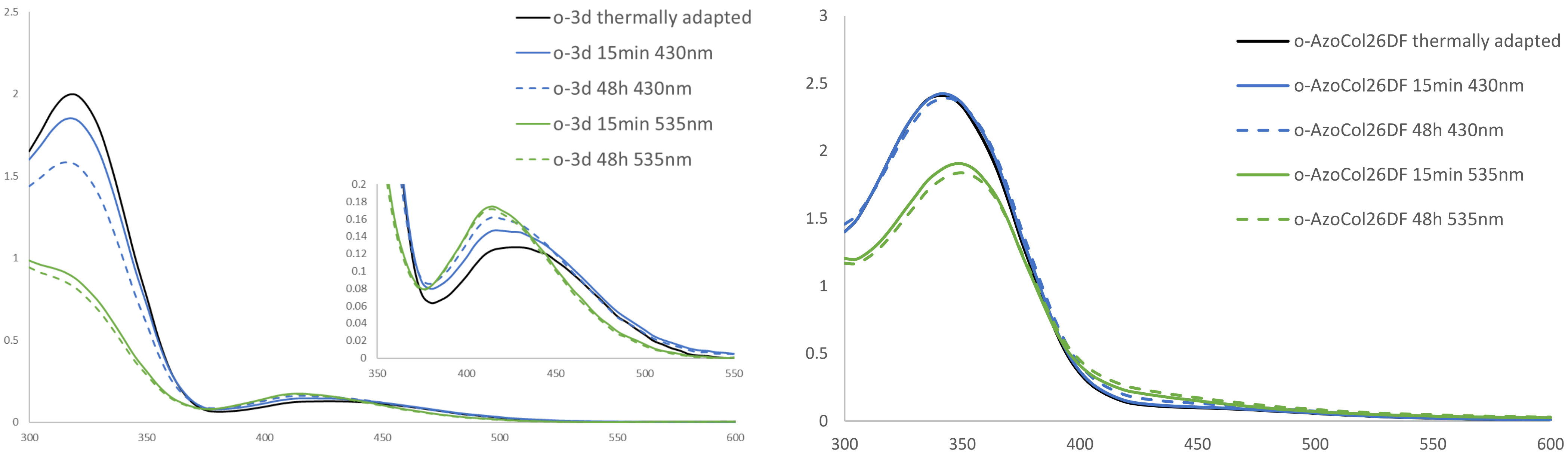

Figure S5. UV-Vis absorption spectra of thermally adapted and pulsed green (535 nm) and blue (430 nm) light irradiated 500 µM of (**a**) *o*-**3d** and (**b**) *o*-**AzoCol26DF** in Dulbecco's Modified Eagle's Medium (Thermo Fisher Scientific, 11054020) supplemented with 4.5 g/L glucose and 4 mM L-glutamine with 10% of DMSO.

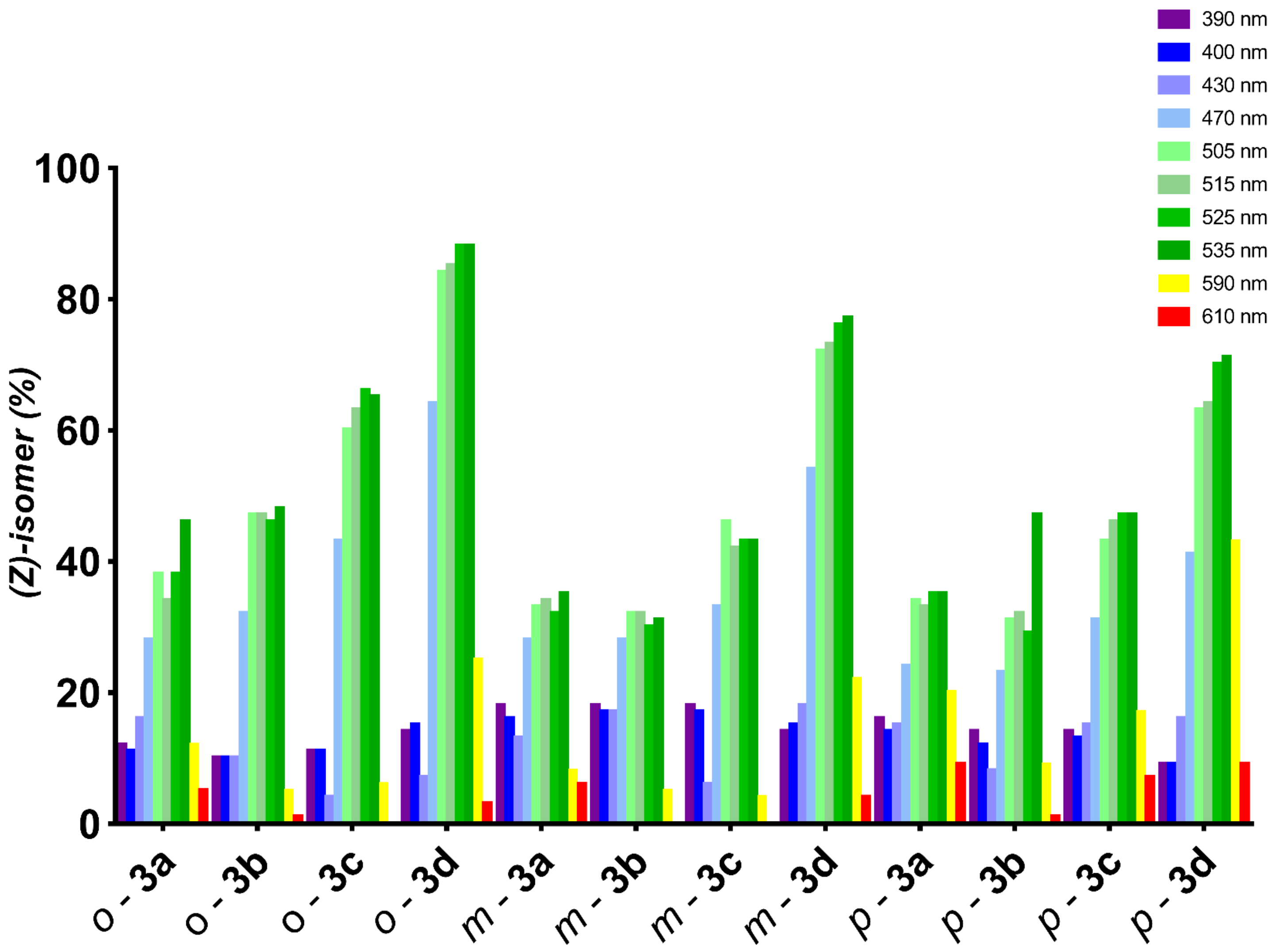

Figure S6. Photostationary state (PSS) compositions for azobenzenes *o*-, *m*-, *p*-**3a-d** determined by ^{1}H or ^{19}F NMR analysis (c ≈ 10 mM in DMSO-d_6).

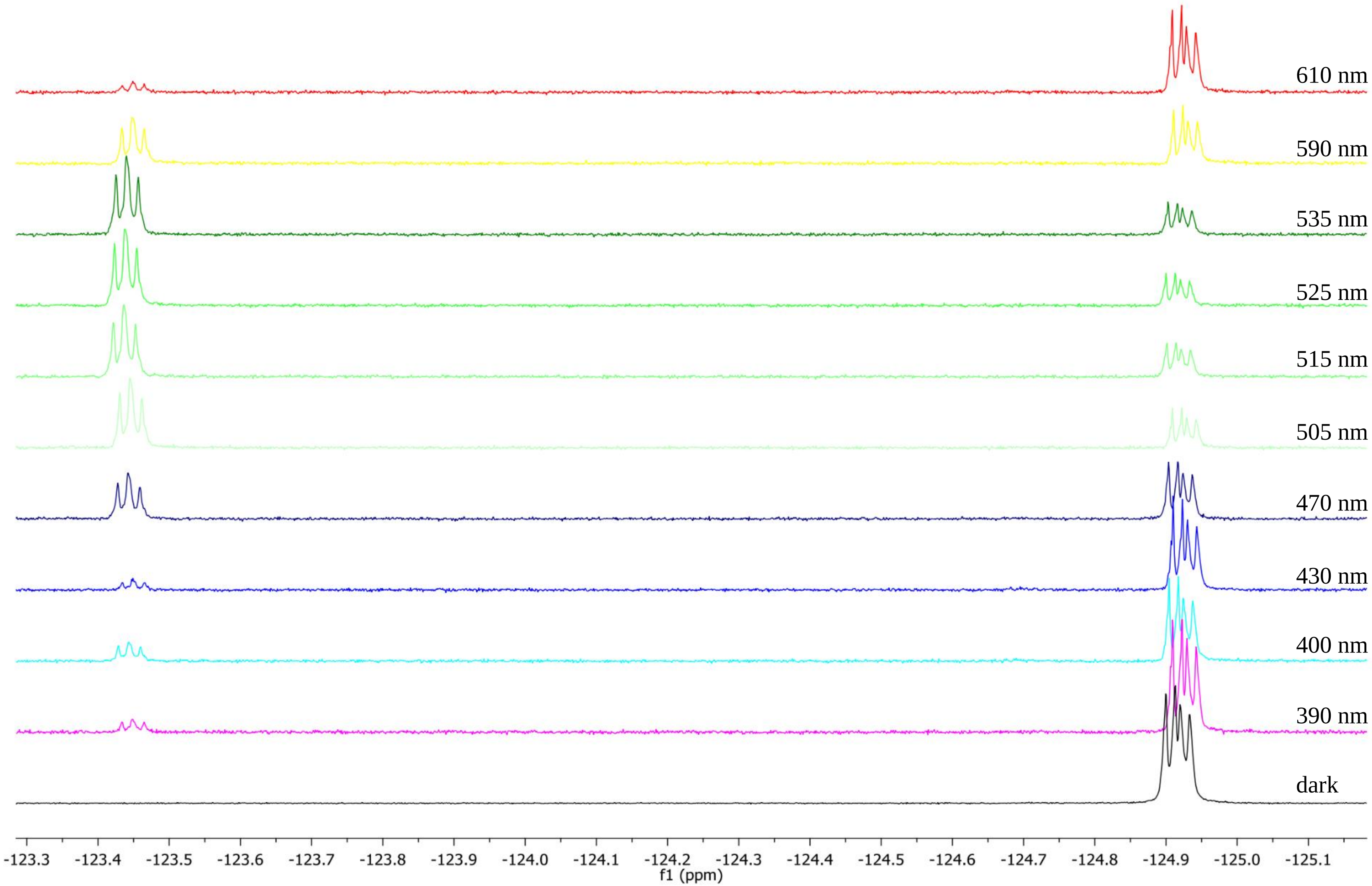

Figure S7. Partial ^{19}F NMR spectra of ***p*-3d** at dark adapted state and after irradiation with light at different wavelength in DMSO-d_6 (c ≈ 2 mM).

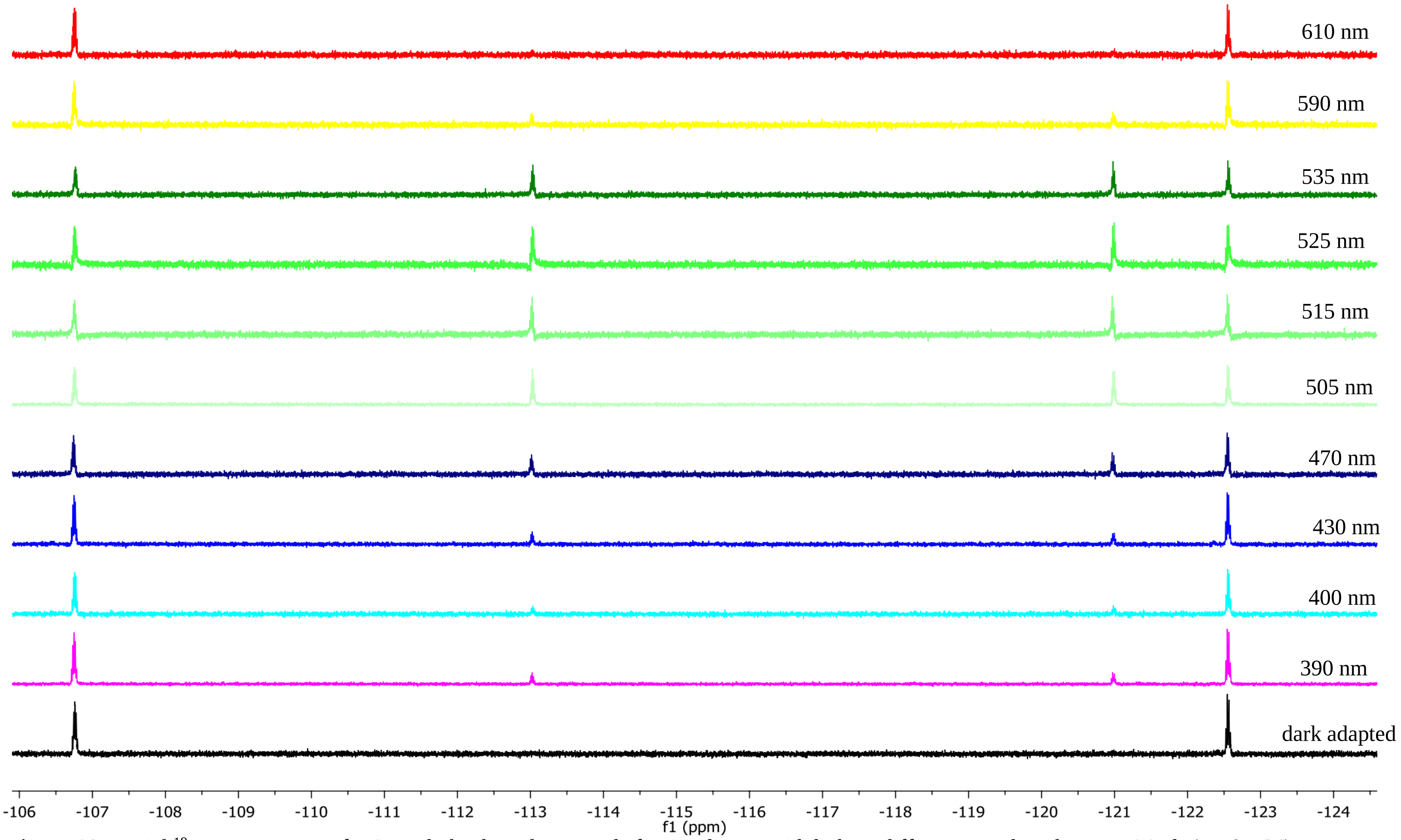

Figure S8. Partial ^{19}F NMR spectra of *p*-**3c** at dark adapted state and after irradiation with light at different wavelength in DMSO-d_6 (c ≈ 2 mM).

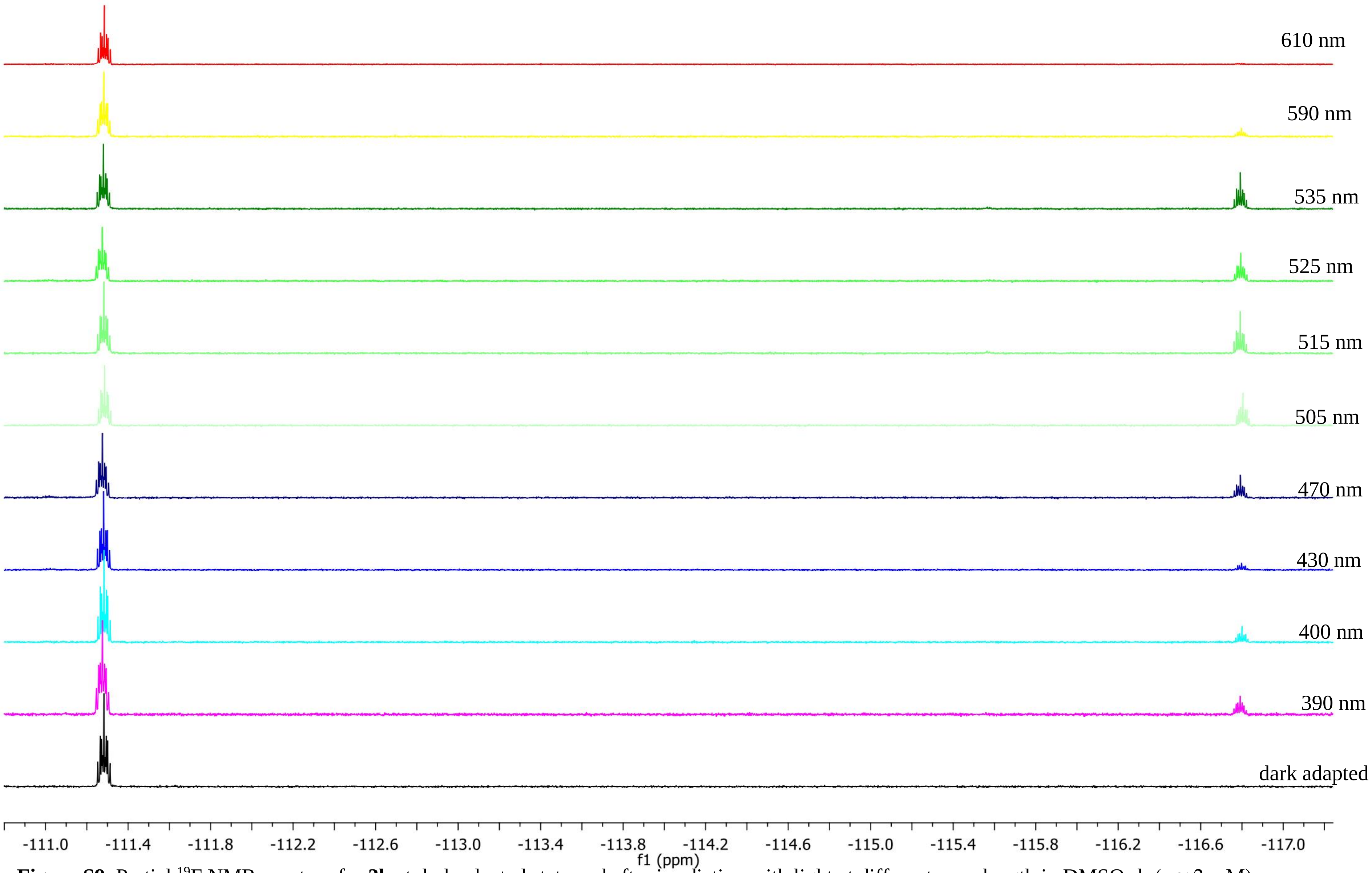

Figure S9. Partial ^{19}F NMR spectra of *p*-**3b** at dark adapted state and after irradiation with light at different wavelength in DMSO-d_6 (c ≈ 2 mM).

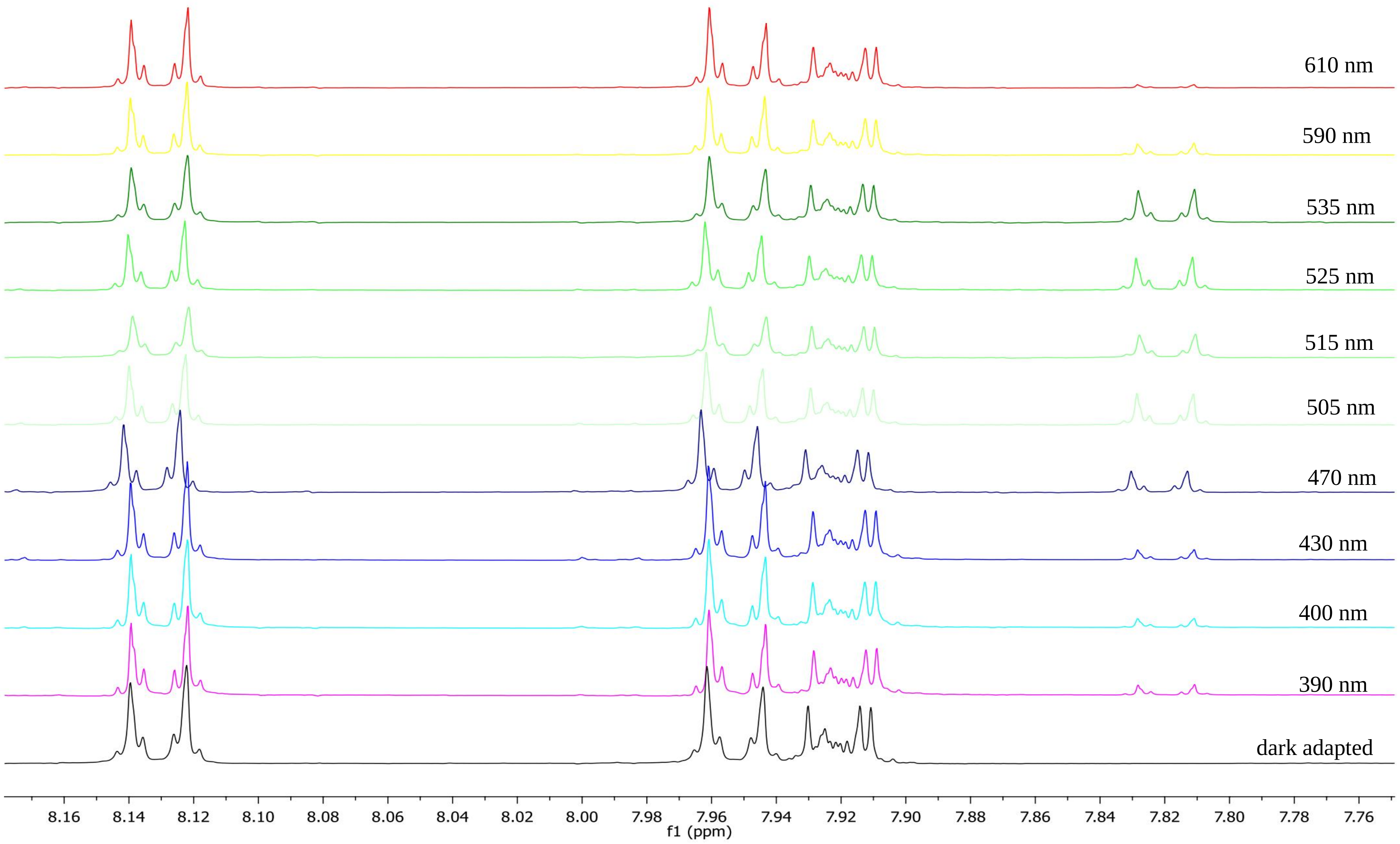

Figure S10. Partial ^{1}H NMR spectra of ***p*-3a** at dark adapted state and after irradiation with light at different wavelength in DMSO-d_6 ($c \approx 2$ mM).

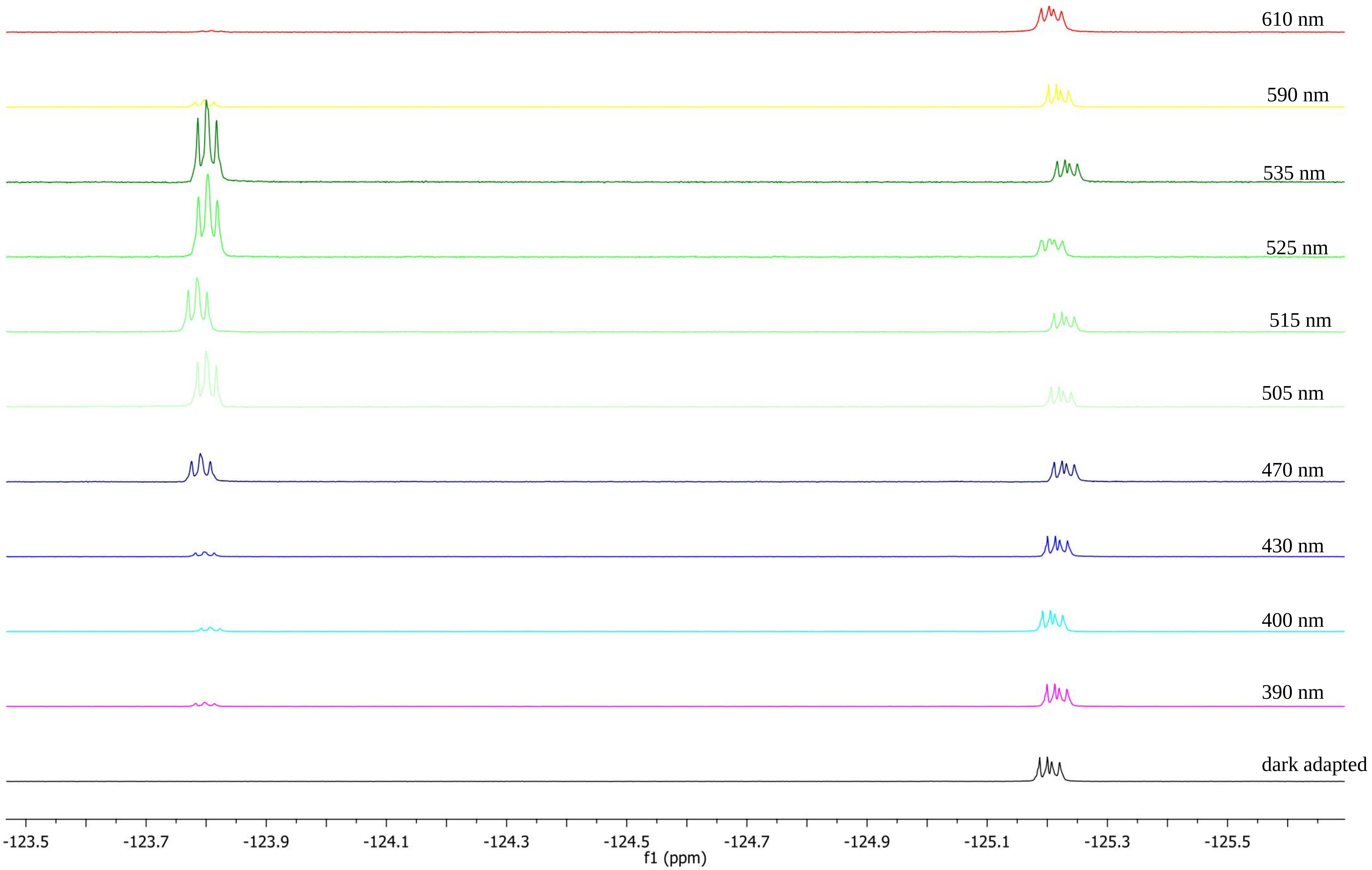

Figure S11. Partial ^{19}F NMR spectra of *m*-**3d** at dark adapted state and after irradiation with light at different wavelength in DMSO-d_6 (c ≈ 2 mM).

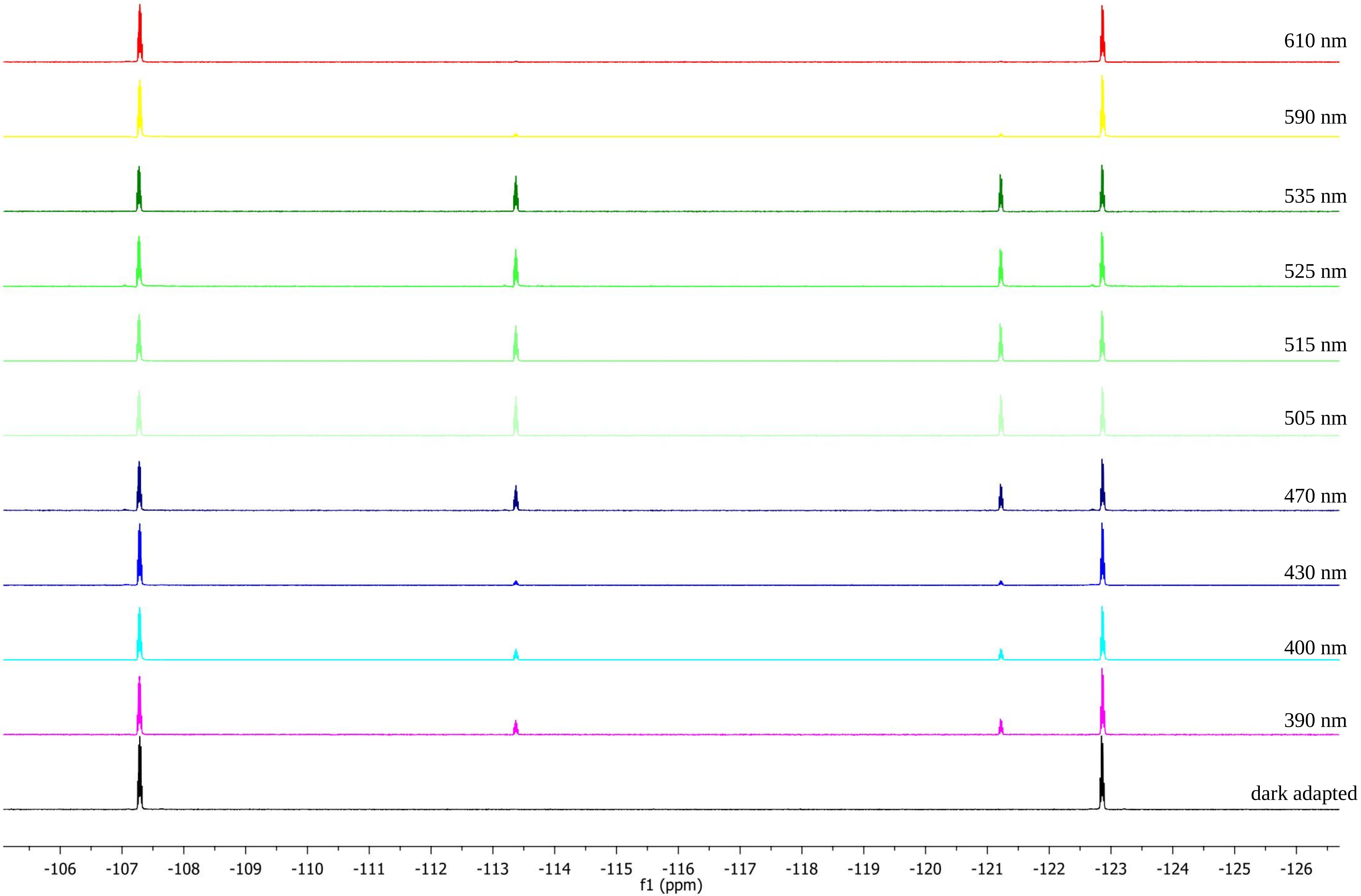

Figure S12. Partial ^{19}F NMR spectra of *m*-**3c** at dark adapted state and after irradiation with light at different wavelength in DMSO-d_6 (c ≈ 2 mM).

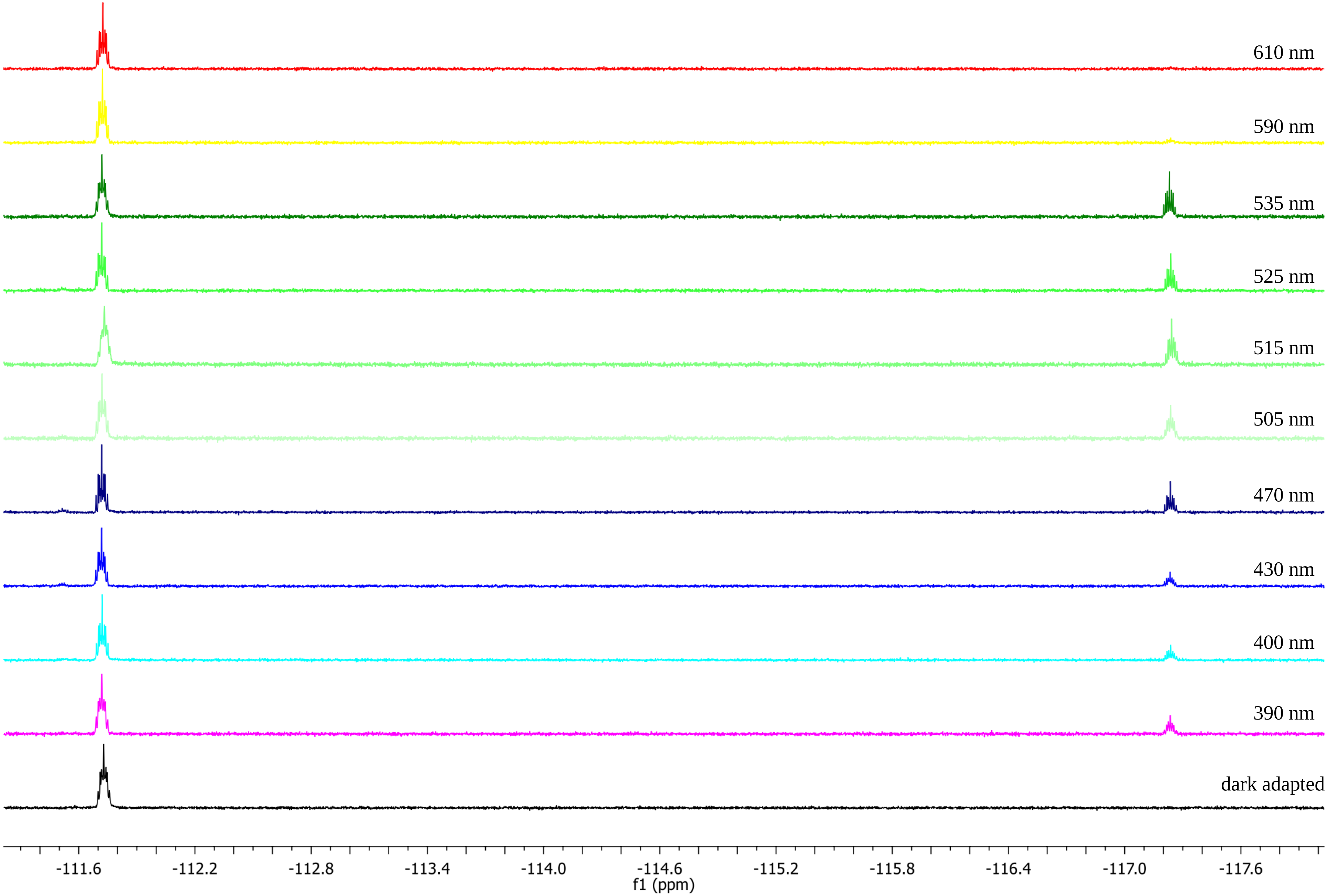

Figure S13. Partial ^{19}F NMR spectra of *m*-**3b** at dark adapted state and after irradiation with light at different wavelength in DMSO-d_6 (c ≈ 2 mM).

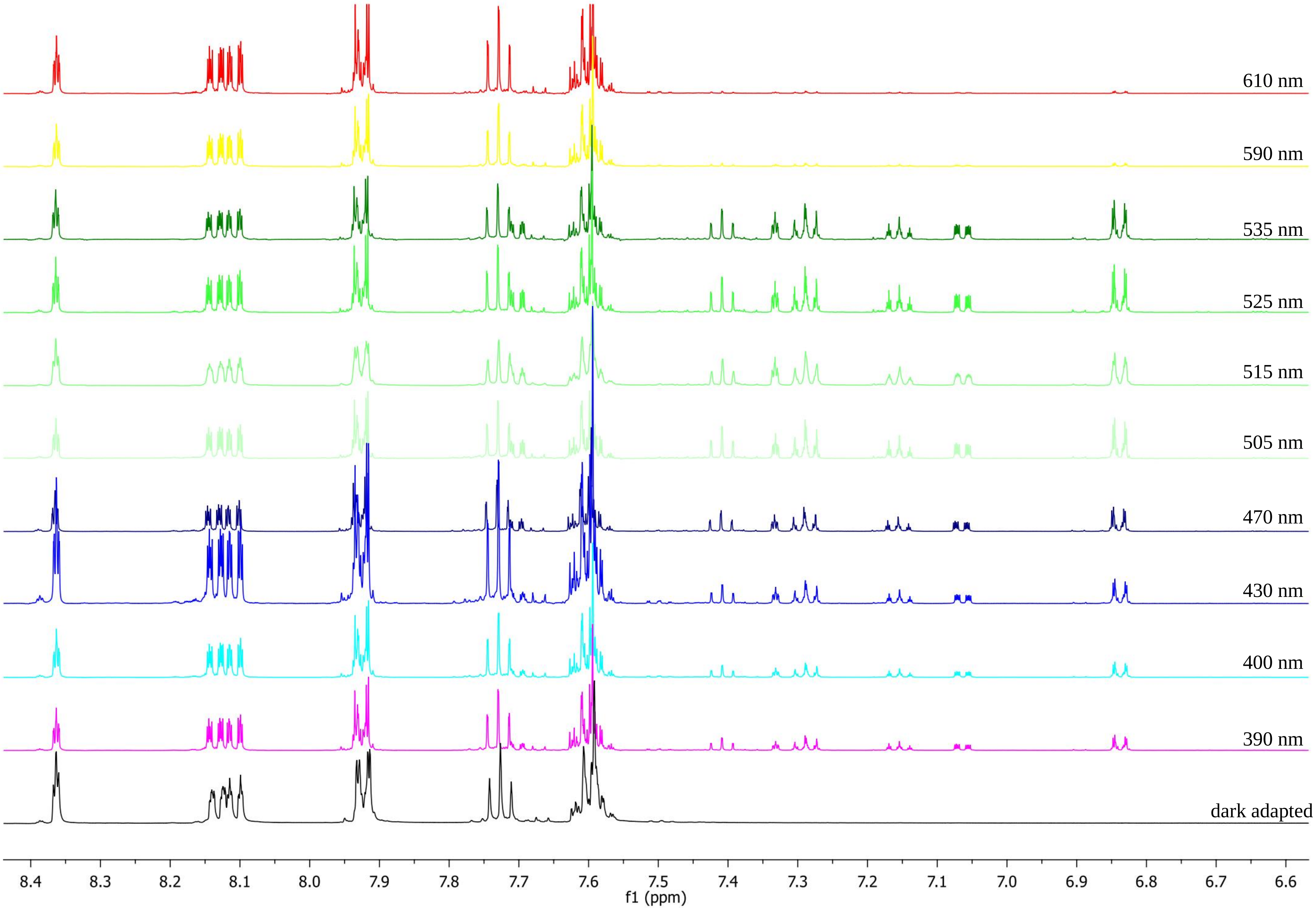

Figure S14. Partial ^{1}H NMR spectra of *m*-**3a** at dark adapted state and after irradiation with light at different wavelength in DMSO-d_6 (c ≈ 2 mM).

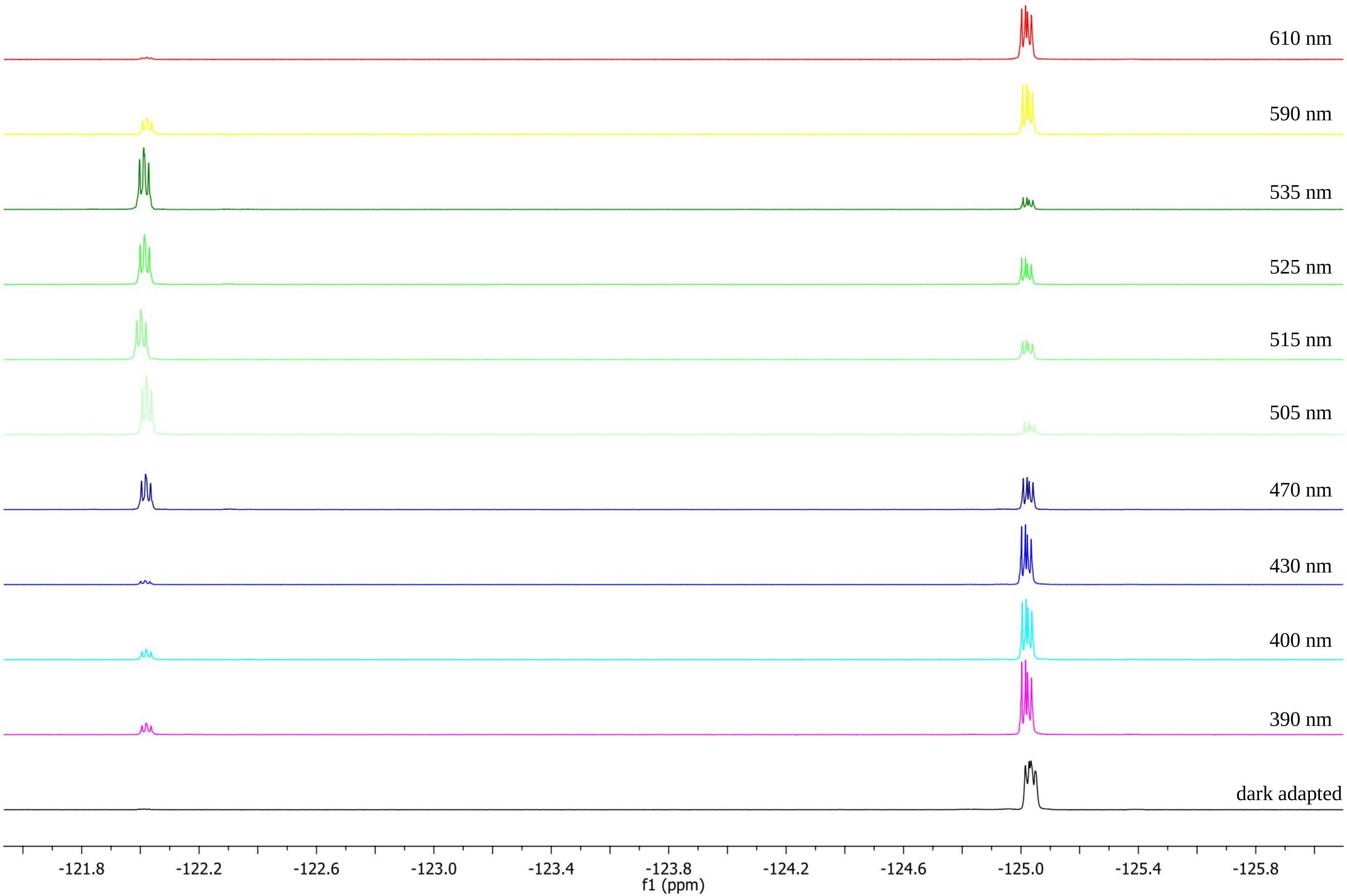

Figure S15. Partial ^{19}F NMR spectra of *o*-**3d** at dark adapted state and after irradiation with light at different wavelength in DMSO-d_6 ($c \approx 2$ mM).

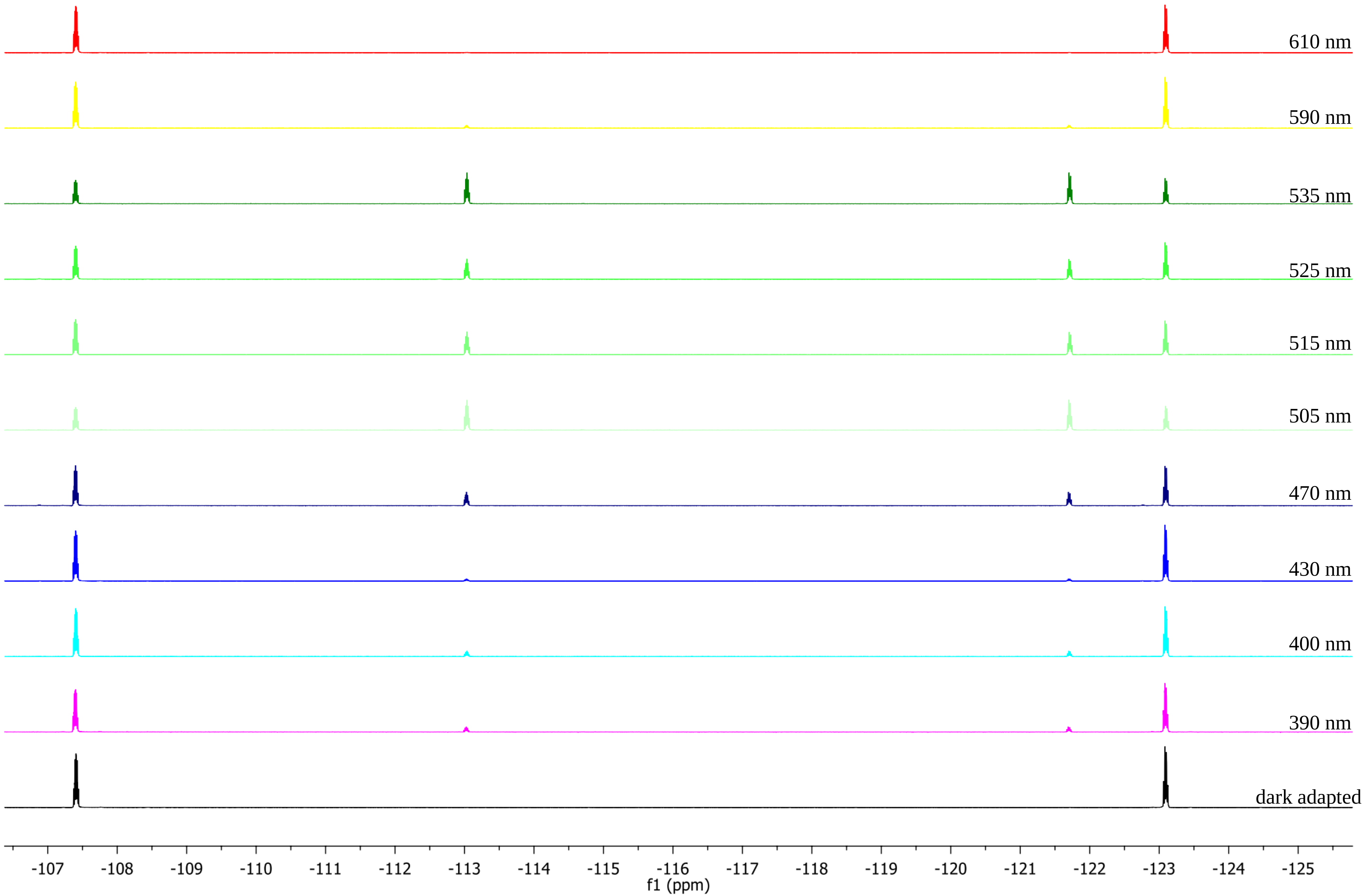

Figure S16. Partial ^{19}F NMR spectra of *o*-**3c** at dark adapted state and after irradiation with light at different wavelength in DMSO-d_6 ($c \approx 2$ mM).

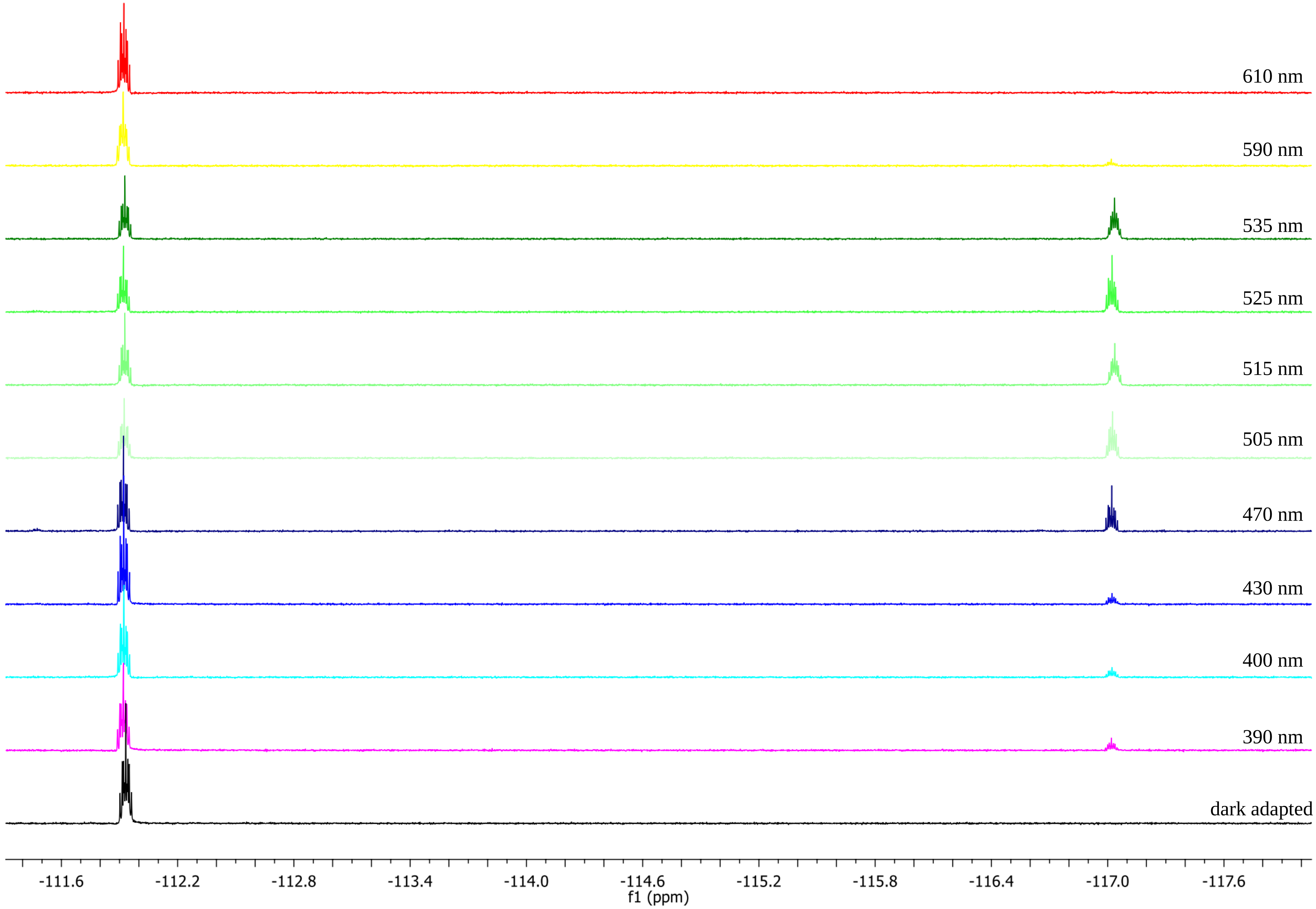

Figure S17. Partial ^{19}F NMR spectra of *o*-**3b** at dark adapted state and after irradiation with light at different wavelength in DMSO-d_6 (c ≈ 2 mM).

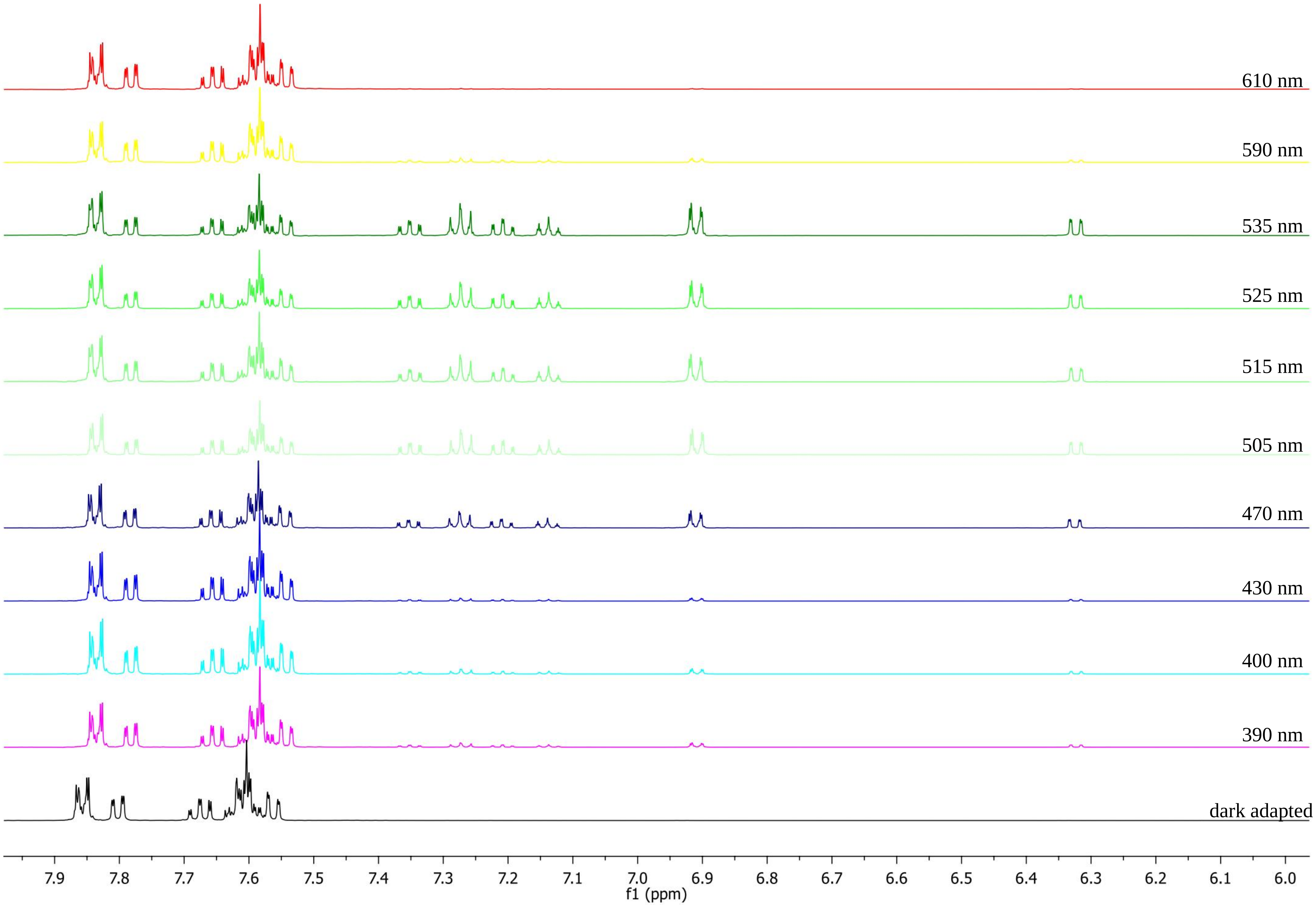

Figure S18. Partial ^{1}H NMR spectra of *o*-**3a** at dark adapted state and after irradiation with light at different wavelength in DMSO-d_6 (c ≈ 2 mM).

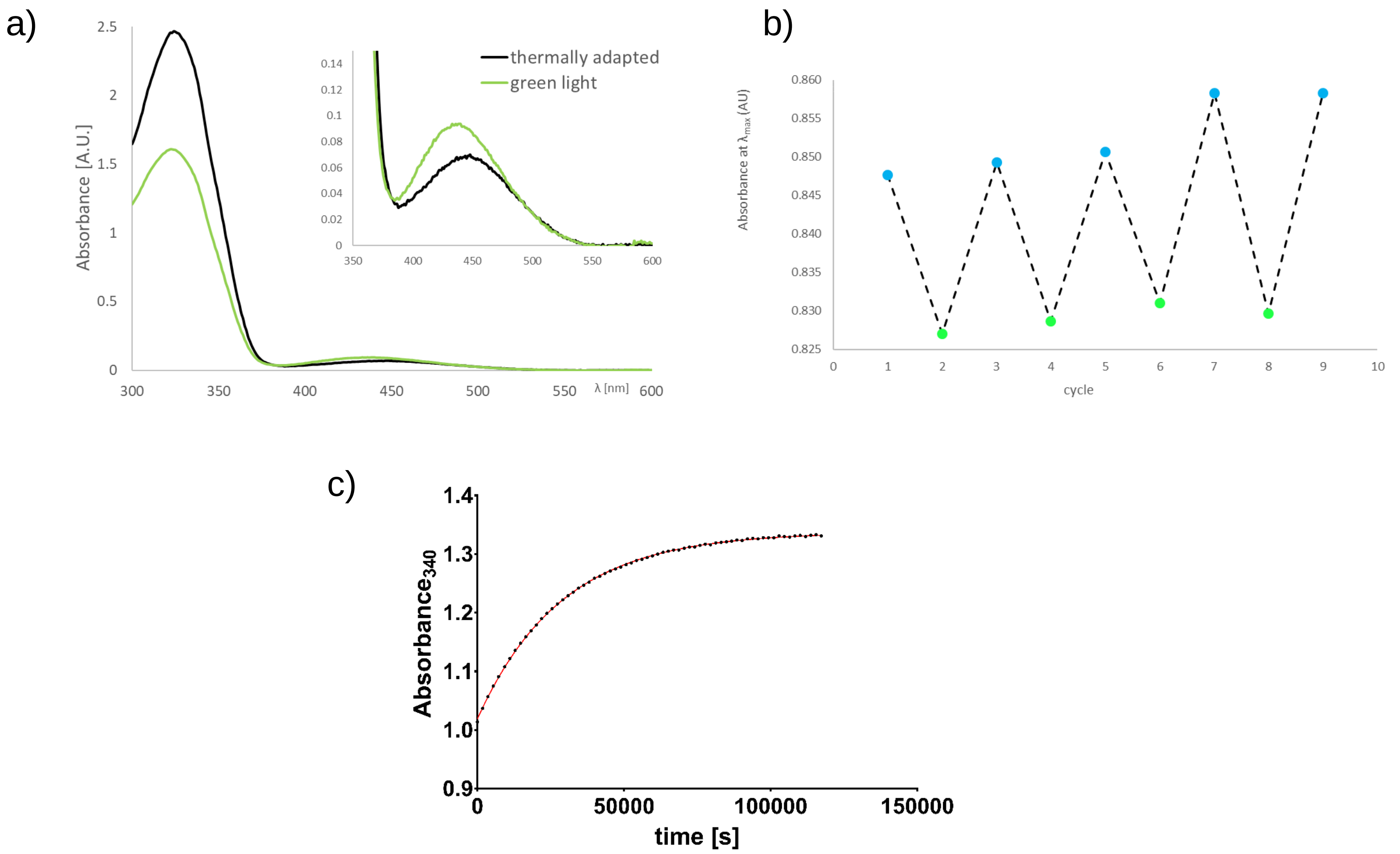

Figure S19. (**a**) UV-Vis absorption spectra of thermally adapted and green light irradiated 500 µM *o*-**3a** in DMSO. (**b**) Multiple photoswitching rounds of 50 µM *o*-**3a** in DMSO by pulsed green and blue light illumination. (**c**) Lifetime plot of *o*-**3a** based on following UV-Vis absorbance over time after irradiation with 535 nm light (37 °C, DMSO).

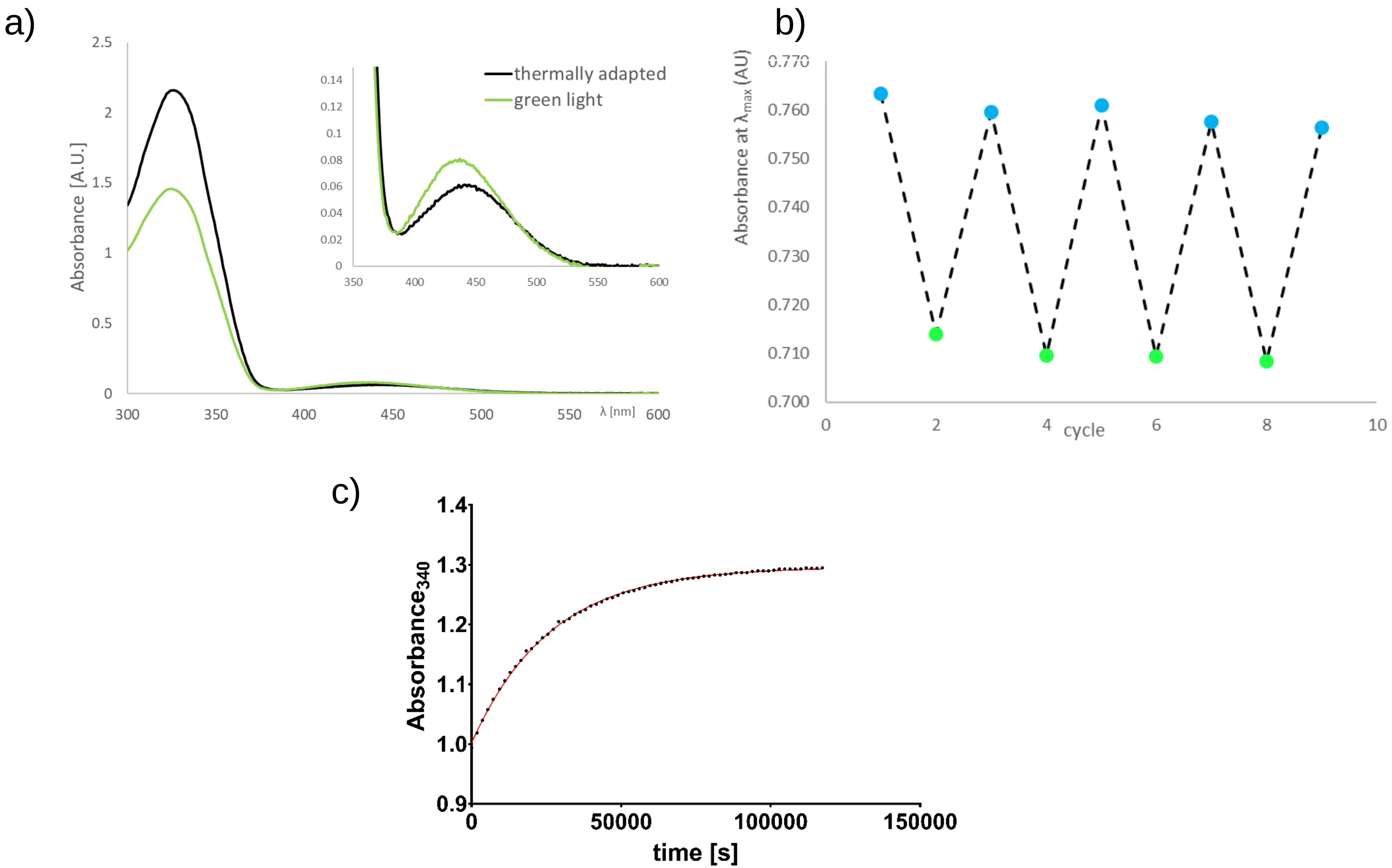

Figure S20. (**a**) UV-Vis absorption spectra of thermally adapted and green light irradiated 500 μM *o*-**3b** in DMSO. (**b**) Multiple photoswitching rounds of 50 μM *o*-**3b** in DMSO by pulsed green and blue light illumination. (**c**) Lifetime plot of *o*-**3b** based on following UV-Vis absorbance over time after irradiation with 530 nm light (37 °C, DMSO).

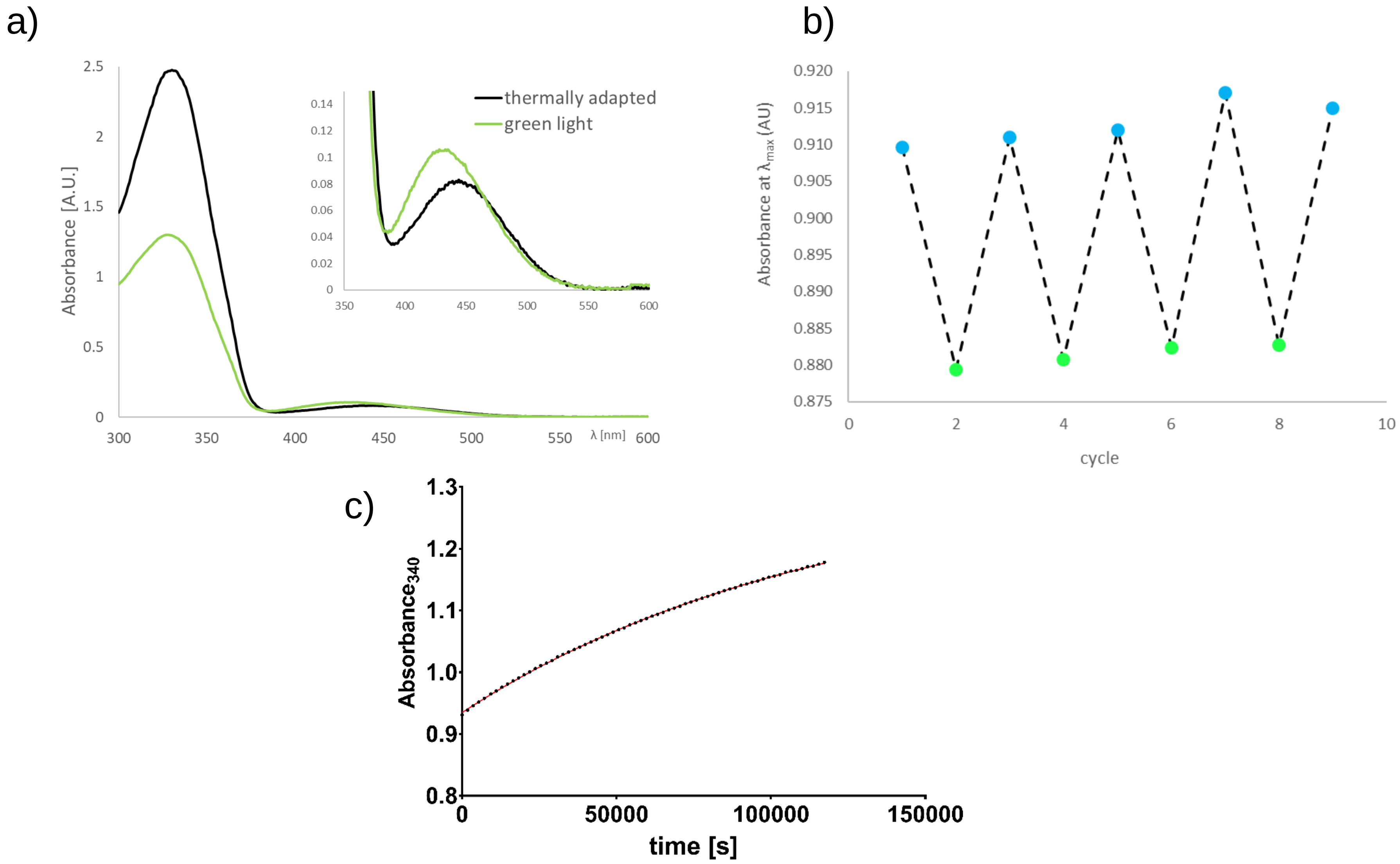

Figure S21. (**a**) UV-Vis absorption spectra of thermally adapted and green light irradiated 500 µM *o*-**3c** in DMSO. (**b**) Multiple photoswitching rounds of 50 µM *o*-**3c** in DMSO by pulsed green and blue light illumination. (**c**) Lifetime plot of *o*-**3c** based on following UV-Vis absorbance over time after irradiation with 530 nm light (37 °C, DMSO).

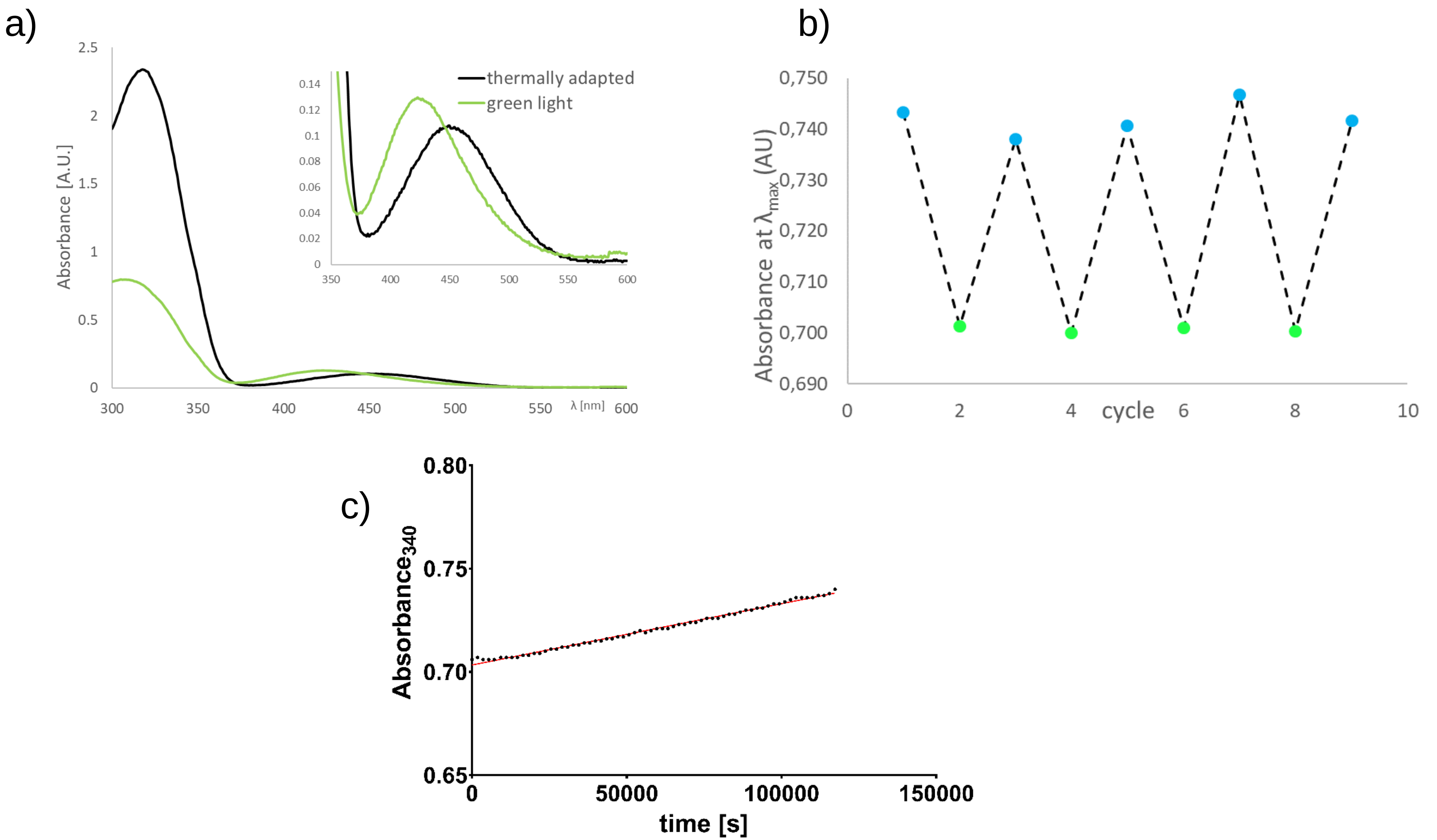

Figure S22. (**a**) UV-Vis absorption spectra of thermally adapted and green light irradiated 500 µM *o*-**3d** in DMSO. (**b**) Multiple photoswitching rounds of 50 µM *o*-**3d** in DMSO by pulsed green and blue light illumination. (**c**) Lifetime plot of *o*-**3d** based on following UV-Vis absorbance over time after irradiation with 530 nm light (37 °C, DMSO).

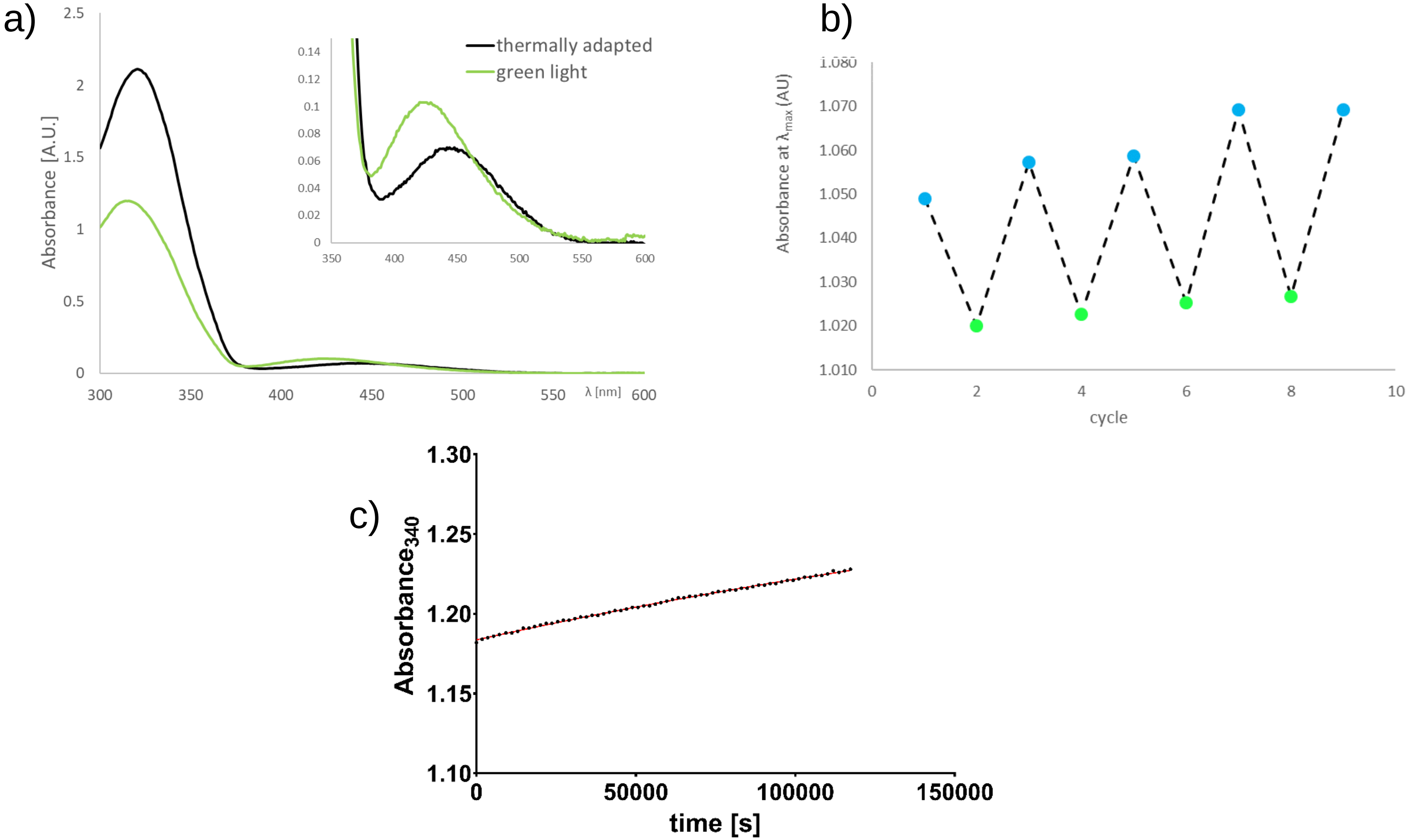

Figure S23. (**a**) UV-Vis absorption spectra of thermally adapted and green light irradiated 500 µM *m*-**3a** in DMSO. (**b**) Multiple photoswitching rounds of 50 µM *m*-**3a** in DMSO by pulsed green and blue light illumination. (**c**) Lifetime plot of *m*-**3a** based on following UV-Vis absorbance over time after irradiation with 530 nm light (37 °C, DMSO)

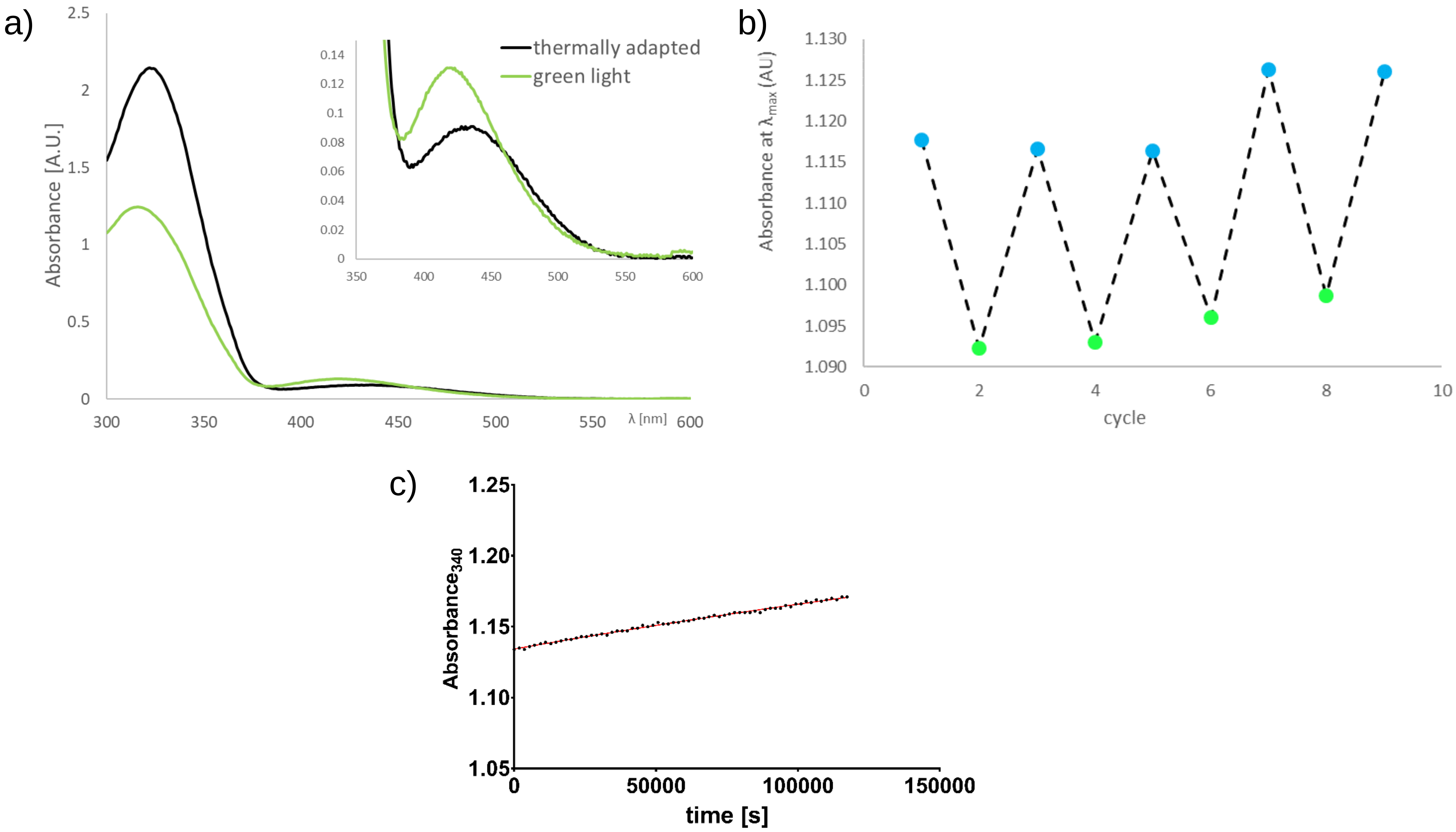

Figure S24. (**a**) UV-Vis absorption spectra of thermally adapted and green light irradiated 500 µM *m*-**3b** in DMSO. (**b**) Multiple photoswitching rounds of 50 µM *m*-**3b** in DMSO by pulsed green and blue light illumination. (**c**) Lifetime plot of *m*-**3b** based on following UV-Vis absorbance over time after irradiation with 530 nm light (37 °C, DMSO)

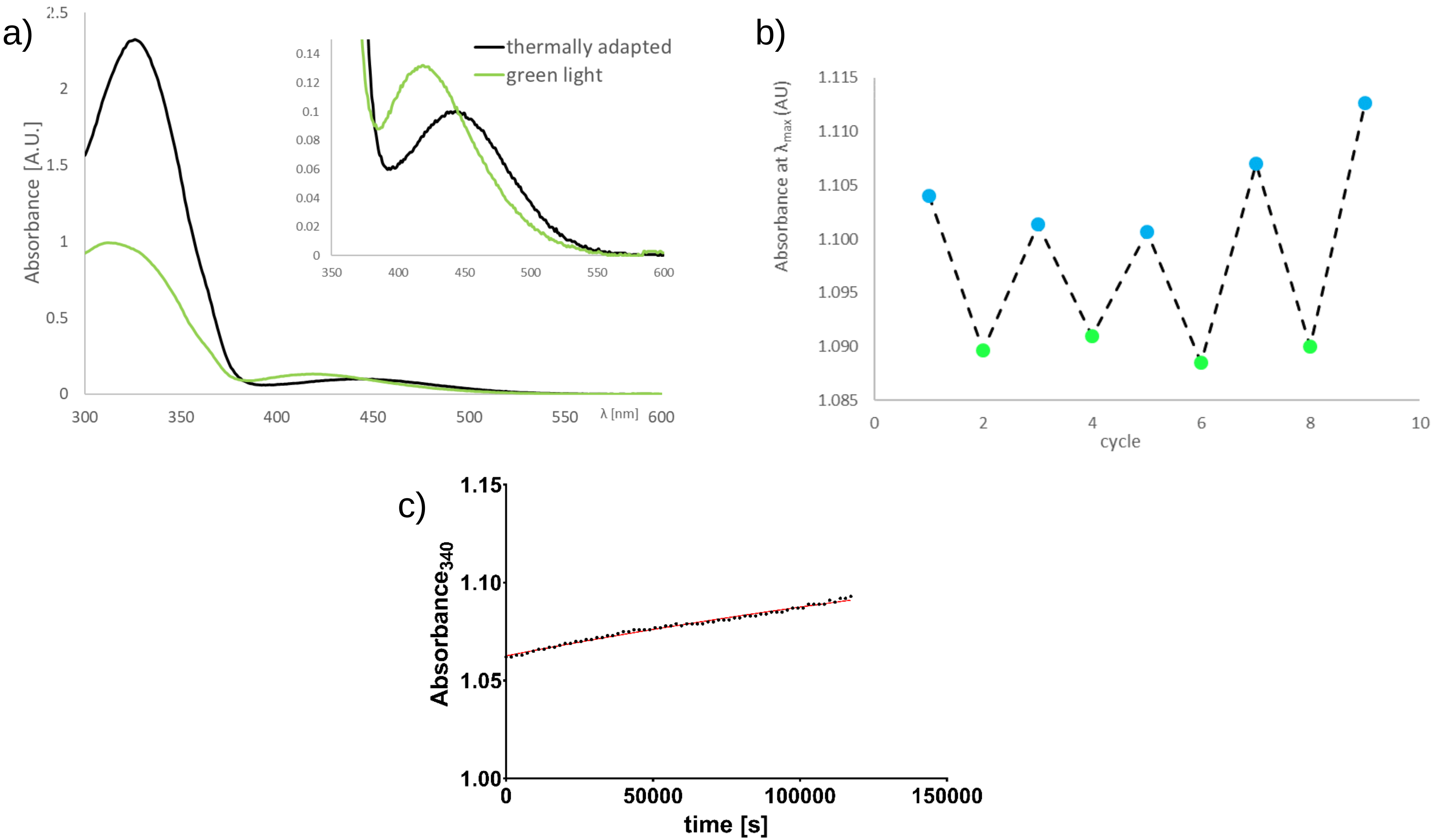

Figure S25. (**a**) UV-Vis absorption spectra of thermally adapted and green light irradiated 500 µM *m*-**3c** in DMSO. (**b**) Multiple photoswitching rounds of 50 µM *m*-**3c** in DMSO by pulsed green and blue light illumination. (**c**) Lifetime plot of *m*-**3c** based on following UV-Vis absorbance over time after irradiation with 530 nm light (37 °C, DMSO)

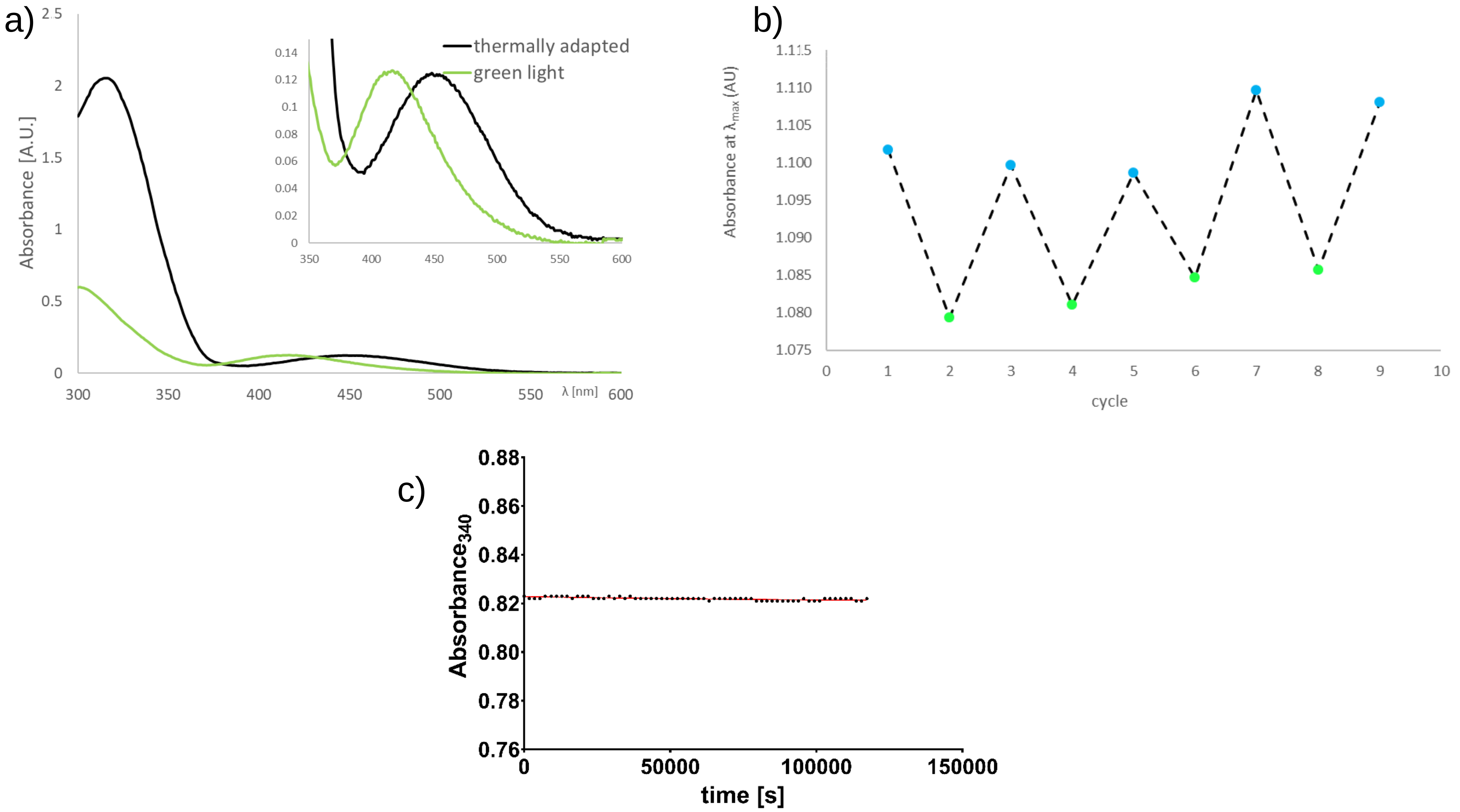

Figure S26. (**a**) UV-Vis absorption spectra of thermally adapted and green light irradiated 500 µM *m*-**3d** in DMSO. (**b**) Multiple photoswitching rounds of 50 µM *m*-**3d** in DMSO by pulsed green and blue light illumination. (**c**) Lifetime plot of *m*-**3d** based on following UV-Vis absorbance over time after irradiation with 530 nm light (37 °C, DMSO)

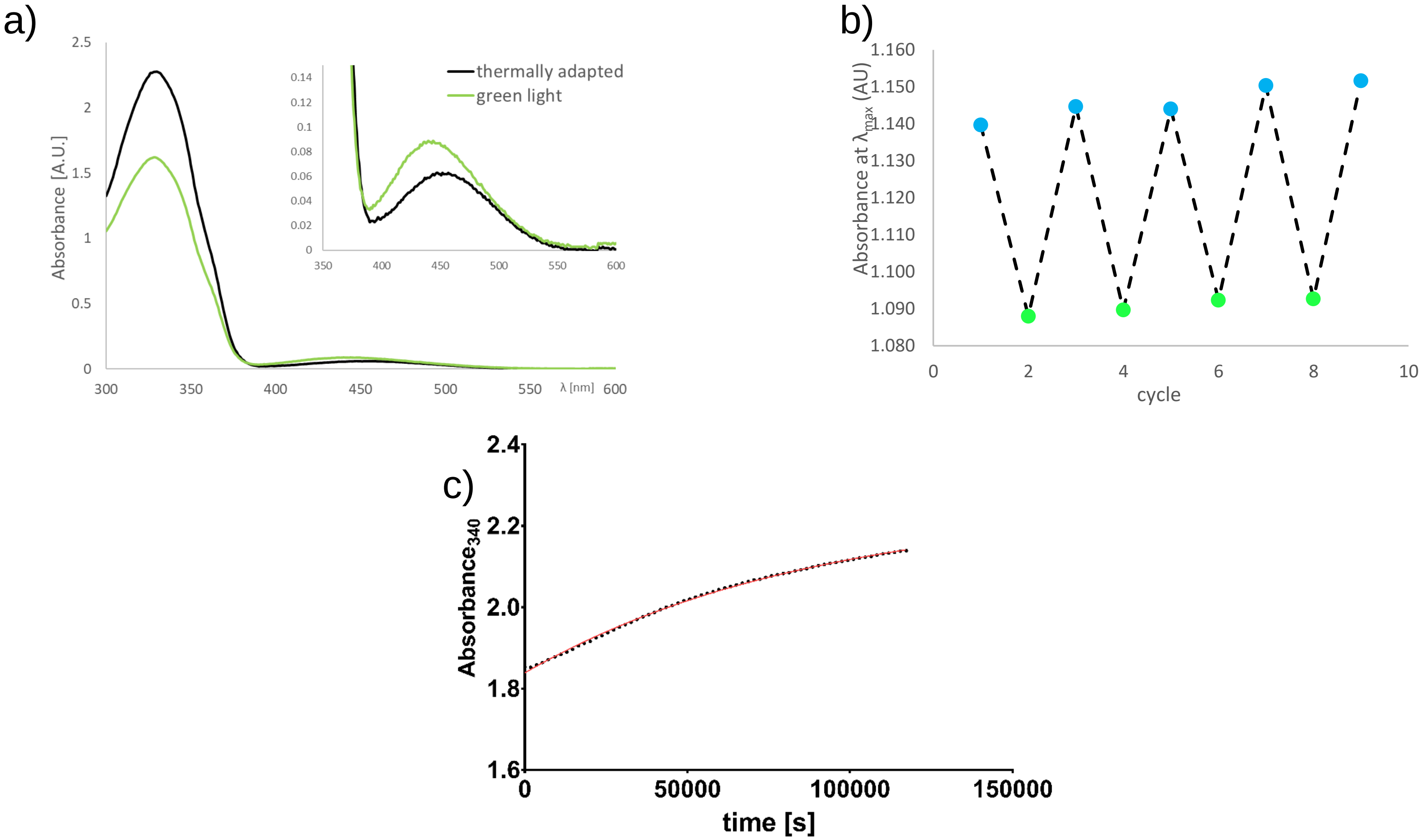

Figure S27. (**a**) UV-Vis absorption spectra of thermally adapted and green light irradiated 500 µM *p*-**3a** in DMSO. (**b**) Multiple photoswitching rounds of 50 µM *p*-**3a** in DMSO by pulsed green and blue light illumination. (**c**) Lifetime plot of *p*-**3a** based on following UV-Vis absorbance over time after irradiation with 530 nm light (37 °C, DMSO)

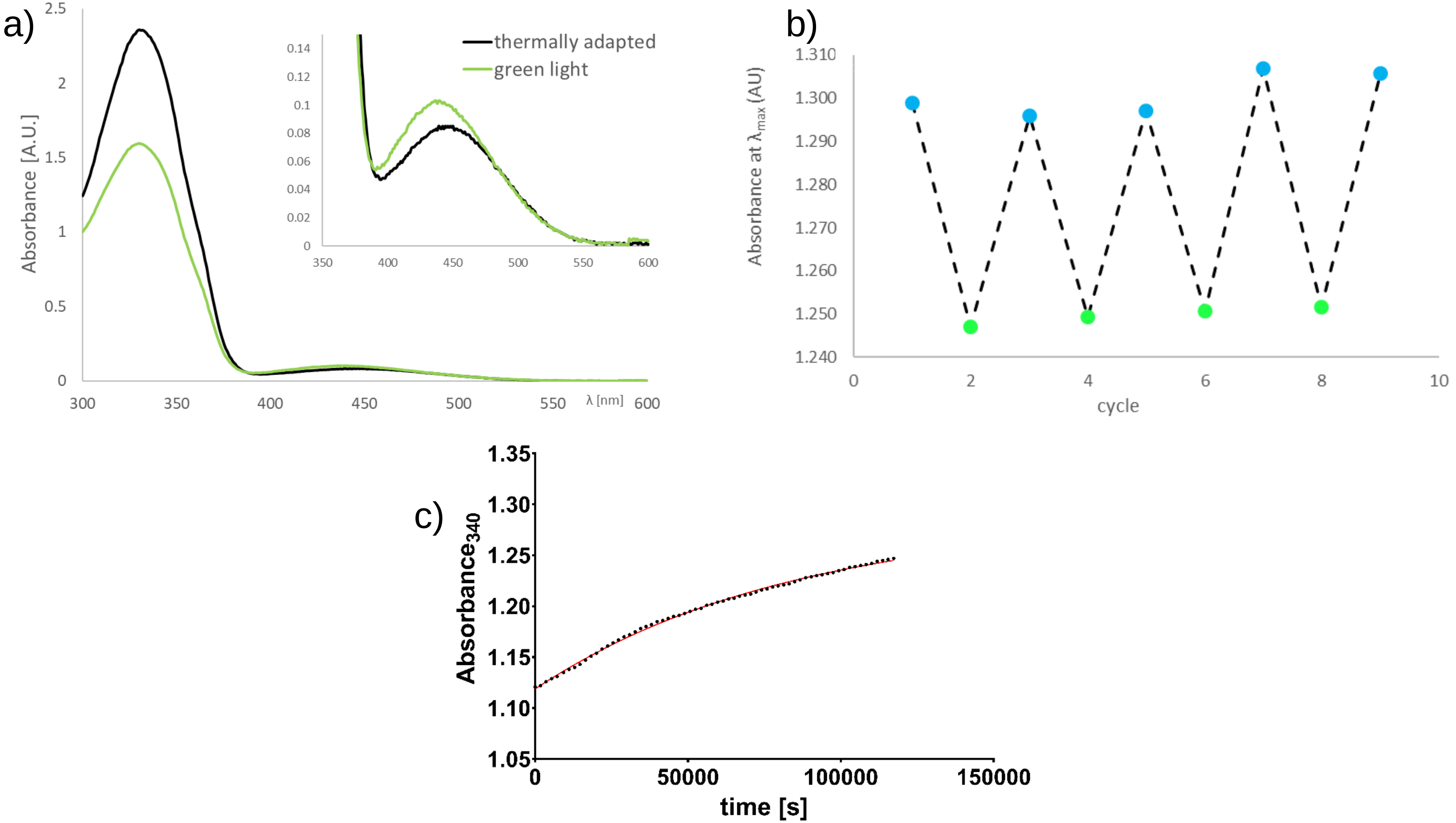

Figure S28. (**a**) UV-Vis absorption spectra of thermally adapted and green light irradiated 500 µM *p*-**3b** in DMSO. (**b**) Multiple photoswitching rounds of 50 µM *p*-**3b** in DMSO by pulsed green and blue light illumination. (**c**) Lifetime plot of *p*-**3b** based on following UV-Vis absorbance over time after irradiation with 530 nm light (37 °C, DMSO)

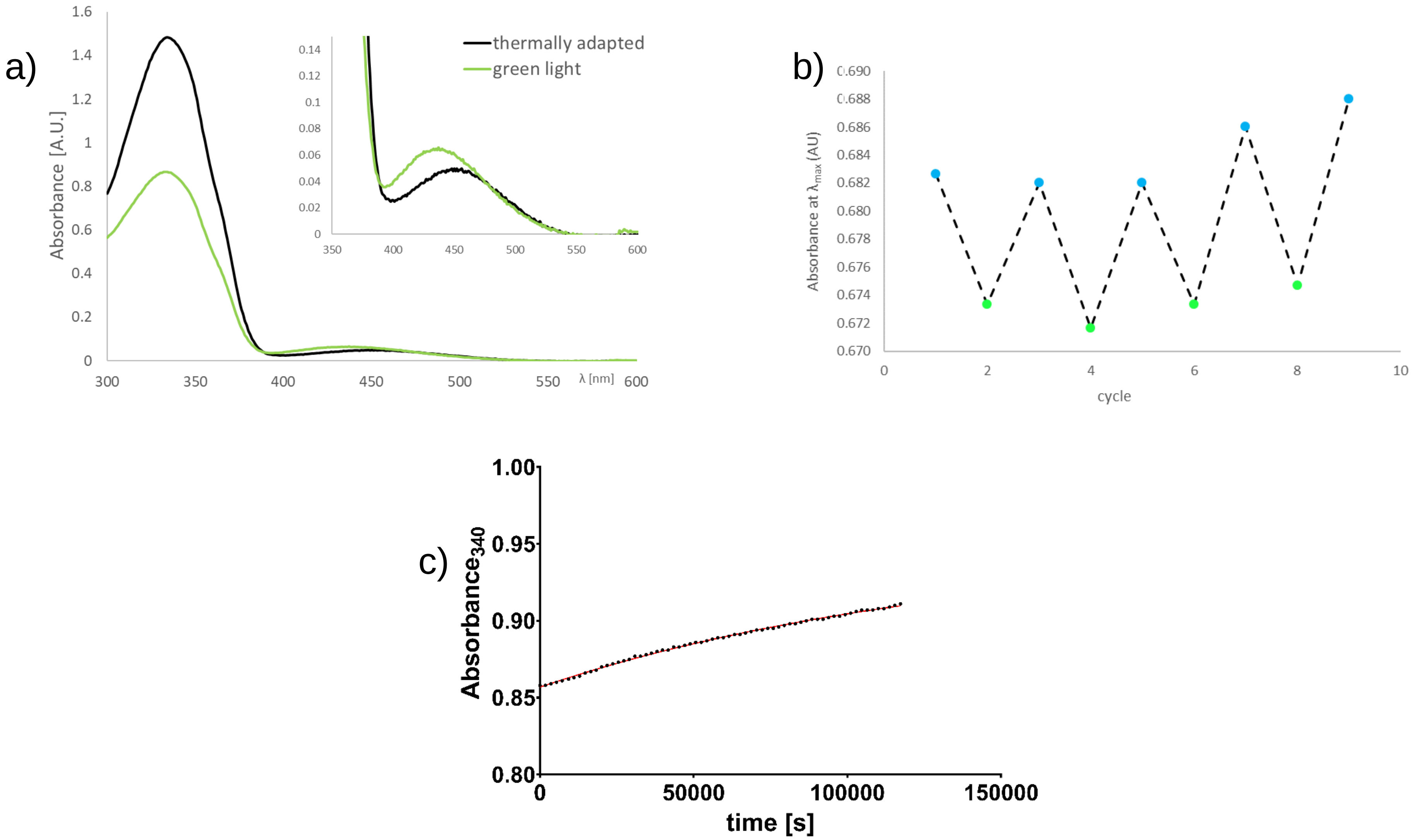

Figure S29. (**a**) UV-Vis absorption spectra of thermally adapted and green light irradiated 500 µM *p*-**3c** in DMSO. (**b**) Multiple photoswitching rounds of 50 µM *p*-**3c** in DMSO by pulsed green and blue light illumination. (**c**) Lifetime plot of *p*-**3c** based on following UV-Vis absorbance over time after irradiation with 530 nm light (37 °C, DMSO)

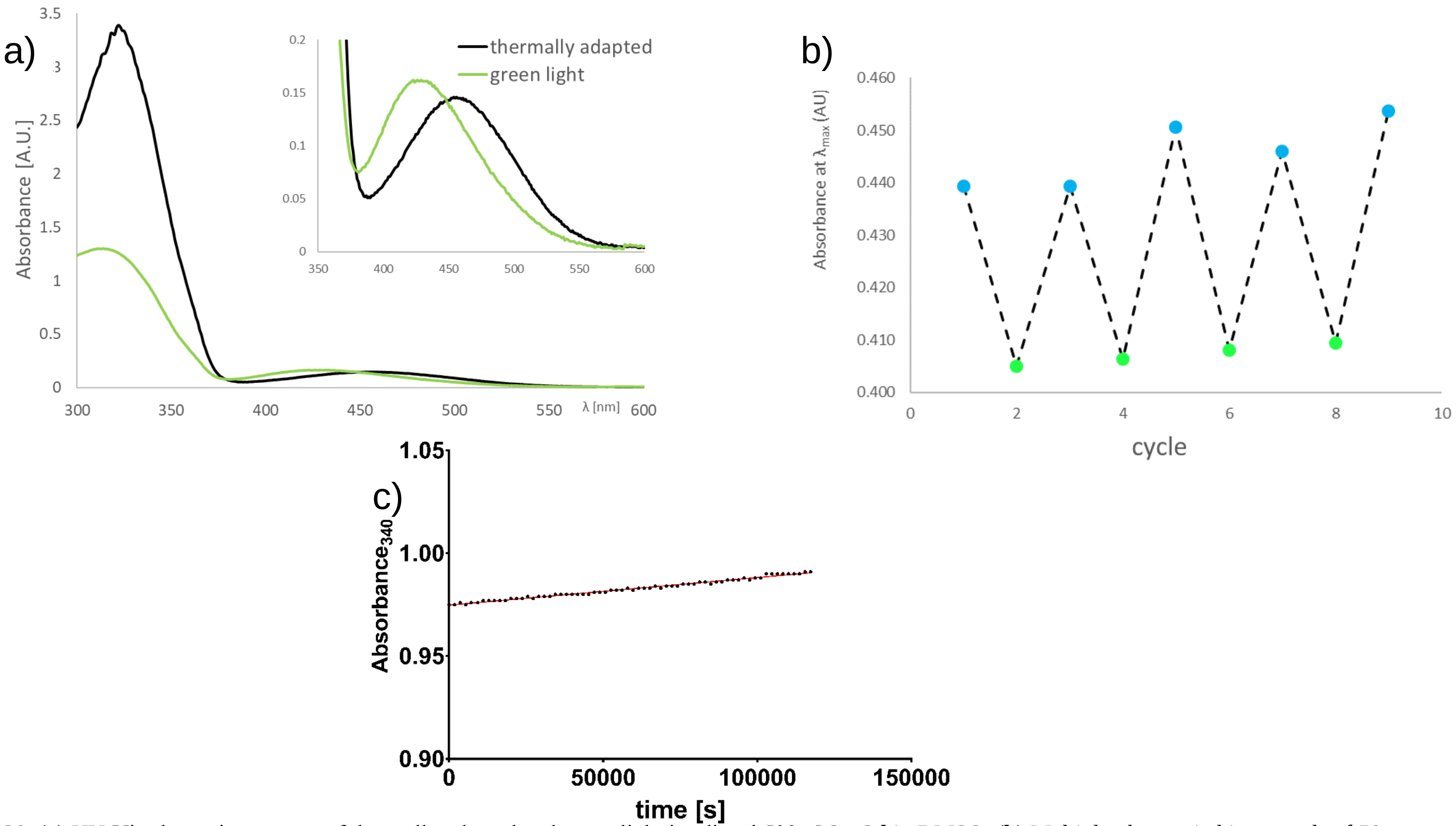

Figure S30. (**a**) UV-Vis absorption spectra of thermally adapted and green light irradiated 500 µM *p*-**3d** in DMSO. (**b**) Multiple photoswitching rounds of 50 µM *p*-**3d** in DMSO by pulsed green and blue light illumination. (**c**) Lifetime plot of *p*-**3d** based on following UV-Vis absorbance over time after irradiation with 530 nm light (37 °C, DMSO)

BIOLOGICAL ASSAYS

Tubulin polymerisation assay:
Tubulin from porcine brain was purified according to protocol published earlier [46]. The tubulin polymerisation reaction was conducted at 3.5 mg/mL tubulin, in tubulin polymerisation buffer (80 mM piperazine-N,N′-bis(2-ethanesulfonic acid) (PIPES) pH = 6.9; 0.5 mM EGTA; 2 mM $MgCl_2$), in a 96-well plate (100 µL final volume) in a EnSpire® multimode plate reader (PerkinElmer, Turku, Finland) with temperature maintained at 37 °C. Tubulin was initially preincubated for 30 min at room temperature with (*Z*) enriched isomer (green light pre-illuminated) or with all (*E*) isomer (thermally adapted in dark) of *o*-AzoCol26DF (final concentration 10 µM) in buffer with 1% DMSO, without GTP. Sample with 1% DMSO alone was used as a control Then GTP was added to concentration 1 mM, and the change in absorbance at 340 nm was monitored with 15 s intervals for 20 minutes.

Cell Culturing:
Cells were cultured in a humidified incubator at 37 °C under 5% CO2. Human breast adenocarcinoma (MCF-7) cells were maintained in phenol red-free Dulbecco’s Modified Eagle’s Medium (Thermo Fisher Scientific, 11054020) supplemented with 2 mM L-glutamine and human colorectal carcinoma (HCT116) cells were maintained in the same medium but supplemented with 4.5 g/L glucose and 4 mM L-glutamine. All culture media were supplemented with 1% Penicillin-Streptomycin (Sigma-Aldrich, P4333) and 10% fetal bovine serum (Gibco, 10270-106). Cells were sub-cultured at approximately 70–90% confluency to maintain the culture in the logarithmic growth phase.

MTT Cytotoxicity Assay with green and blue light irradiation:
Cells were seeded at a 96-well plate at the density of 7×10^3 cells per well and allowed to grow for 24 h. Afterward, the medium was aspirated and fresh medium was added (200 µL) with serial dilutions of tested compounds or DMSO at corresponding concentrations as a control. Following 48 h of incubation under 500 ms pulsed green or blue light irradiation every 15 seconds, the medium was replaced with medium (100 µL) containing MTT (3-(4,5-dimethylthiazol-2-yl)-2,5-diphenyl-2H-tetrazolium bromide, 0.5 mg/mL) and incubated at 37 °C for 4 hours. The formed formazan was dissolved in DMSO (100 µL) and incubated at 37 °C for 10 min. The absorbance was measured at 540 nm. After blank subtraction, the half maximal effective concentration (IC_{50}) was calculated by GraphPad Prism software (GraphPad Software Inc, San Diego, CA, USA). Each independent experiment was performed in triplicate

Immunofluorescence:
Cells were seeded on coverslips on a 24-well plate at a density of 5 × 104 cells per well and allowed to grow overnight. Next, the medium was replaced with a medium containing the o-**AzoCol26DF** or the colchicine (without illumination) or DMSO as control and incubated for 24 h under 500 ms pulsed green or blue light irradiation every 15 s. Then, cells were fixed and permeabilised with 100% methanol at −20 °C for 15 min and subsequently washed three times with PBS at room temperature. After 1 h blocking with 3% BSA/PBS at 4 °C, slides were incubated with anti-αalpha tubulin 12G10 antibody (Developmental Studies Hybridoma Bank, University of Iowa, Iowa City, IA, USA) (diluted 1:300 in 3% BSA/PBS) and anti-acetylated α- tubulin antibody (Cell Signalling, Danvers, MA, USA) (diluted 1:1000 in 3% BSA/PBS) overnight at 4 °C. After 3 × 10 min washing with PBS, slides were incubated with AlexaFlu-or555-conjugated anti-rabbit and AlexaFluor488-conjugated anti-mouse secondary an-tibodies (diluted 1:400 in 3% BSA/PBS) (Thermo Fisher Scientific, Waltham, MA, USA, A31570) and with DAPI (50 ng/mL) for 1 h at room temperature. After washing (3 × 10 min with PBS), slides were mounted in Fluoromount-G

(Southern Biotech., Birming-ham, AL, USA). Images were recorded using Leica TCS SP8 (Leica Microsystems, Wetzlar, Germany) confocal microscope and analysed using ImageJ 1.53t soft-ware

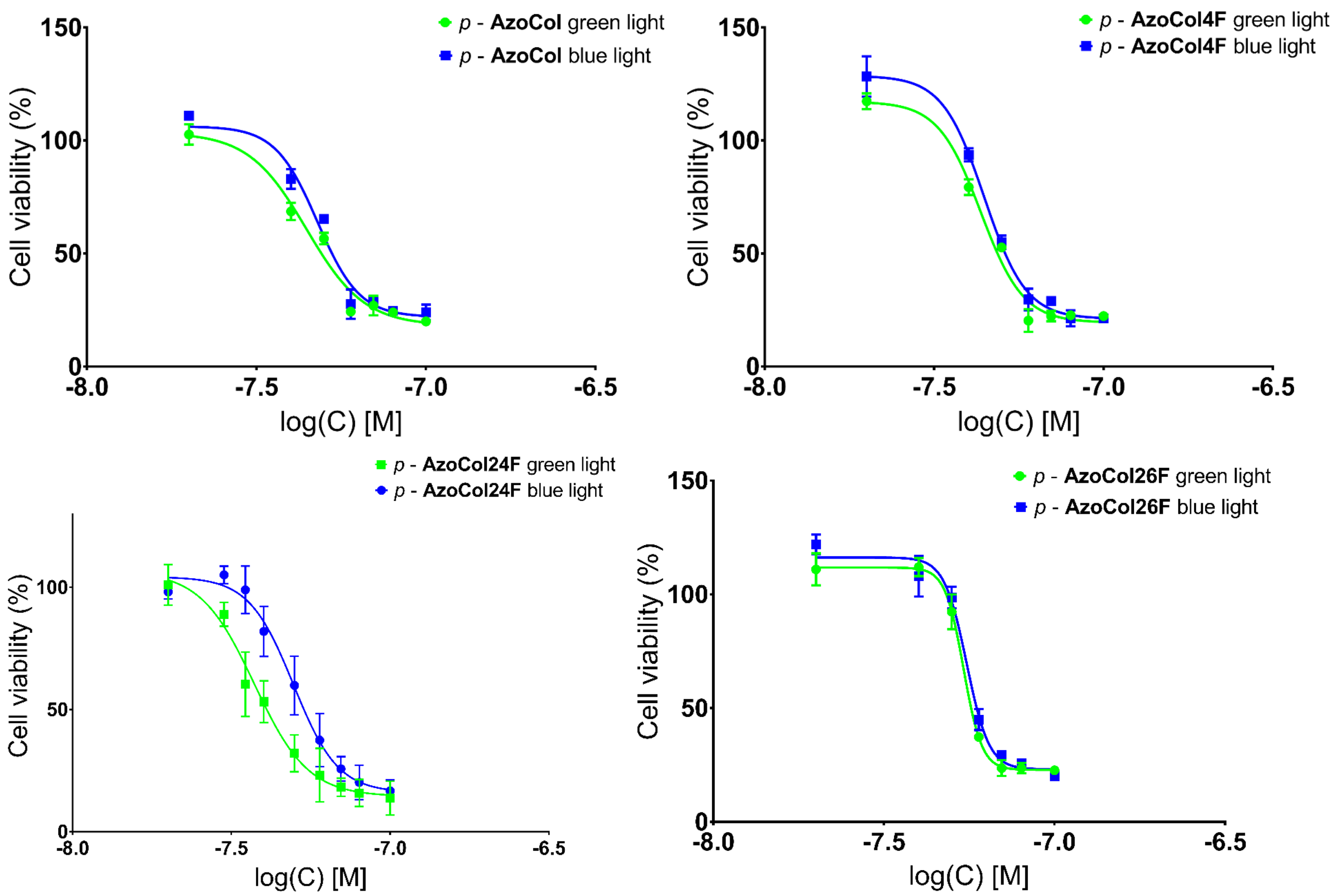

Figure S31. Representative viability of MCF-7 cells from MTT assays for *p*-**AzoCols**

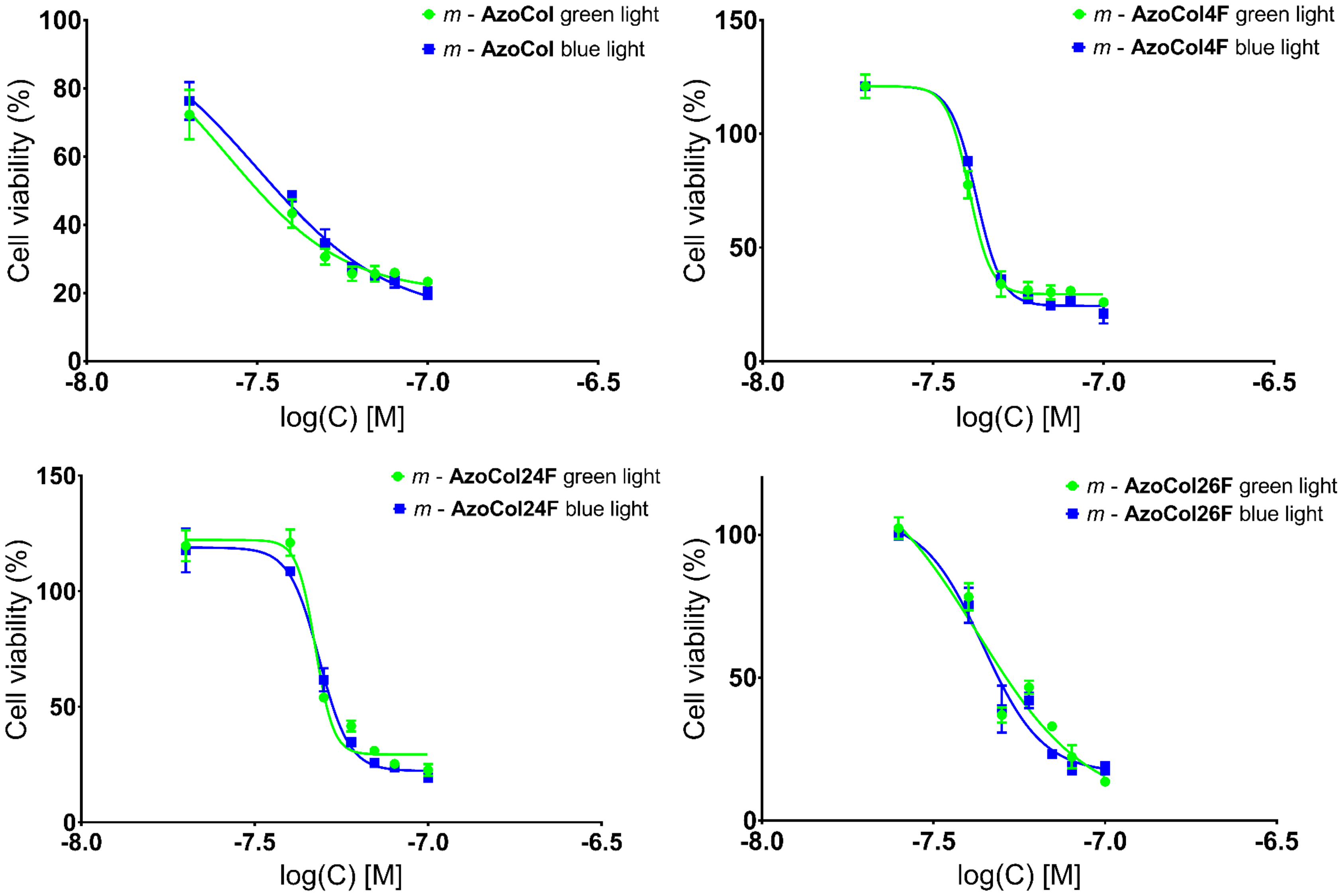

Figure S32. Viability of MCF-7 cells from MTT assays for *m*-**AzoCols**

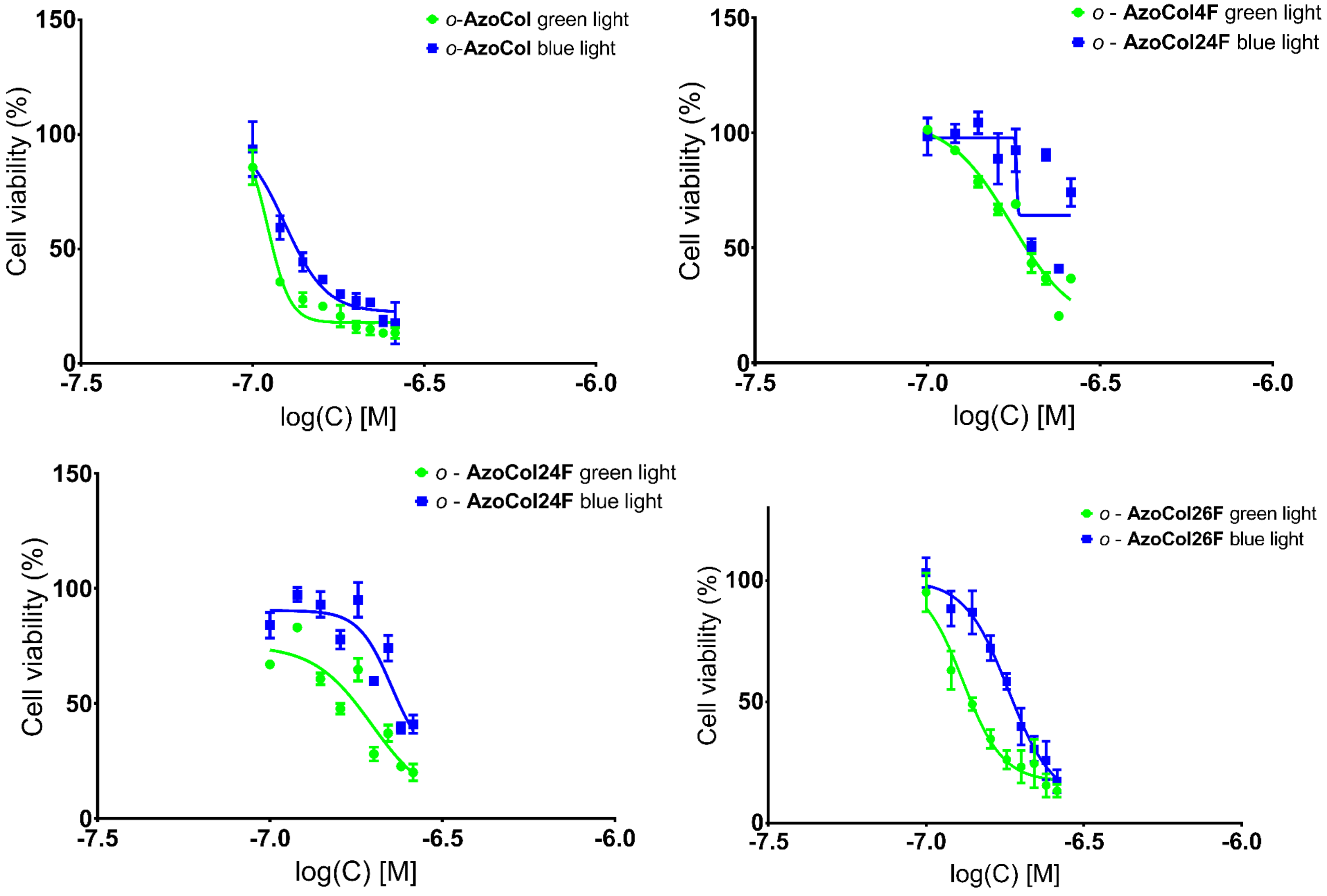

Figure S33. Viability of MCF-7 cells from MTT assays for *o*-**AzoCols**

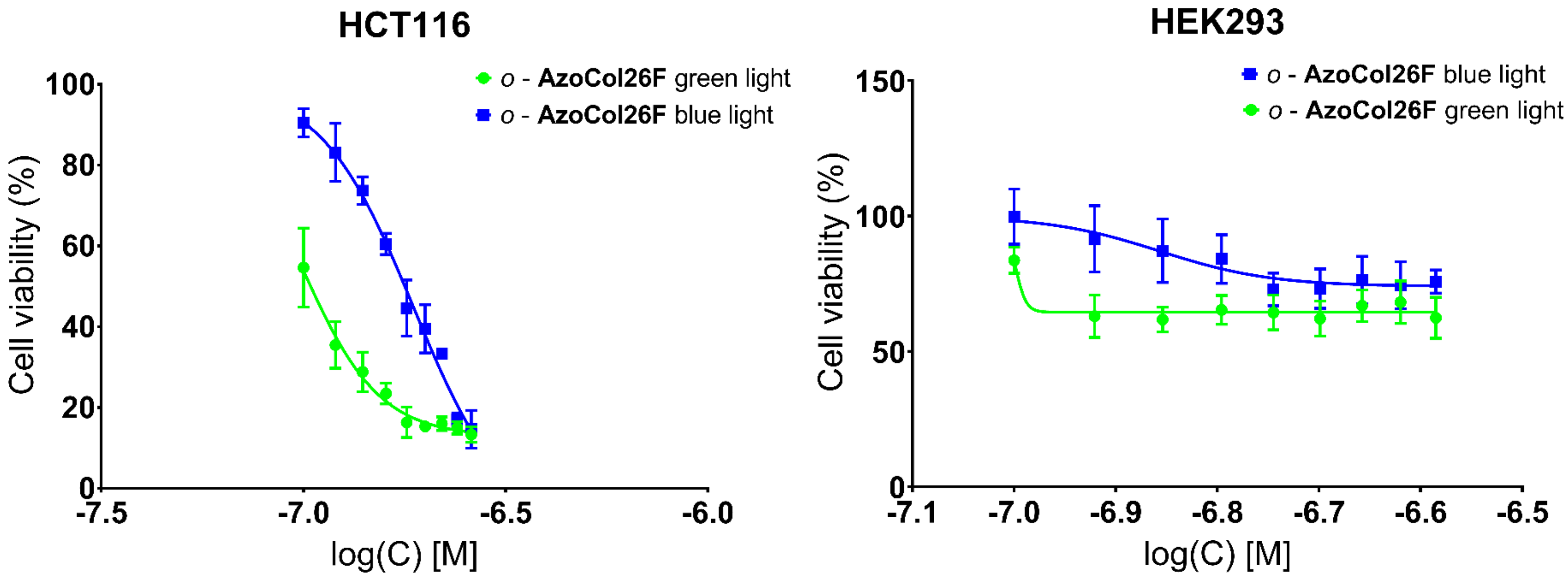

Figure S34. Representative viability of HCT116 and HEK293 cells from MTT assays for *o*-**AzoCol26DF**.

COPIES OF NMR SPECTRA

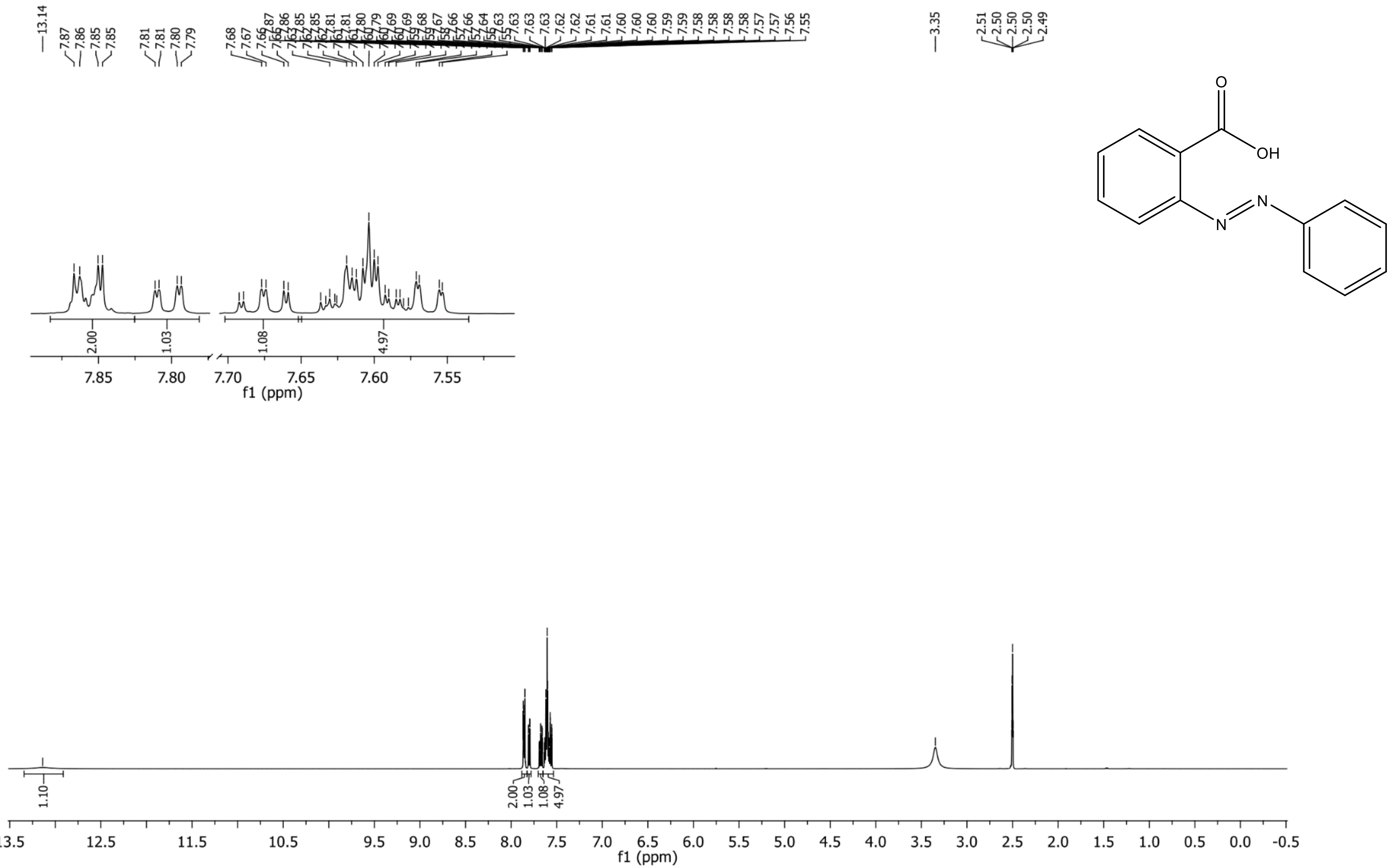

Figure S35. ^{1}H NMR spectra of *o*-**3a (**(*E*)-2-(phenyldiazenyl)benzoic acid).

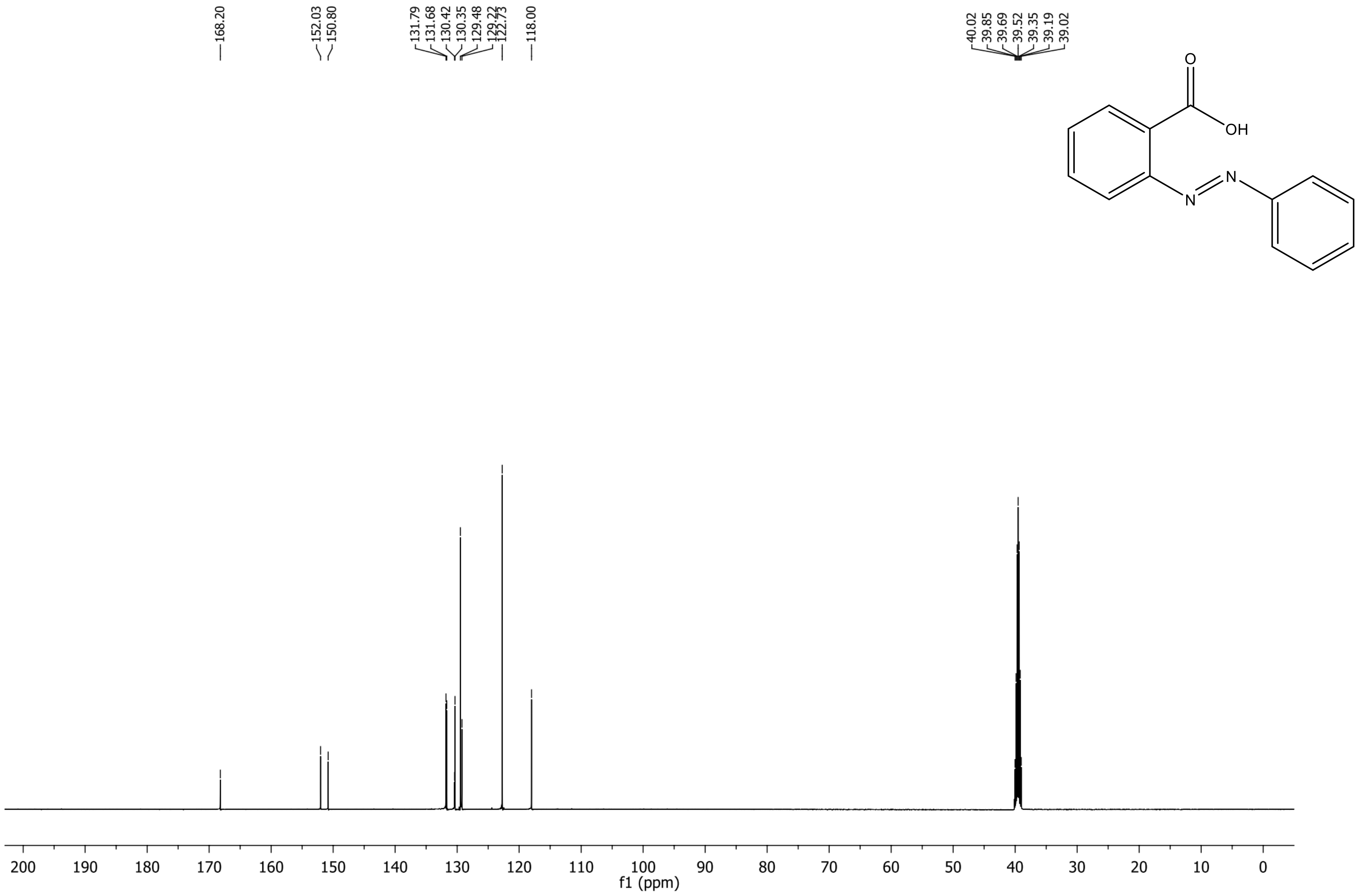

Figure S36. ^{13}C NMR spectra of *o*-**3a** ((*E*)-2-(phenyldiazenyl)benzoic acid).

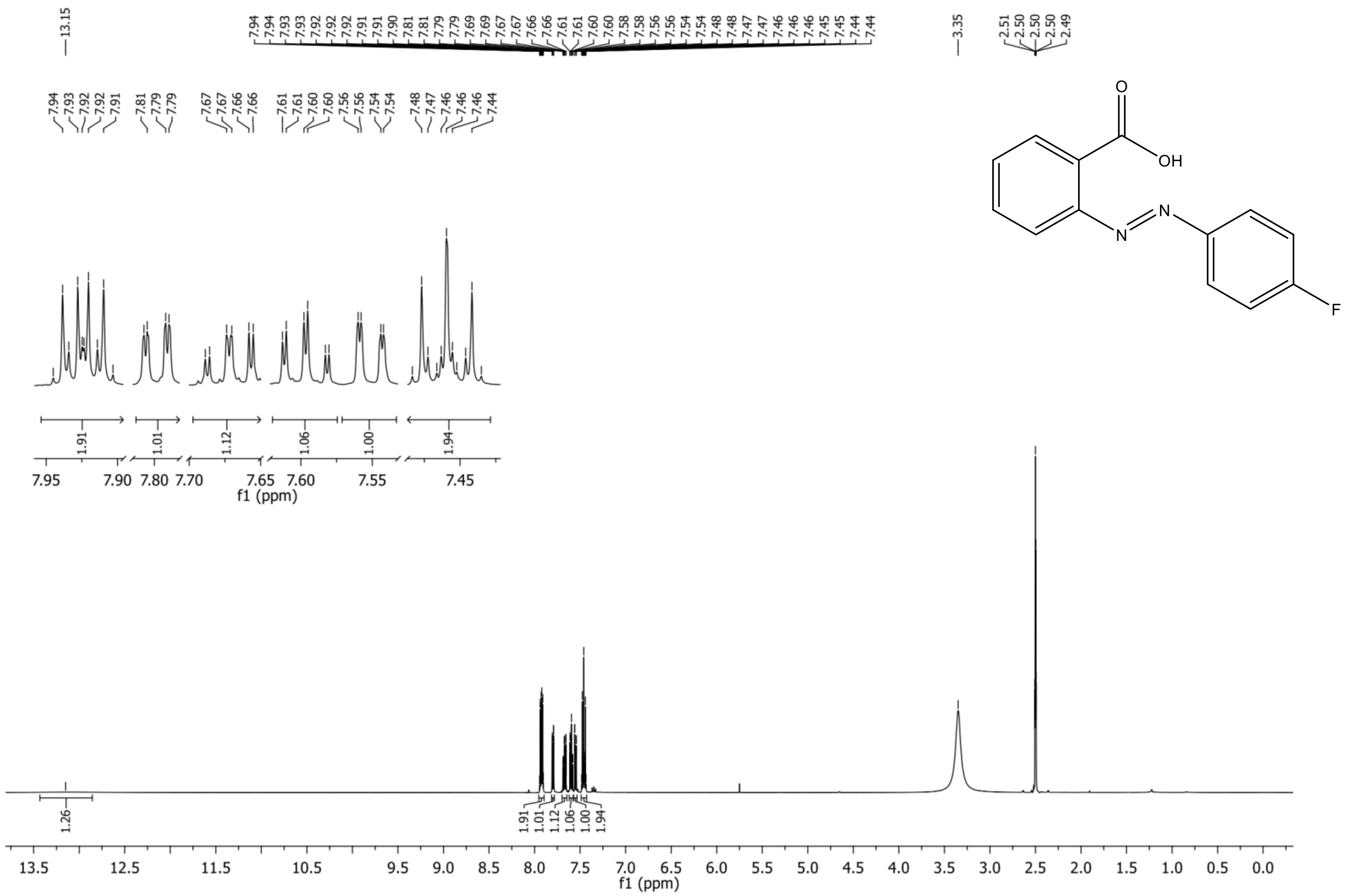

Figure S37. ^{1}H NMR spectra of *o*-**3b** ((*E*)-2-((4-fluorophenyl)diazenyl)benzoic).

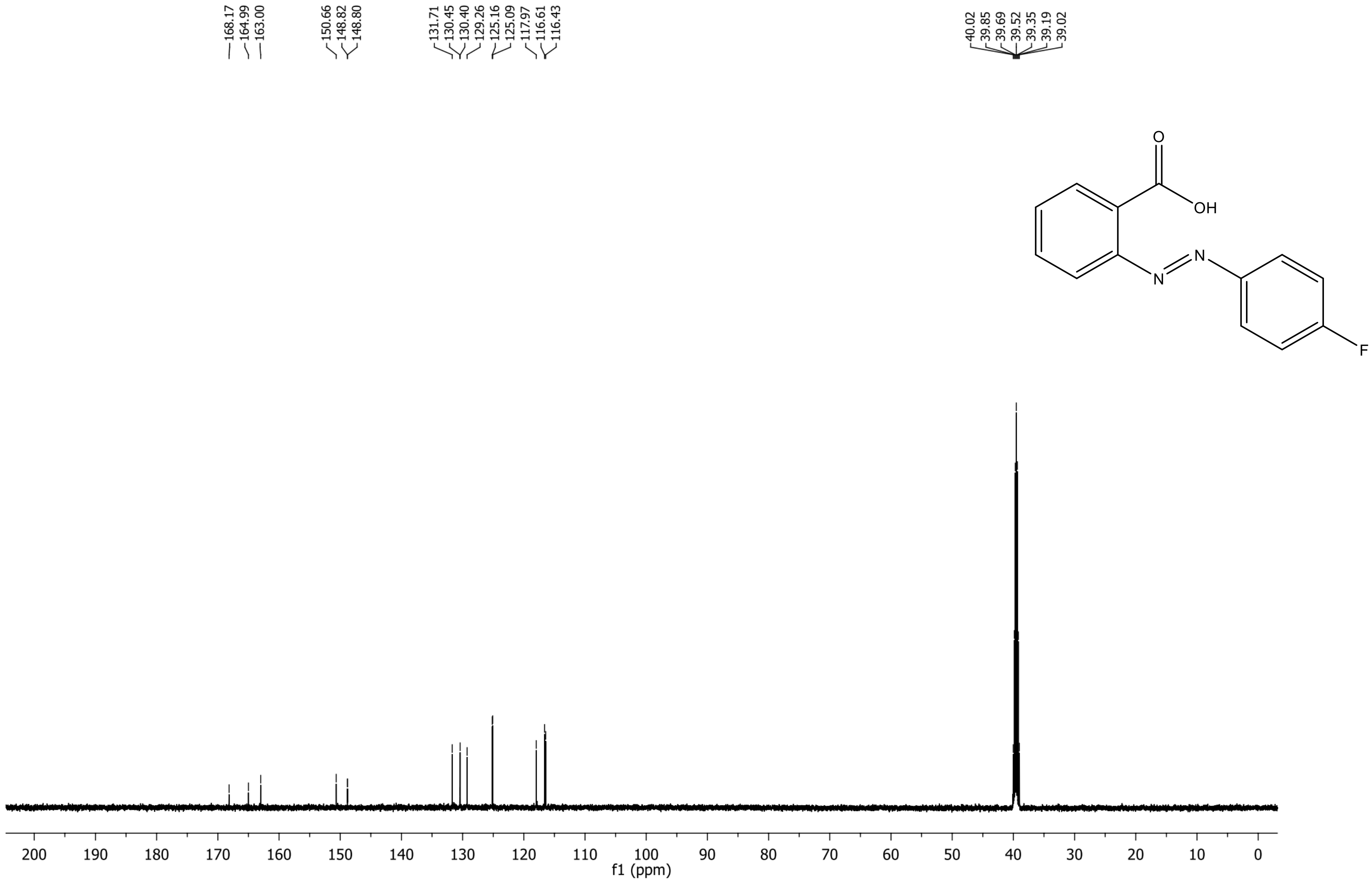

Figure S38. ^{13}C NMR spectra of *o*-**3b** ((*E*)-2-((4-fluorophenyl)diazenyl)benzoic).

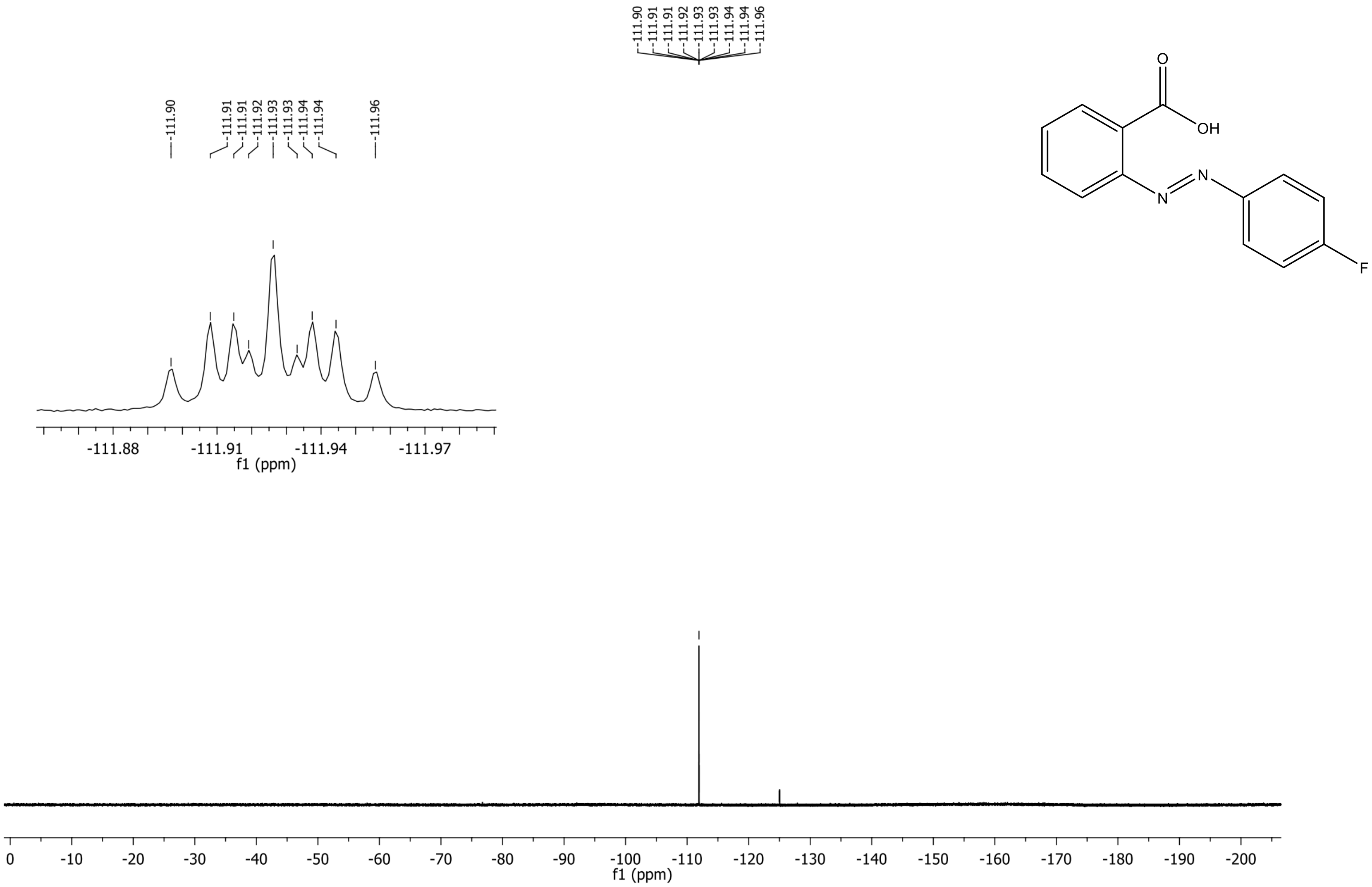

Figure S39. ^{19}F NMR spectra of *o*-**3b** ((*E*)-2-((4-fluorophenyl)diazenyl)benzoic).

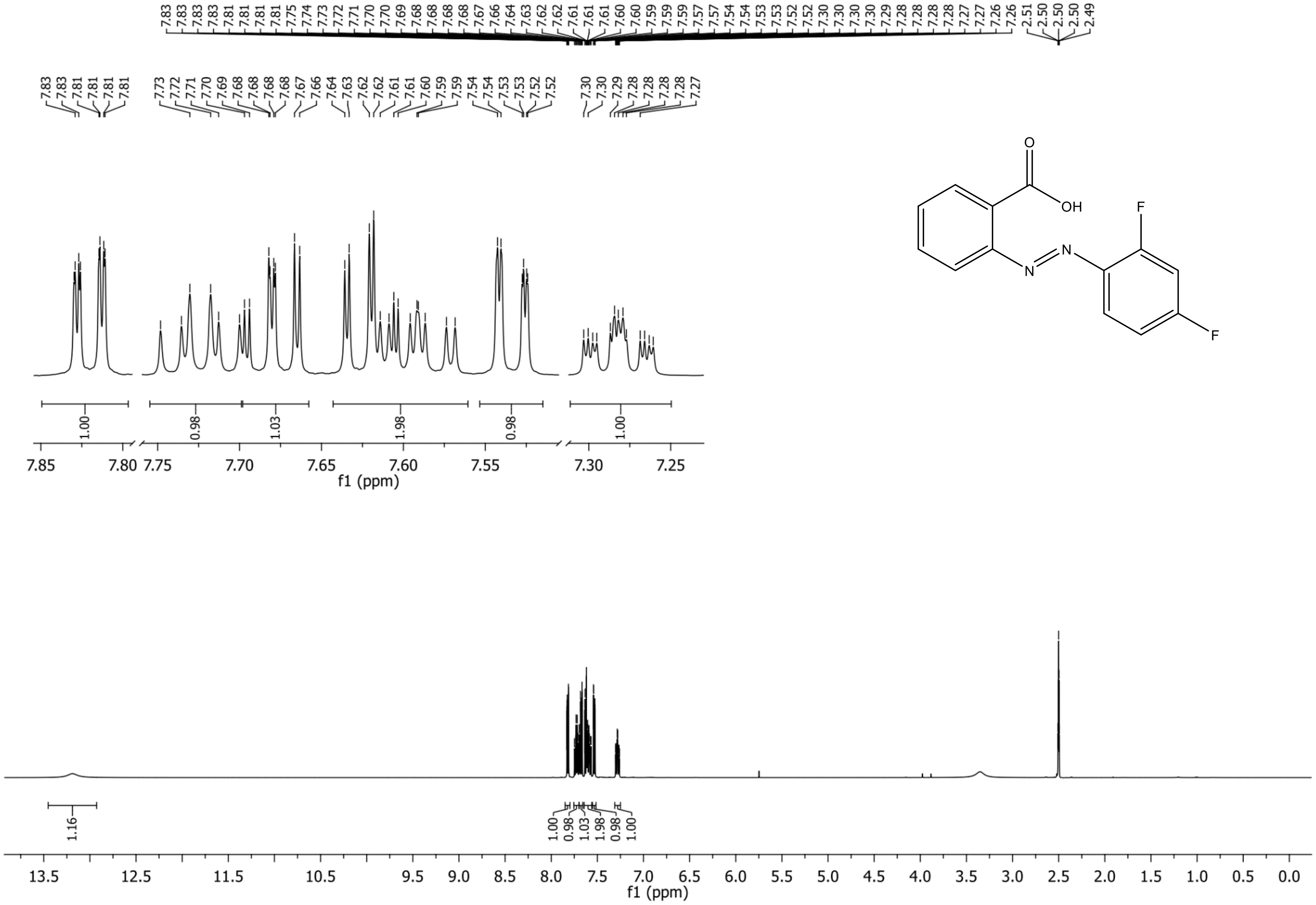

Figure S40. ^{1}H NMR spectra of *o*-**3c** ((*E*)-2-((2,4-difluorophenyl)diazenyl)benzoic acid).

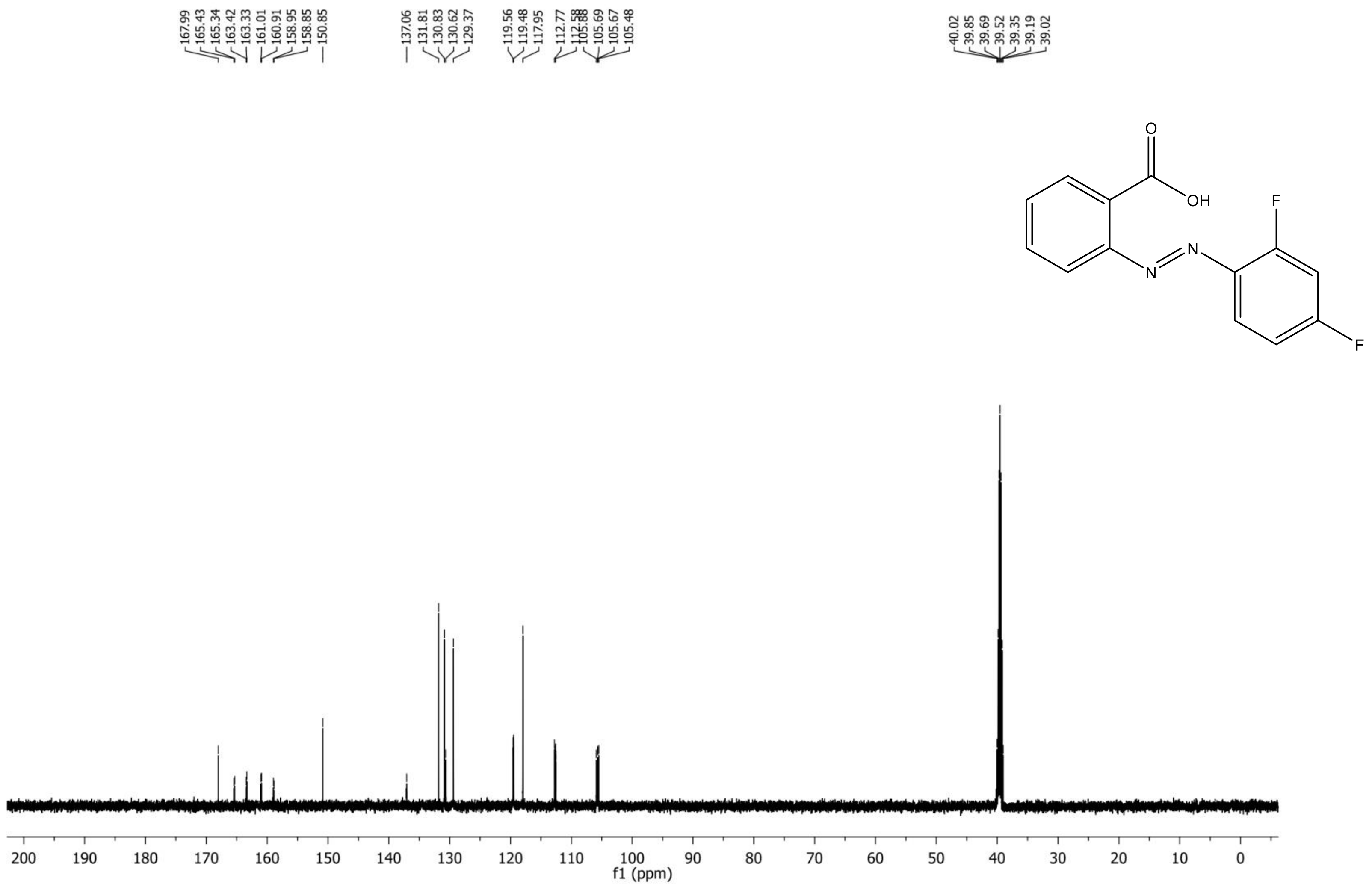

Figure S41. ^{13}C NMR spectra of *o*-**3c** ((*E*)-2-((2,4-difluorophenyl)diazenyl)benzoic acid).

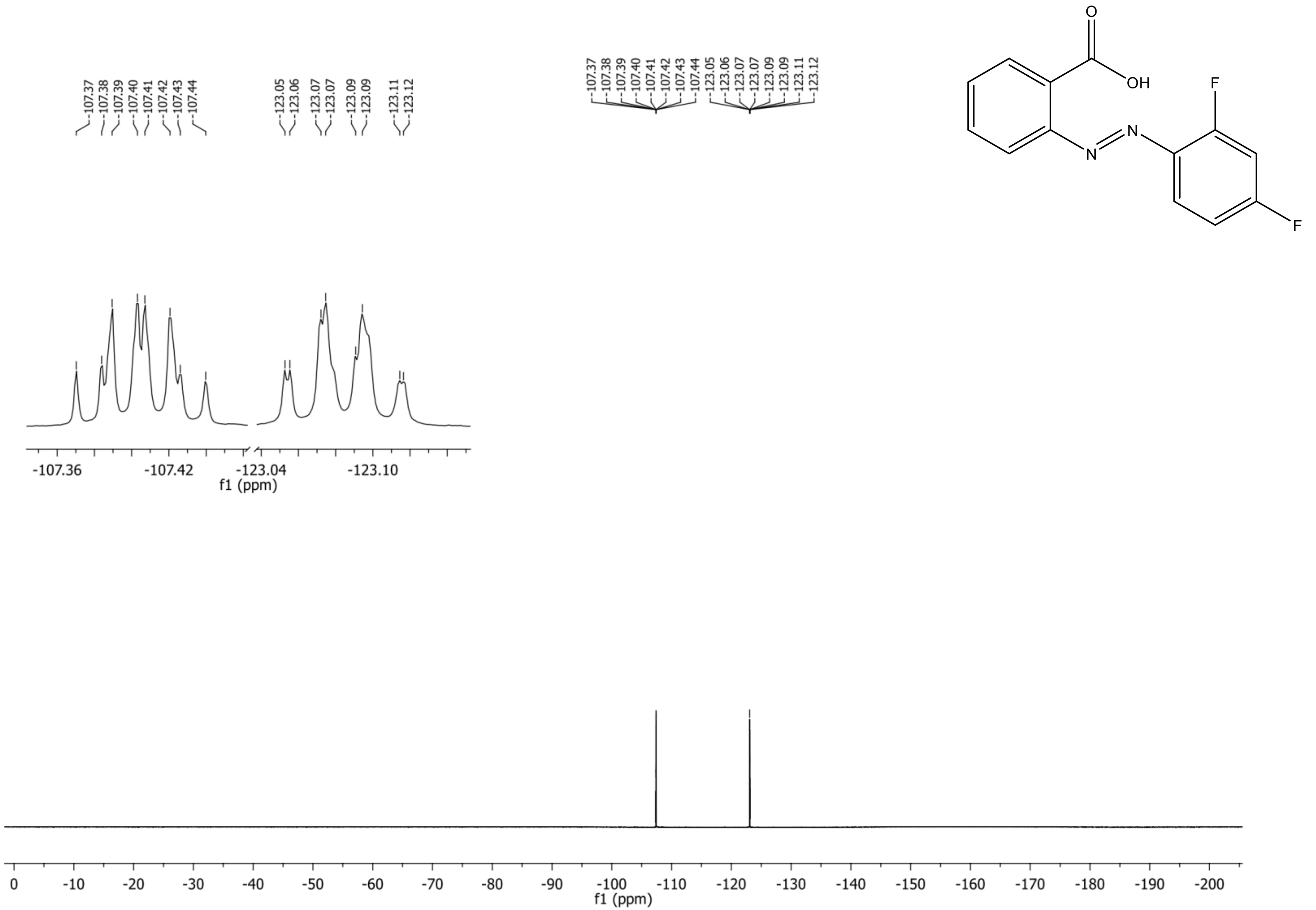

Figure S42. ^{19}F NMR spectra of *o*-**3c** ((*E*)-2-((2,4-difluorophenyl)diazenyl)benzoic acid).

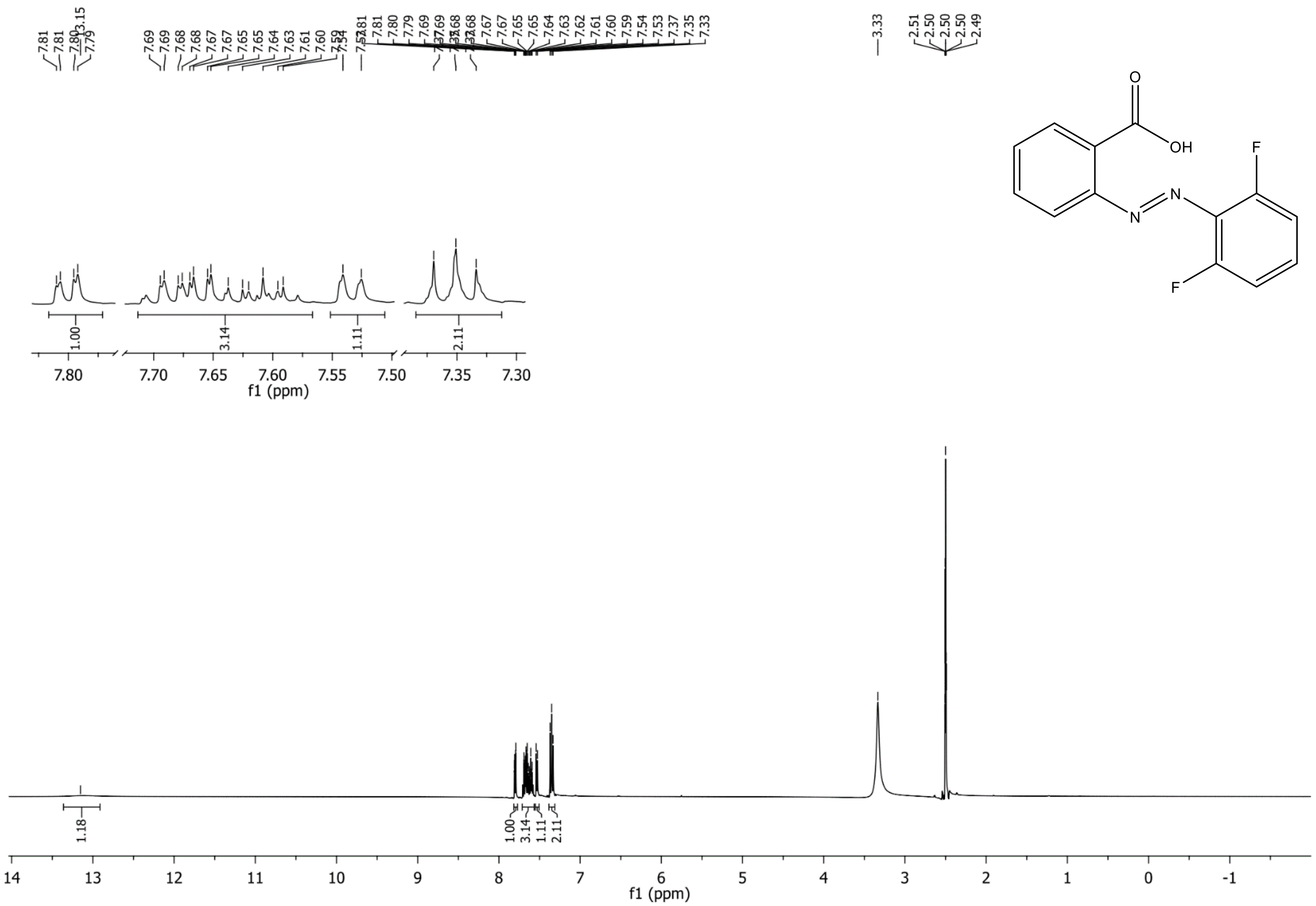

Figure S43. ^{1}H NMR spectra of *o*-**3d** ((*E*)-2-((2,6-difluorophenyl)diazenyl)benzoic acid).

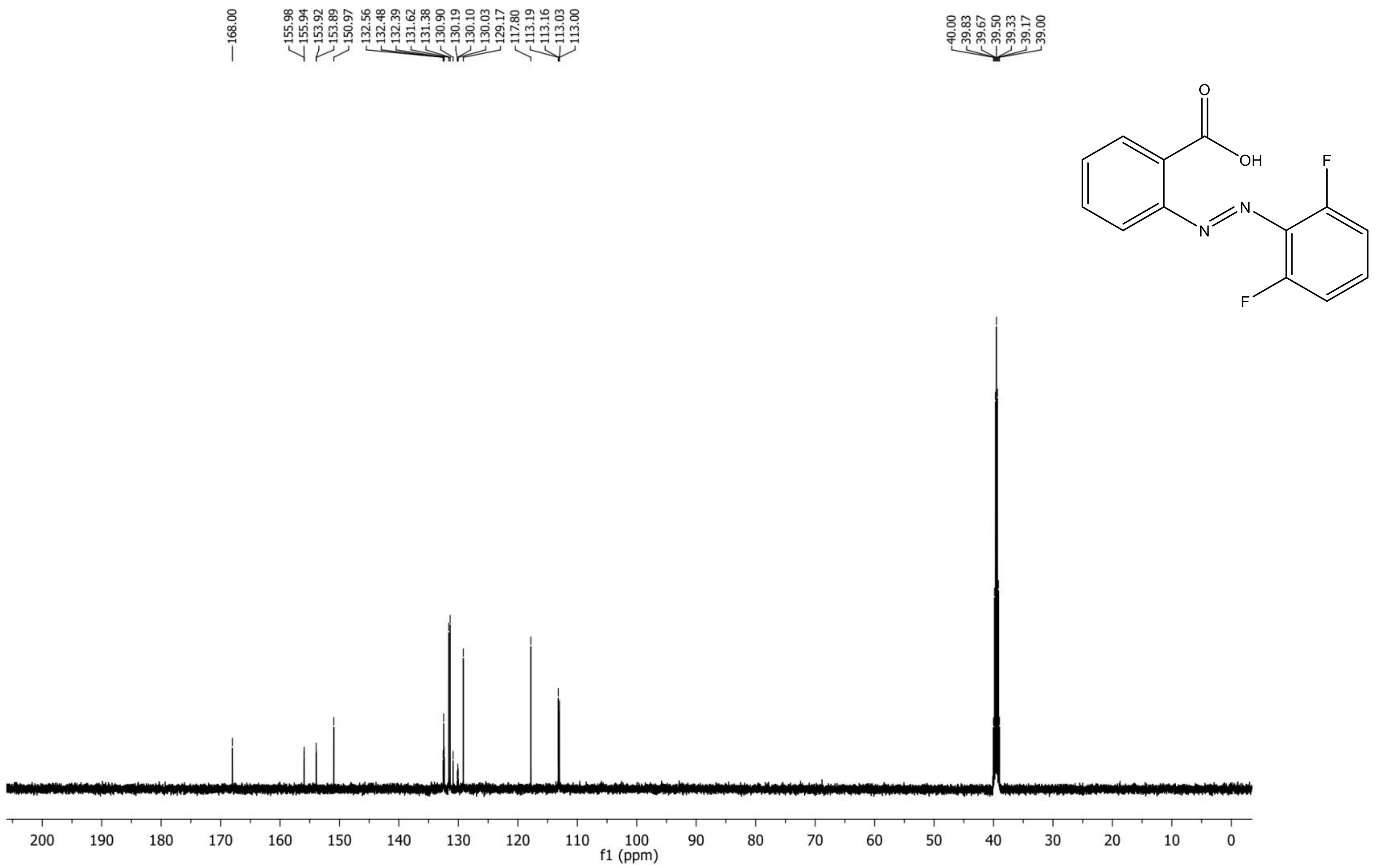

Figure S44. ^{13}C NMR spectra of *o*-**3d** ((*E*)-2-((2,6-difluorophenyl)diazenyl)benzoic acid).

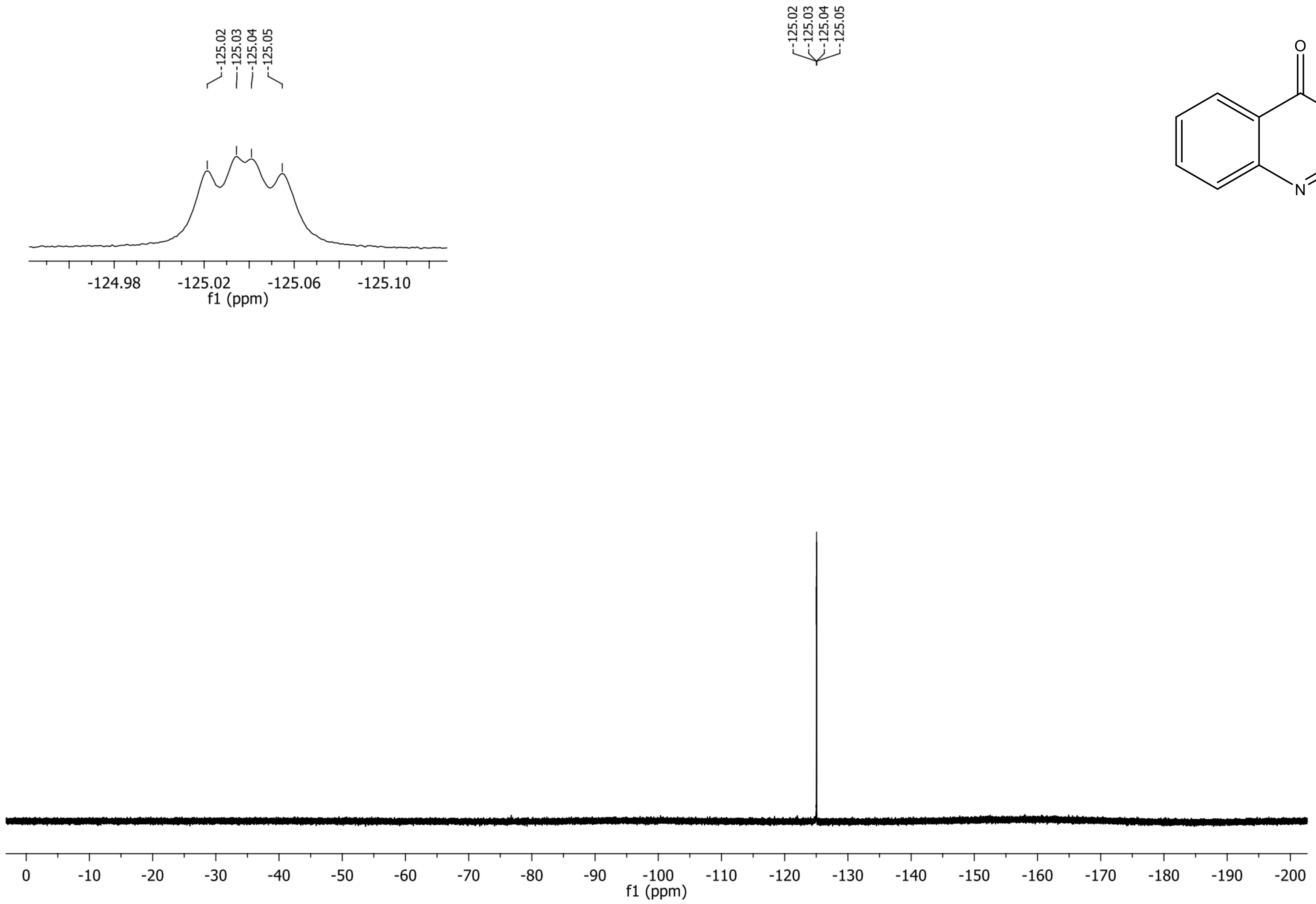

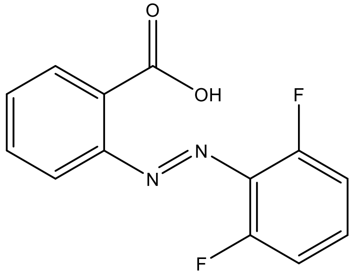

Figure S45. ^{19}F NMR spectra of *o*-**3d** ((*E*)-2-((2,6-difluorophenyl)diazenyl)benzoic acid).

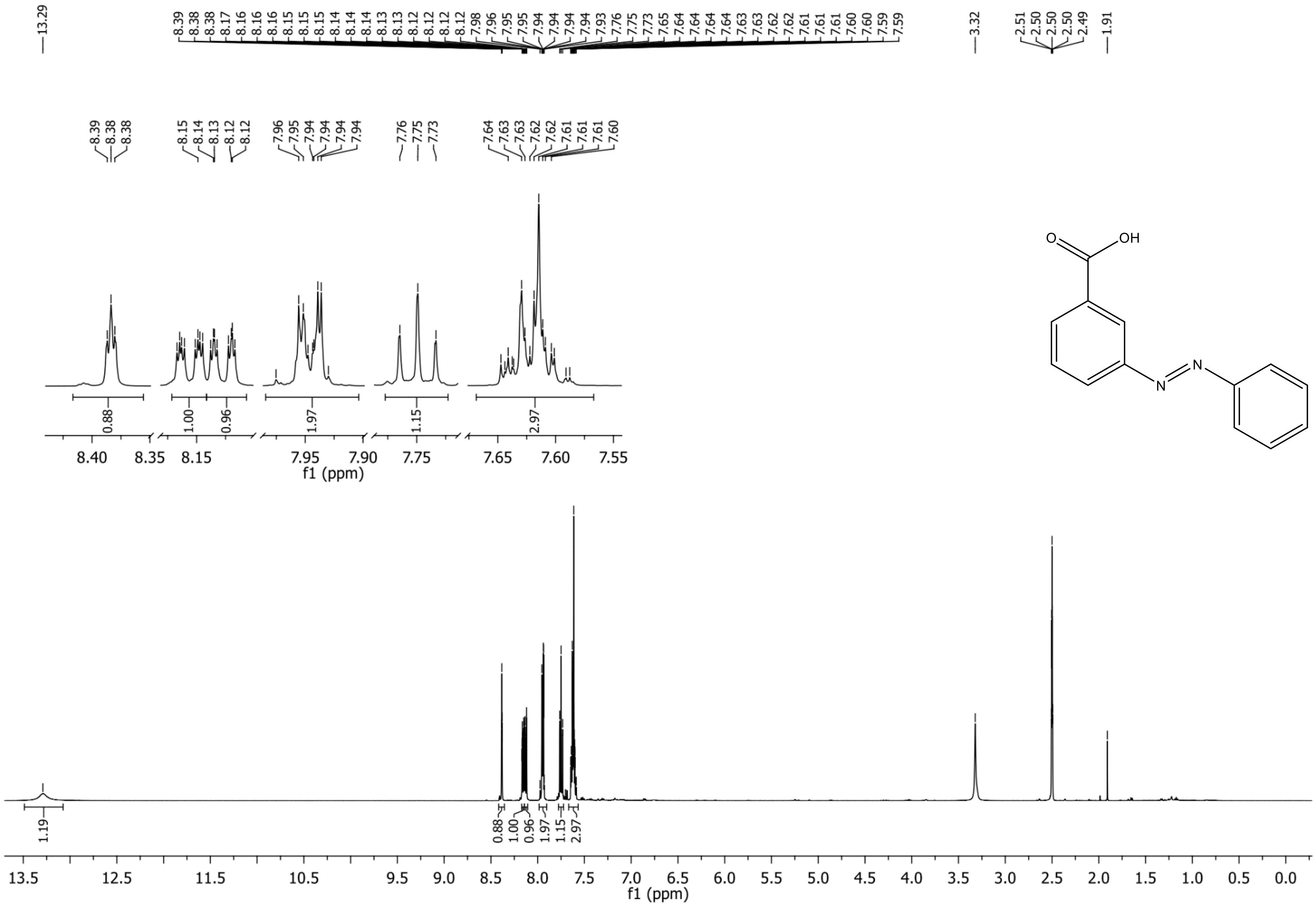

Figure S46. ^{1}H NMR spectra of *m*-**3a** ((*E*)-3-(phenyldiazenyl)benzoic acid).

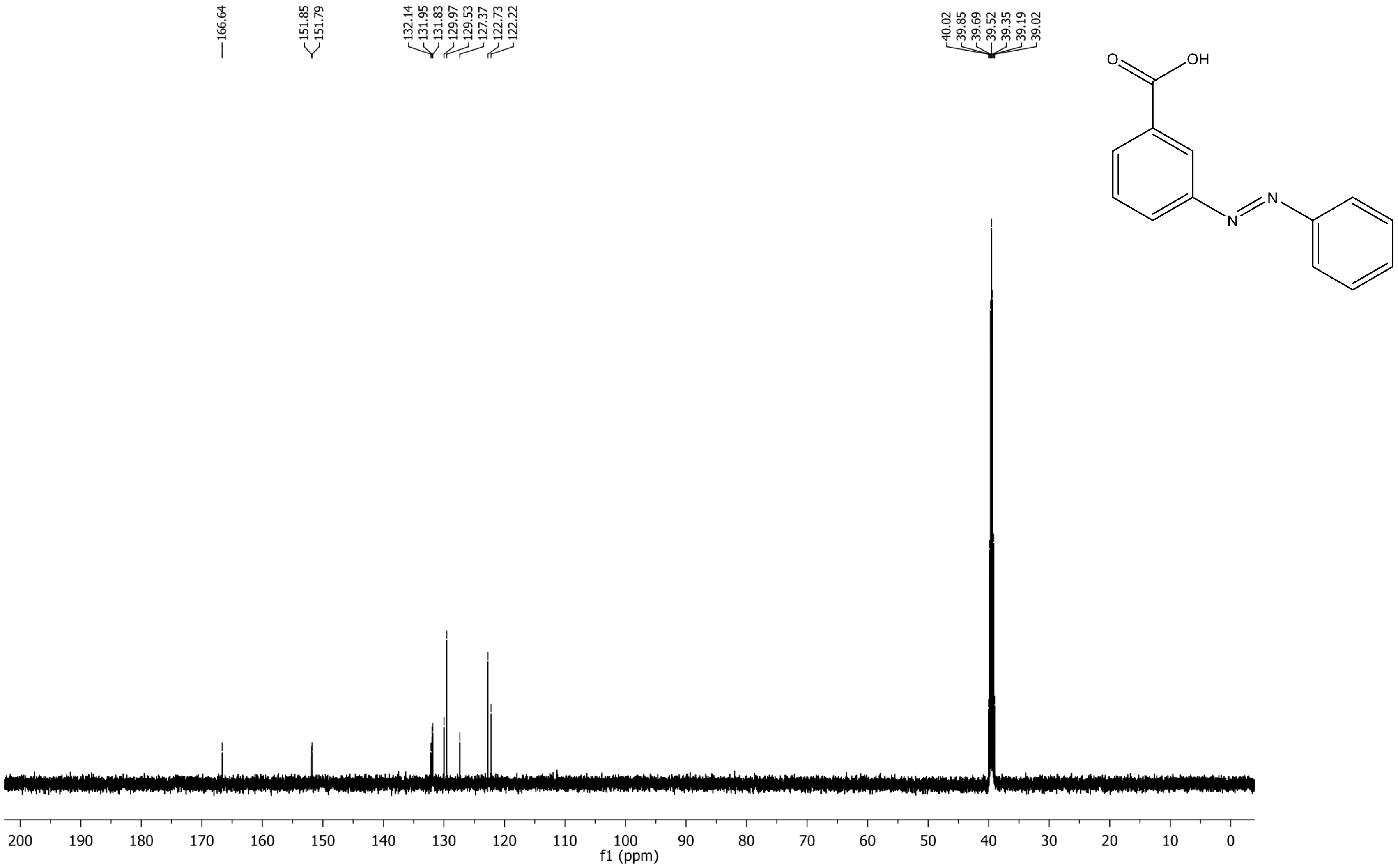

Figure S47. ^{13}C NMR spectra of *m*-**3a** ((*E*)-3-(phenyldiazenyl)benzoic acid).

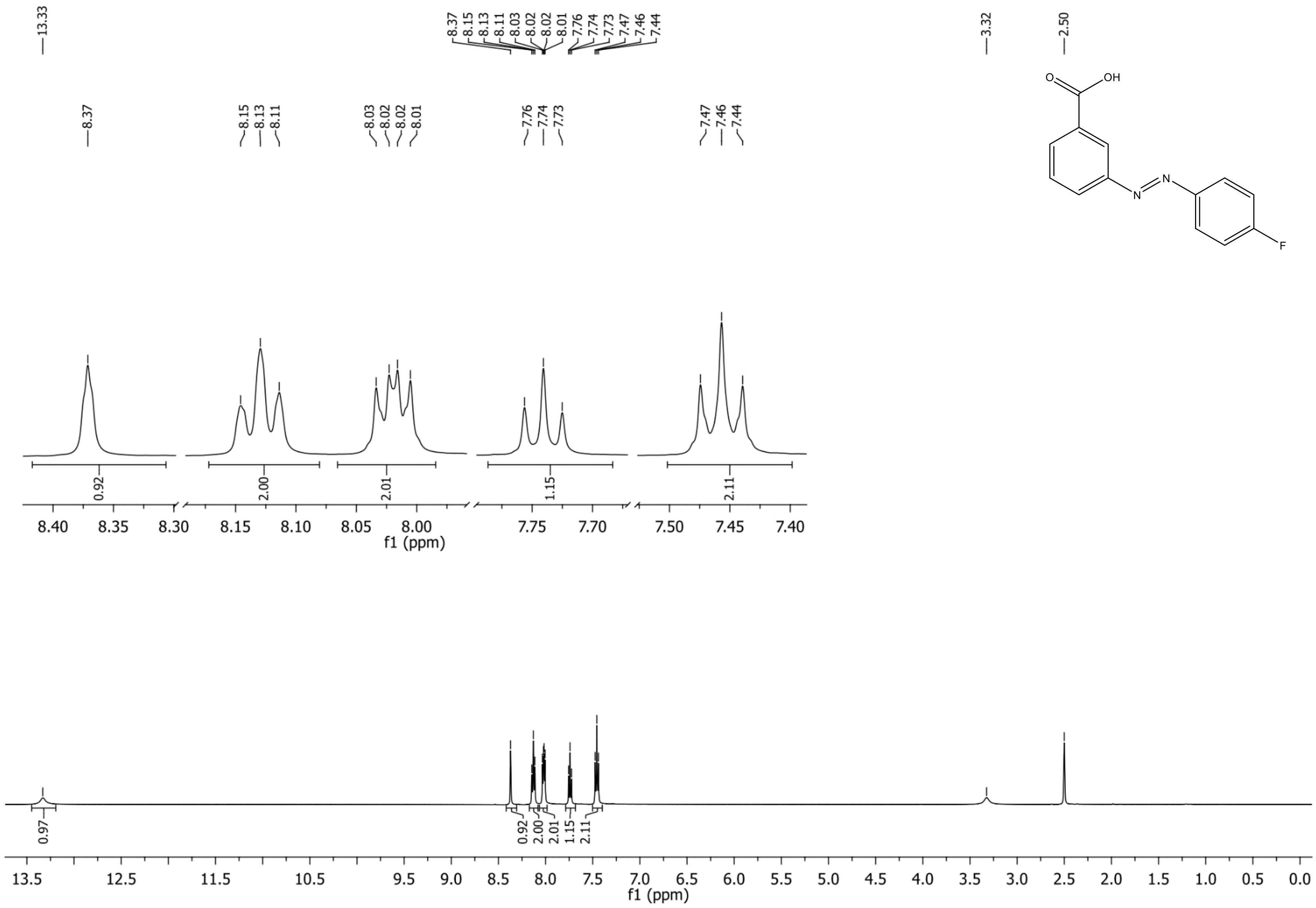

Figure S48. ^{1}H NMR spectra of *m*-**3b** ((*E*)-3-((4-fluorophenyl)diazenyl)benzoic acid).

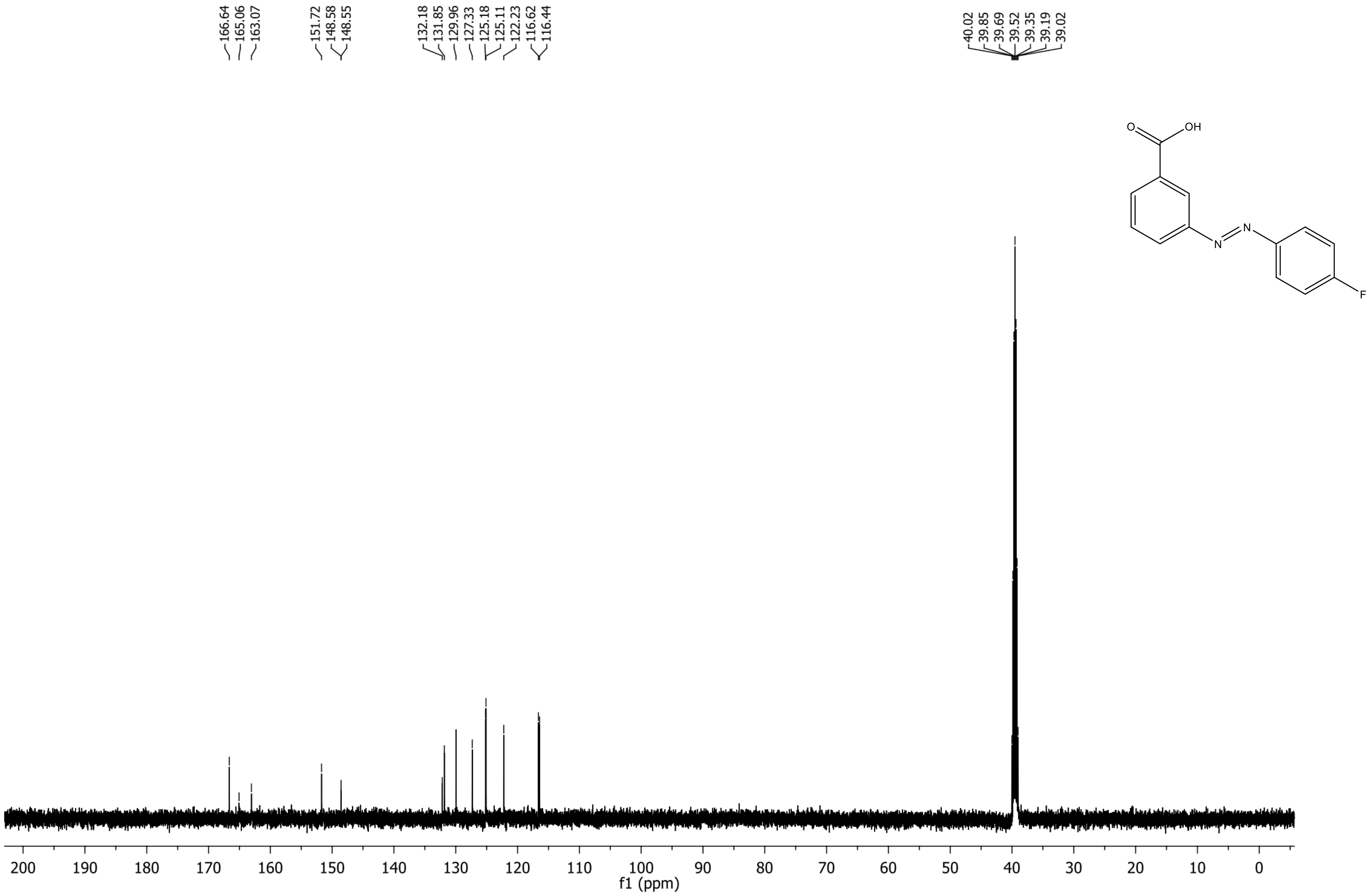

Figure S49. ^{13}C NMR spectra of *m*-**3b** ((*E*)-3-((4-fluorophenyl)diazenyl)benzoic acid).

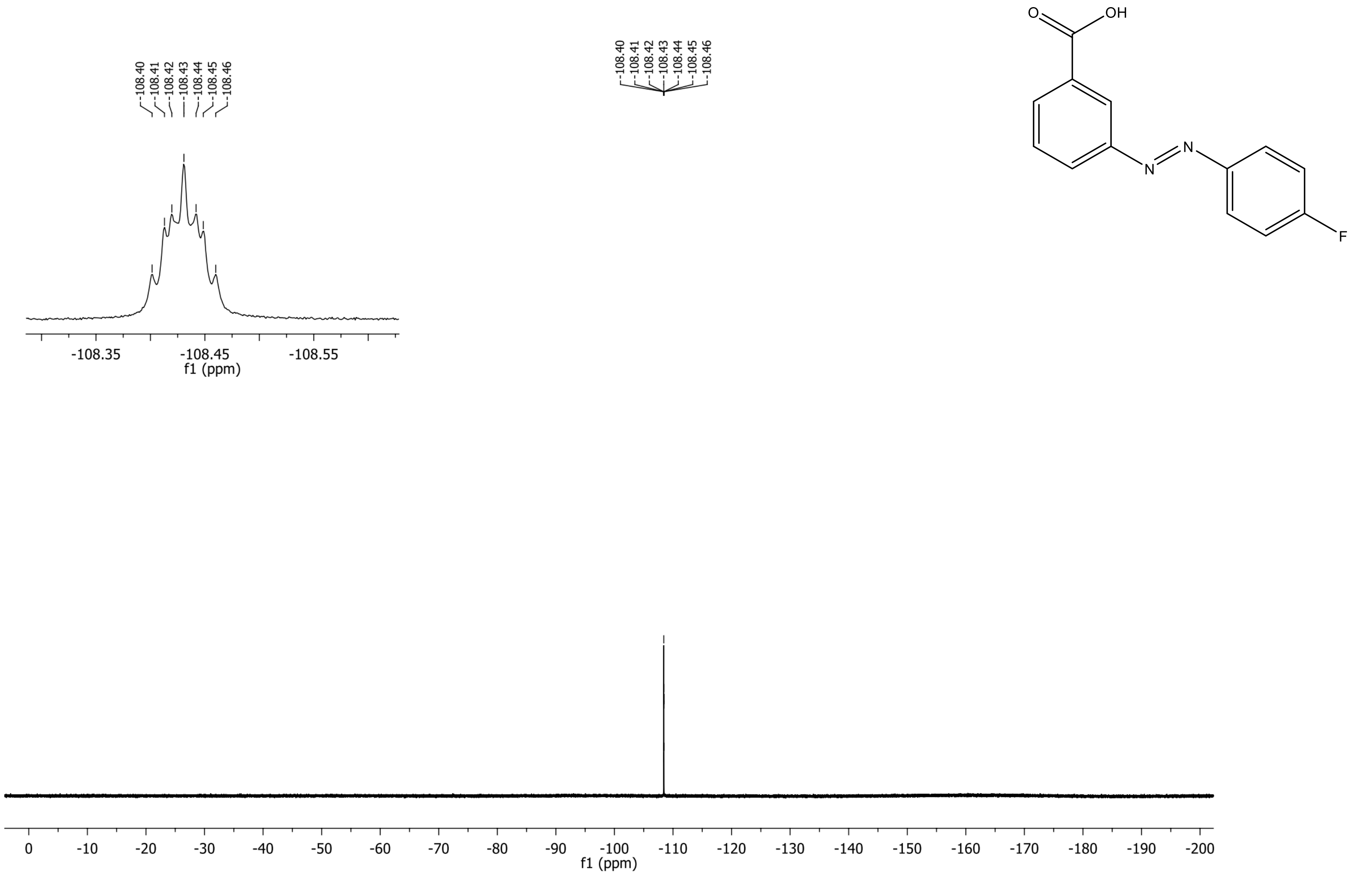

Figure S50. ^{19}F NMR spectra of *m*-**3b** ((*E*)-3-((4-fluorophenyl)diazenyl)benzoic acid).

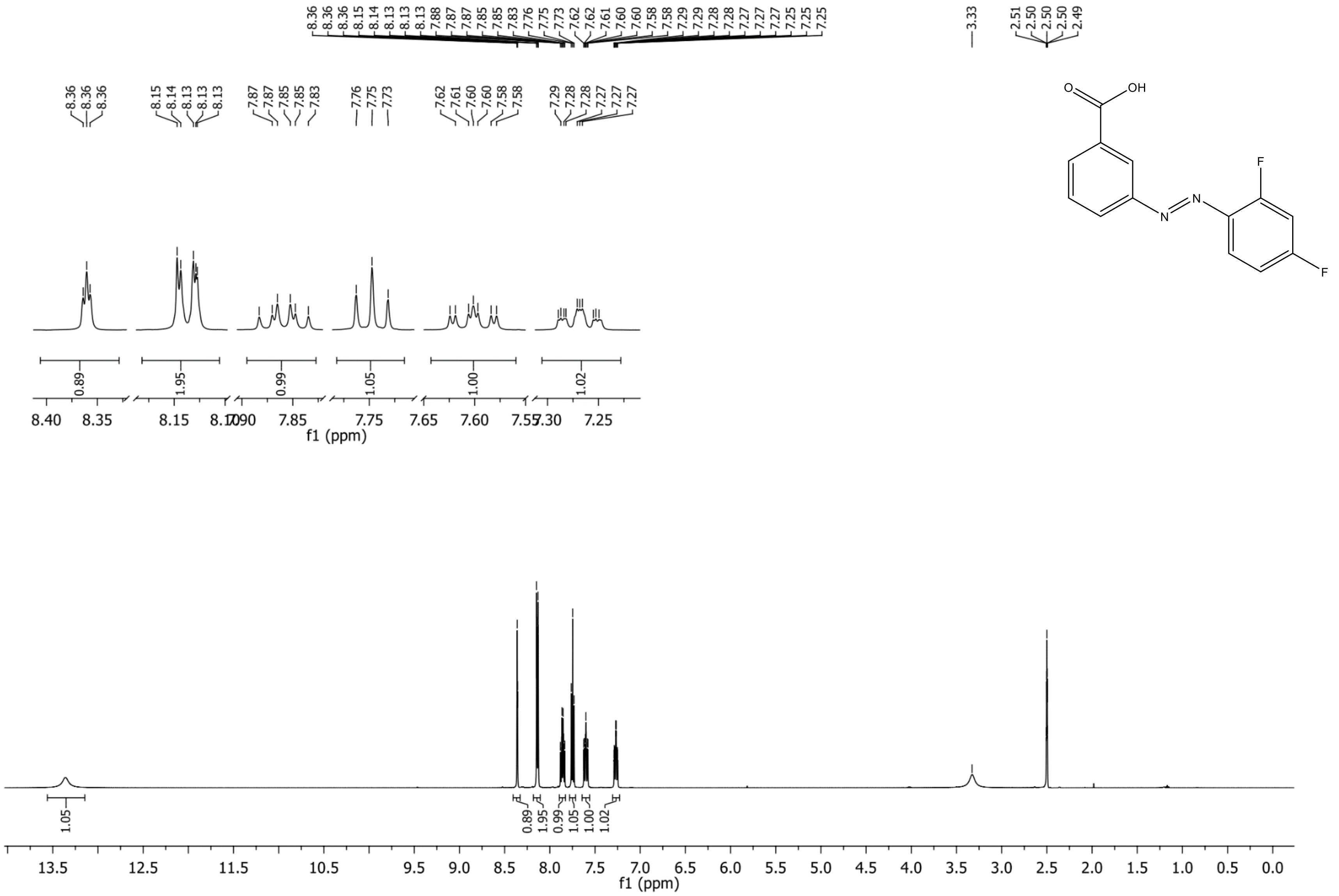

Figure S51. ^{1}H NMR spectra of *m*-**3c** ((*E*)-3-((2,4-difluorophenyl)diazenyl)benzoic acid)

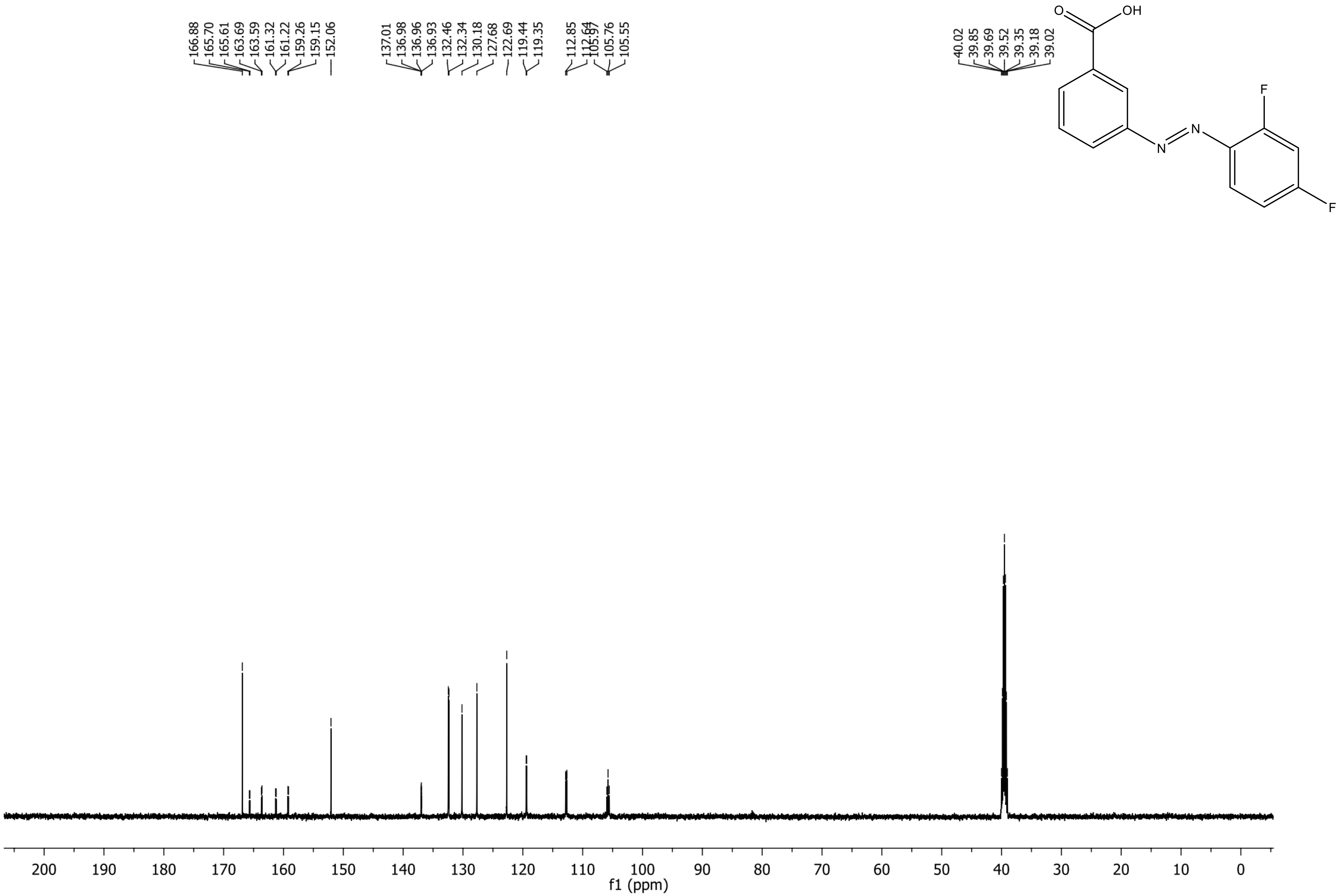

Figure S52. ^{13}C NMR spectra of *m*-**3c** ((*E*)-3-((2,4-difluorophenyl)diazenyl)benzoic acid).

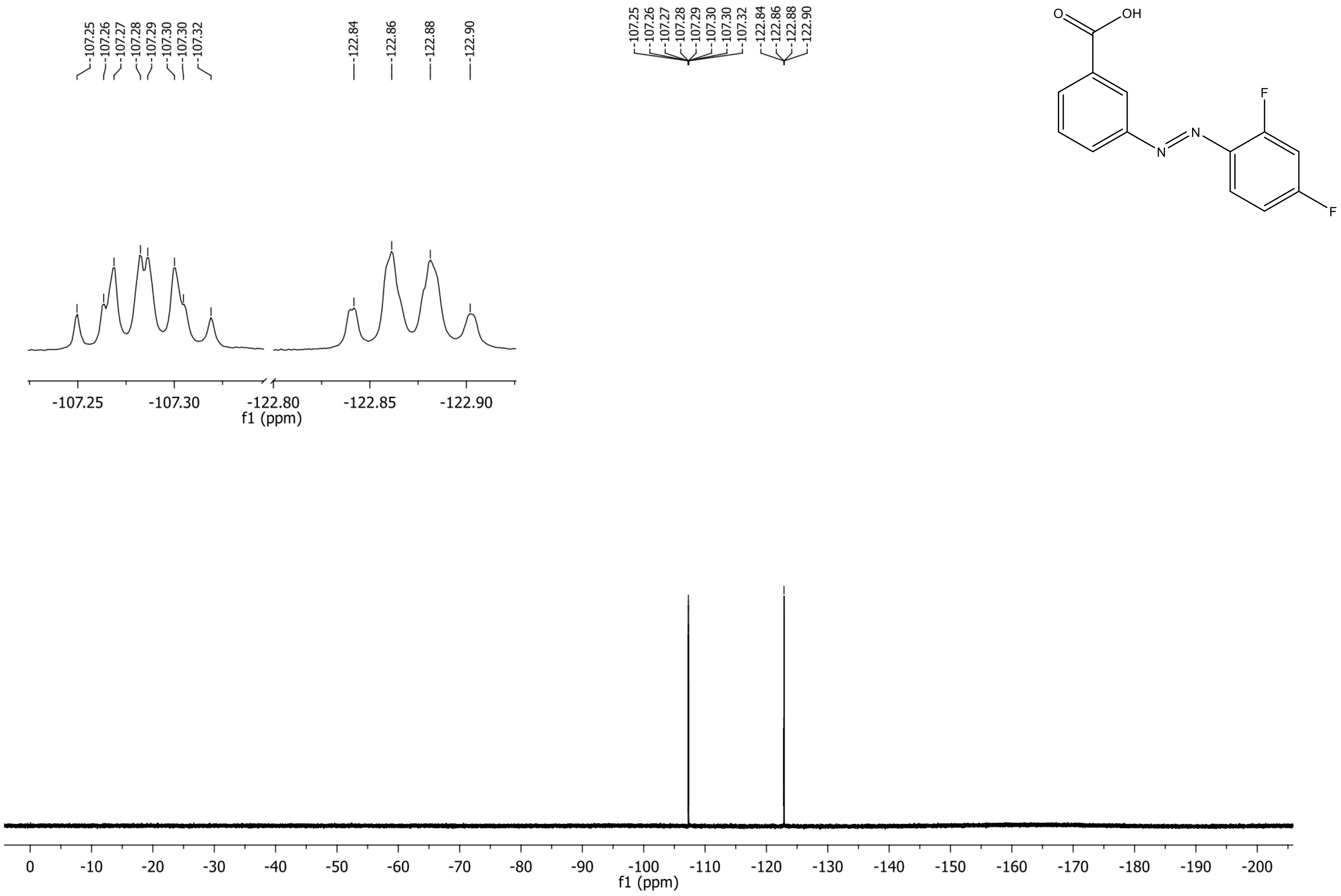

Figure S53. ^{19}F NMR spectra of *m*-**3c** ((*E*)-3-((2,4-difluorophenyl)diazenyl)benzoic acid).

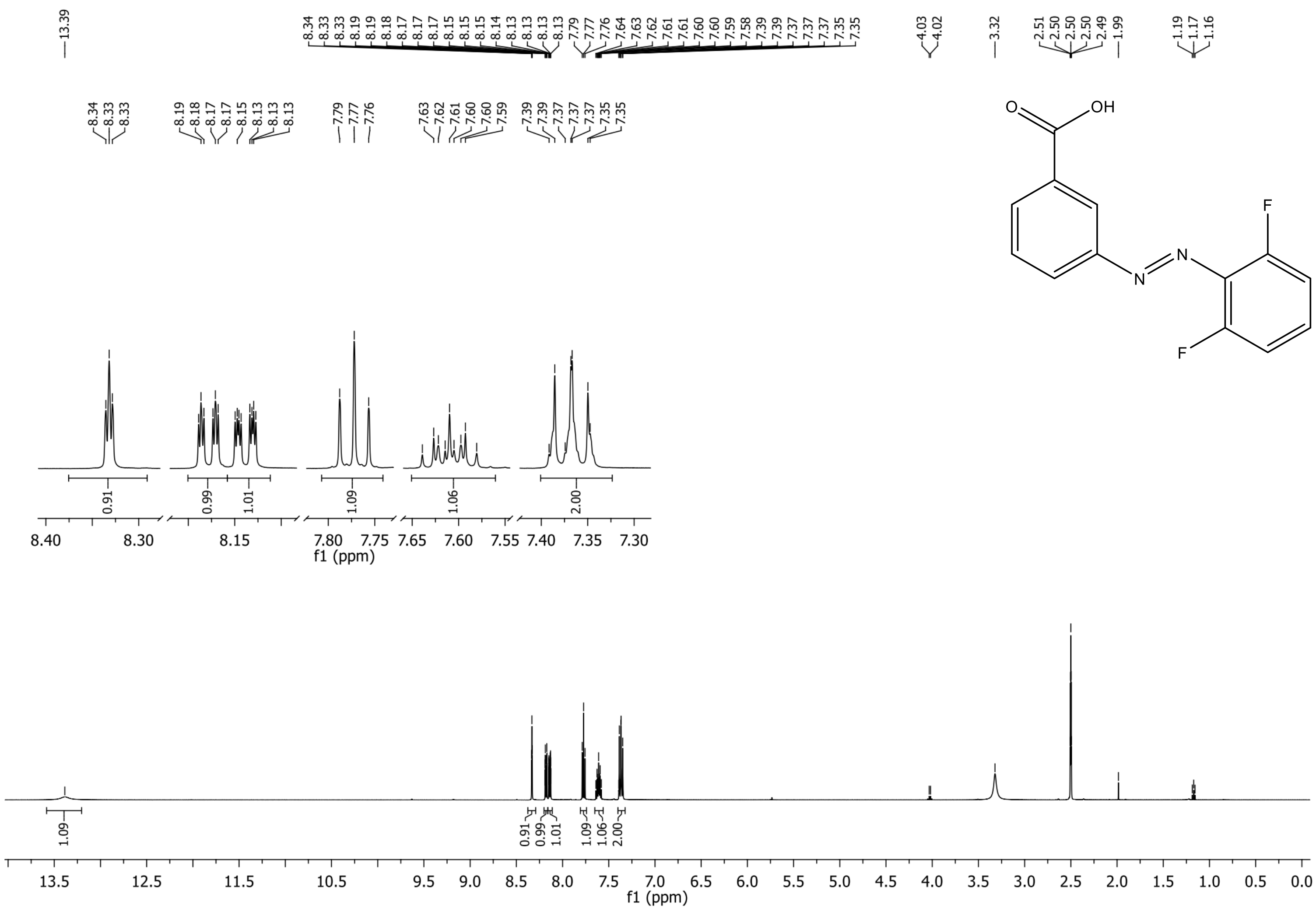

Figure S54. ^{1}H NMR spectra of *m*-**3d** ((*E*)-3-((2,6-difluorophenyl)diazenyl)benzoic acid).

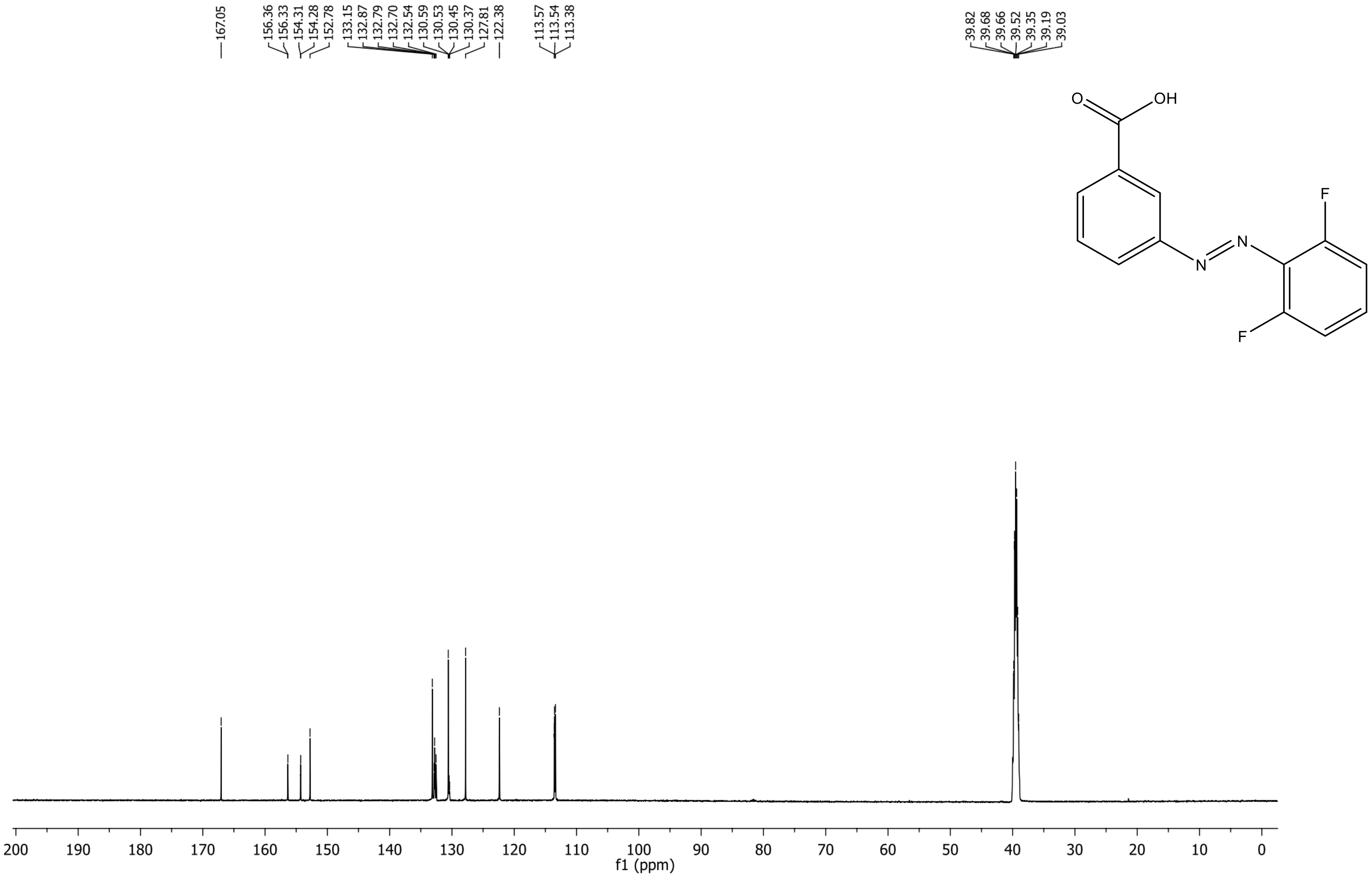

Figure S55. ^{13}C NMR spectra of *m*-**3d** ((*E*)-3-((2,6-difluorophenyl)diazenyl)benzoic acid).

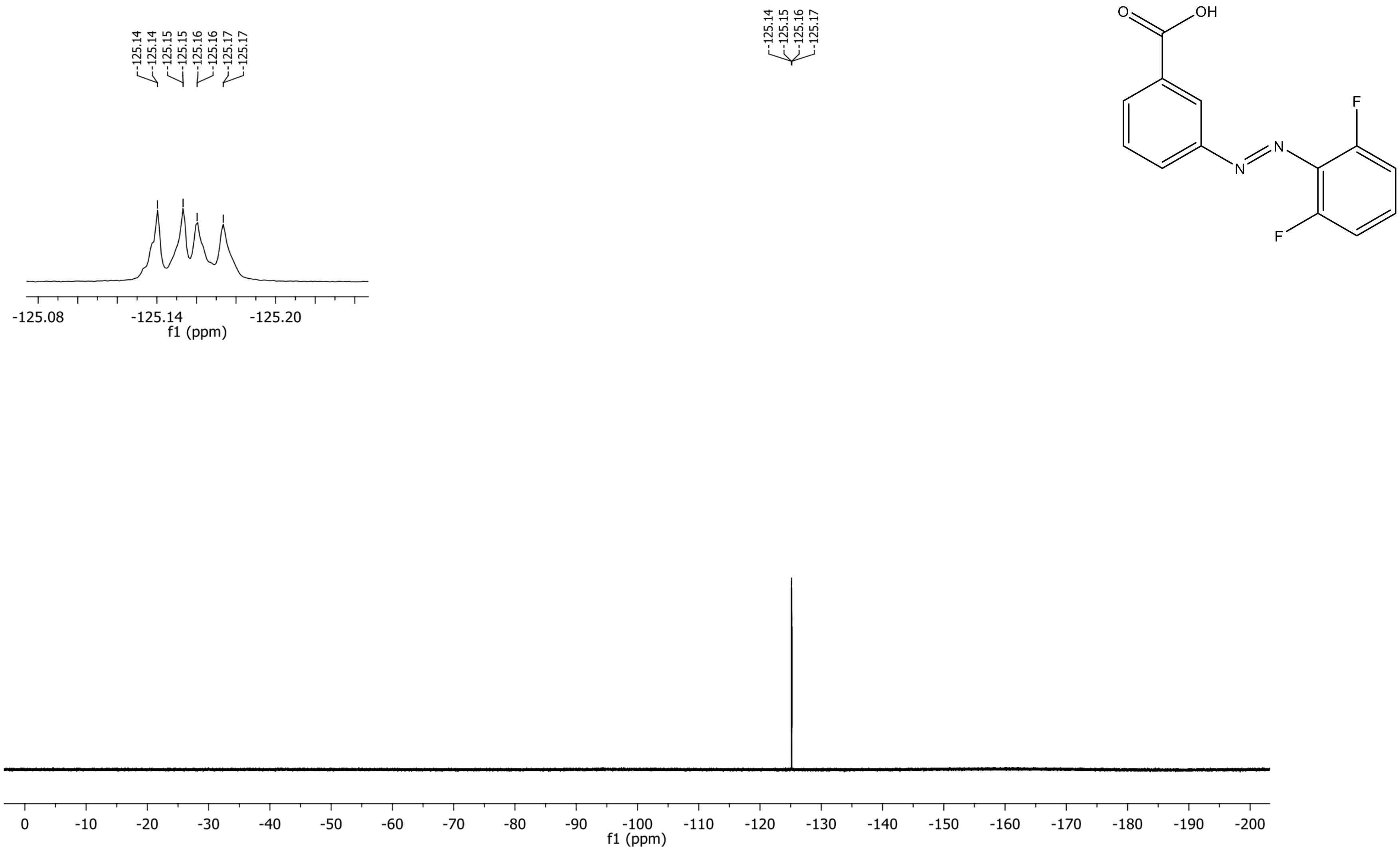

Figure S56. ^{19}F NMR spectra of *m*-**3d** ((*E*)-3-((2,6-difluorophenyl)diazenyl)benzoic acid).

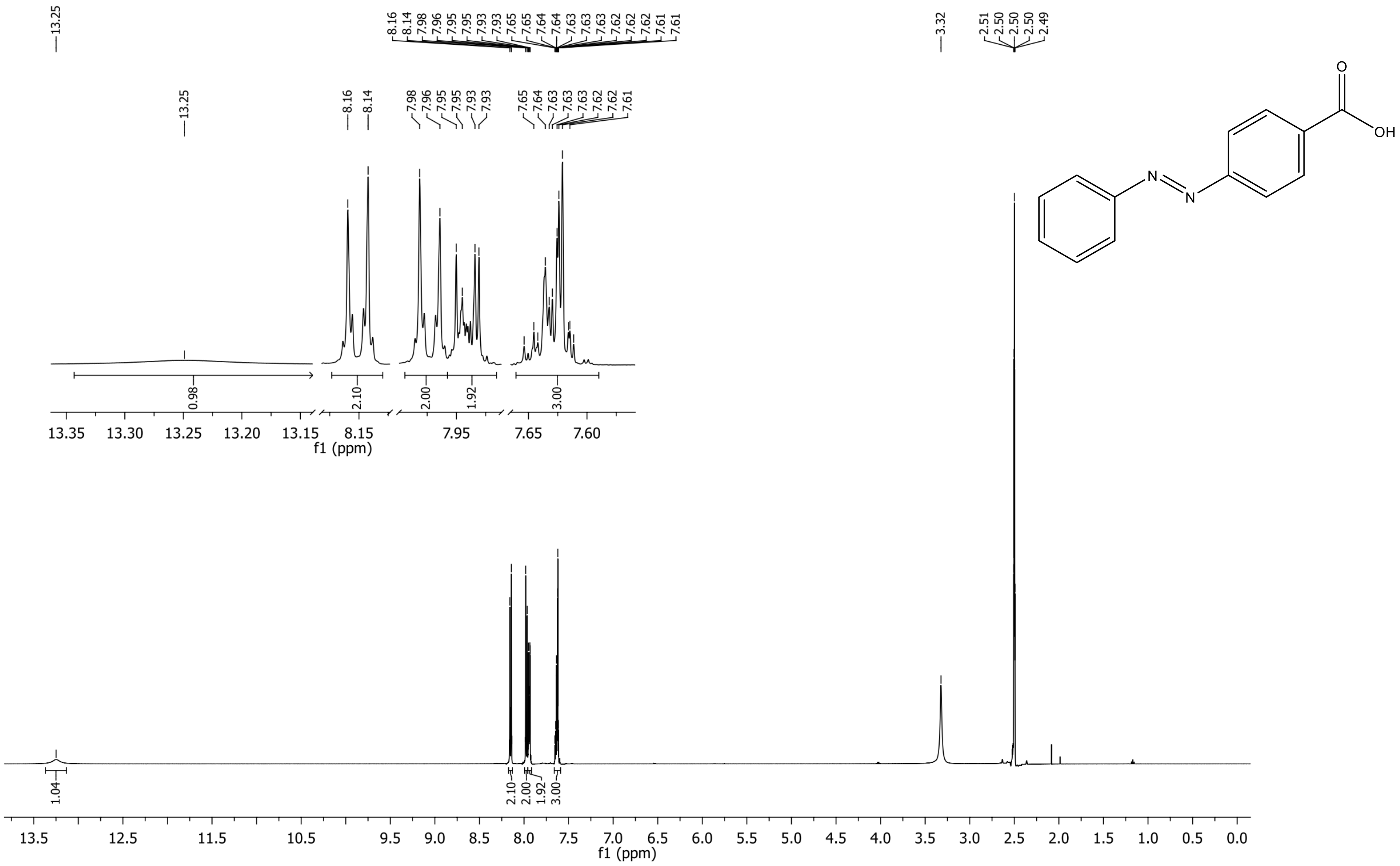

Figure S57. ^{1}H NMR spectra of *p*-**3a** ((*E*)-4-(phenyldiazenyl)benzoic acid).

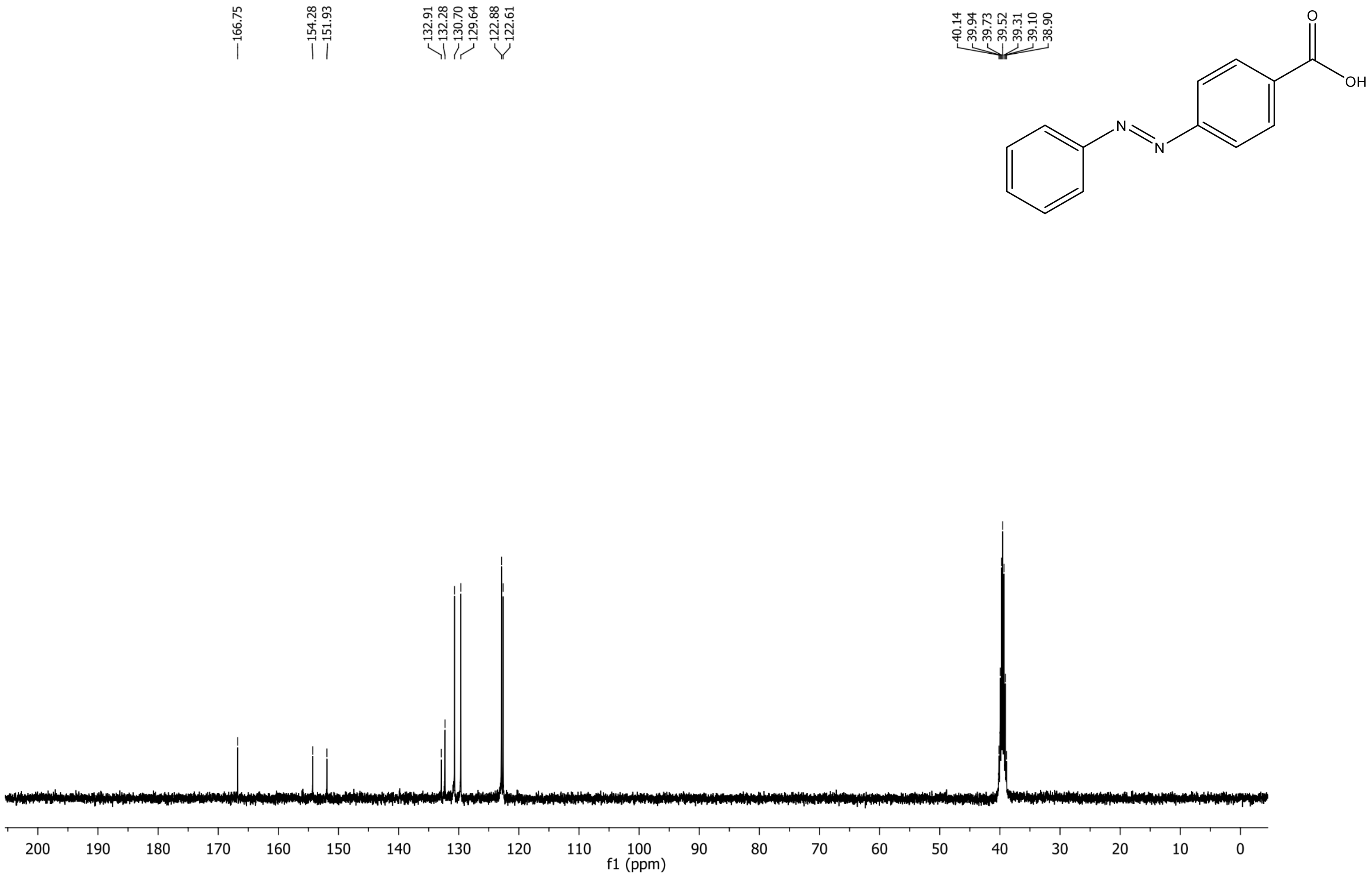

Figure S58. ^{13}C NMR spectra of *p*-**3a** ((*E*)-4-(phenyldiazenyl)benzoic acid).

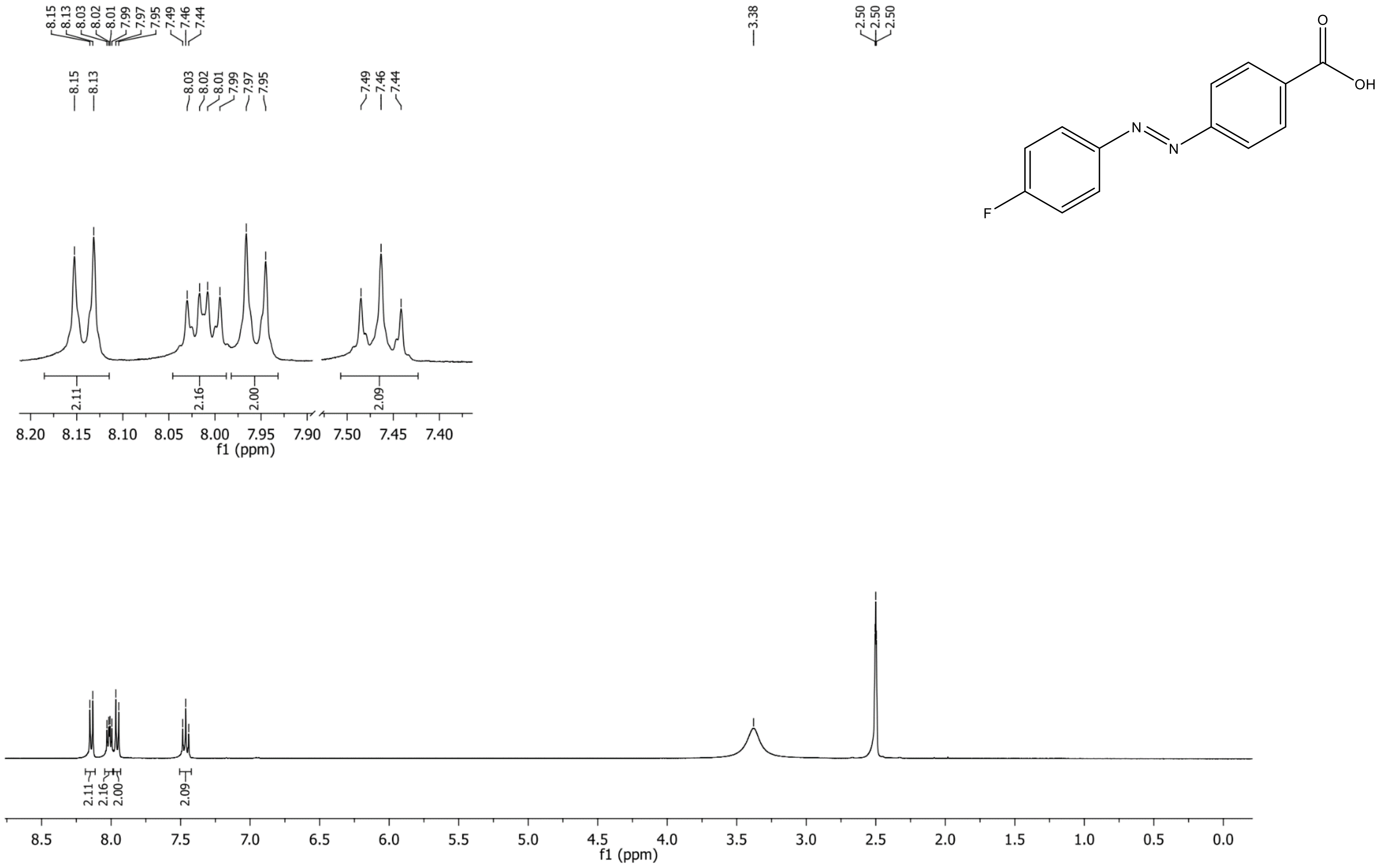

Figure S59. ^{1}H NMR spectra of *p*-**3b** ((*E*)-4-((4-fluorophenyl)diazenyl)benzoic acid).

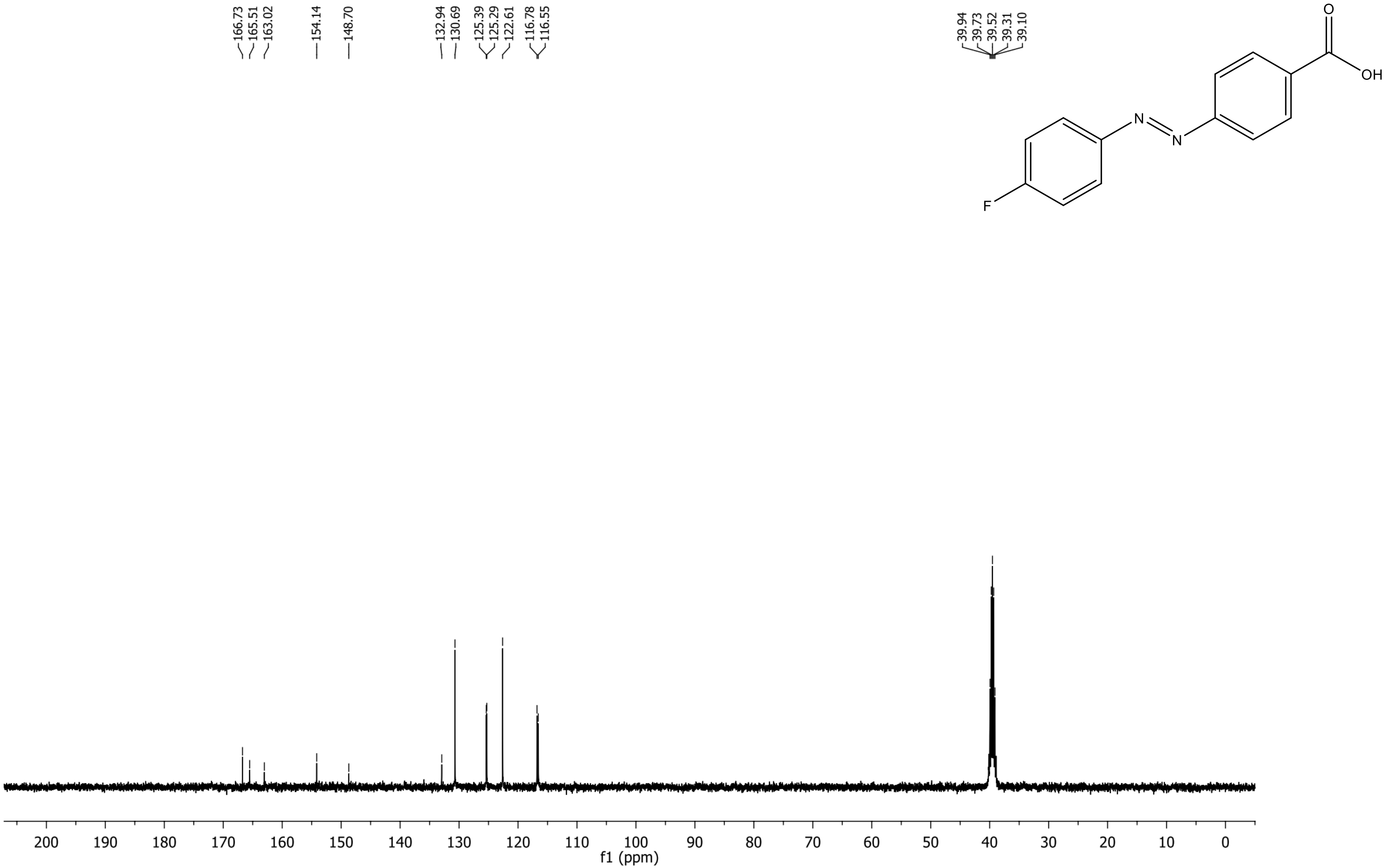

Figure S60. ^{13}C NMR spectra of *p*-**3b** ((*E*)-4-((4-fluorophenyl)diazenyl)benzoic acid).

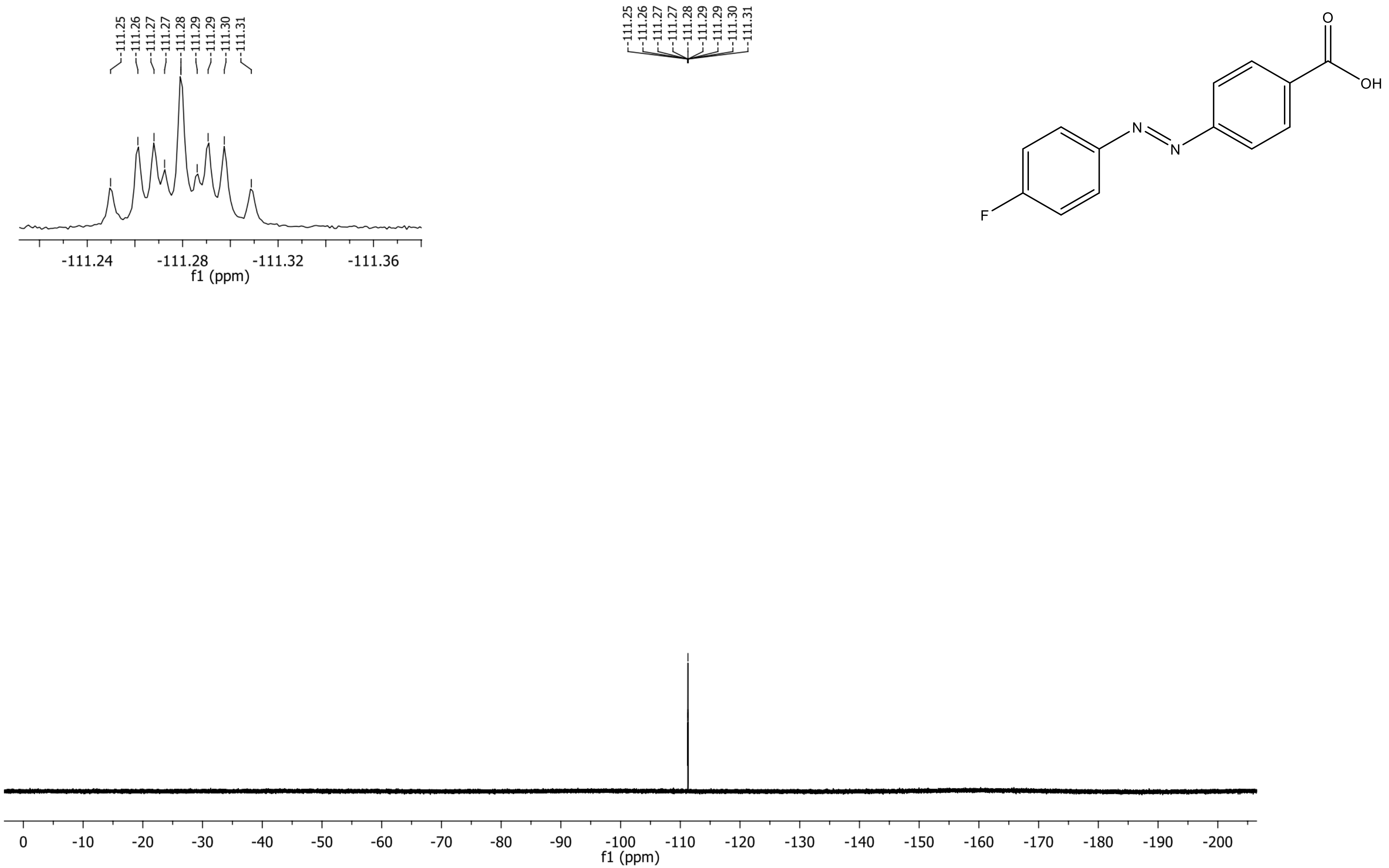

Figure S61. ^{19}F NMR spectra of *p*-**3b** ((*E*)-4-((4-fluorophenyl)diazenyl)benzoic acid).

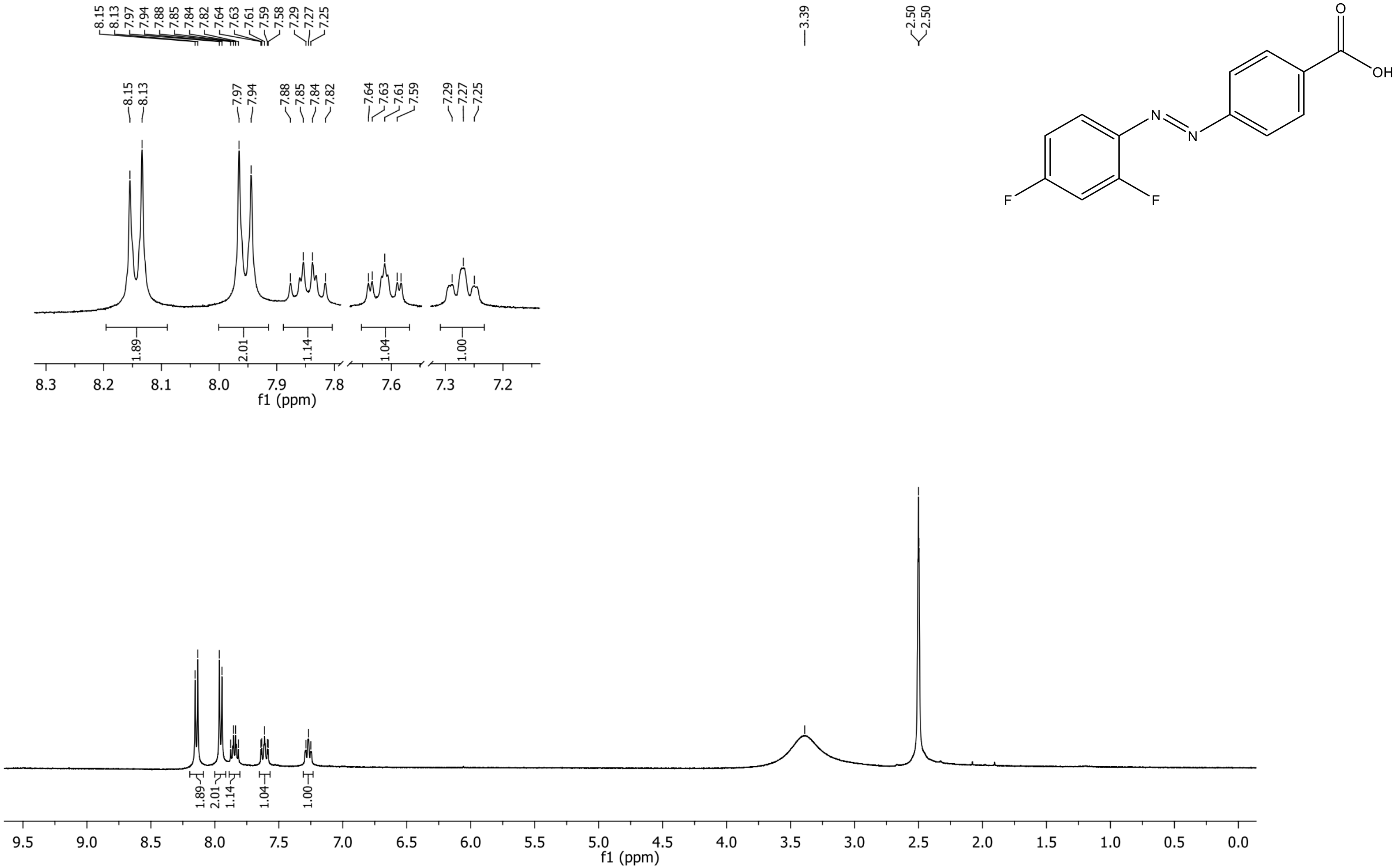

Figure S62. ^{1}H NMR spectra of *p*-**3c** ((*E*)-4-((2,4-difluorophenyl)diazenyl)benzoic acid).

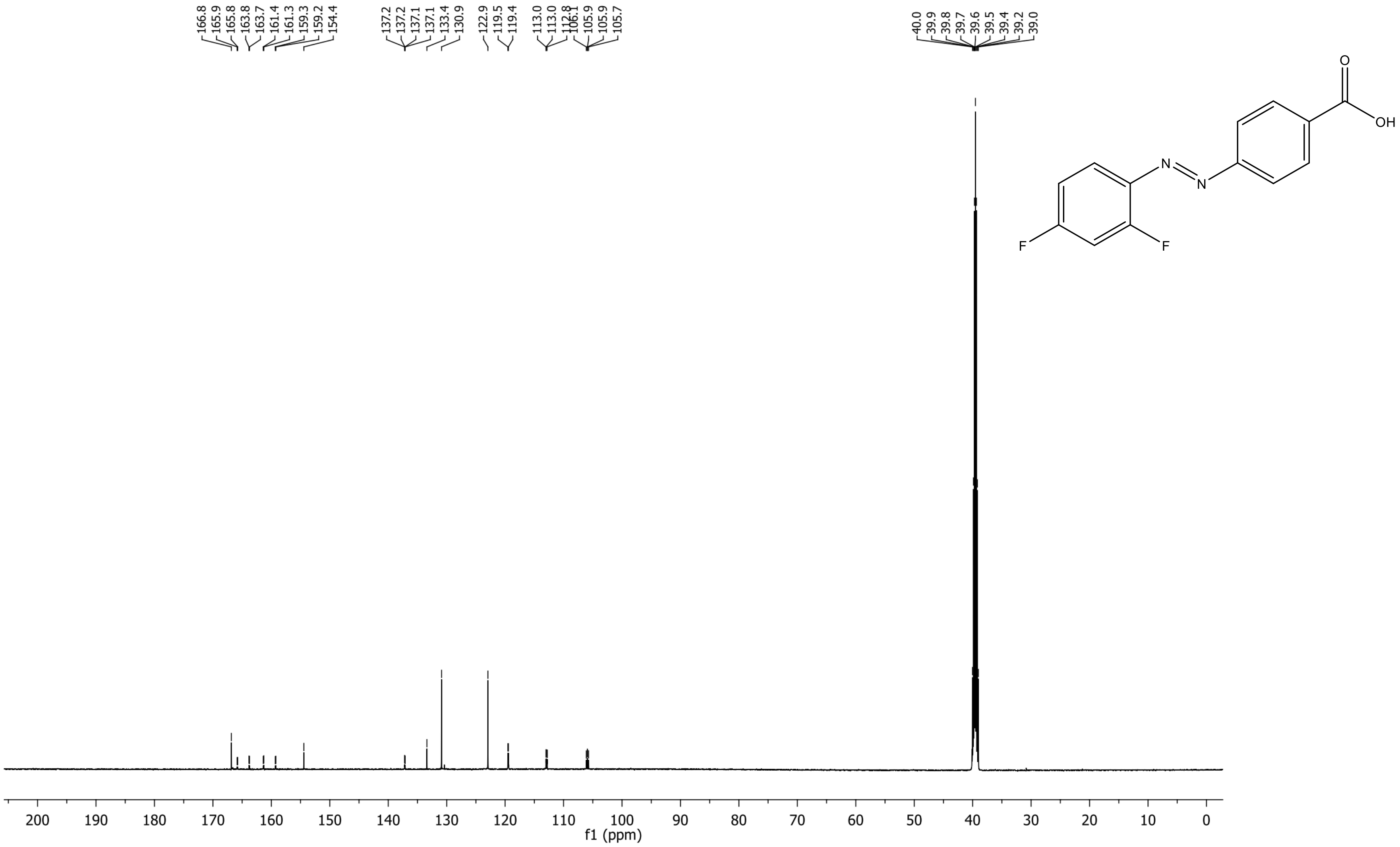

Figure S63. ^{13}C NMR spectra of *p*-**3c** ((*E*)-4-((2,4-difluorophenyl)diazenyl)benzoic acid).

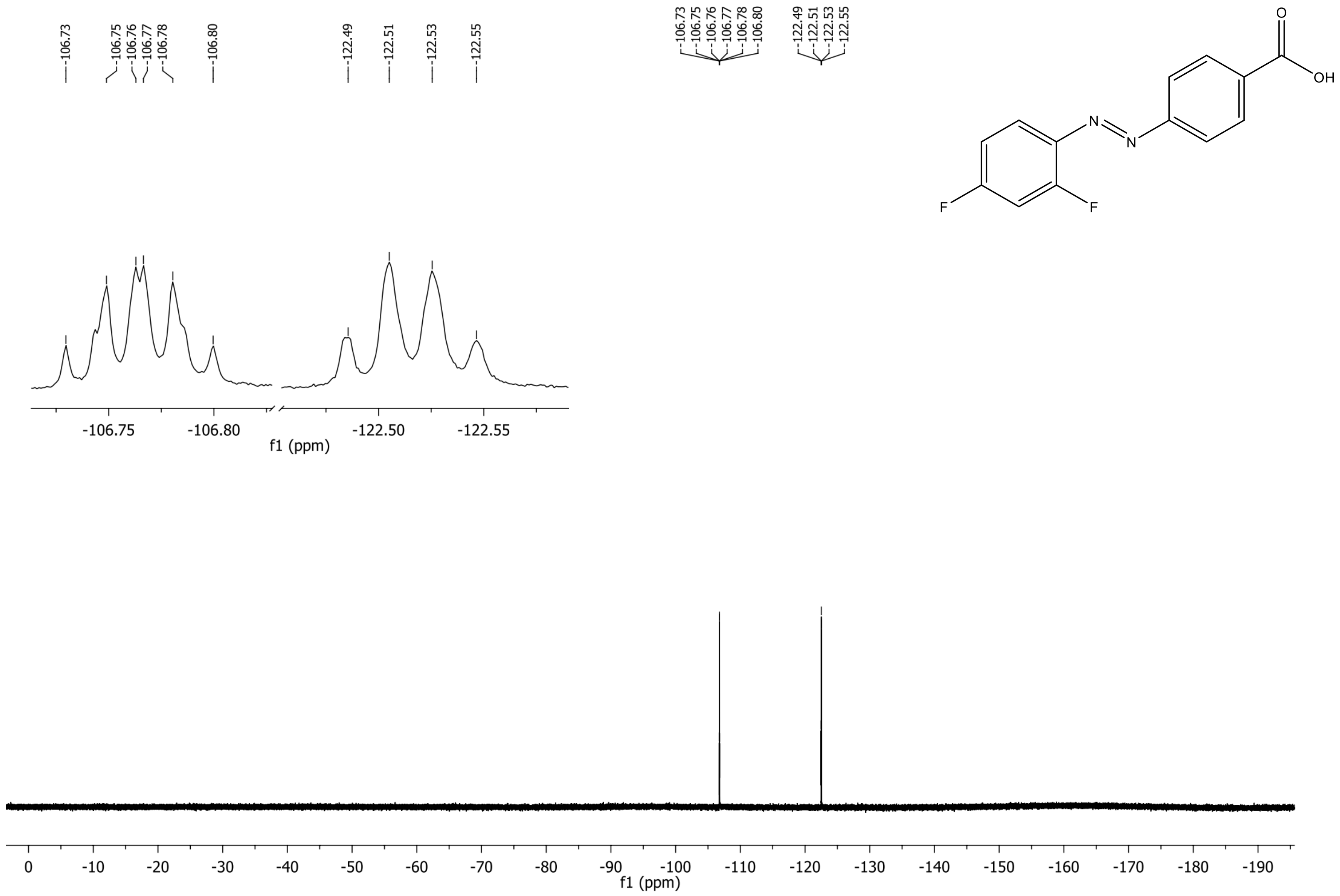

Figure S64. ^{19}F NMR spectra of *p*-**3c** ((*E*)-4-((2,4-difluorophenyl)diazenyl)benzoic acid).

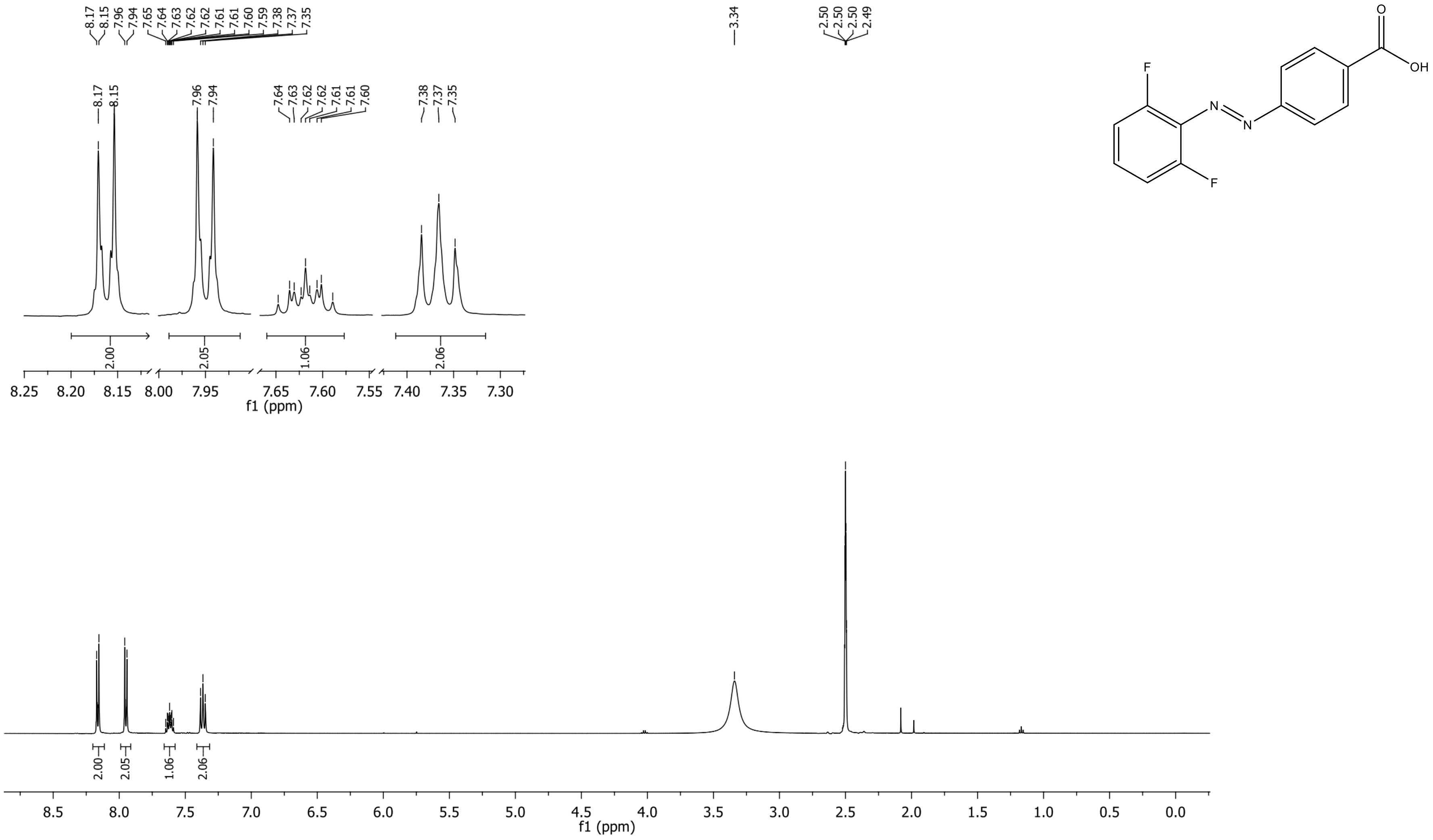

Figure S65. ^{1}H NMR spectra of *p*-**3d** ((*E*)-4-((2,6-difluorophenyl)diazenyl)benzoic acid).

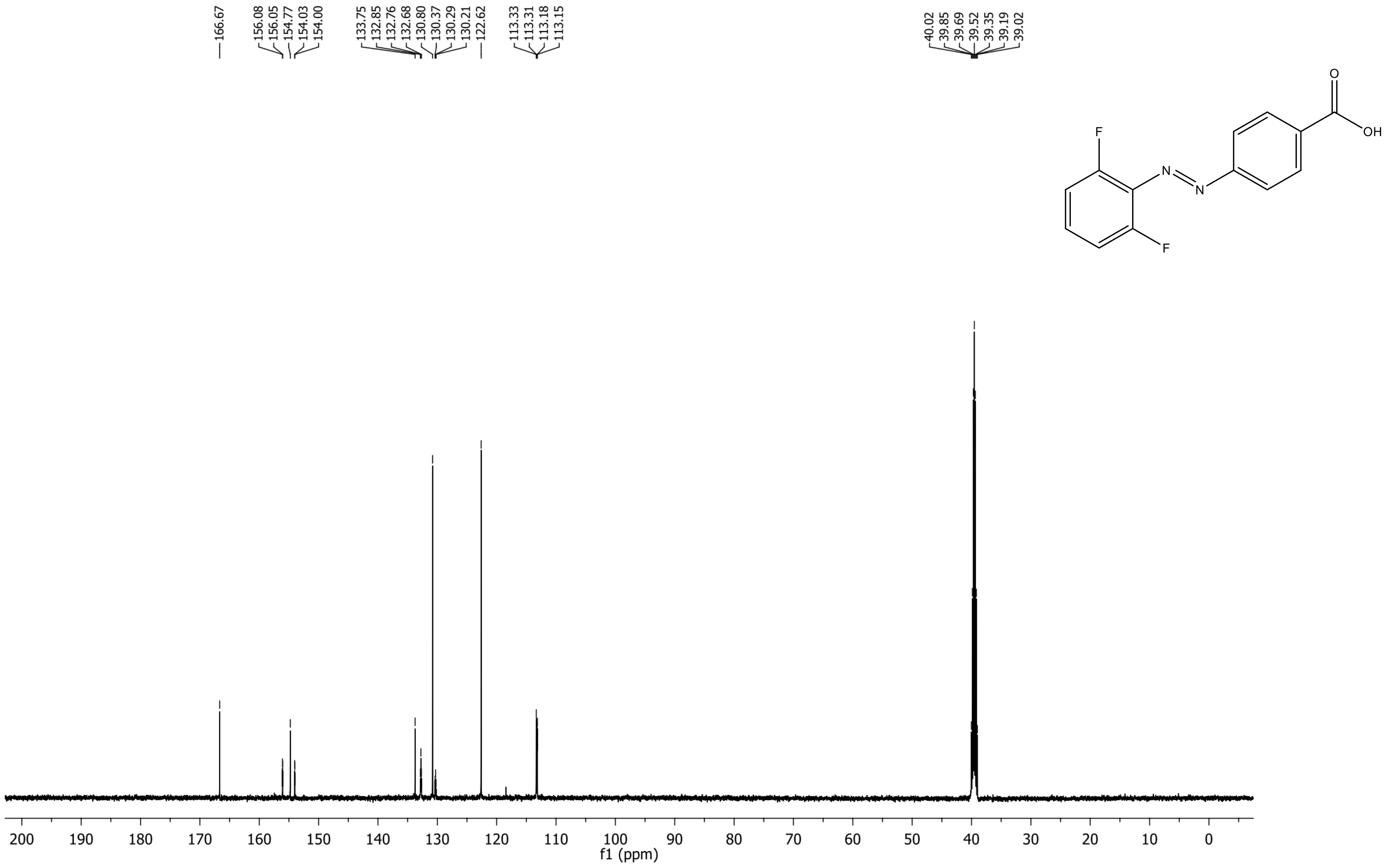

Figure S66. ^{13}C NMR spectra of *p*-**3d** ((*E*)-4-((2,6-difluorophenyl)diazenyl)benzoic acid).

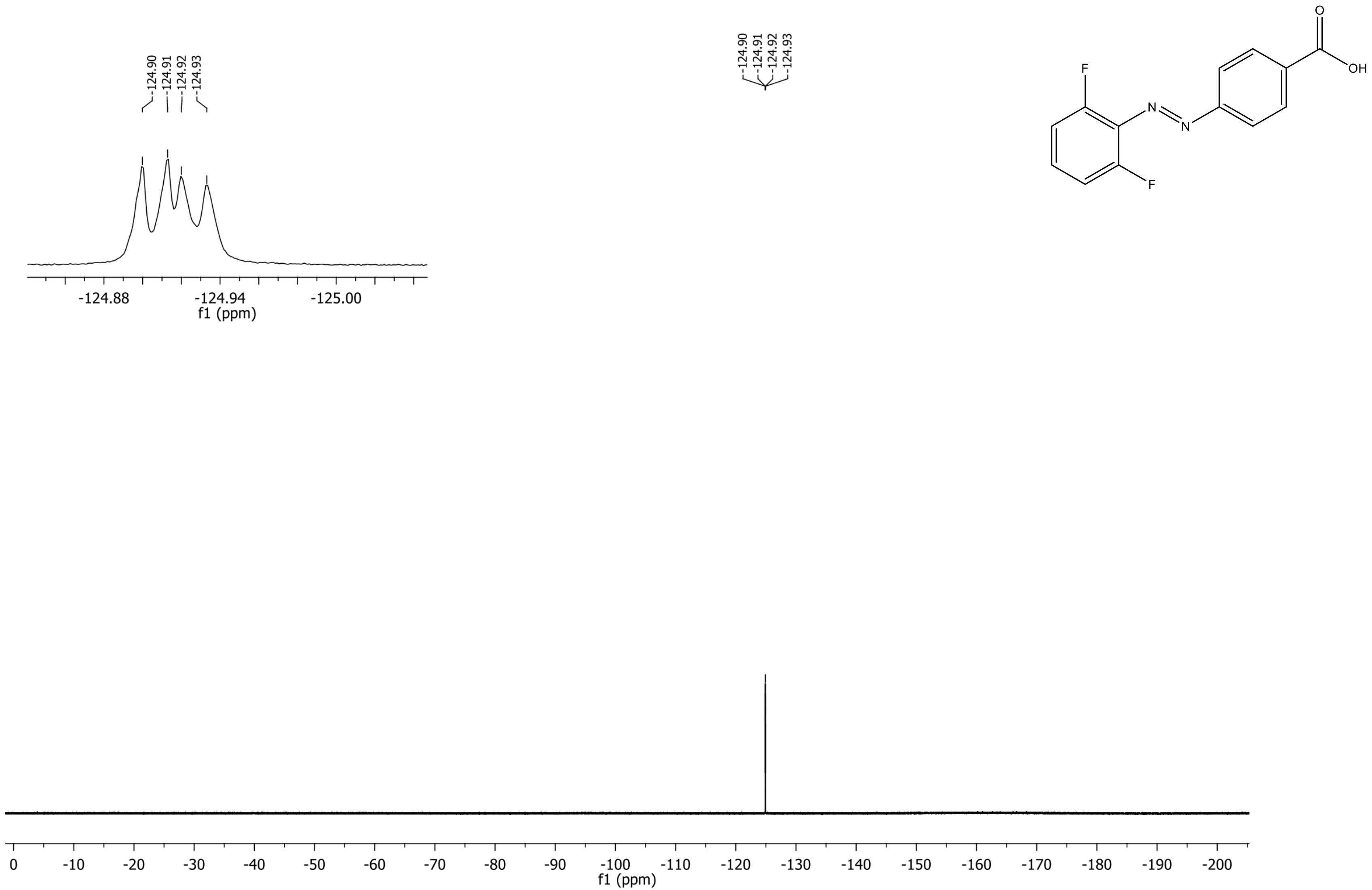

Figure S67. ^{19}F NMR spectra of *p*-**3d** ((*E*)-4-((2,6-difluorophenyl)diazenyl)benzoic acid).

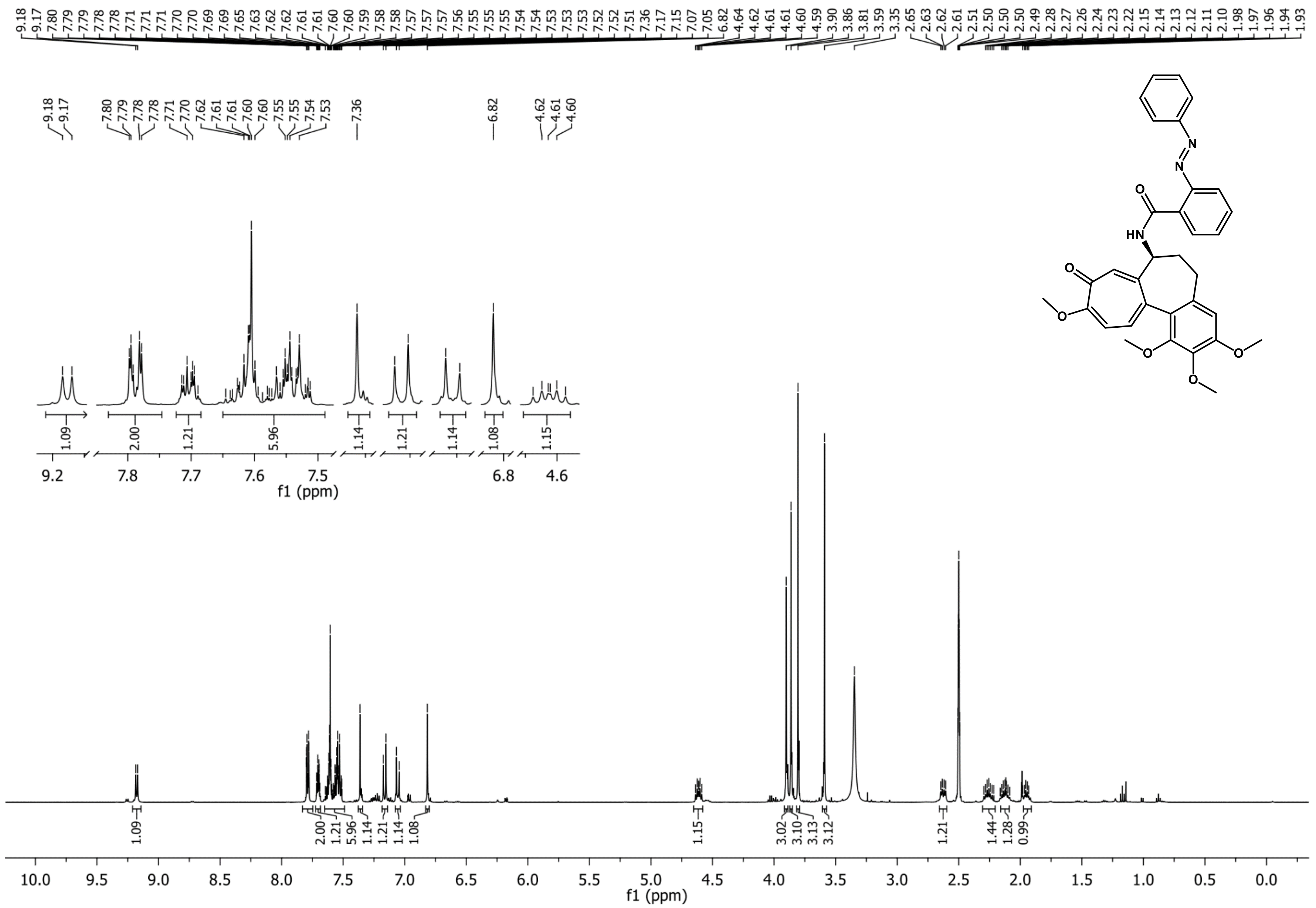

Figure S68. ^{1}H NMR spectra of *o*-**AzoCol** ((S,E)-2-(phenyldiazenyl)-N-(1,2,3,10-tetramethoxy-9-oxo-5,6,7,9-tetrahydrobenzo[a]heptalen-7-yl)benzamide).

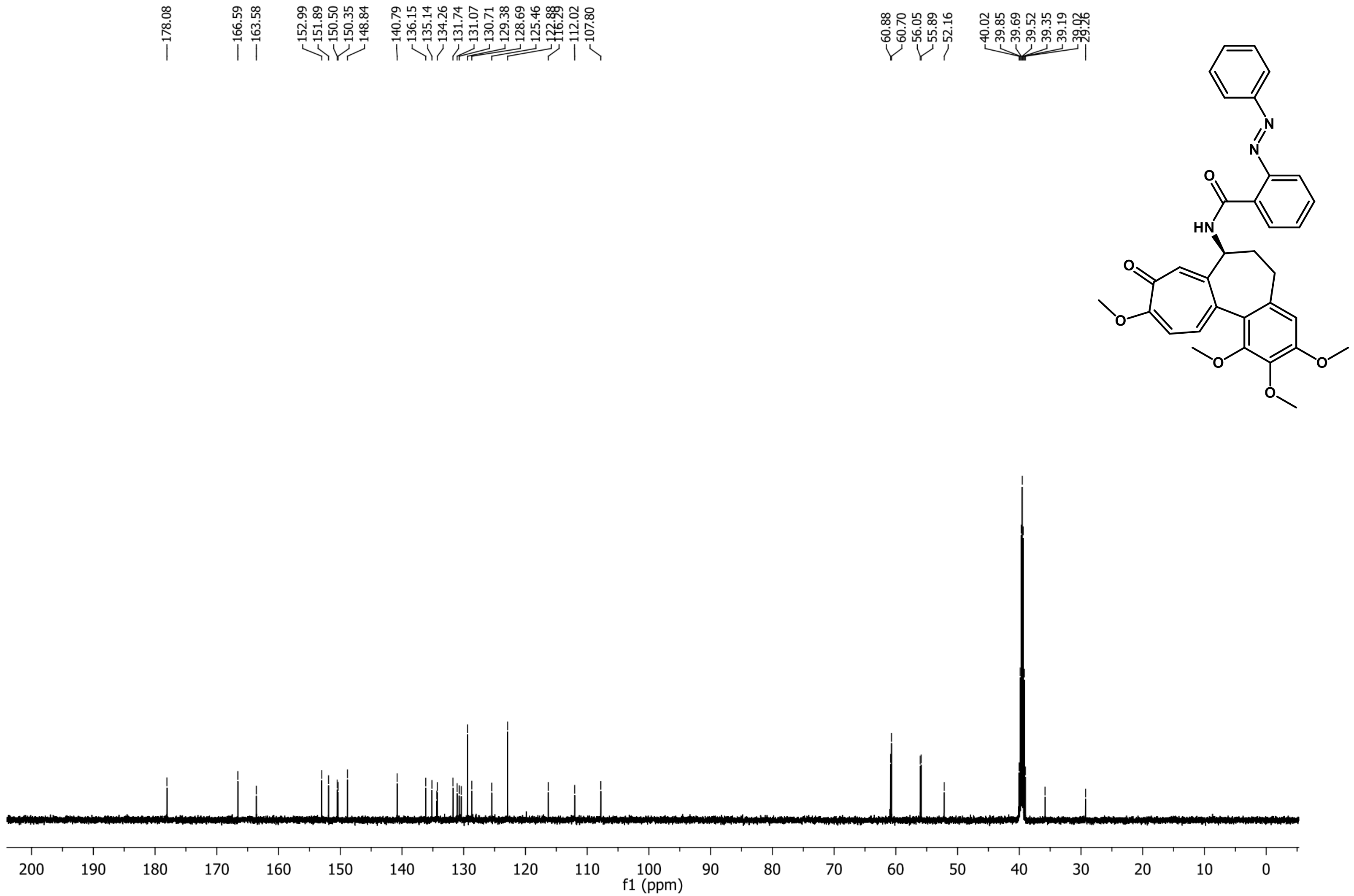

Figure S69. ^{13}C NMR spectra of *o*-**AzoCol** ((S,E)-2-(phenyldiazenyl)-N-(1,2,3,10-tetramethoxy-9-oxo-5,6,7,9-tetrahydrobenzo[a]heptalen-7-yl)benzamide).

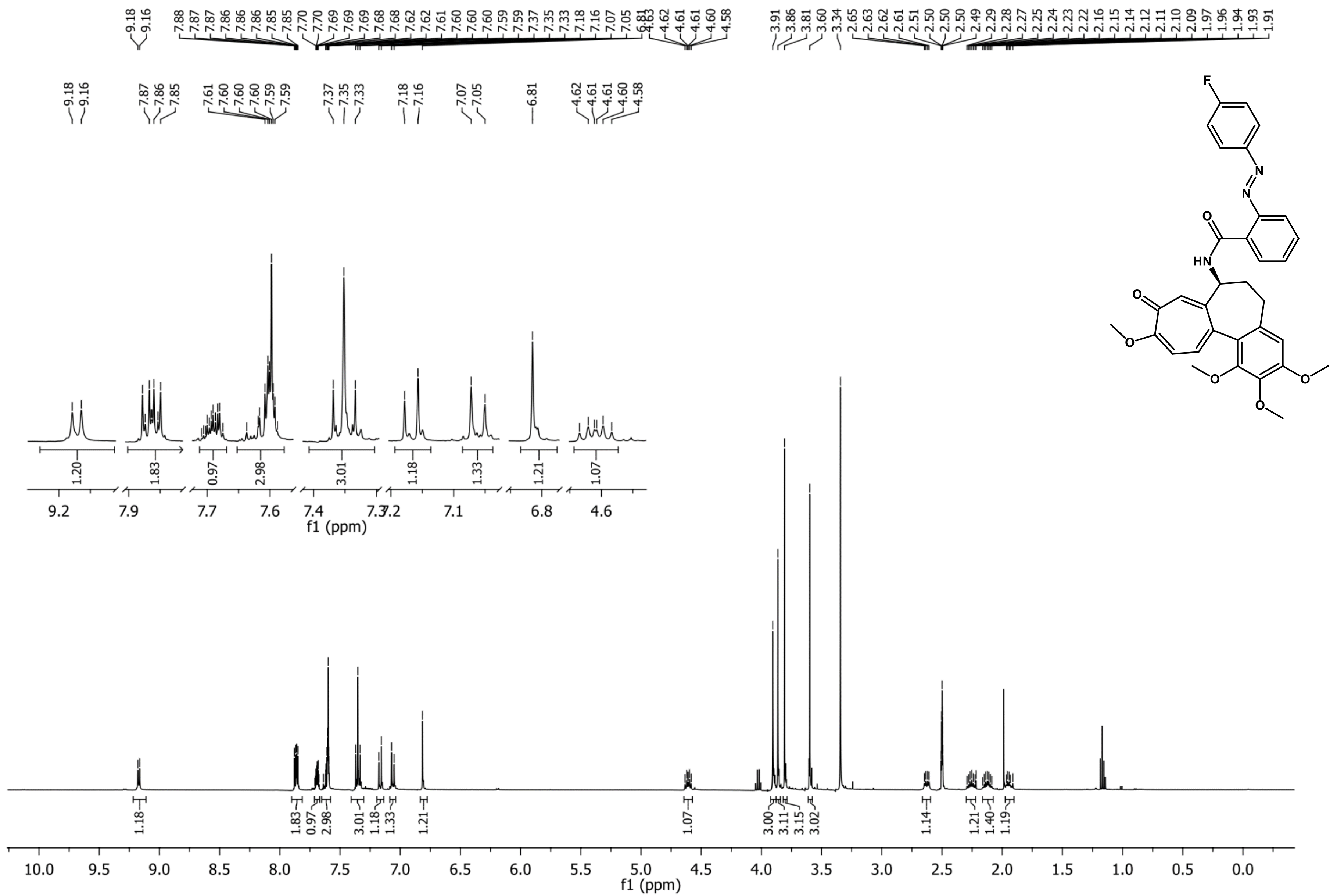

Figure S70. ^{1}H NMR spectra of *o*-**AzoCol4F** (S,E)-2-((4-fluorophenyl)diazenyl)-N-(1,2,3,10-tetramethoxy-9-oxo-5,6,7,9-tetrahydrobenzo[a]heptalen-7-yl)benzamide).

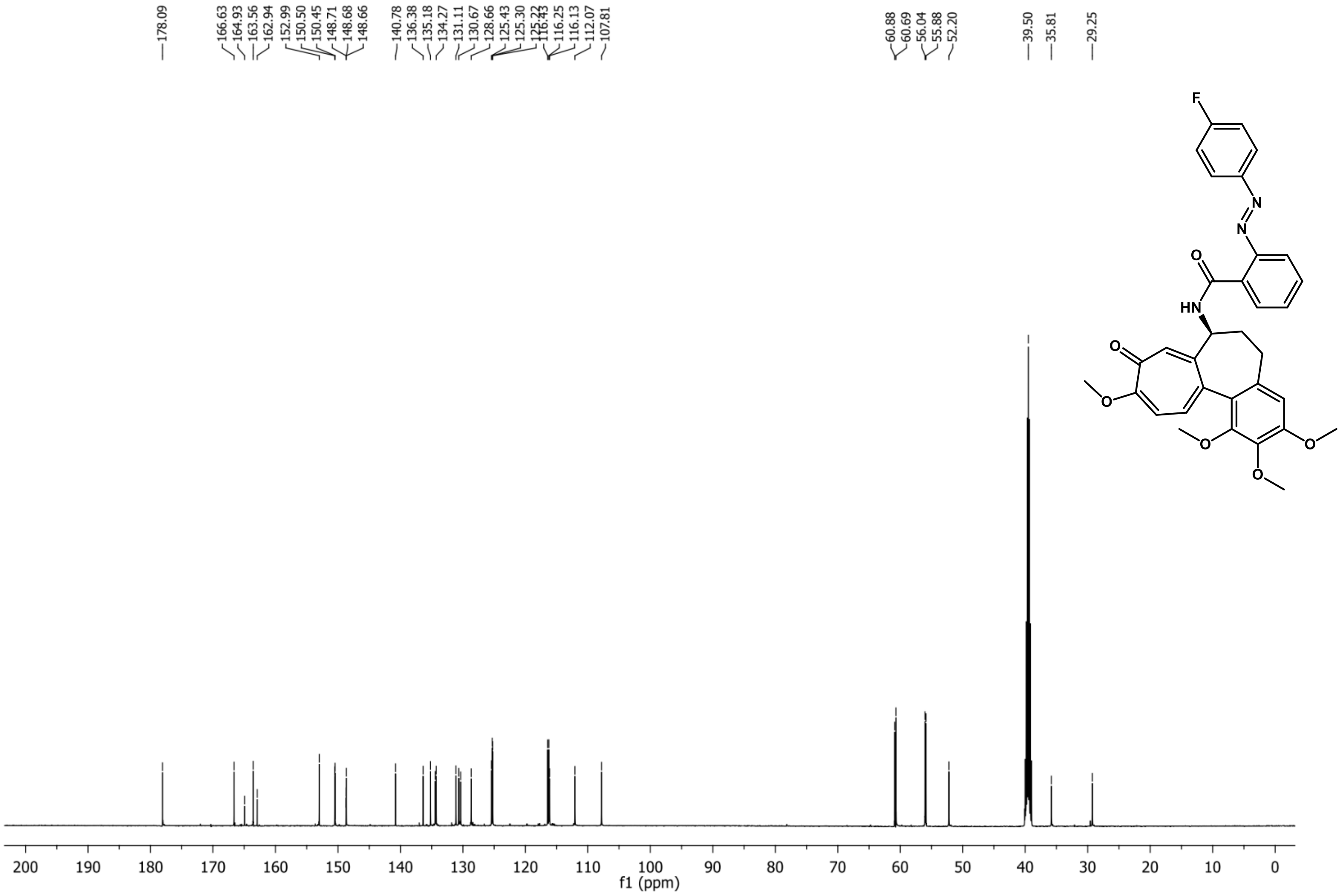

Figure S71. ^{13}C NMR spectra of *o*-**AzoCol4F** (S,E)-2-((4-fluorophenyl)diazenyl)-N-(1,2,3,10-tetramethoxy-9-oxo-5,6,7,9-tetrahydrobenzo[a]heptalen-7-yl)benzamide).

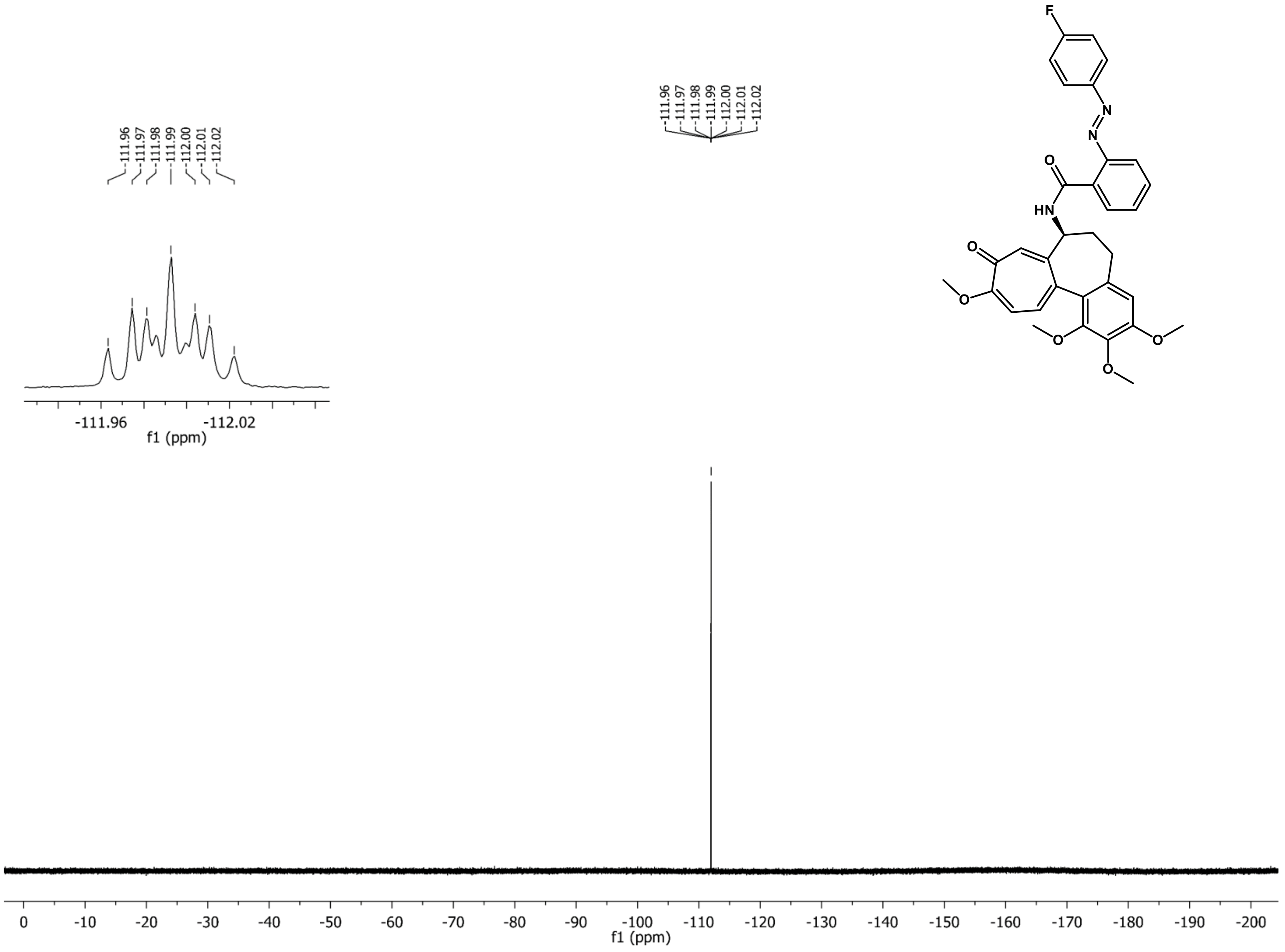

Figure S72. ^{19}F NMR spectra of *o*-**AzoCol4F** (S,E)-2-((4-fluorophenyl)diazenyl)-N-(1,2,3,10-tetramethoxy-9-oxo-5,6,7,9-tetrahydrobenzo[a]heptalen-7-yl)benzamide).

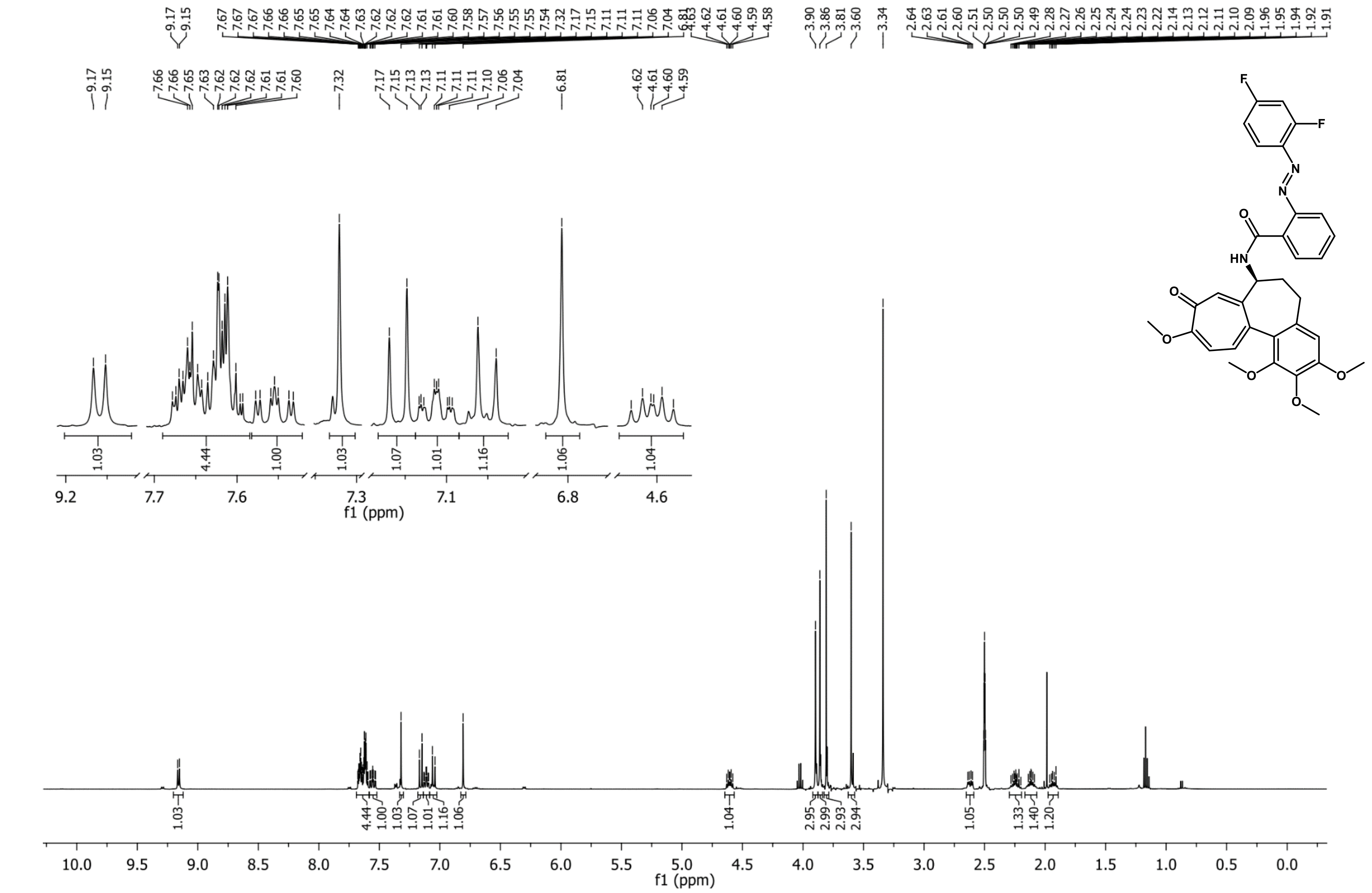

Figure S73. ^{1}H NMR spectra of *o*-**AzoCol24DF** ((S,E)-2-((2,4-difluorophenyl)diazenyl)-N-(1,2,3,10-tetramethoxy-9-oxo-5,6,7,9-tetrahydrobenzo[a]heptalen-7-yl)benzamide).

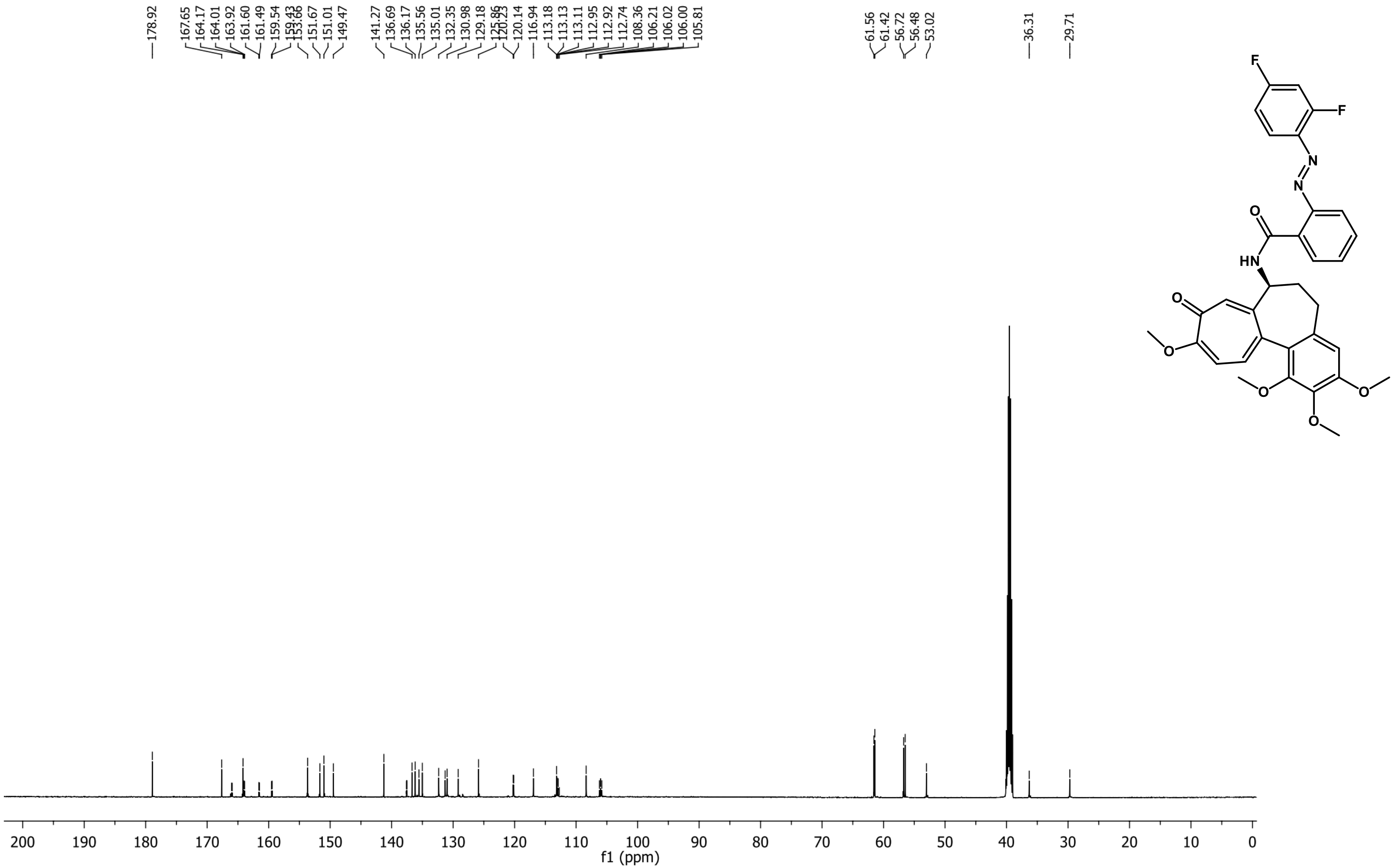

Figure S74. ^{13}C NMR spectra of *o*-**AzoCol24DF** ((S,E)-2-((2,4-difluorophenyl)diazenyl)-N-(1,2,3,10-tetramethoxy-9-oxo-5,6,7,9-tetrahydrobenzo[a]heptalen-7-yl)benzamide).

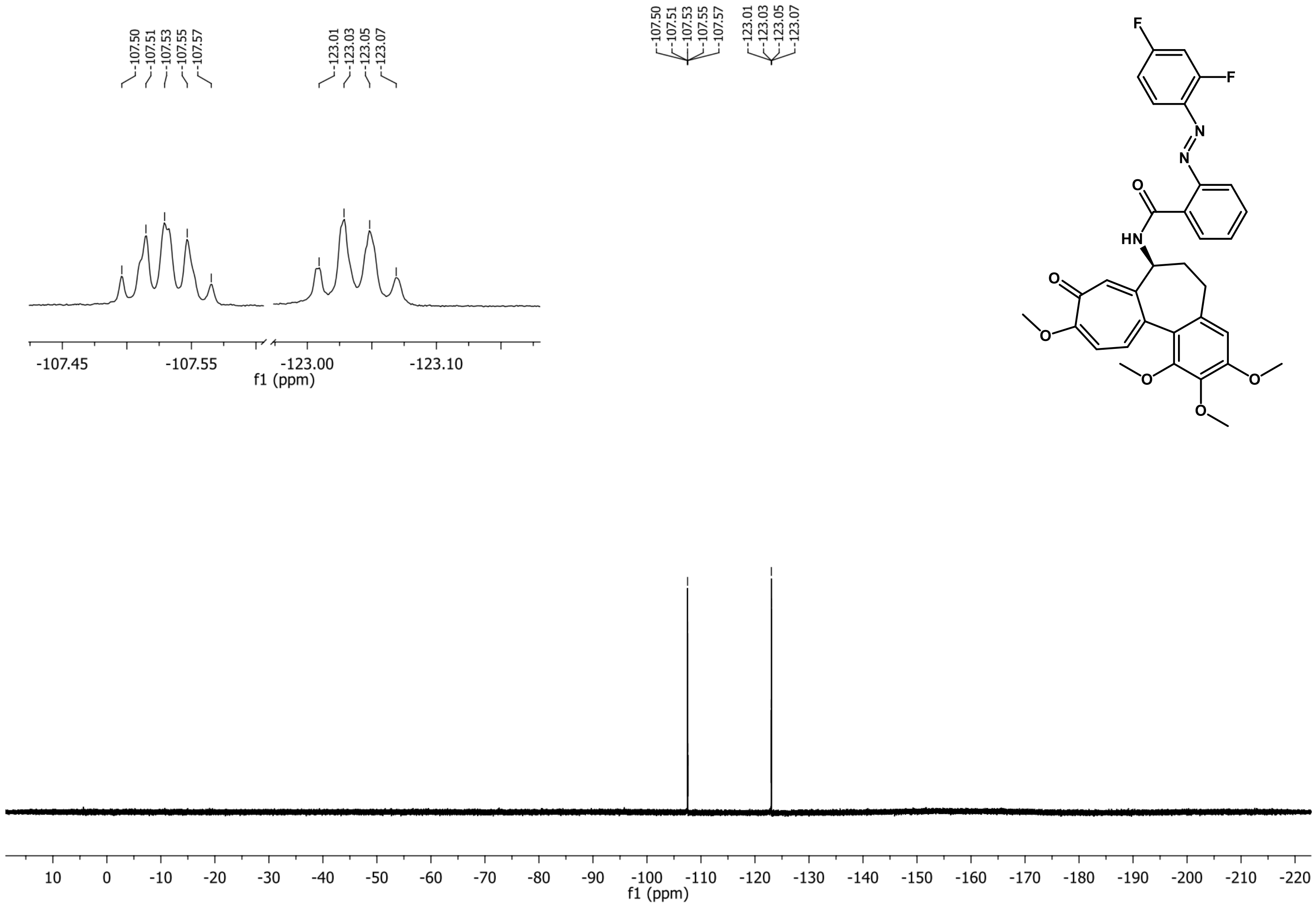

Figure S75. ^{19}F NMR spectra of *o*-**AzoCol24DF** ((S,E)-2-((2,4-difluorophenyl)diazenyl)-N-(1,2,3,10-tetramethoxy-9-oxo-5,6,7,9-tetrahydrobenzo[a]heptalen-7-yl)benzamide).

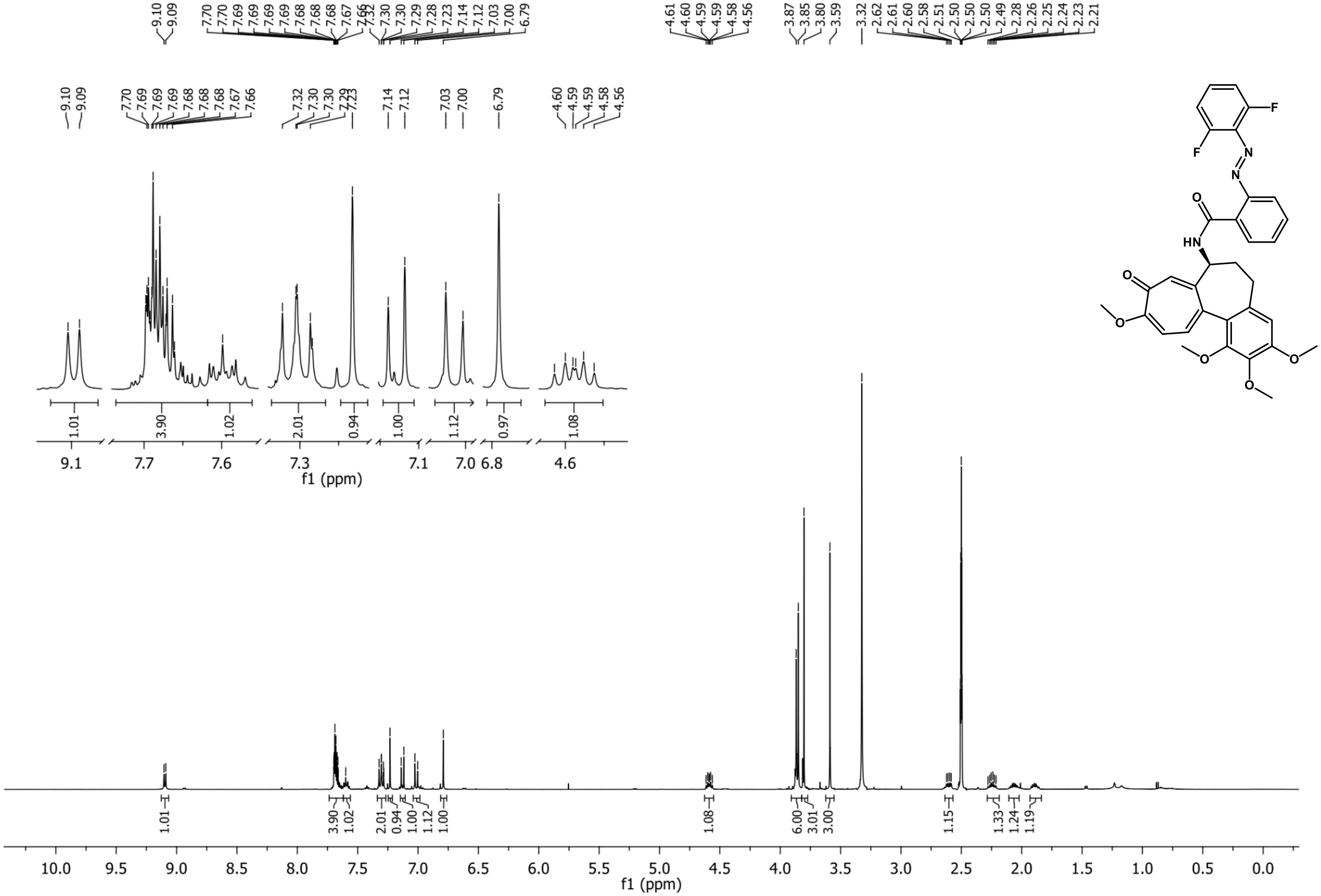

Figure S76. ^{1}H NMR spectra of *o*-**AzoCol26DF** (S,E)-2-((2,6-difluorophenyl)diazenyl)-N-(1,2,3,10-tetramethoxy-9-oxo-5,6,7,9-tetrahydrobenzo[a]heptalen-7-yl)benzamide).

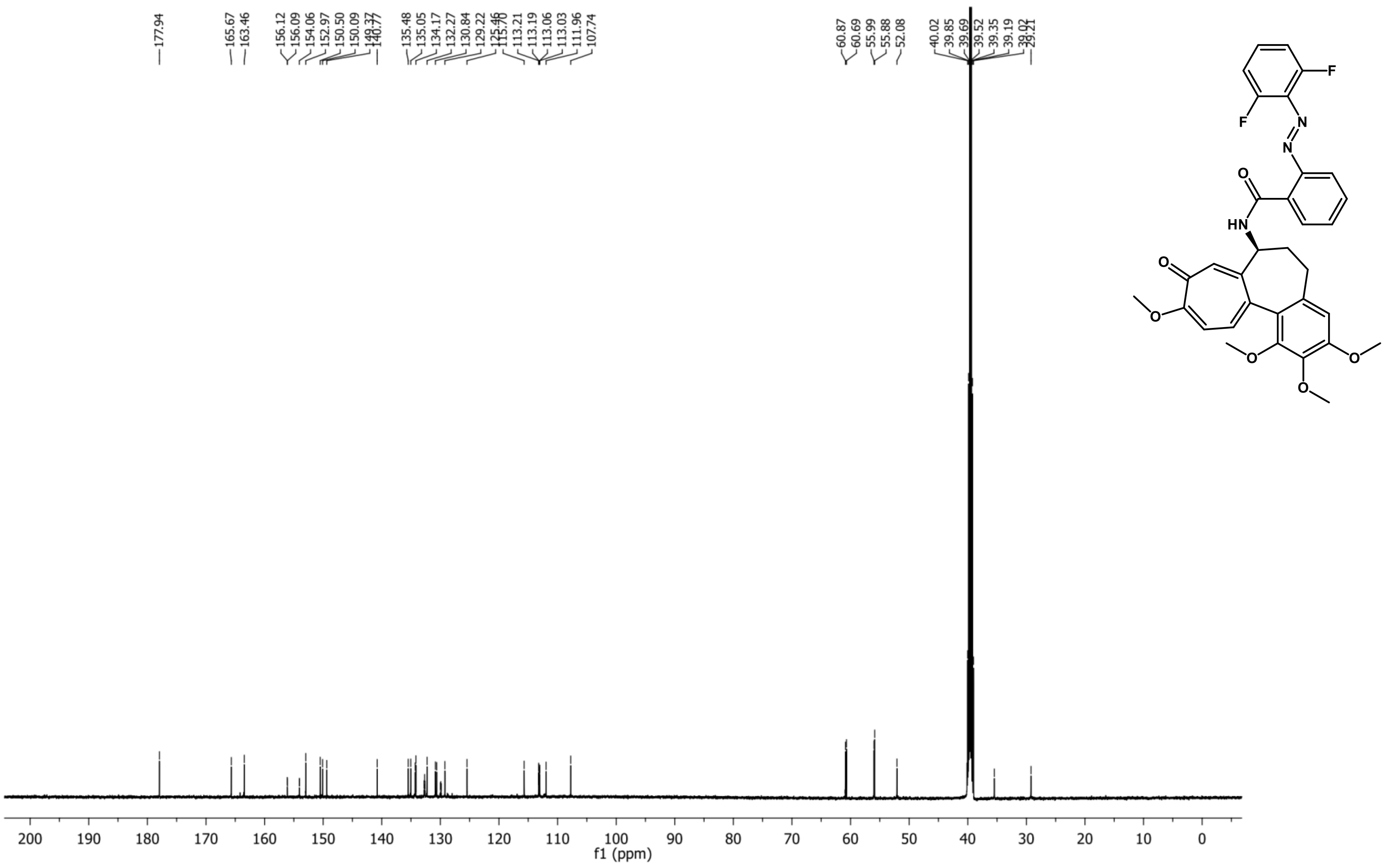

Figure S77. ^{13}C NMR spectra of *o*-**AzoCol26DF** (S,E)-2-((2,6-difluorophenyl)diazenyl)-N-(1,2,3,10-tetramethoxy-9-oxo-5,6,7,9-tetrahydrobenzo[a]heptalen-7-yl)benzamide).

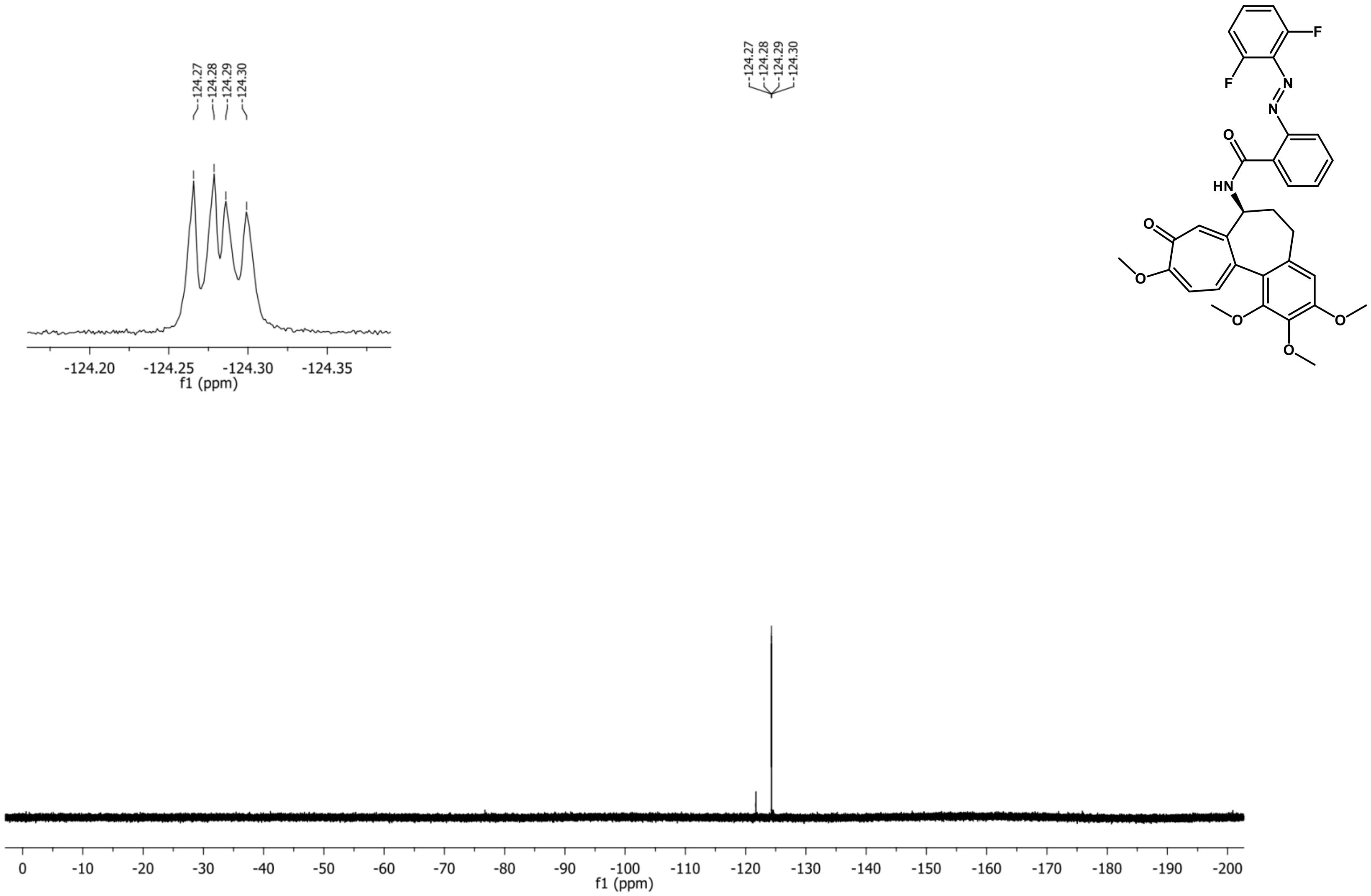

Figure S78. ^{19}F NMR spectra of *o*-**AzoCol26DF** (S,E)-2-((2,6-difluorophenyl)diazenyl)-N-(1,2,3,10-tetramethoxy-9-oxo-5,6,7,9-tetrahydrobenzo[a]heptalen-7-yl)benzamide).

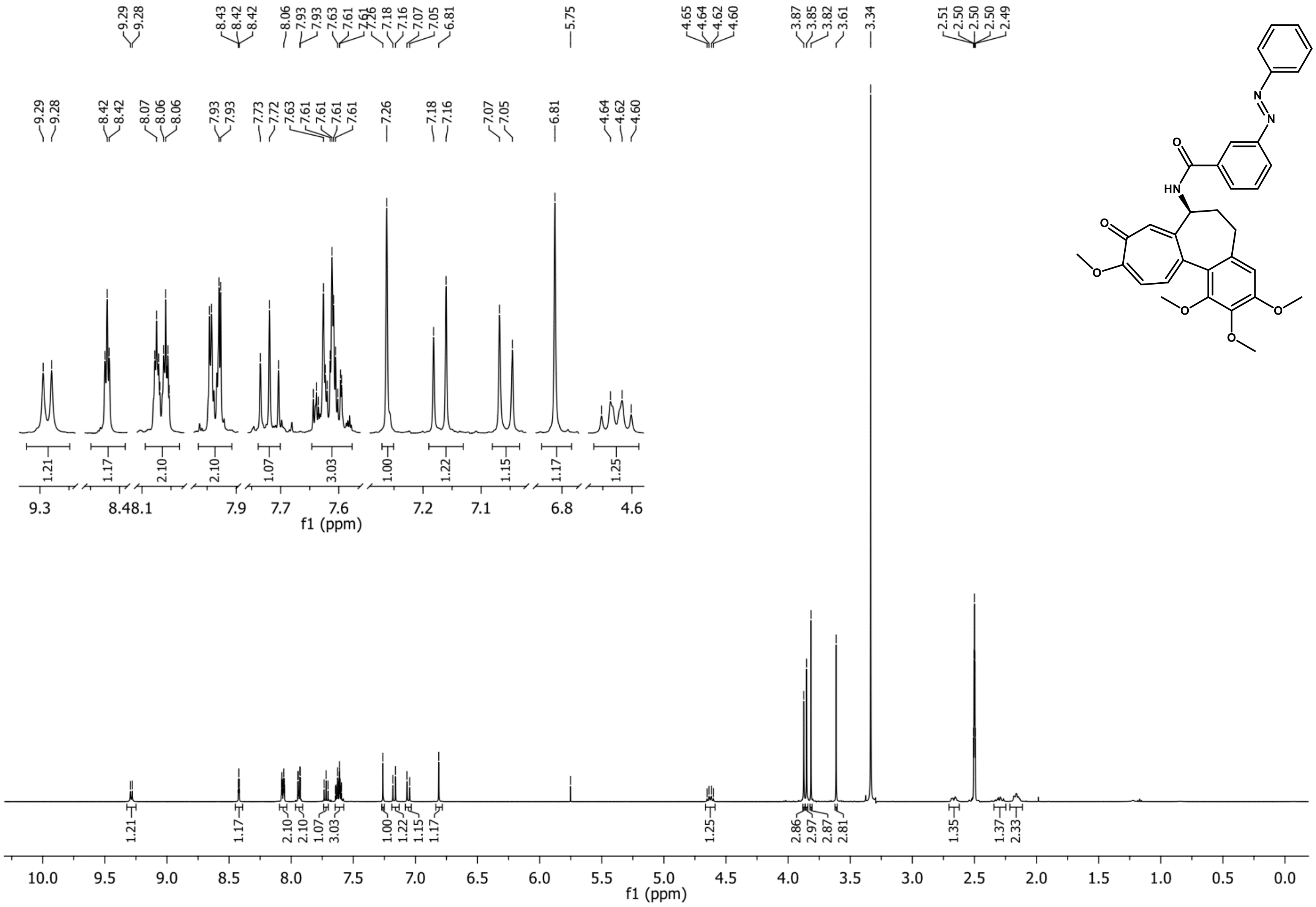

Figure S79. ^{1}H NMR spectra of *m*-**AzoCol** ((S,E)-3-(phenyldiazenyl)-N-(1,2,3,10-tetramethoxy-9-oxo-5,6,7,9-tetrahydrobenzo[a]heptalen-7-yl)benzamide).

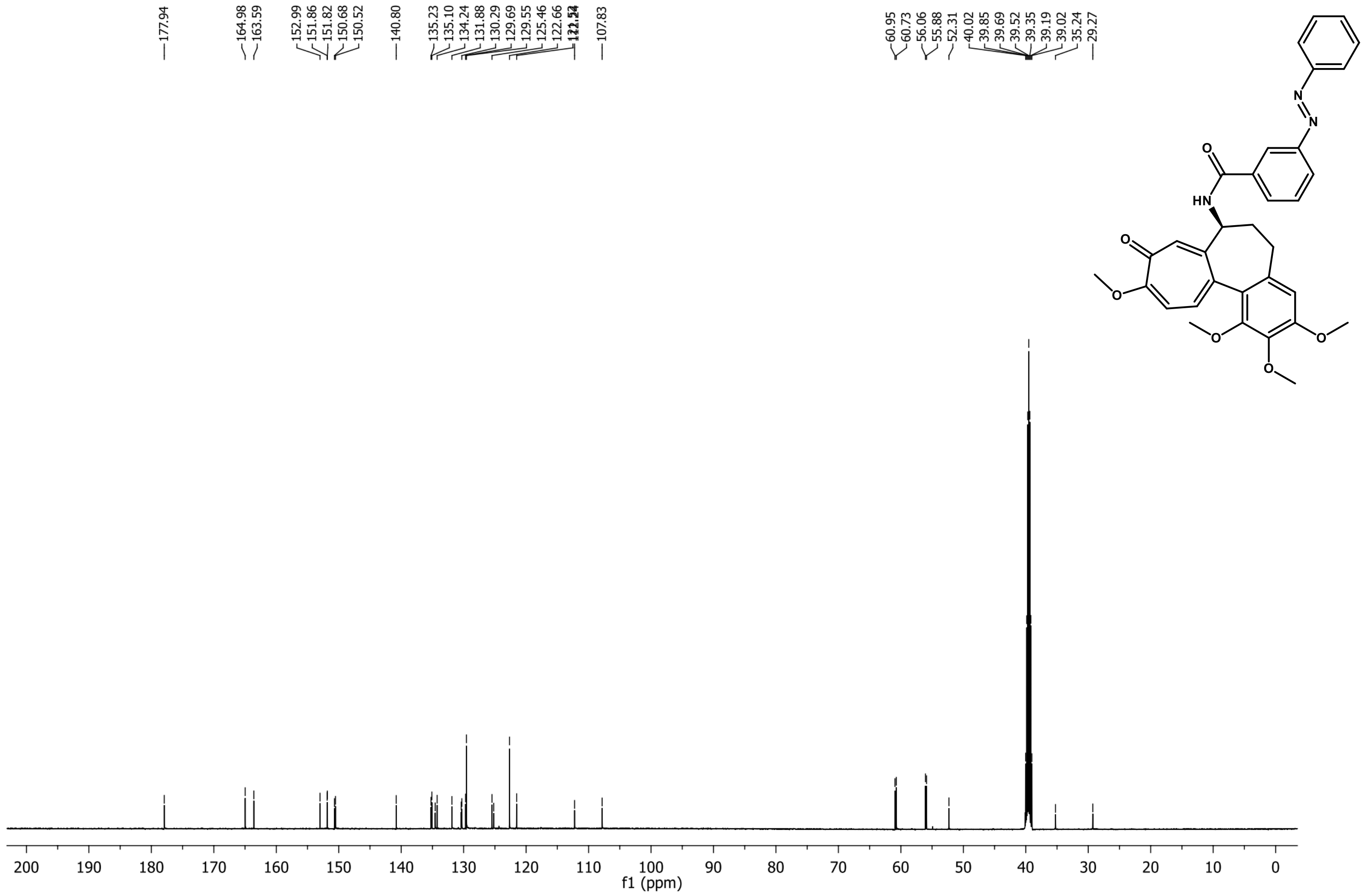

Figure S80. ^{13}C NMR spectra of *m*-**AzoCol** ((S,E)-3-(phenyldiazenyl)-N-(1,2,3,10-tetramethoxy-9-oxo-5,6,7,9-tetrahydrobenzo[a]heptalen-7-yl)benzamide).

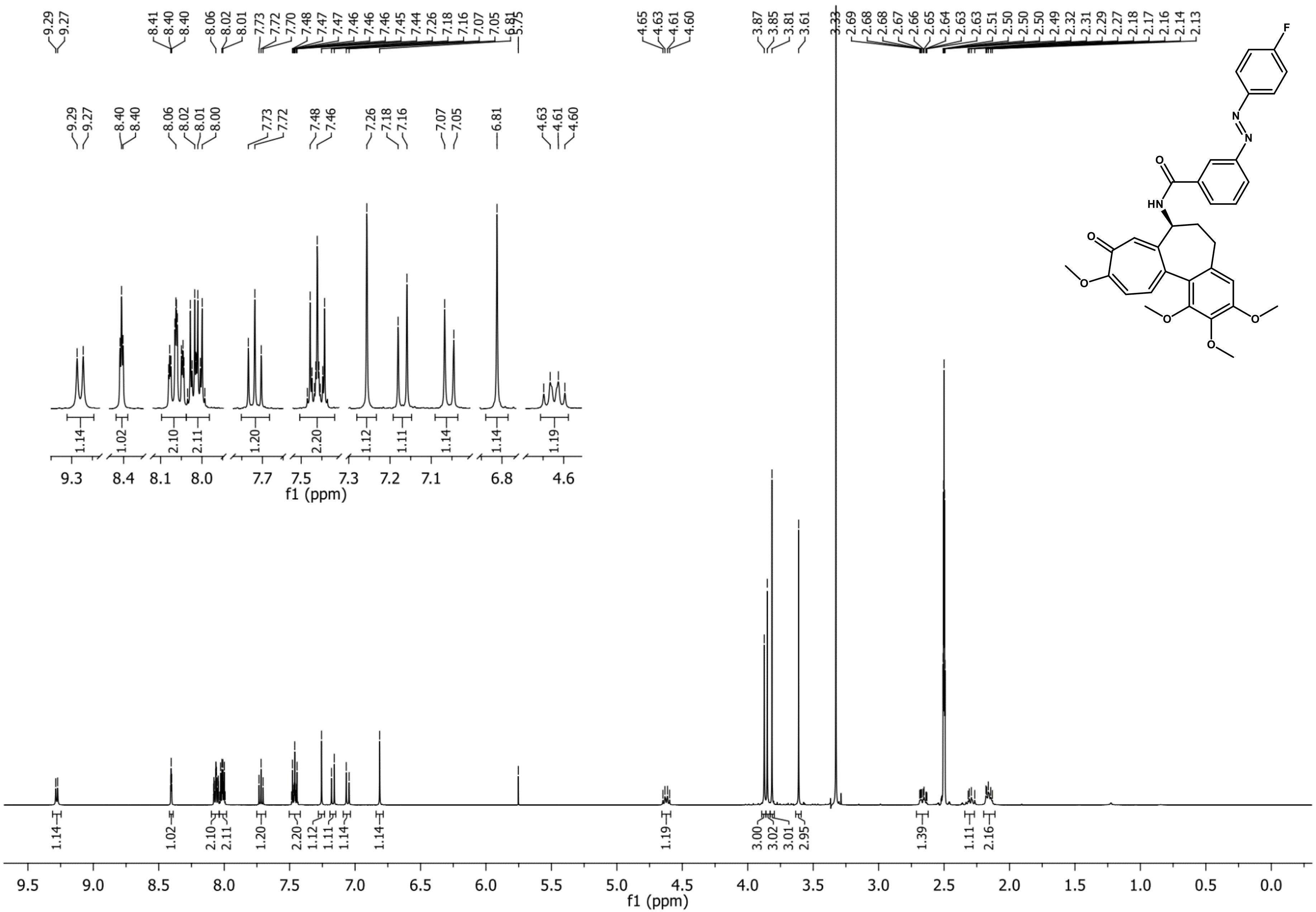

Figure S81. ^{1}H NMR spectra of *m*-**AzoCol4F** ((S,E)-3-((4-fluorophenyl)diazenyl)-N-(1,2,3,10-tetramethoxy-9-oxo-5,6,7,9-tetrahydrobenzo[a]heptalen-7-yl)benzamide).

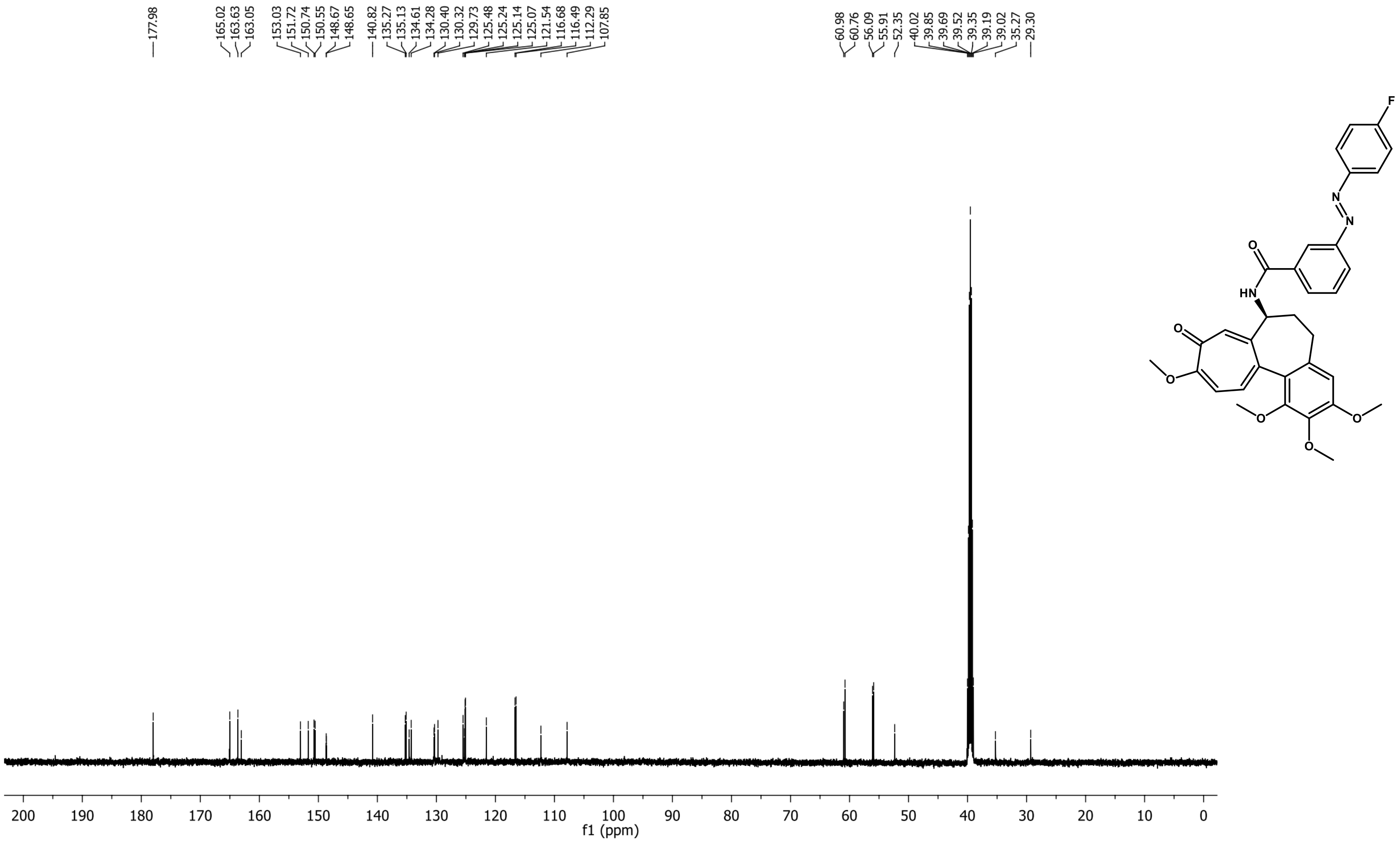

Figure S82. ^{13}C NMR spectra of *m*-**AzoCol4F** ((S,E)-3-((4-fluorophenyl)diazenyl)-N-(1,2,3,10-tetramethoxy-9-oxo-5,6,7,9-tetrahydrobenzo[a]heptalen-7-yl)benzamide).

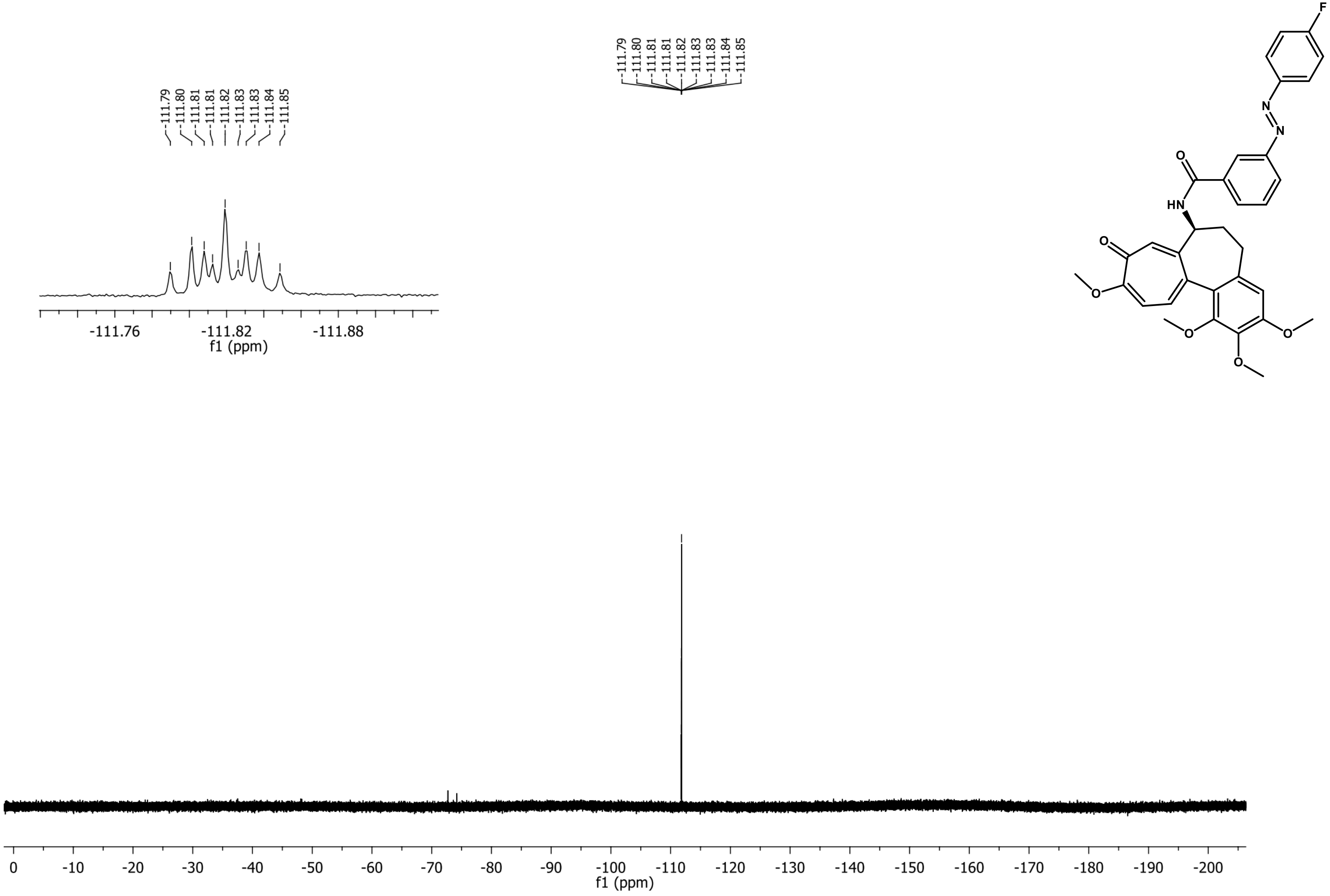

Figure S83. ^{19}F NMR spectra of *m*-**AzoCol4F** ((S,E)-3-((4-fluorophenyl)diazenyl)-N-(1,2,3,10-tetramethoxy-9-oxo-5,6,7,9-tetrahydrobenzo[a]heptalen-7-yl)benzamide).

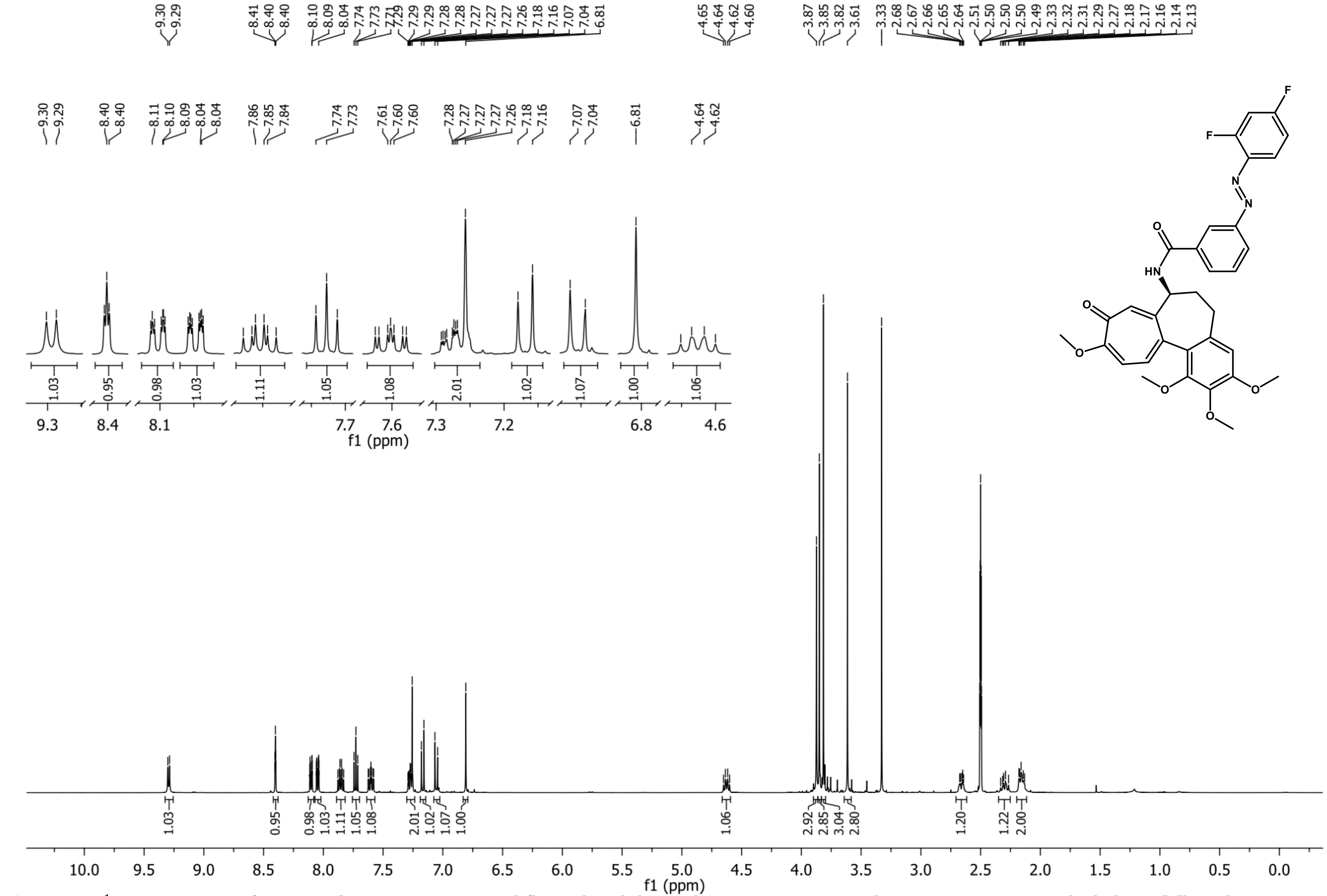

Figure S84. ^{1}H NMR spectra of *m*-**AzoCol24DF** ((S,E)-3-((2,4-difluorophenyl)diazenyl)-N-(1,2,3,10-tetramethoxy-9-oxo-5,6,7,9-tetrahydrobenzo[a]heptalen-7-yl)benzamide).

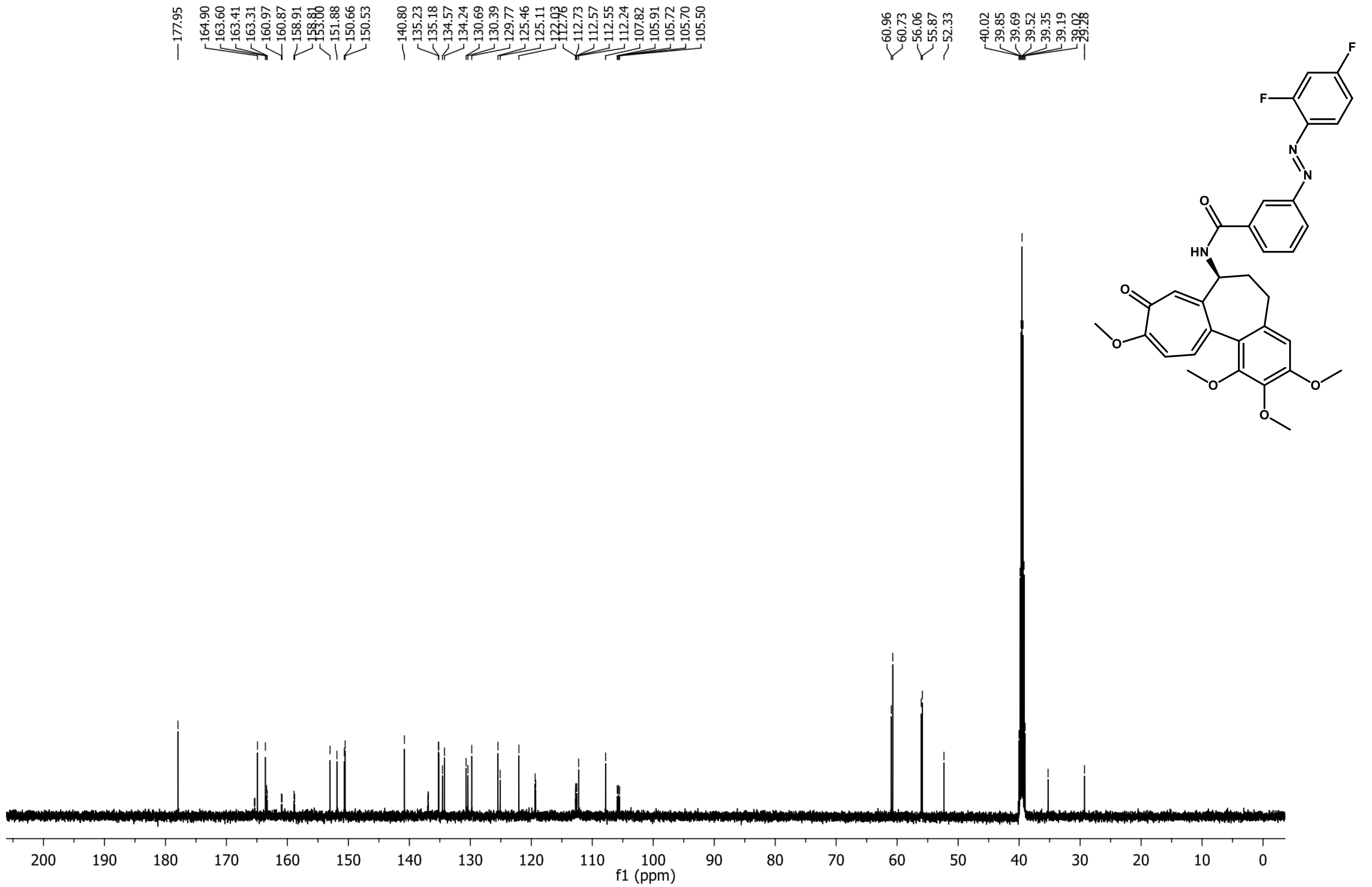

Figure S85. ^{13}C NMR spectra of *m*-**AzoCol24DF** ((S,E)-3-((2,4-difluorophenyl)diazenyl)-N-(1,2,3,10-tetramethoxy-9-oxo-5,6,7,9-tetrahydrobenzo[a]heptalen-7-yl)benzamide).

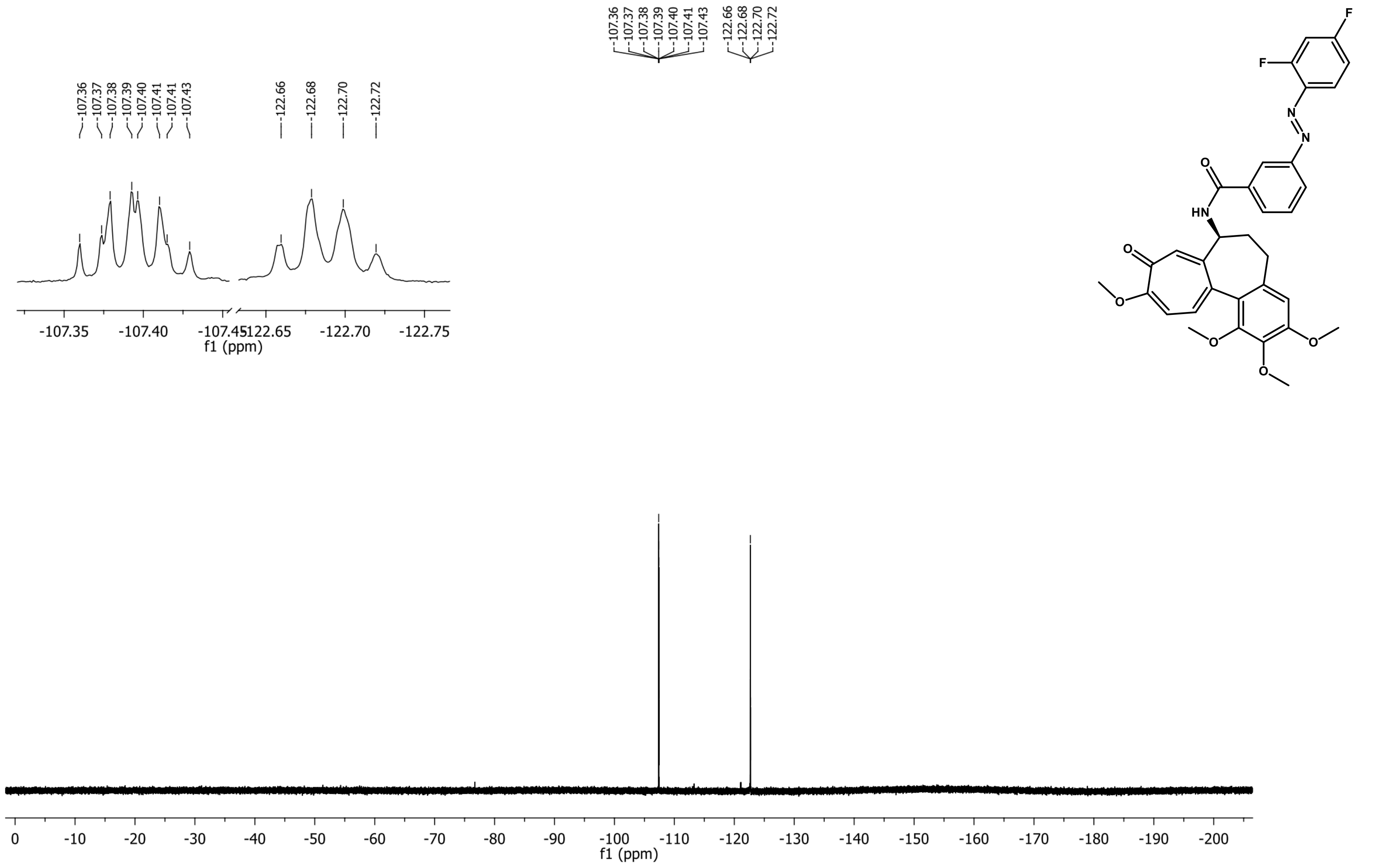

Figure S86. ^{19}F NMR spectra of *m*-**AzoCol24DF** ((S,E)-3-((2,4-difluorophenyl)diazenyl)-N-(1,2,3,10-tetramethoxy-9-oxo-5,6,7,9-tetrahydrobenzo[a]heptalen-7-yl)benzamide).

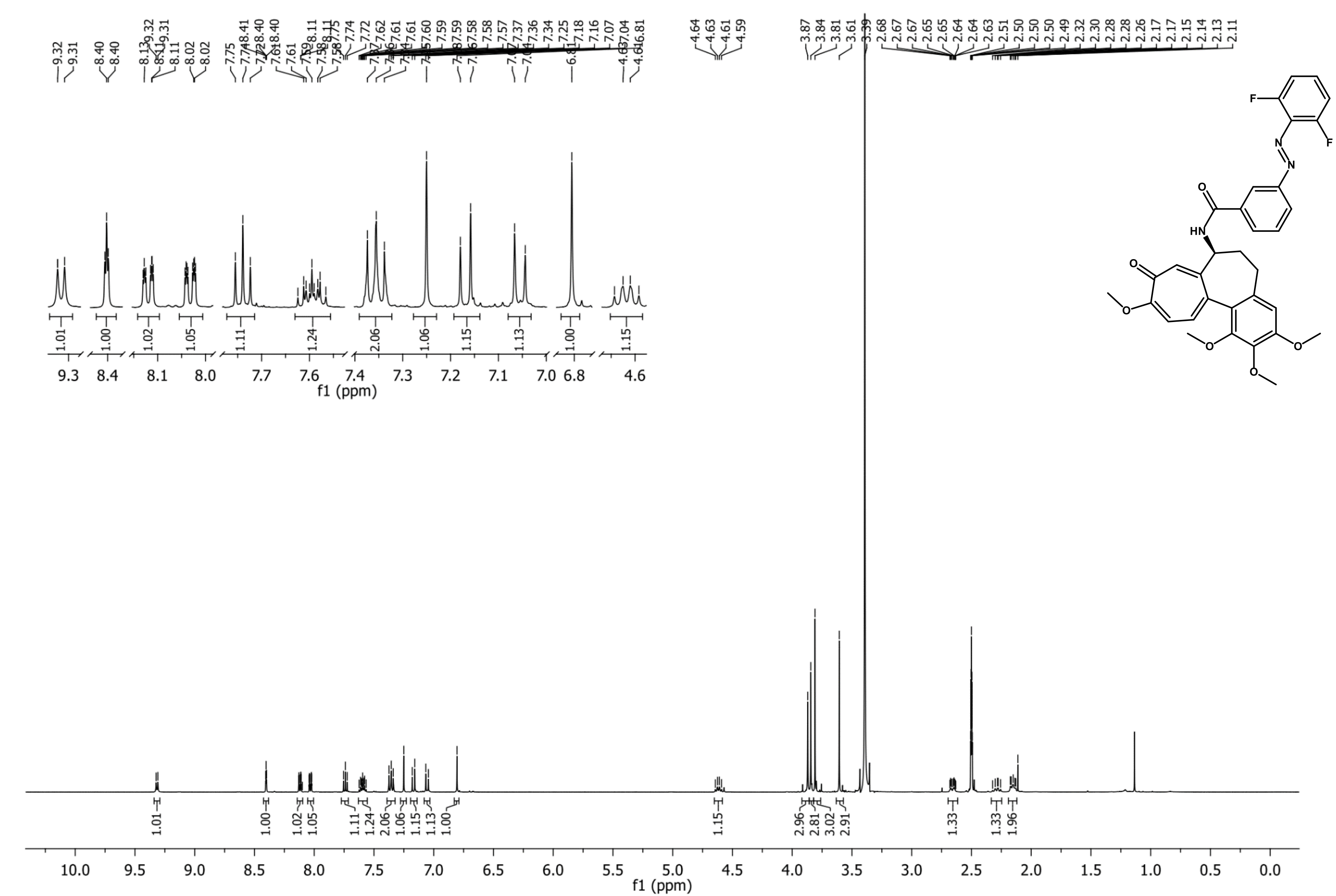

Figure S87. ^{1}H NMR spectra of *m*-**AzoCol26DF** ((S,E)-3-((2,6-difluorophenyl)diazenyl)-N-(1,2,3,10-tetramethoxy-9-oxo-5,6,7,9-tetrahydrobenzo[a]heptalen-7-yl)benzamide).

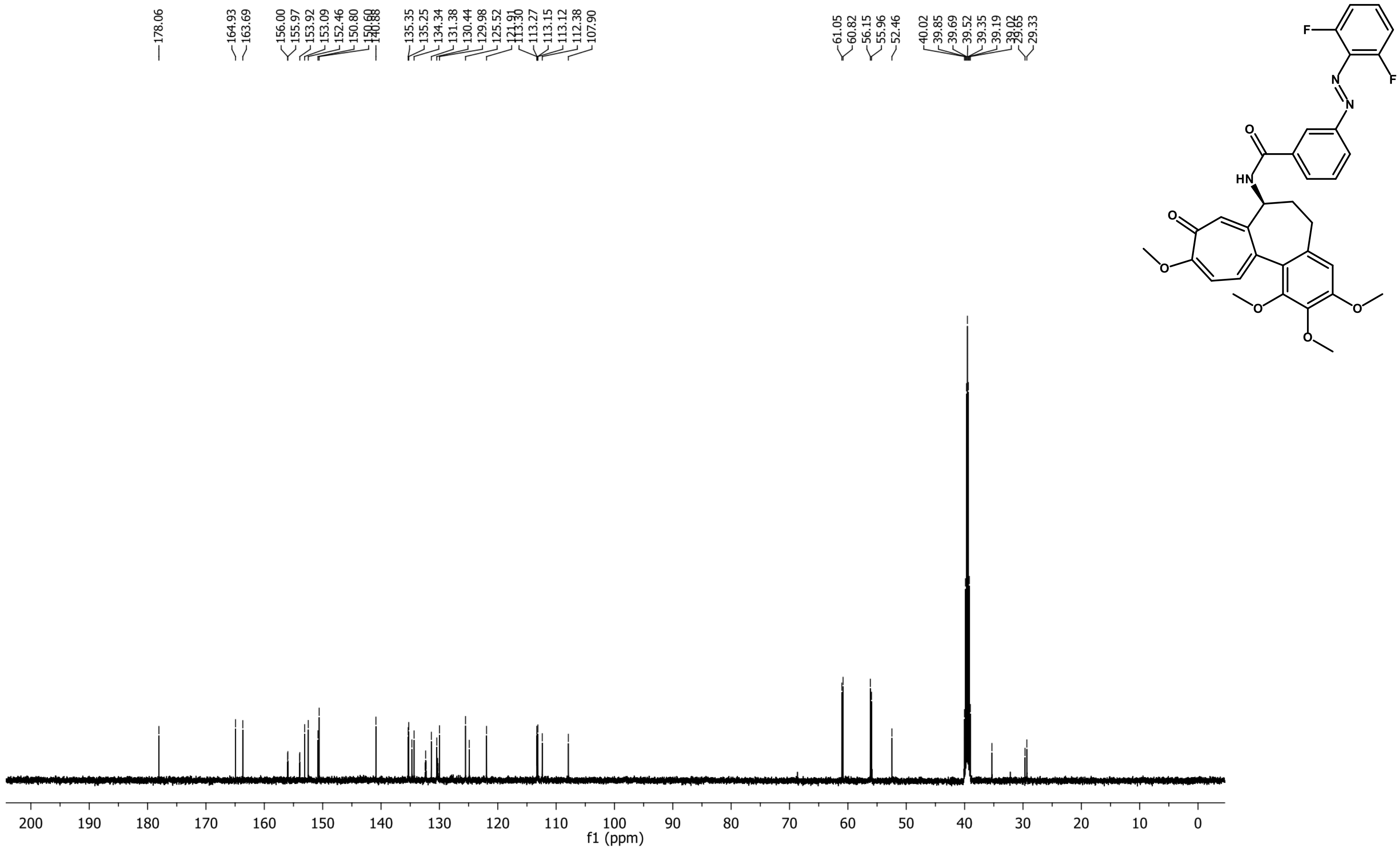

Figure S88. ^{13}C NMR spectra of *m*-**AzoCol26DF** ((S,E)-3-((2,6-difluorophenyl)diazenyl)-N-(1,2,3,10-tetramethoxy-9-oxo-5,6,7,9-tetrahydrobenzo[a]heptalen-7-yl)benzamide).

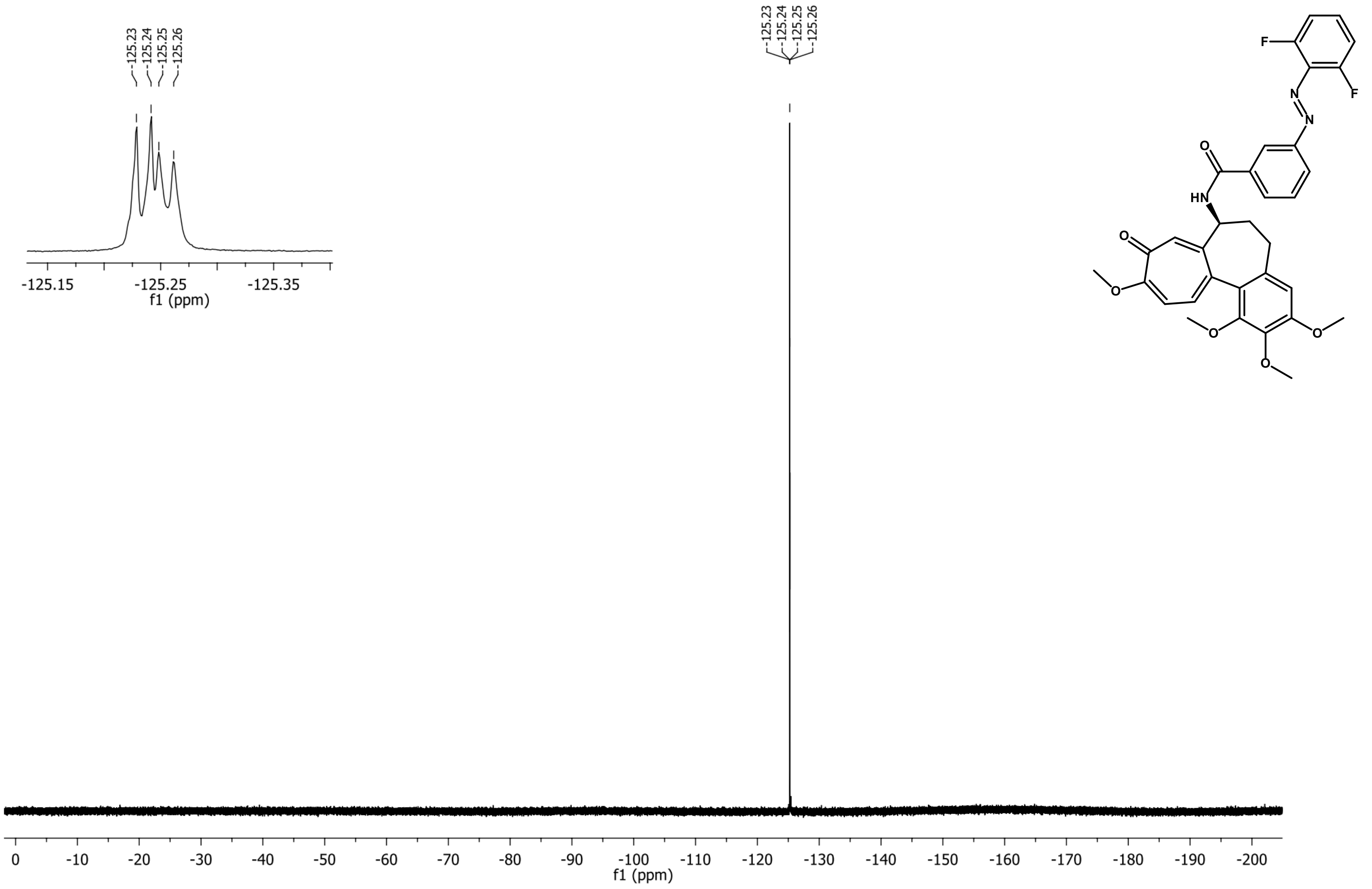

Figure S89. ^{19}F NMR spectra of *m*-**AzoCol26DF** ((S,E)-3-((2,6-difluorophenyl)diazenyl)-N-(1,2,3,10-tetramethoxy-9-oxo-5,6,7,9-tetrahydrobenzo[a]heptalen-7-yl)benzamide).

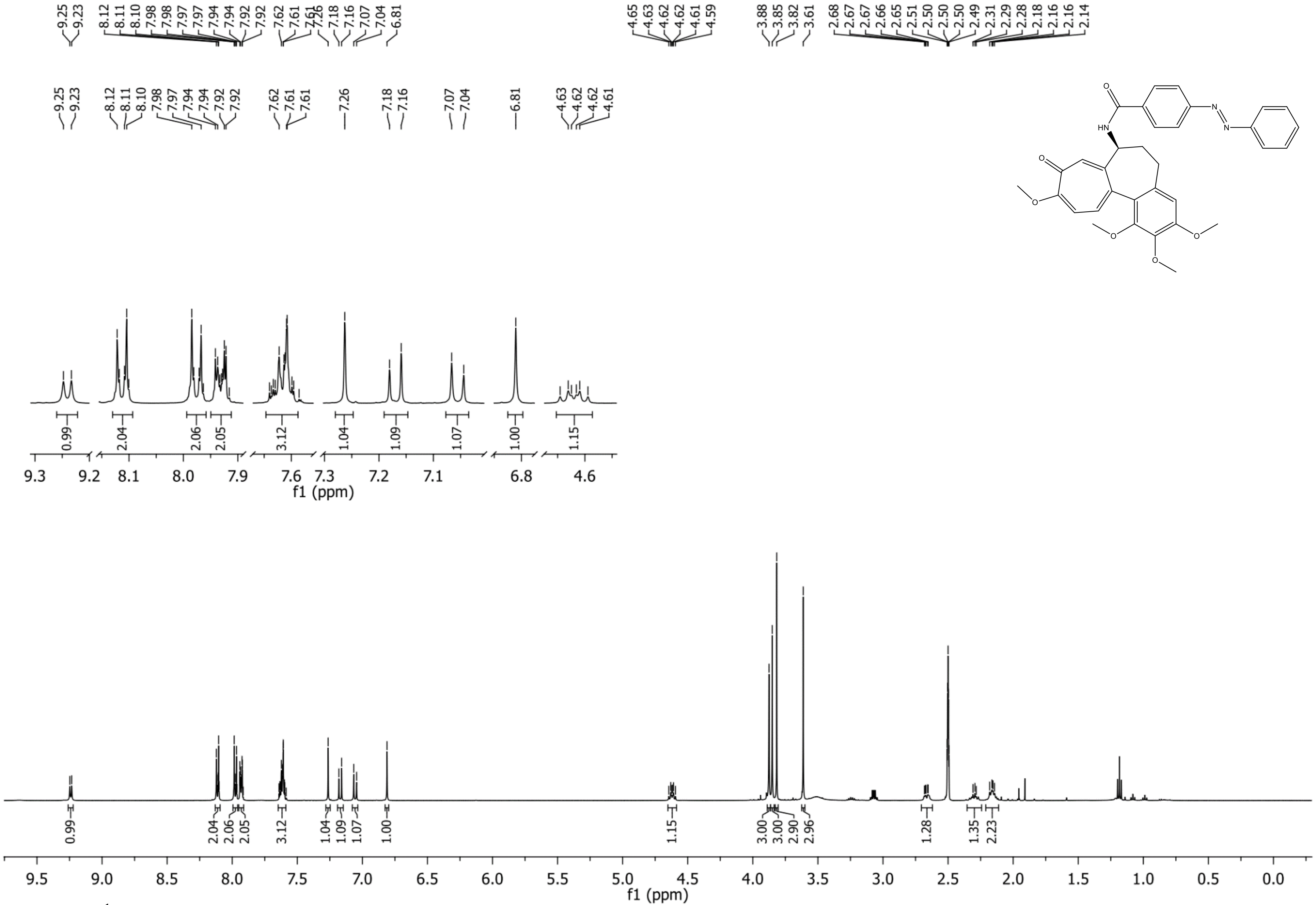

Figure S90. ^{1}H NMR spectra of *p*-**AzoCol** ((S,E)-4-(phenyldiazenyl)-N-(1,2,3,10-tetramethoxy-9-oxo-5,6,7,9-tetrahydrobenzo[a]heptalen-7-yl)benzamide).

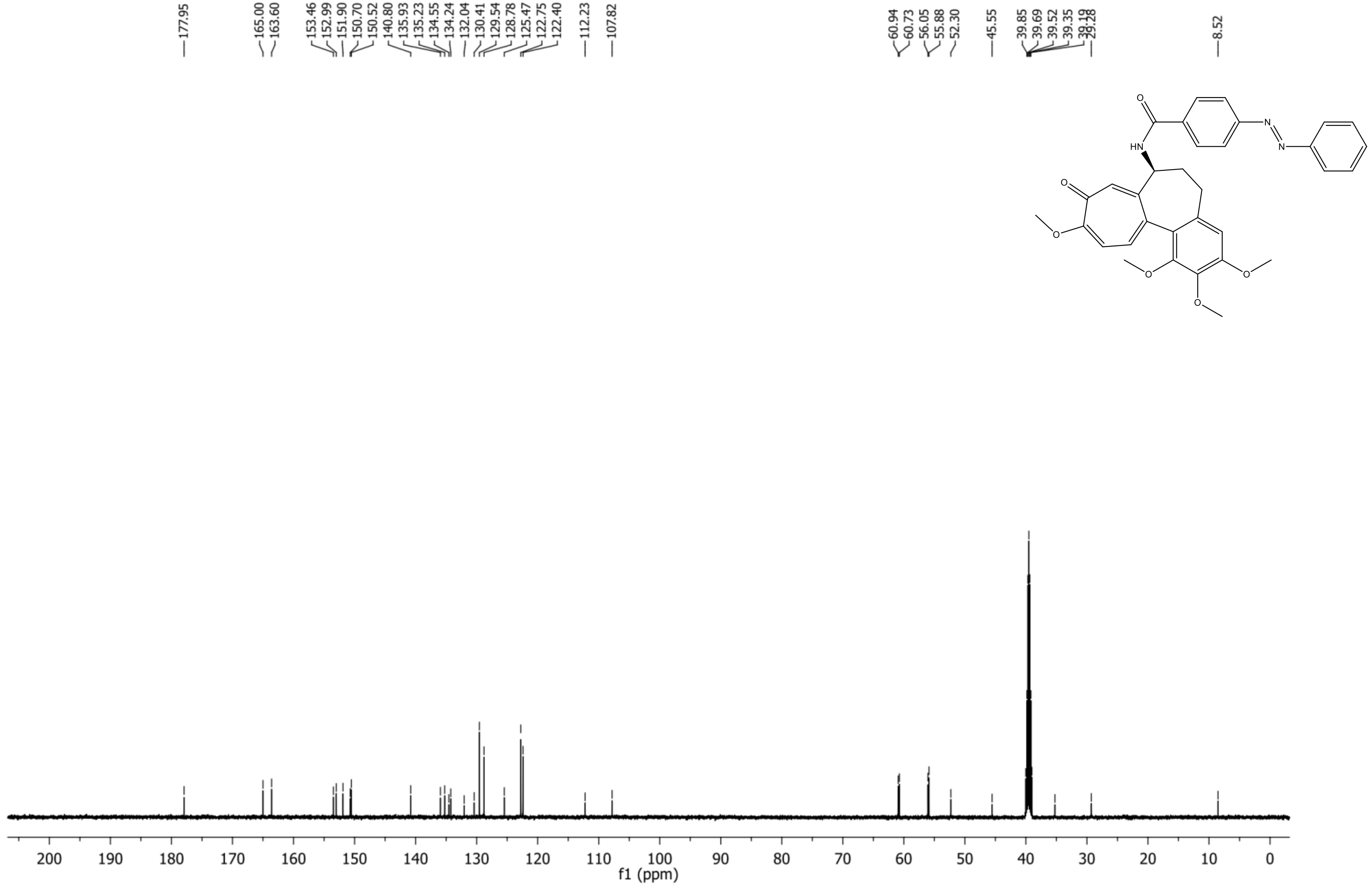

Figure S91. ^{13}C NMR spectra of *p*-**AzoCol** ((S,E)-4-(phenyldiazenyl)-N-(1,2,3,10-tetramethoxy-9-oxo-5,6,7,9-tetrahydrobenzo[a]heptalen-7-yl)benzamide).

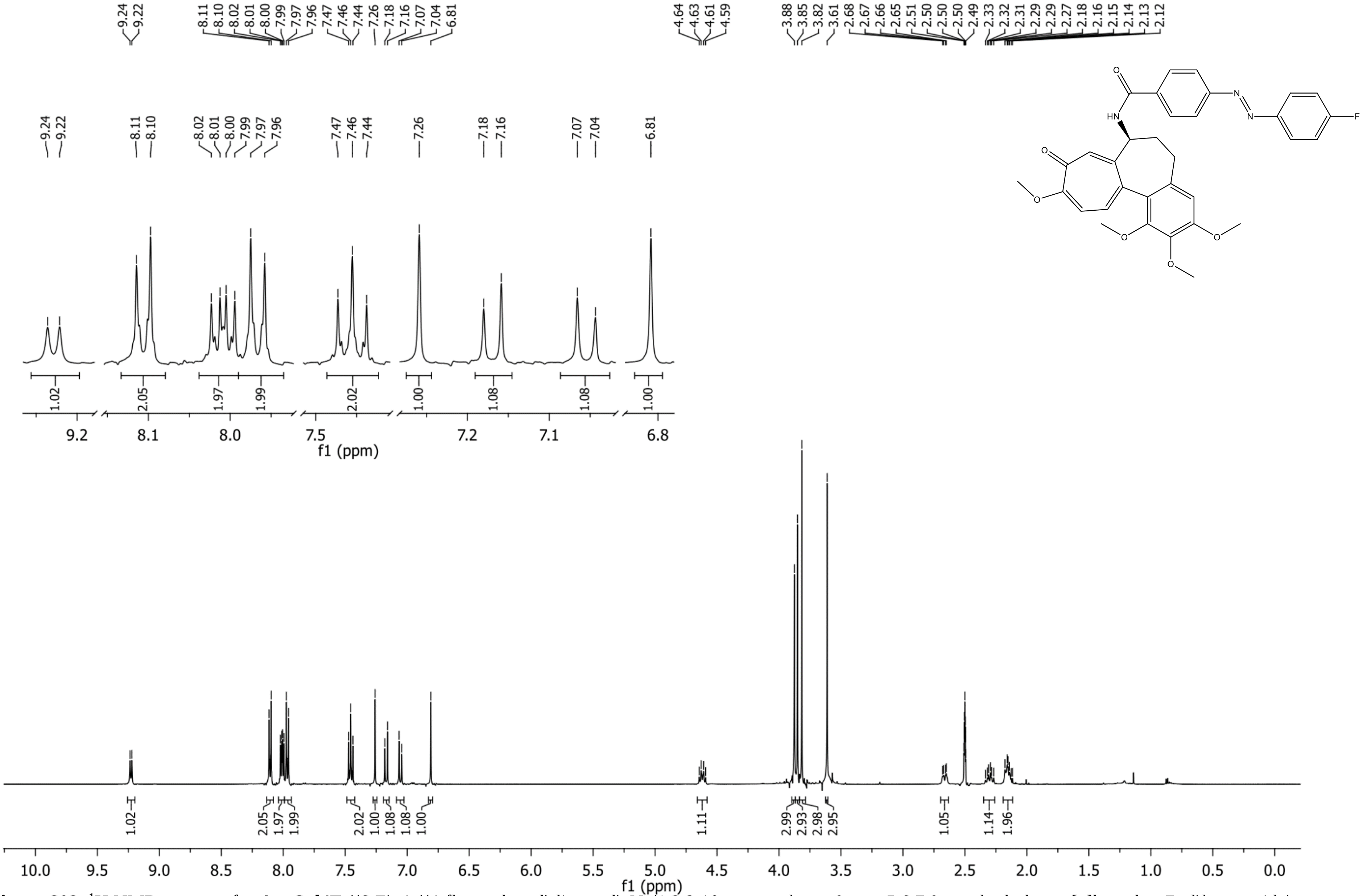

Figure S92. ^{1}H NMR spectra of *p*-**AzoCol4F** ((S,E)-4-((4-fluorophenyl)diazenyl)-N-(1,2,3,10-tetramethoxy-9-oxo-5,6,7,9-tetrahydrobenzo[a]heptalen-7-yl)benzamide).

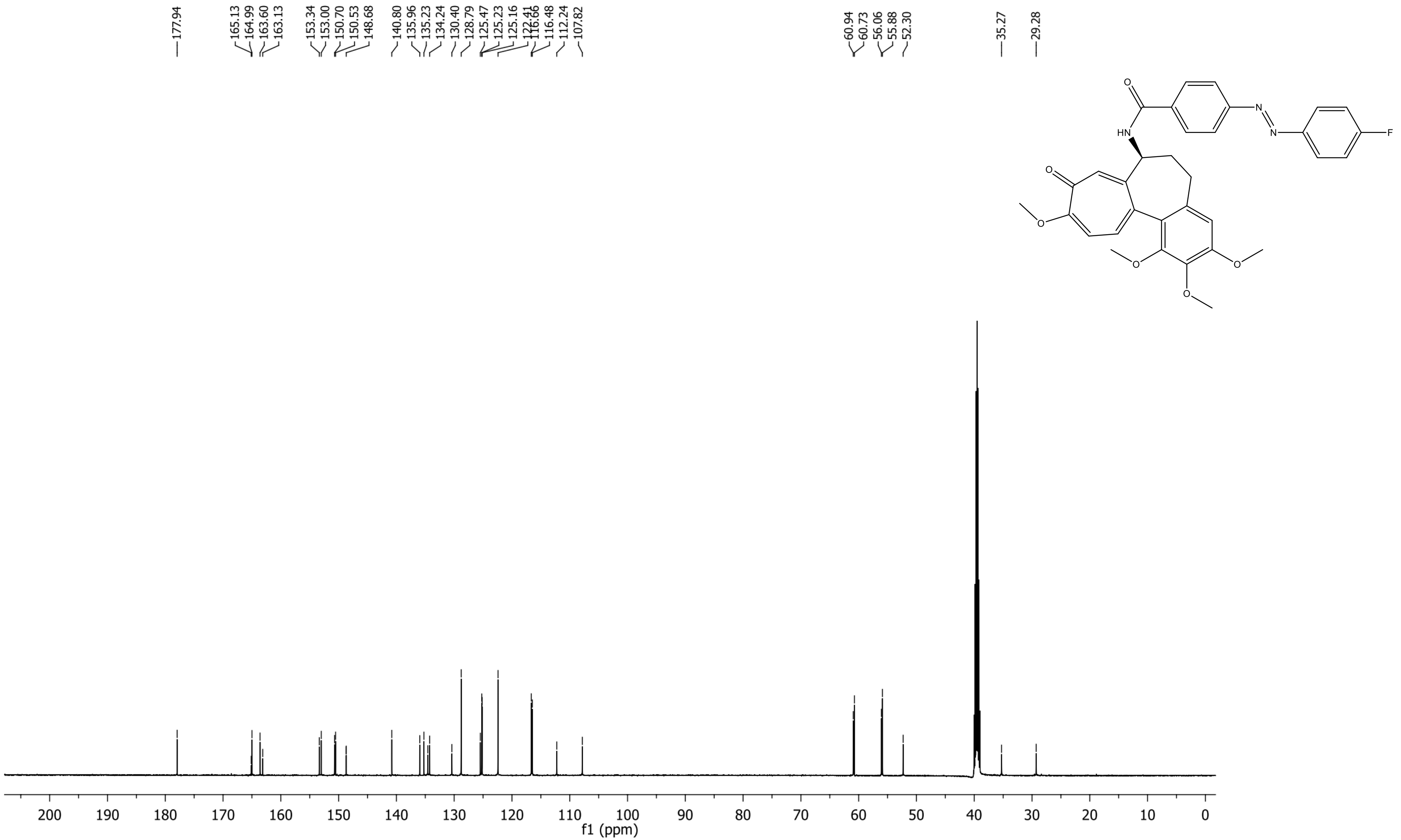

Figure S93. ^{13}C NMR spectra of *p*-**AzoCol4F** ((S,E)-4-((4-fluorophenyl)diazenyl)-N-(1,2,3,10-tetramethoxy-9-oxo-5,6,7,9-tetrahydrobenzo[a]heptalen-7-yl)benzamide).

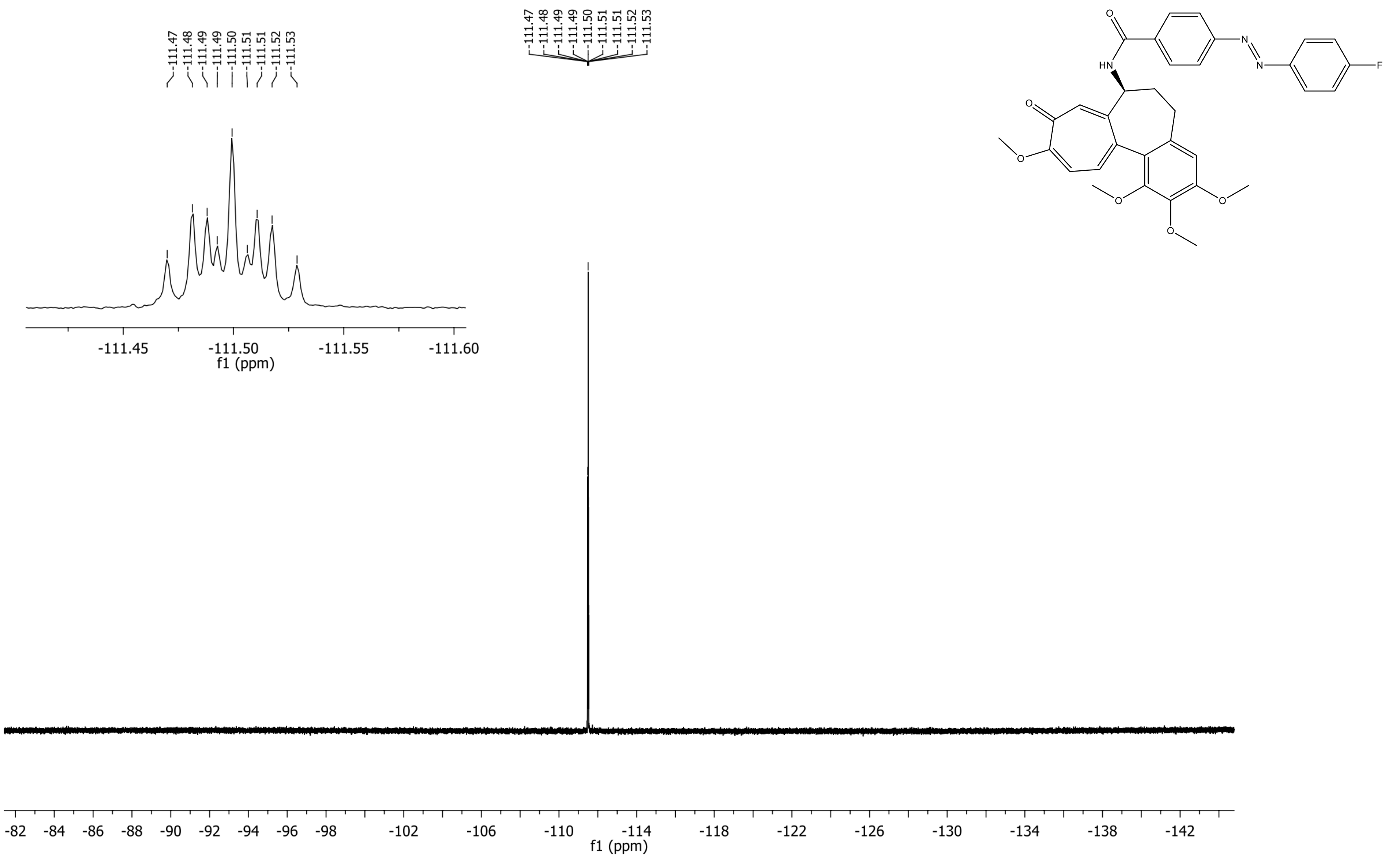

Figure S94. ^{19}F NMR spectra of *p*-**AzoCol4F** ((S,E)-4-((4-fluorophenyl)diazenyl)-N-(1,2,3,10-tetramethoxy-9-oxo-5,6,7,9-tetrahydrobenzo[a]heptalen-7-yl)benzamide).

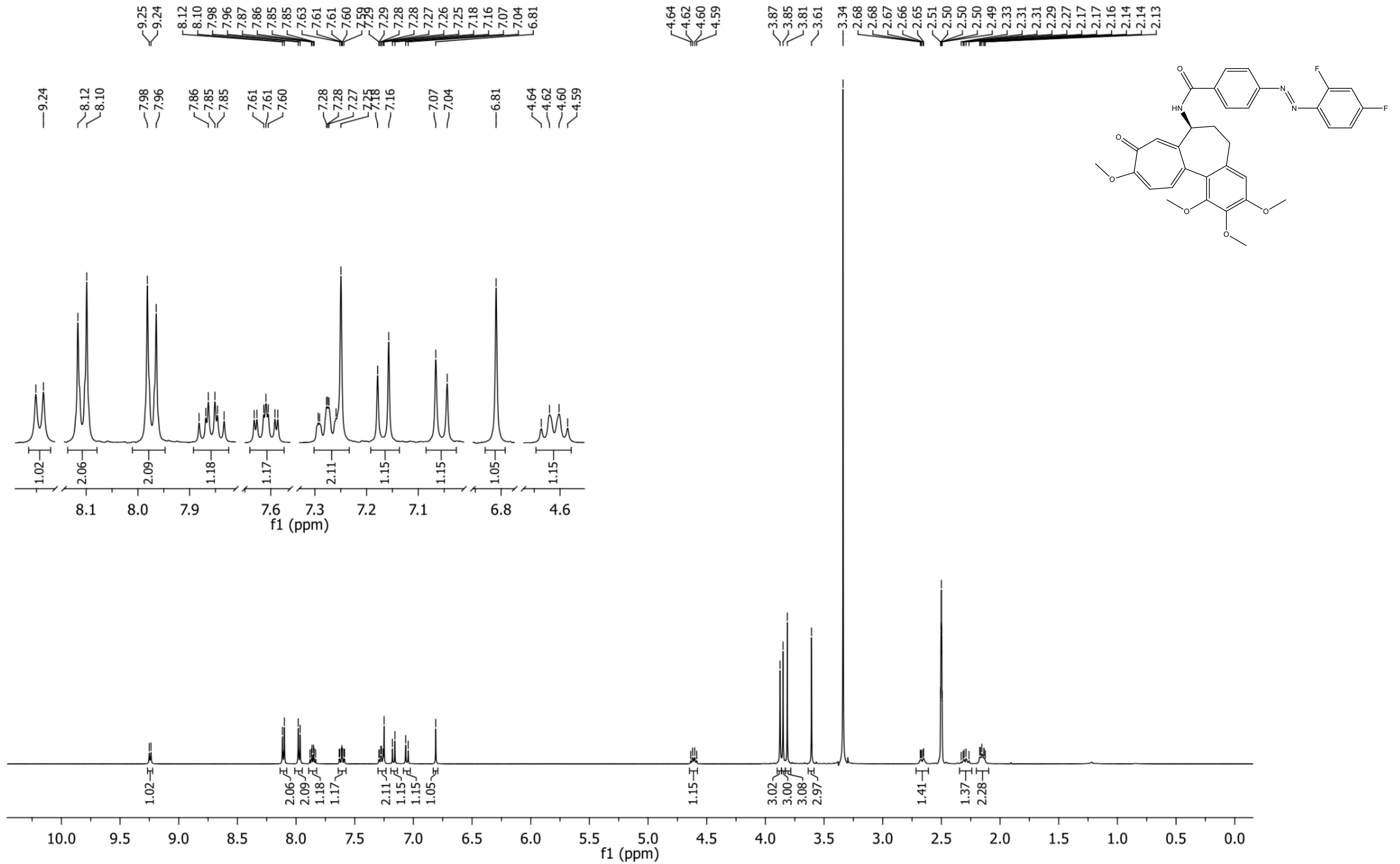

Figure S95. ^{1}H NMR spectra of *p*-**AzoCol24DF** ((S,E)-4-((2,4-difluorophenyl)diazenyl)-N-(1,2,3,10-tetramethoxy-9-oxo-5,6,7,9-tetrahydrobenzo[a]heptalen-7-yl)benzamide).

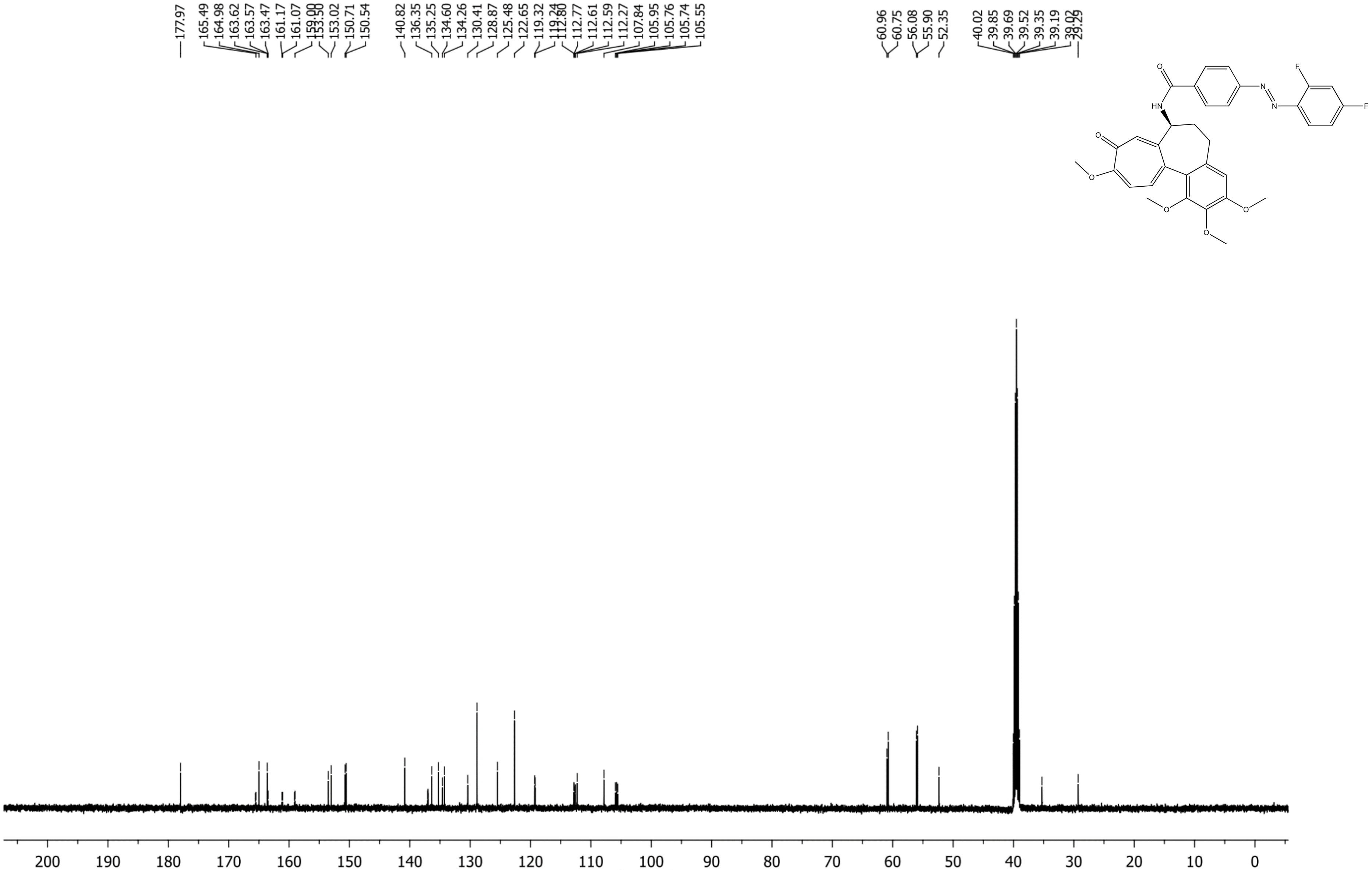

Figure S96. ^{13}C NMR spectra of *p*-**AzoCol24DF** ((S,E)-4-((2,4-difluorophenyl)diazenyl)-N-(1,2,3,10-tetramethoxy-9-oxo-5,6,7,9-tetrahydrobenzo[a]heptalen-7-yl)benzamide).

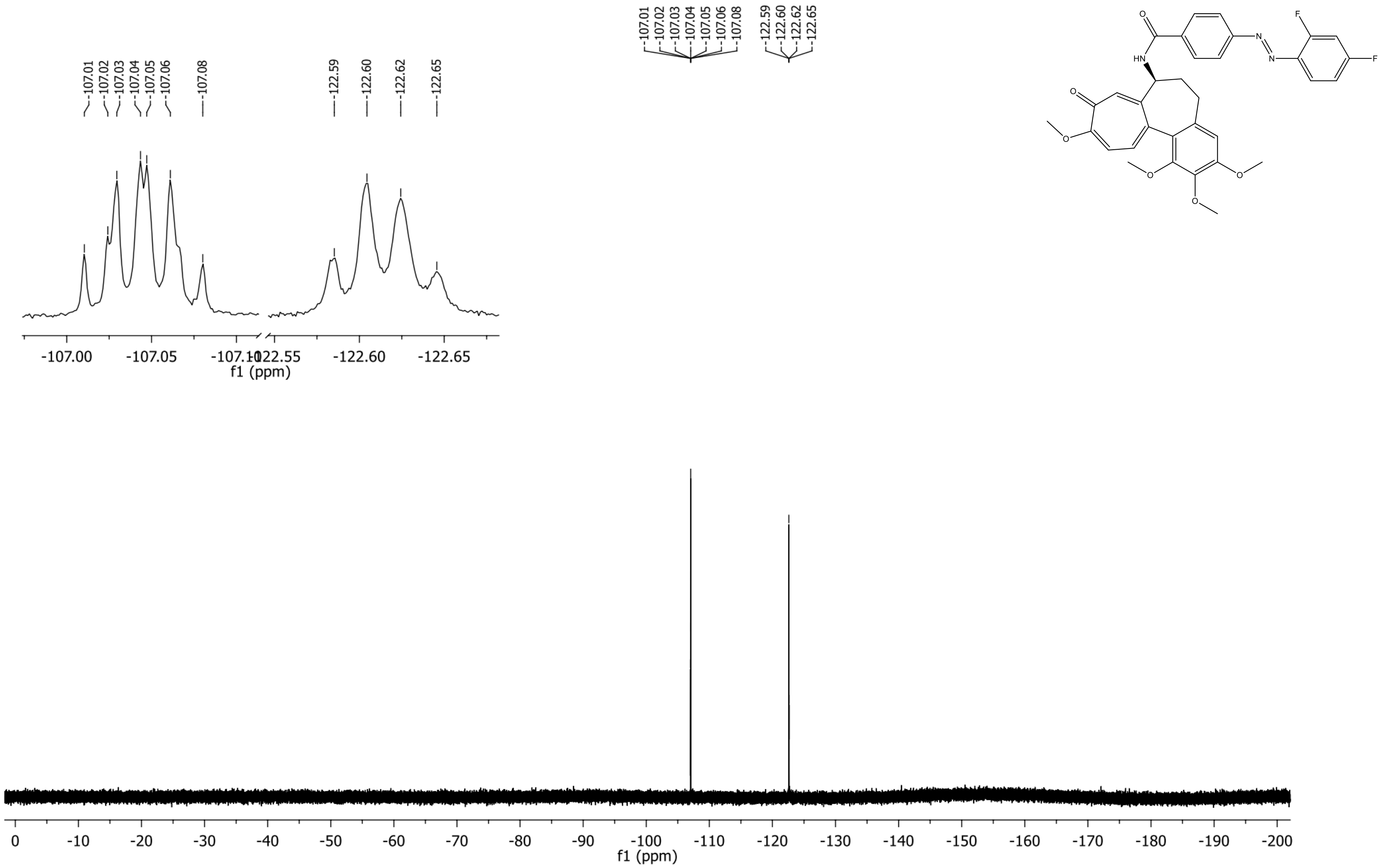

Figure S97. ^{19}F NMR spectra of *p*-**AzoCol24DF** ((S,E)-4-((2,4-difluorophenyl)diazenyl)-N-(1,2,3,10-tetramethoxy-9-oxo-5,6,7,9-tetrahydrobenzo[a]heptalen-7-yl)benzamide).

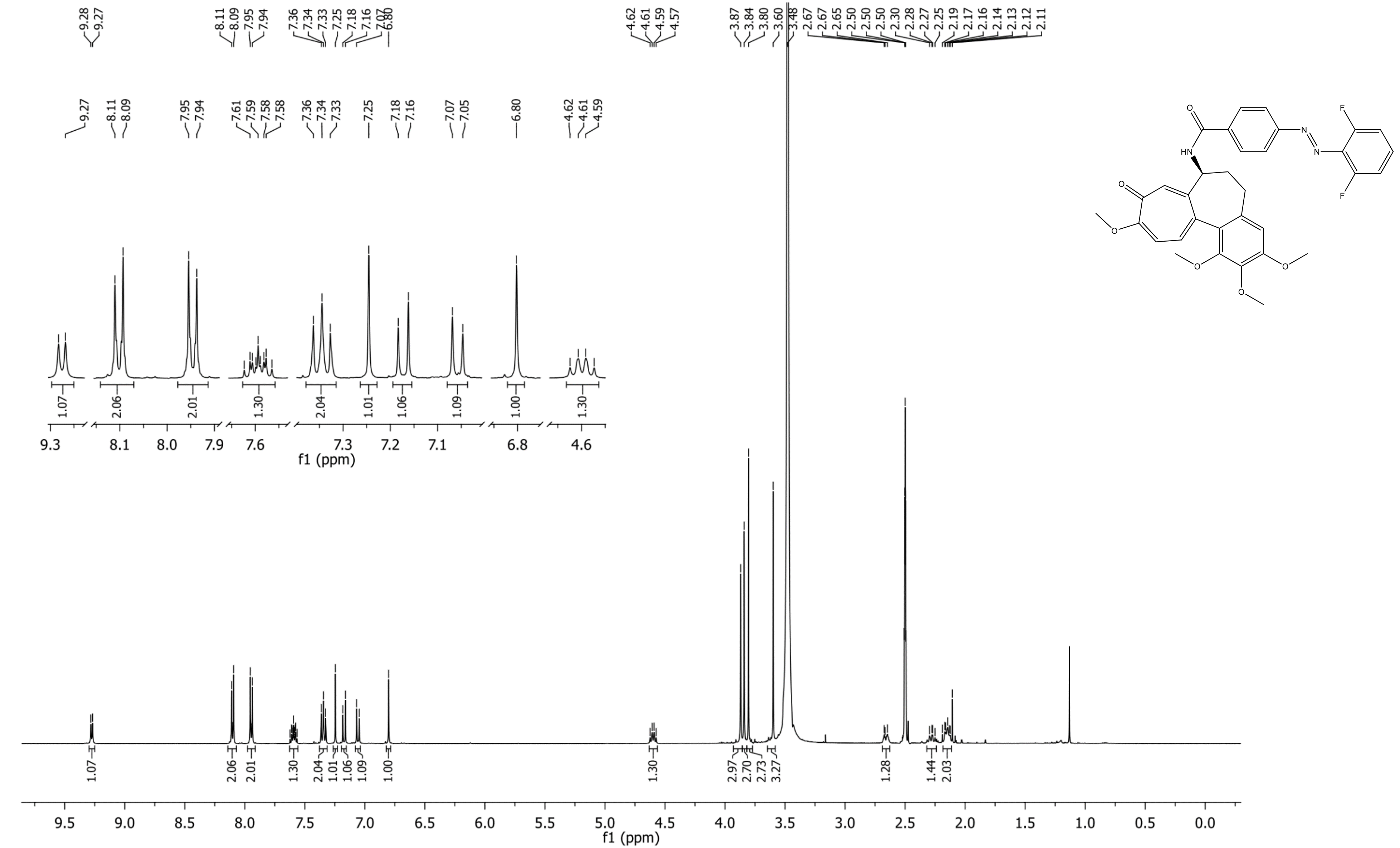

Figure S98. ^{1}H NMR spectra of *p*-**AzoCol26DF** ((S,E)-4-((2,6-difluorophenyl)diazenyl)-N-(1,2,3,10-tetramethoxy-9-oxo-5,6,7,9-tetrahydrobenzo[a]heptalen-7-yl)benzamide).

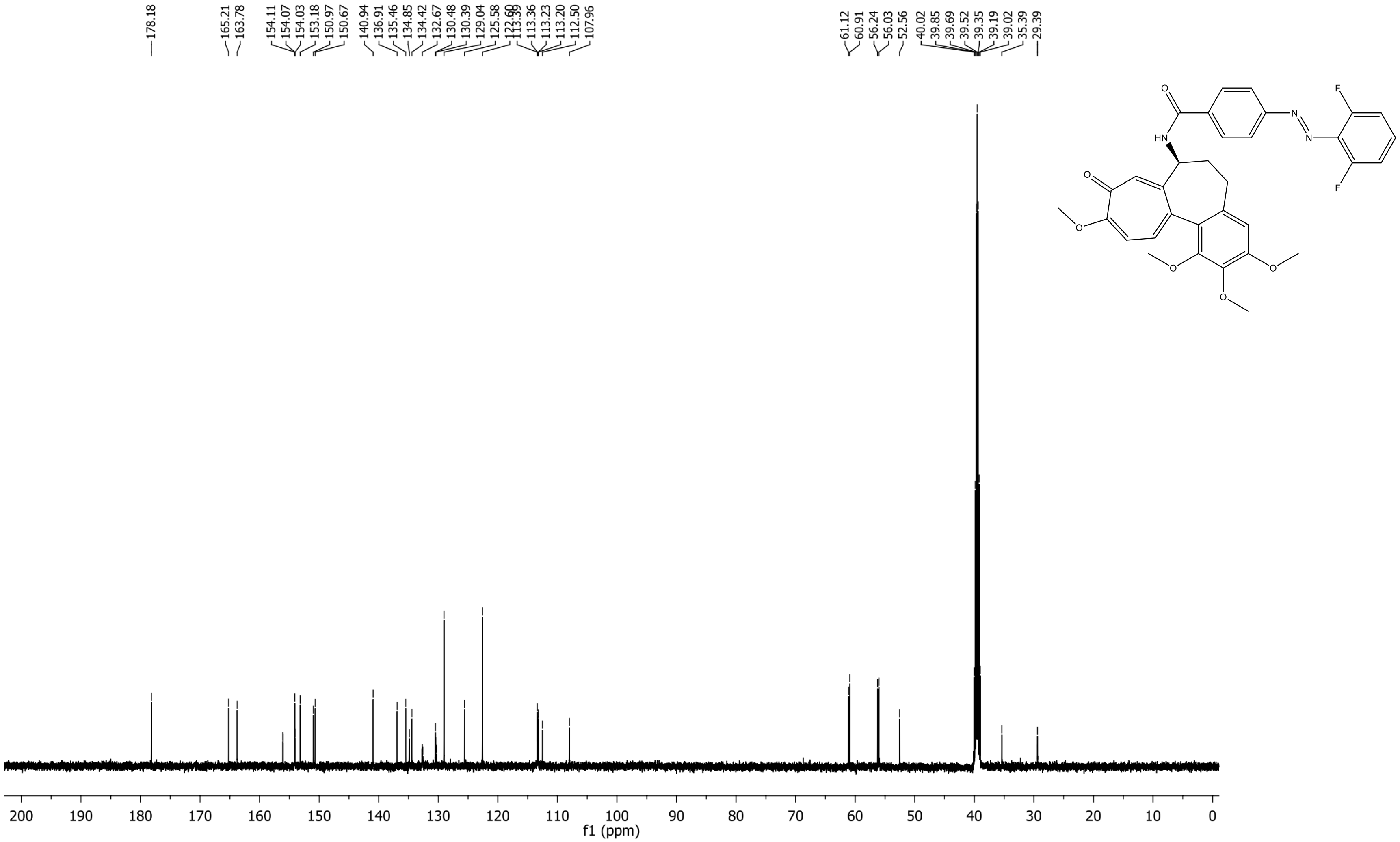

Figure S99. ^{13}C NMR spectra of *p*-**AzoCol26DF** ((S,E)-4-((2,6-difluorophenyl)diazenyl)-N-(1,2,3,10-tetramethoxy-9-oxo-5,6,7,9-tetrahydrobenzo[a]heptalen-7-yl)benzamide).

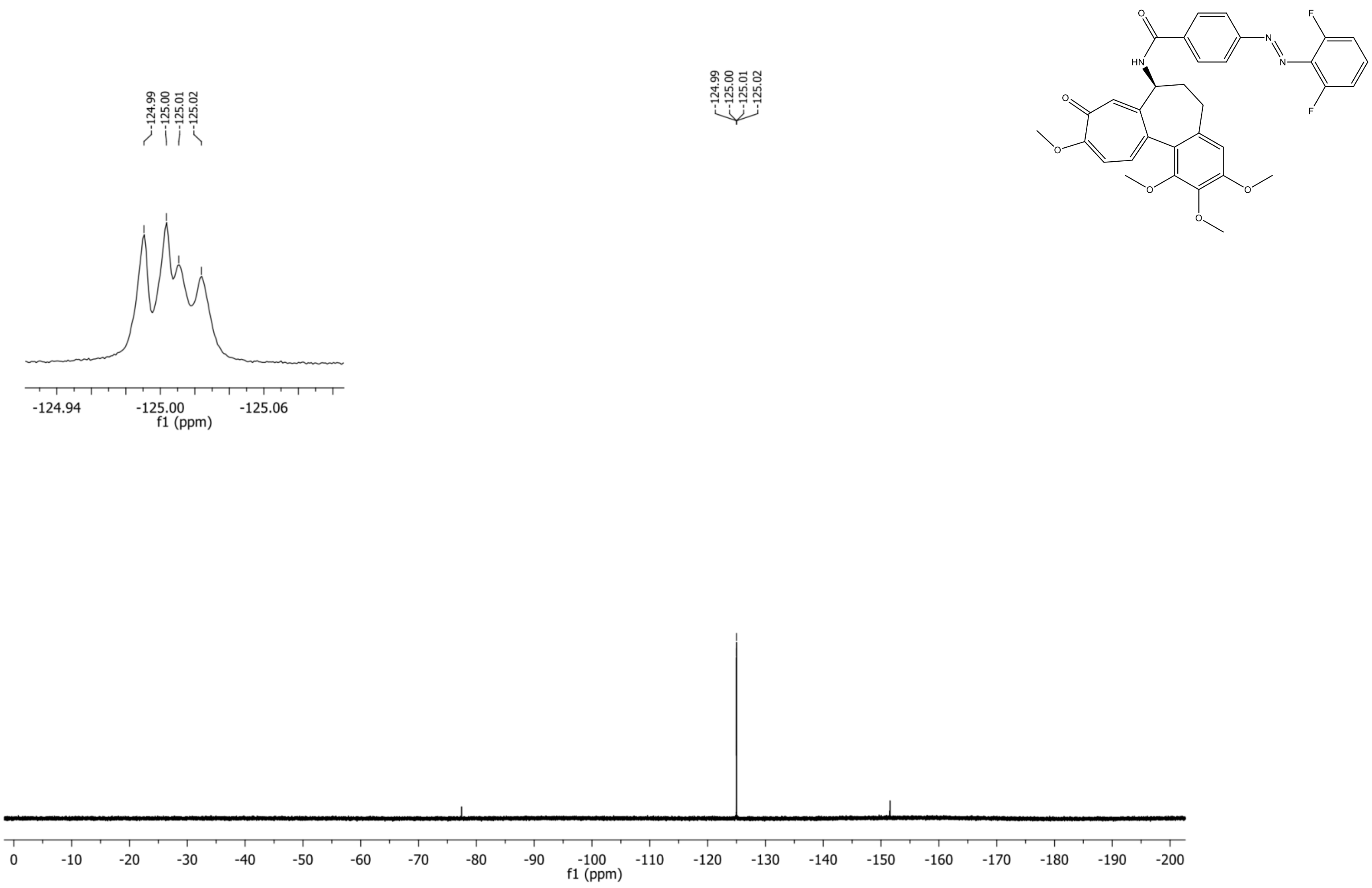

Figure S100. ^{19}F NMR spectra of *p*-**AzoCol26DF** ((S,E)-4-((2,6-difluorophenyl)diazenyl)-N-(1,2,3,10-tetramethoxy-9-oxo-5,6,7,9-tetrahydrobenzo[a]heptalen-7-yl)benzamide).

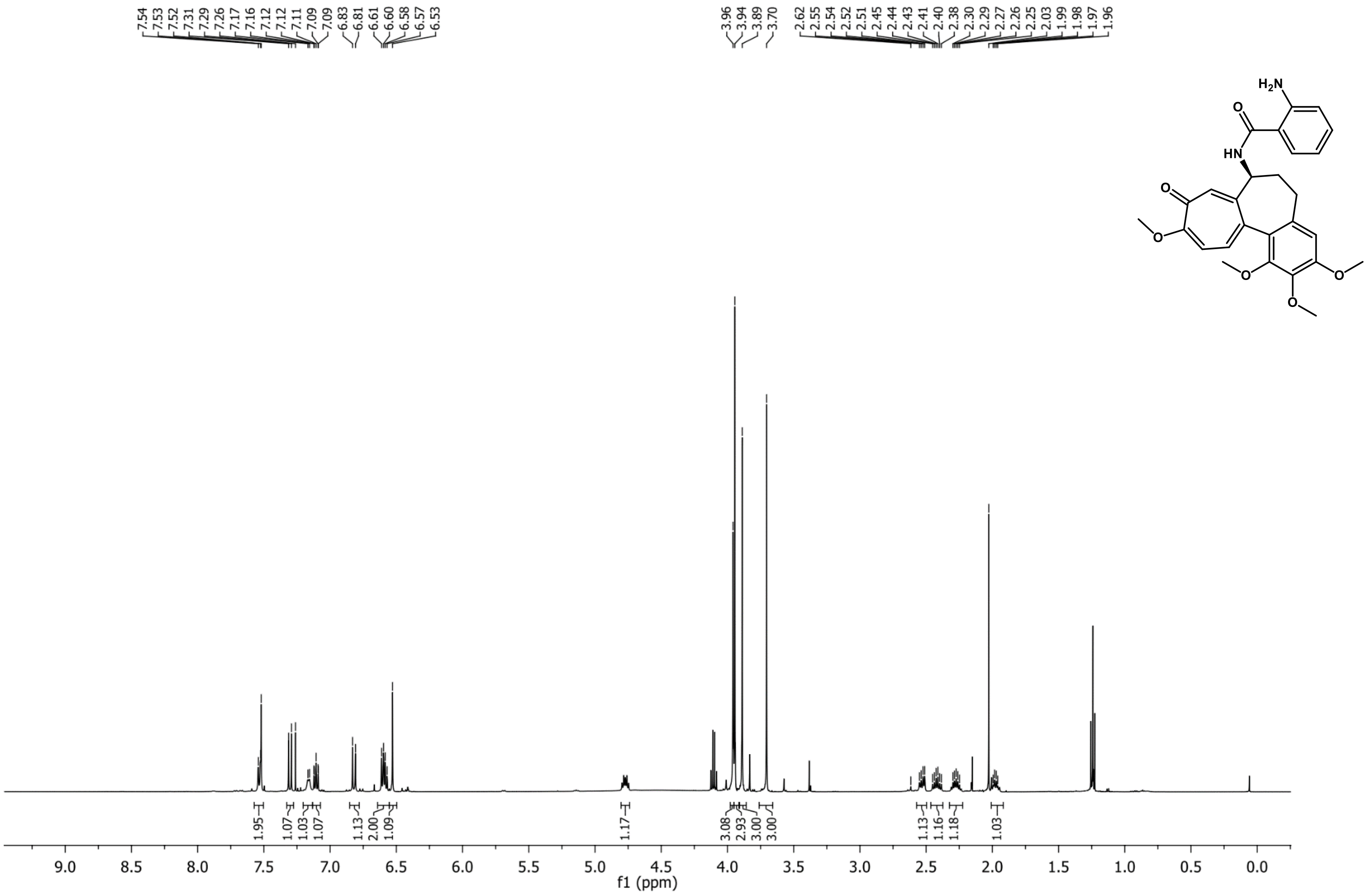

Figure S101. ^{1}H NMR spectra of intermediate **S1 (**(S)-2-amino-N-(1,2,3,10-tetramethoxy-9-oxo-5,6,7,9-tetrahydrobenzo[a]heptalen-7-yl)benzamide).

Computational calculations

The optimum ground-state geometry for *o*-, *m*-, *p*-**3a-d** compounds was calculated using the density functional theory (DFT). In calculation, the B3LYP functional and 6-31G*for basis set was employed and the continuum model (PCM; Gaussian 03W) [1,2] was used to simulate the effects of the solvent. HOMO and LUMO molecular orbitals for all compounds were computed. All the calculations were performed on a server equipped with a 16 quad-core XEON (R) CPU E7310 processor operating at 1.60 GHz. The operating system was Open SUSE 10.3, in DMSO as a solvent. The Avogadro program (version:1.2.0; http://avogadro.cc/) was used to obtain corresponding difference densities and molecular orbitals.

Table S1. Comparison of total energies of investigated model systems ((*E*) and (*Z*) isomers of *o*-, *m*-, *p*-*3a-d*) obtained on B3LYP/ 6-31G* level of theory, and dihedral angle for C-N=N-C and C−C−N−N bounds and N−N bond lengths. The comparison between the (*E*) and (*Z*) configuration shows the energetic impact of the isomerization.

Compound	Total Energy [au]	Δ Total Energy [au]	Δ Total Energy [kJ/mol]	C-N=N-C dihedral angle[°]
(*E*)-*p*-**3d**	-959.787408820	0.016434109	43.38	-179.7
(*Z*)-*p*-**3d**	-959.770974711			10.6
(*E*)-*p*-**3c**	-959.794443470	0.02173095	57.36	-179.8
(*Z*)-*p*-**3c**	-959.772712520			11.3
(*E*)-*p*-**3b**	-860.566168990	0.02186061	57.71	-179.9
(*Z*)-*p*-**3b**	-860.544308380			10.1
(*E*)-*p*-**3a**	-761.332464486	0.021123026	55.76	-179.9
(*Z*)-*p*-**3a**	-761.311341460			10.2
(*E*)-*o*-**3d**	-959.798051057	0.021261267	56.13	-180.0
(*Z*)-*o*-**3d**	-959.776789790			11.6
(*E*)-*o*-**3c**	-959.801368527	0.021508987	56.78	-177.7
(*Z*)-*o*-**3c**	-959.779859540			10.9
(*E*)-*o*-**3b**	-860.573463907	0.030715255	81.08	-180.0

(*Z*)-*o*-**3b**	-860.542748652			13.0
(*E*)-*o*-**3a**	-761.340164832	0.030536116	80.61	-178.0
(*Z*)-*o*-**3a**	-761.309628716			12.7
(*E*)-*m*-**3d**	-959.787663128	0.017419366	45.98	-179.9
(*Z*)-*m*-**3d**	-959.770243762			10.5
(*E*)-*m*-**3c**	-959.794651678	0.021702788	57.29	-179.0
(*Z*)-*m*-**3c**	-959.772948890			9.3
(*E*)-*m*-**3b**	-860.565938396	0.022668602	59.84	-180.0
(*Z*)-*m*-**3b**	-860.543269794			9.8
(*E*)-*m*-**3a**	-761.332222055	0.021959282	57.97	-180.0
(*Z*)-*m*-**3a**	-761.310262773			9.7

Table S2. Energy of HOMO-LUMO orbitals of (*E*) and (*Z*) isomers for *o*-, *m*-, *p*-***3a-d*** compouds culated using Avogadro 1.2.0 programm.

**Compound**	**Energy of HOMO-LUMO orbitals/*eV***		**ΔE[eV]**
	**HOMO**	**LUMO**	
(*E*)-*p*-**3d**	-6.474	-2.801	3.673
(*Z*)-*p*-**3d**	- 6.416	-2.639	3.777
(*E*)-*p*-**3c**	-6.540	-2.801	3.739
(*Z*)-*p*-**3c**	-6.283	- 2.593	3.690
(*E*)-*p*-**3b**	-6.472	- 2.716	3.756
(*Z*)-*p*-**3b**	-6.178	- 2.480	3.698
(*E*)-*p*-**3a**	- 6.487	- 2.705	3.782

(*Z*)-*p*-**3a**	-6. 170	-2.458	3.712
(*E*)-*o*-**3d**	-6.777	- 3.054	3.723
(*Z*)-*o*-**3d**	-6.592	-2.995	3.597
(*E*)-*o*-**3c**	-6.659	-3.060	3.599
(*Z*)-*o*-**3c**	-6.478	-2.933	3.545
(*E*)-*o*-**3b**	-6.625	-2.972	3.653
(*Z*)-*o*-**3b**	-6.121	-2.251	3.870
(*E*)-*o*-**3a**	-6.650	-2.955	3.695
(*Z*)-*o*-**3a**	-6.114	-2.220	3.894
(*E*)-*m*-**3d**	-6.473	-2.661	3.812
(*Z*)-*m*-**3d**	- 6.369	-2.523	3.846
(*E*)-*m*-**3c**	- 6.512	-2.659	3.853
(*Z*)-*m*-**3c**	-6.262	-2.431	3.831
(*E*)-*m*-**3b**	-6.447	-2.566	3.881
(*Z*)-*m*-**3b**	- 6.140	-2.352	3.788
(*E*)-*m*-**3a**	-6.490	-2.555	3.935
(*Z*)-*m*-**3a**	-6.129	-2.313	3.816

Calculated Structures

Optimized Geometries:

- The calculated coordinates of (*E*)-4-((2,6-difluorophenyl)diazenyl)benzoic acid (*E*)-*p*-**3d** (the part of calculated log file) -

Standard orientation:

Center Number	Atomic Number	Atomic Type	Coordinates (Angstroms) X	Y	Z
1	6	0	-2.591099	0.101881	0.006403
2	7	0	-1.209160	0.335379	0.015652
3	7	0	-0.457100	-0.678148	-0.063156
4	6	0	0.926503	-0.357777	-0.041145
5	6	0	1.793934	-1.453624	-0.135718
6	6	0	3.172487	-1.260919	-0.117142
7	6	0	3.699605	0.033232	-0.007888
8	6	0	2.824092	1.132032	0.065525
9	6	0	1.451363	0.944311	0.059370
10	6	0	-3.399487	1.260442	0.027198
11	6	0	-4.785027	1.222019	0.029460
12	6	0	-5.416874	-0.021738	0.015281
13	6	0	-4.670151	-1.201639	-0.001138
14	6	0	-3.284769	-1.131459	-0.006032
15	9	0	-2.791436	2.456217	0.042787
16	9	0	-2.601305	-2.283183	-0.016288
17	6	0	5.168757	0.313495	-0.010592
18	8	0	5.638636	1.386626	-0.327977
19	8	0	6.002421	-0.691366	0.352270
20	1	0	1.367823	-2.447777	-0.227970
21	1	0	3.824120	-2.125352	-0.221446
22	1	0	3.246400	2.129473	0.131229
23	1	0	0.768588	1.783736	0.126661
24	1	0	-5.343498	2.151719	0.042524
25	1	0	-6.501316	-0.074245	0.017284
26	1	0	-5.144137	-2.177651	-0.008743
27	1	0	5.516052	-1.441440	0.738661

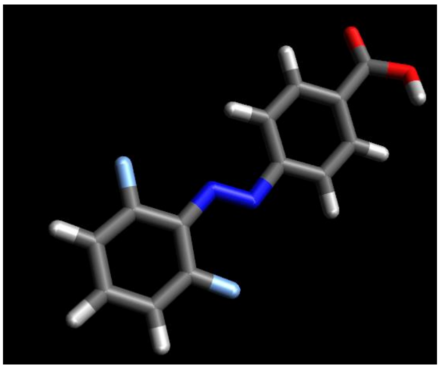

Visualization of calculated geometry of (*E*)-*p*-**3d** .

• The calculated coordinates of (*Z*)-4-((2,6-difluorophenyl)diazenyl)benzoic acid (*Z*)-*p*-***3d*** (the part of calculated log file) -

Standard orientation:

Center Number	Atomic Number	Atomic Type	Coordinates (Angstroms) X	Y	Z
1	6	0	2.227720	-0.428179	0.057590
2	7	0	1.884156	-1.803069	-0.163233
3	7	0	0.705558	-2.219014	-0.153621
4	6	0	-0.436879	-1.361175	-0.142619
5	6	0	-1.474924	-1.684940	0.739569
6	6	0	-2.658690	-0.956891	0.710164
7	6	0	-2.838406	0.069936	-0.229713
8	6	0	-1.801829	0.368453	-1.126080
9	6	0	-0.607598	-0.340780	-1.091102
10	6	0	1.853991	0.302860	1.194237
11	6	0	2.378267	1.556000	1.478053
12	6	0	3.315606	2.108624	0.603891
13	6	0	3.732579	1.409427	-0.531685
14	6	0	3.201670	0.151243	-0.767844
15	9	0	0.978154	-0.252557	2.054125
16	9	0	3.589865	-0.542646	-1.853857
17	6	0	-4.083860	0.894368	-0.309334
18	8	0	-4.117398	1.995449	-0.818555
19	8	0	-5.217180	0.379408	0.225430
20	1	0	-1.334916	-2.490298	1.453949
21	1	0	-3.429900	-1.190833	1.440383
22	1	0	-1.949056	1.161642	-1.851447
23	1	0	0.177306	-0.124804	-1.808639
24	1	0	2.054675	2.073781	2.374912
25	1	0	3.727624	3.091316	0.810635
26	1	0	4.459889	1.819749	-1.224181
27	1	0	-5.115037	-0.561687	0.454187

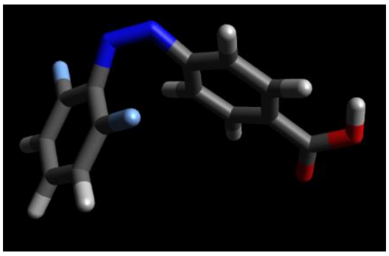

Visualization of calculated geometry of (*Z*)-*p*-***3d*** :

• The calculated coordinates of (*E*)-4-((2,4-difluorophenyl)diazenyl)benzoic acid (*E*)-*p*-**3c** (the part of calculated log file) -

Standard orientation:

Center Number	Atomic Number	Atomic Type	Coordinates (Angstroms) X	Y	Z
1	6	0	-2.345200	0.004198	0.002537
2	7	0	-0.968911	0.297161	0.014265
3	7	0	-0.212490	-0.713104	-0.038371
4	6	0	1.171725	-0.405596	-0.029361
5	6	0	2.032020	-1.510607	-0.068954
6	6	0	3.411633	-1.326473	-0.057582
7	6	0	3.947948	-0.032236	-0.011564
8	6	0	3.079581	1.074558	0.005468
9	6	0	1.705591	0.896270	0.006538
10	6	0	-3.223012	1.101488	0.063477

11	6	0	-4.603004	0.949175	0.058181
12	6	0	-5.104603	-0.344625	-0.010634
13	6	0	-4.280722	-1.470577	-0.073009
14	6	0	-2.906545	-1.286214	-0.065958
15	9	0	-6.437474	-0.515555	-0.017071
16	9	0	-2.720468	2.342739	0.128932
17	6	0	5.418743	0.238022	-0.026359
18	8	0	5.895562	1.293204	-0.390232
19	8	0	6.246150	-0.755495	0.379950
20	1	0	1.600000	-2.505582	-0.112975
21	1	0	4.056908	-2.199818	-0.117780
22	1	0	3.508519	2.071145	0.021937
23	1	0	1.029297	1.743192	0.031310
24	1	0	-5.260308	1.809956	0.105815
25	1	0	-4.724972	-2.459050	-0.125575
26	1	0	-2.231943	-2.133664	-0.113387
27	1	0	5.755157	-1.485209	0.798241

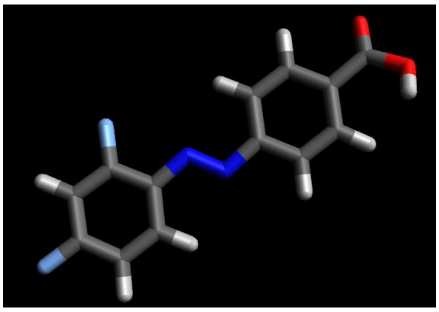

Visualization of calculated geometry of (*E*)-*p*-**3c**:

• The calculated coordinates of (*Z*)-4-((2,4-difluorophenyl)diazenyl)benzoic acid (*Z*)-*p*-**3c** (the part of calculated log file) -

Standard orientation:

Center Number	Atomic Number	Atomic Type	Coordinates (Angstroms) X	Y	Z
1	6	0	2.000672	-0.771598	-0.240259
2	7	0	1.513022	-2.115305	-0.284695
3	7	0	0.301272	-2.406452	-0.389059
4	6	0	-0.768349	-1.464380	-0.246486
5	6	0	-0.908323	-0.676952	0.905504
6	6	0	-2.056811	0.088368	1.079801
7	6	0	-3.067140	0.094188	0.106369
8	6	0	-2.912781	-0.693049	-1.045268
9	6	0	-1.790854	-1.495080	-1.206238
10	6	0	3.110740	-0.529525	0.583657
11	6	0	3.771750	0.688947	0.602481
12	6	0	3.327083	1.672802	-0.274634
13	6	0	2.257947	1.472268	-1.143943
14	6	0	1.596501	0.248605	-1.117639
15	9	0	3.962527	2.857550	-0.284197
16	9	0	3.523793	-1.503813	1.410907
17	6	0	-4.301941	0.929113	0.218035
18	8	0	-4.971649	1.252536	-0.741182
19	8	0	-4.673355	1.345939	1.452979
20	1	0	-0.131776	-0.674422	1.663429
21	1	0	-2.133752	0.707412	1.970742
22	1	0	-3.694783	-0.680025	-1.797079
23	1	0	-1.686307	-2.139041	-2.074607
24	1	0	4.608797	0.860372	1.269994
25	1	0	1.962681	2.260266	-1.828464
26	1	0	0.777529	0.069233	-1.805060
27	1	0	-4.170256	0.894826	2.154279

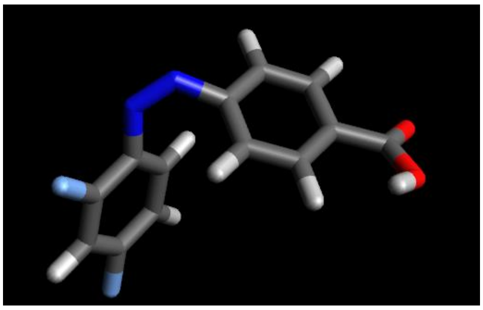

Visualization of calculated geometry of (*Z*)-*p*-**3c.**

• The calculated coordinates of (*E*)-4-((4-fluorophenyl)diazenyl)benzoic acid (*E*)-*p*-**3b** (the part of calculated log file) -

Standard orientation:

Center Number	Atomic Number	Atomic Type	Coordinates (Angstroms) X	Y	Z
1	6	0	-2.524189	0.219205	0.017574
2	7	0	-1.134363	0.470944	0.031540
3	7	0	-0.402523	-0.555128	-0.042052
4	6	0	0.990162	-0.284467	-0.029093
5	6	0	1.820661	-1.410712	-0.097093
6	6	0	3.204778	-1.264757	-0.084633
7	6	0	3.776544	0.012865	-0.008430
8	6	0	2.938347	1.141741	0.037332
9	6	0	1.559892	1.000770	0.037040
10	6	0	-3.341666	1.357183	0.098541
11	6	0	-4.728345	1.235779	0.092941
12	6	0	-5.276083	-0.039481	0.005411
13	6	0	-4.490199	-1.190811	-0.076226
14	6	0	-3.108991	-1.059110	-0.070126
15	9	0	-6.616683	-0.173165	-0.001032
16	6	0	5.253848	0.243639	-0.019313
17	8	0	5.759137	1.294628	-0.356212
18	8	0	6.054761	-0.782151	0.359539
19	1	0	1.361640	-2.392260	-0.163992
20	1	0	3.825571	-2.153815	-0.167869
21	1	0	3.394115	2.125711	0.077350
22	1	0	0.907470	1.865340	0.083841
23	1	0	-2.868122	2.331947	0.165291
24	1	0	-5.379531	2.101630	0.154360
25	1	0	-4.970420	-2.162163	-0.142706
26	1	0	-2.466533	-1.930288	-0.132298
27	1	0	5.544422	-1.509216	0.758732

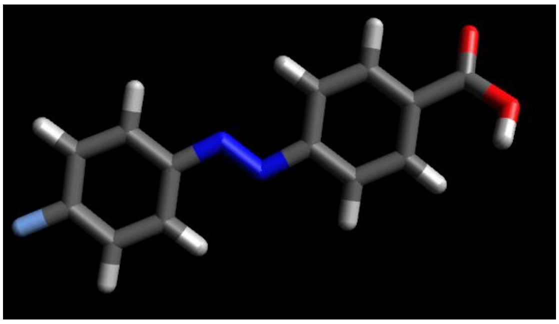

Visualization of calculated geometry of (*E*)-*p*-**3b**.

• The calculated coordinates of (*Z*)-4-((4-fluorophenyl)diazenyl)benzoic acid (*Z*)-*p*-**3b** (the part of calculated log file) -

Standard orientation:

Center Number	Atomic Number	Atomic Type	Coordinates (Angstroms) X	Y	Z
1	6	0	-2.213984	0.983167	0.030257
2	7	0	-1.648260	2.292861	0.149867
3	7	0	-0.425428	2.539913	0.054753
4	6	0	0.601983	1.542106	0.020586
5	6	0	0.748702	0.602045	1.051274
6	6	0	1.867069	-0.224829	1.074307
7	6	0	2.840151	-0.141145	0.066781
8	6	0	2.679882	0.799293	-0.962576
9	6	0	1.590374	1.659966	-0.968154
10	6	0	-3.325200	0.728824	0.848821
11	6	0	-4.005394	-0.481552	0.759148
12	6	0	-3.596761	-1.401048	-0.202139
13	6	0	-2.535229	-1.151008	-1.067725
14	6	0	-1.833754	0.044333	-0.943770
15	9	0	-4.265107	-2.565726	-0.314811
16	6	0	4.035674	-1.035305	0.016870
17	8	0	4.638771	-1.282228	-1.007197
18	8	0	4.446848	-1.601622	1.178486
19	1	0	-0.001471	0.527676	1.831916
20	1	0	1.947928	-0.962731	1.869304
21	1	0	3.431316	0.854955	-1.743205
22	1	0	1.484180	2.418257	-1.738527
23	1	0	-3.636902	1.485986	1.562145
24	1	0	-4.850600	-0.708621	1.400821
25	1	0	-2.271560	-1.884798	-1.822725
26	1	0	-1.013220	0.254447	-1.620418
27	1	0	4.006084	-1.201336	1.949261

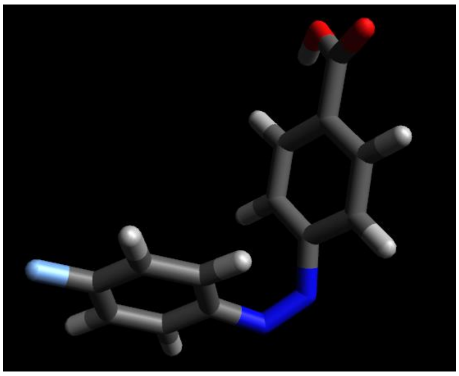

Visualization of calculated geometry of (*Z*)-*p*-**3b**.

• The calculated coordinates of (*E*)-4-((phenyl)diazenyl)benzoic acid (*E*)-*p*-**3a** (the part of calculated log file) -

Standard orientation:

Center Number	Atomic Number	Atomic Type	Coordinates (Angstroms) X	Y	Z
1	6	0	2.977072	-0.186674	0.017449
2	7	0	1.585863	-0.447387	0.031772
3	7	0	0.848088	0.573696	-0.042754
4	6	0	-0.543451	0.293741	-0.029523
5	6	0	-1.381329	1.414420	-0.096467
6	6	0	-2.764498	1.259257	-0.083673

7	6	0	-3.327631	-0.022241	-0.008461
8	6	0	-2.481969	-1.145598	0.036144
9	6	0	-1.104465	-0.995244	0.035664
10	6	0	3.797950	-1.320945	0.100442
11	6	0	5.184955	-1.183525	0.094195
12	6	0	5.755538	0.087878	0.005207
13	6	0	4.936870	1.223072	-0.077734
14	6	0	3.553423	1.093933	-0.072298
15	6	0	-4.803397	-0.262545	-0.019205
16	8	0	-5.302076	-1.316104	-0.357912
17	8	0	-5.610693	0.757474	0.361815
18	1	0	-0.928768	2.399003	-0.162839
19	1	0	-3.391378	2.144126	-0.165929
20	1	0	-2.931145	-2.132628	0.075305
21	1	0	-0.445851	-1.855148	0.081710
22	1	0	3.325741	-2.296686	0.168612
23	1	0	5.818166	-2.063893	0.158416
24	1	0	6.836535	0.199206	0.000140
25	1	0	5.386472	2.210067	-0.146827
26	1	0	2.905065	1.960884	-0.135929
27	1	0	-5.104759	1.486782	0.762560

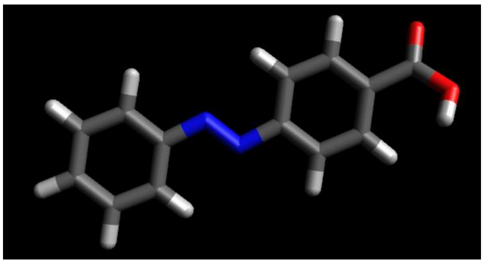

Visualization of calculated geometry of (*E*)-*p*-**3a** :

- The calculated coordinates of (*Z*)-*p*-**3a** (the part of calculated log file) -

Standard orientation:

Center Number	Atomic Number	Atomic Type	Coordinates (Angstroms) X	Y	Z
1	6	0	2.581126	0.514092	-0.027508
2	7	0	2.175533	1.865608	-0.280487
3	7	0	0.988976	2.257754	-0.234814
4	6	0	-0.145849	1.388103	-0.131725
5	6	0	-0.377055	0.361278	-1.058768
6	6	0	-1.582779	-0.331820	-1.031650
7	6	0	-2.563147	-0.025672	-0.075558
8	6	0	-2.318224	0.999671	0.851006
9	6	0	-1.136346	1.726482	0.802248
10	6	0	3.616083	0.024381	-0.836912
11	6	0	4.133339	-1.248470	-0.607729
12	6	0	3.665697	-2.010559	0.466553
13	6	0	2.673259	-1.497378	1.306674
14	6	0	2.119676	-0.242417	1.060988
15	6	0	-3.856708	-0.765607	0.029153
16	8	0	-4.516756	-0.808534	1.047071
17	8	0	-4.296408	-1.426376	-1.070374
18	1	0	0.377432	0.113738	-1.798065
19	1	0	-1.730998	-1.138699	-1.745789
20	1	0	-3.076716	1.227635	1.592501

21	1	0	-0.960778	2.550244	1.488067
22	1	0	3.991611	0.646319	-1.644594
23	1	0	4.915521	-1.636950	-1.253913
24	1	0	4.085357	-2.993856	0.659802
25	1	0	2.327206	-2.076917	2.158116
26	1	0	1.356805	0.156820	1.720736
27	1	0	-3.786134	-1.185087	-1.863953

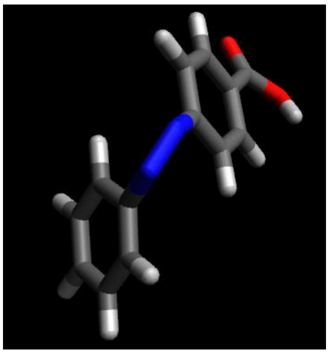

Visualization of calculated geometry of (*Z*)-*p*-**3a**.

- The calculated coordinates of (*E*)-*m*-**3d** (the part of calculated log file) -

Standard orientation:

Center Number	Atomic Number	Atomic Type	Coordinates (Angstroms) X	Y	Z
1	6	0	2.455376	0.040172	-0.005531
2	7	0	1.168480	0.598807	0.015428
3	7	0	0.196408	-0.207755	-0.020079
4	6	0	-1.072819	0.429723	0.007056
5	6	0	-2.170828	-0.437712	-0.009888
6	6	0	-3.474901	0.069148	0.005196
7	6	0	-3.671704	1.457142	0.020242
8	6	0	-2.576651	2.322940	0.048322
9	6	0	-1.279548	1.819262	0.042481
10	6	0	3.518763	0.969292	0.019951
11	6	0	4.854374	0.598364	0.007640
12	6	0	5.168282	-0.760381	-0.030718
13	6	0	4.159143	-1.725122	-0.056410
14	6	0	2.831416	-1.322986	-0.044051
15	9	0	3.216936	2.276258	0.057373
16	9	0	1.890338	-2.276132	-0.067790
17	6	0	-4.687515	-0.807676	-0.036332
18	8	0	-5.776189	-0.415850	-0.402137
19	8	0	-4.545144	-2.098151	0.349012
20	1	0	-1.960229	-1.503099	-0.063960
21	1	0	-4.686404	1.841026	0.013592
22	1	0	-2.739481	3.396771	0.071404
23	1	0	-0.417672	2.477206	0.058962
24	1	0	5.620146	1.366190	0.028220
25	1	0	6.208161	-1.072316	-0.040625
26	1	0	4.383628	-2.786276	-0.085812
27	1	0	-3.677500	-2.258975	0.761792

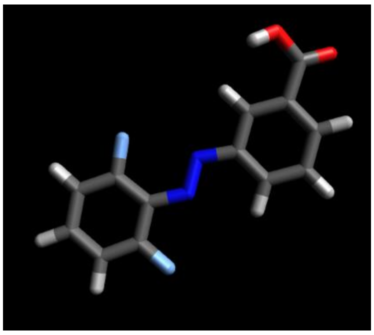

Visualization of calculated geometry of (*E*)-*m*-**3d** :

- The calculated coordinates of (*Z*)-*m*-**3d** (the part of calculated log file) -

Standard orientation:

Center Number	Atomic Number	Atomic Type	Coordinates (Angstroms) X	Y	Z
1	6	0	2.120729	-0.194929	-0.521019
2	7	0	1.498430	-1.172791	-1.364615
3	7	0	0.276561	-1.436347	-1.327373
4	6	0	-0.608941	-0.963141	-0.307586
5	6	0	-0.328359	-1.105157	1.060118
6	6	0	-1.292889	-0.747927	2.000088
7	6	0	-2.531407	-0.254354	1.589923
8	6	0	-2.823348	-0.137504	0.222676
9	6	0	-1.861264	-0.505713	-0.722370
10	6	0	3.372068	-0.504306	0.032492
11	6	0	4.140311	0.424091	0.717233
12	6	0	3.672919	1.737042	0.815427
13	6	0	2.455771	2.105307	0.240256
14	6	0	1.705806	1.141225	-0.418359
15	9	0	3.808651	-1.772216	-0.081713
16	9	0	0.550661	1.501950	-1.009941
17	6	0	-4.127408	0.390133	-0.292481
18	8	0	-4.258911	0.855973	-1.404793
19	8	0	-5.196971	0.343871	0.536379
20	1	0	0.623499	-1.511819	1.384593
21	1	0	-1.077332	-0.854429	3.058982
22	1	0	-3.251026	0.051115	2.345570
23	1	0	-2.087470	-0.426729	-1.780195
24	1	0	5.085428	0.116582	1.151980
25	1	0	4.265494	2.480289	1.339413
26	1	0	2.084265	3.123661	0.285884
27	1	0	-5.018463	-0.189648	1.331506

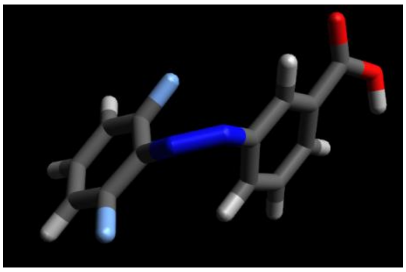

Visualization of calculated geometry of (*Z*)-*m*-**3d** :

- The calculated coordinates of (*E*)-*m*-**3c** (the part of calculated log file) -

Standard orientation:

Center Number	Atomic Number	Atomic Type	Coordinates (Angstroms) X	Y	Z
1	6	0	2.187656	0.082051	-0.003156

2	7	0	0.898354	0.648939	0.018389
3	7	0	-0.046627	-0.187126	-0.014586
4	6	0	-1.341609	0.391527	0.006826
5	6	0	-2.401982	-0.521800	-0.006762
6	6	0	-3.726247	-0.070494	0.001709
7	6	0	-3.981810	1.307949	0.007381
8	6	0	-2.924500	2.219452	0.032530
9	6	0	-1.607210	1.771344	0.032758
10	6	0	3.270487	0.978215	0.033305
11	6	0	4.590788	0.548671	0.017091
12	6	0	4.818817	-0.820653	-0.037224
13	6	0	3.782757	-1.755962	-0.075222
14	6	0	2.474570	-1.295850	-0.057831
15	9	0	6.089464	-1.258882	-0.053729
16	9	0	3.031921	2.296954	0.085713
17	6	0	-4.900553	-0.998071	-0.038448
18	8	0	-6.003364	-0.654422	-0.410181
19	8	0	-4.705264	-2.279232	0.354724
20	1	0	-2.147108	-1.577914	-0.053465
21	1	0	-5.011801	1.648521	-0.004494
22	1	0	-3.132581	3.285553	0.048188
23	1	0	-0.774451	2.465776	0.046578
24	1	0	5.409366	1.258848	0.045928
25	1	0	4.015939	-2.814774	-0.117412
26	1	0	1.641610	-1.989366	-0.086083
27	1	0	-3.833496	-2.401032	0.772203

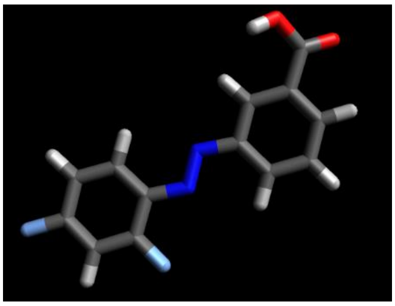

Visualization of calculated geometry of (*E*)-*m*-**3c**.

- The calculated coordinates of (*Z*)-*m*-**3c** (the part of calculated log file) -

Standard orientation:

Center Number	Atomic Number	Atomic Type	Coordinates (Angstroms) X	Y	Z
1	6	0	1.677085	-0.877702	-0.729678
2	7	0	1.029265	-1.948996	-1.429851
3	7	0	-0.166134	-2.271389	-1.250449
4	6	0	-1.090957	-1.502413	-0.472785
5	6	0	-1.926127	-2.206241	0.406717
6	6	0	-2.920733	-1.530569	1.106703
7	6	0	-3.134155	-0.168759	0.884970
8	6	0	-2.339309	0.525355	-0.037638
9	6	0	-1.300489	-0.140248	-0.700653
10	6	0	1.693580	-0.748295	0.666651
11	6	0	2.459385	0.212508	1.310797
12	6	0	3.229455	1.061592	0.523818

13	6	0	3.272663	0.957445	-0.863870
14	6	0	2.516156	-0.036361	-1.475900
15	9	0	3.971537	2.003954	1.133532
16	9	0	0.973274	-1.602038	1.418648
17	6	0	-2.517993	1.982157	-0.337152
18	8	0	-1.645841	2.669331	-0.825741
19	8	0	-3.712968	2.544933	-0.036937
20	1	0	-1.771121	-3.272352	0.544006
21	1	0	-3.542045	-2.066142	1.818142
22	1	0	-3.910760	0.340164	1.450911
23	1	0	-0.689207	0.412653	-1.404413
24	1	0	2.459130	0.288205	2.392561
25	1	0	3.901021	1.629172	-1.438701
26	1	0	2.548504	-0.166544	-2.553572
27	1	0	-4.381632	1.875366	0.193082

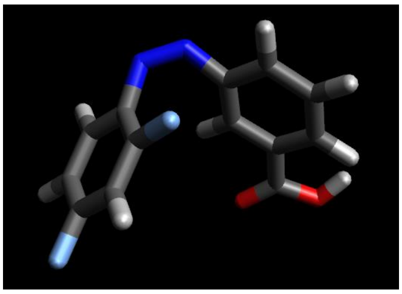

Visualization of calculated geometry of (*Z*)-*m*-**3c** :

- The calculated coordinates of (*E*)-*m*-**3b** (the part of calculated log file) -

Standard orientation:

Center Number	Atomic Number	Atomic Type	Coordinates (Angstroms) X	Y	Z
1	6	0	2.384942	0.311093	0.000052
2	7	0	1.069316	0.829128	0.000586
3	7	0	0.152808	-0.037435	-0.000606
4	6	0	-1.162883	0.495074	-0.000797
5	6	0	-2.187883	-0.457497	-0.000380
6	6	0	-3.530057	-0.061909	-0.000081
7	6	0	-3.834317	1.307544	-0.000474
8	6	0	-2.813782	2.259686	-0.000964
9	6	0	-1.480108	1.863699	-0.001095
10	6	0	3.408768	1.270491	0.001729
11	6	0	4.745460	0.881237	0.001741
12	6	0	5.034111	-0.478993	-0.000193
13	6	0	4.038459	-1.457649	-0.001639
14	6	0	2.709090	-1.059141	-0.001564
15	9	0	6.323467	-0.871195	0.000359
16	6	0	-4.685603	-1.017205	0.000699
17	8	0	-5.842272	-0.647211	0.000704
18	8	0	-4.429455	-2.345128	0.001291
19	1	0	-1.873595	-1.499100	-0.000032
20	1	0	-4.876669	1.606813	-0.000296
21	1	0	-3.064020	3.316830	-0.001305
22	1	0	-0.674583	2.589722	-0.001161
23	1	0	3.134928	2.321140	0.003418
24	1	0	5.553146	1.605907	0.002744
25	1	0	4.319357	-2.506301	-0.003220
26	1	0	1.909155	-1.791046	-0.002396
27	1	0	-3.477165	-2.544932	0.000995

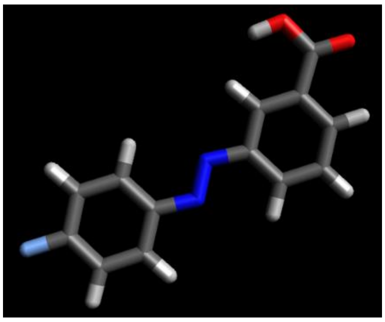

Visualization of calculated geometry of (*E*)-*m*-**3b** :

- The calculated coordinates of (*Z*)-*m*-**3b** (the part of calculated log file) -
-

Standard orientation:

Center Number	Atomic Number	Atomic Type	Coordinates (Angstroms) X	Y	Z
1	6	0	1.768658	-1.032576	-0.512508
2	7	0	1.101527	-2.133757	-1.141554
3	7	0	-0.099668	-2.428353	-0.953253
4	6	0	-1.030173	-1.593655	-0.244041
5	6	0	-1.853212	-2.211156	0.708533
6	6	0	-2.860077	-1.479743	1.333739
7	6	0	-3.099261	-0.155381	0.964604
8	6	0	-2.316844	0.446476	-0.031712
9	6	0	-1.266631	-0.268263	-0.619238
10	6	0	1.654878	-0.724815	0.853472
11	6	0	2.454633	0.268140	1.411365
12	6	0	3.344774	0.955115	0.590682
13	6	0	3.488929	0.659606	-0.761338
14	6	0	2.715782	-0.363187	-1.302076
15	9	0	4.107804	1.926803	1.130379
16	6	0	-2.523494	1.858504	-0.487761
17	8	0	-1.664804	2.507155	-1.047553
18	8	0	-3.731132	2.424840	-0.251051
19	1	0	-1.683675	-3.254432	0.959720
20	1	0	-3.470318	-1.945782	2.101573
21	1	0	-3.884105	0.399341	1.473258
22	1	0	-0.659423	0.215639	-1.375876
23	1	0	0.966272	-1.274116	1.485468
24	1	0	2.396811	0.512909	2.467190
25	1	0	4.208597	1.207081	-1.361375
26	1	0	2.827089	-0.646767	-2.344475
27	1	0	-4.384792	1.769323	0.051263

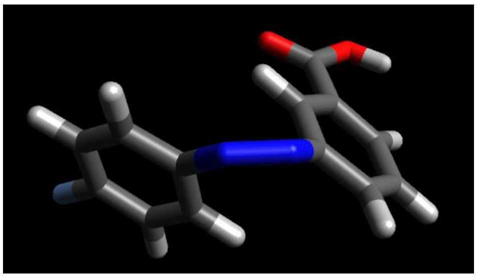

Visualization of calculated geometry of (*Z*)-*m*-**3b** :

- The calculated coordinates of (*E*)-*m*-**3a** (the part of calculated log file) -

Standard orientation:

Center Number	Atomic Number	Atomic Type	Coordinates (Angstroms) X	Y	Z
1	6	0	2.823798	0.145903	0.000681
2	7	0	1.525306	0.712499	0.002893

3	7	0	0.577958	-0.119767	-0.004414
4	6	0	-0.717580	0.461333	-0.003169
5	6	0	-1.777247	-0.452411	-0.001538
6	6	0	-3.103783	-0.007068	-0.000528
7	6	0	-3.356909	1.372777	-0.002340
8	6	0	-2.301677	2.286305	-0.004893
9	6	0	-0.983731	1.840726	-0.005002
10	6	0	3.878946	1.069513	0.012824
11	6	0	5.199936	0.624225	0.012734
12	6	0	5.470355	-0.745621	0.000087
13	6	0	4.416903	-1.670551	-0.012729
14	6	0	3.097579	-1.233993	-0.012440
15	6	0	-4.294283	-0.918688	0.002326
16	8	0	-5.436393	-0.506092	-0.006495
17	8	0	-4.088064	-2.255104	0.017724
18	1	0	-1.501784	-1.504945	-0.001819
19	1	0	-4.387480	1.710310	-0.002096
20	1	0	-2.512300	3.352050	-0.005946
21	1	0	-0.151234	2.535675	-0.007789
22	1	0	3.638499	2.128646	0.021708
23	1	0	6.014973	1.342411	0.022365
24	1	0	6.498771	-1.096855	0.000989
25	1	0	4.632644	-2.735665	-0.023241
26	1	0	2.270797	-1.935705	-0.022205
27	1	0	-3.143928	-2.490180	0.022771

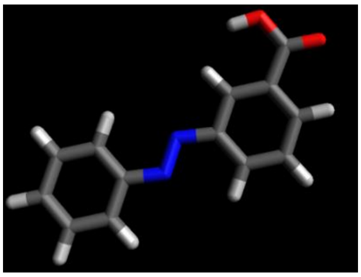

Visualization of calculated geometry of (*E*)-*m*-**3a** :

- The calculated coordinates of (*Z*)-*m*-**3a** (the part of calculated log file) -

Standard orientation:

Center Number	Atomic Number	Atomic Type	Coordinates (Angstroms) X	Y	Z
1	6	0	-2.185016	0.518535	-0.386816
2	7	0	-1.742056	1.610403	-1.204917
3	7	0	-0.593801	2.102951	-1.153261
4	6	0	0.496034	1.520253	-0.418342
5	6	0	1.268480	2.381548	0.373775
6	6	0	2.410981	1.901715	1.009092
7	6	0	2.830594	0.587151	0.801324
8	6	0	2.094196	-0.258862	-0.040664
9	6	0	0.912171	0.203291	-0.631157
10	6	0	-1.976616	0.461503	0.999982
11	6	0	-2.571643	-0.556998	1.741712
12	6	0	-3.354587	-1.527911	1.110849
13	6	0	-3.571155	-1.460673	-0.268205
14	6	0	-3.014292	-0.423034	-1.012257
15	6	0	2.492585	-1.675286	-0.322660
16	8	0	1.716741	-2.515141	-0.727689
17	8	0	3.784913	-2.019860	-0.105171
18	1	0	0.954559	3.414432	0.495785
19	1	0	2.985556	2.556794	1.657113
20	1	0	3.720263	0.231719	1.315540
21	1	0	0.342945	-0.468014	-1.264039
22	1	0	-1.378174	1.218994	1.494670
23	1	0	-2.421888	-0.592441	2.817330

24	1	0	-3.807644	-2.324114	1.694904
25	1	0	-4.191235	-2.204388	-0.760889
26	1	0	-3.201550	-0.333893	-2.078672
27	1	0	4.344550	-1.239953	0.058582

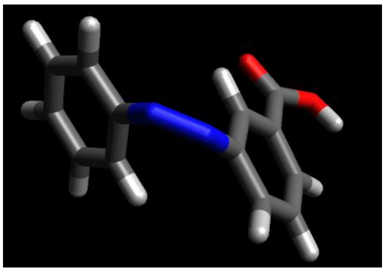

Visualization of calculated geometry of (*Z*)-*m*-**3a**.

- The calculated coordinates of (*E*)-*o*-**3d** (the part of calculated log file) -

Standard orientation:

Center Number	Atomic Number	Atomic Type	Coordinates (Angstroms) X	Y	Z
1	6	0	1.967687	0.234929	0.000029
2	7	0	0.682487	0.778086	0.000065
3	7	0	-0.299623	-0.015872	-0.000201
4	6	0	-1.577289	0.597551	-0.000306
5	6	0	-2.705430	-0.260316	0.000245
6	6	0	-3.981610	0.317388	0.000062
7	6	0	-4.148285	1.698691	-0.000741
8	6	0	-3.027179	2.537635	-0.001319
9	6	0	-1.750694	1.992077	-0.001083
10	6	0	3.019760	1.180780	0.000937
11	6	0	4.358850	0.824458	0.000908
12	6	0	4.688666	-0.530993	-0.000134
13	6	0	3.692967	-1.511490	-0.001098
14	6	0	2.363960	-1.123436	-0.000975
15	9	0	2.699663	2.480846	0.001904
16	9	0	1.429594	-2.089450	-0.001943
17	6	0	-2.663386	-1.773347	0.001031
18	8	0	-3.689192	-2.431499	0.002007
19	8	0	-1.472922	-2.378758	0.000535
20	1	0	-4.837453	-0.348148	0.000546
21	1	0	-5.148029	2.123466	-0.000934
22	1	0	-3.152558	3.616863	-0.001982
23	1	0	-0.869252	2.622780	-0.001531
24	1	0	5.116066	1.600912	0.001698
25	1	0	5.732353	-0.829815	-0.000195
26	1	0	3.931681	-2.569825	-0.001957
27	1	0	-0.747349	-1.695794	-0.000016

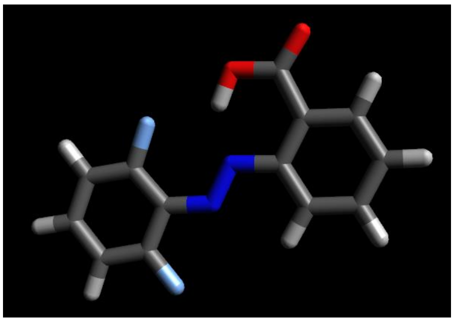

Visualization of calculated geometry of (*E*)-*o*-**3d**

• The calculated coordinates of (*Z*)-*o*-**3d** (the part of calculated log file) -

Standard orientation:

Center Number	Atomic Number	Atomic Type	X	Y	Z
1	6	0	-1.706787	-0.557049	-0.053240
2	7	0	-0.766926	-1.476983	-0.600335
3	7	0	0.449829	-1.218549	-0.751983
4	6	0	1.058798	0.065068	-0.637667
5	6	0	0.471263	1.217652	-1.179519
6	6	0	1.162453	2.425443	-1.161743
7	6	0	2.436557	2.497055	-0.592279
8	6	0	3.030845	1.347864	-0.076429
9	6	0	2.374651	0.113237	-0.119183
10	6	0	-2.994657	-0.526583	-0.612869
11	6	0	-4.037428	0.192880	-0.051179
12	6	0	-3.812601	0.871134	1.149045
13	6	0	-2.562246	0.831938	1.769768
14	6	0	-1.536581	0.121903	1.165454
15	9	0	-3.191207	-1.196136	-1.761006
16	9	0	-0.341613	0.041807	1.779496
17	6	0	3.142772	-1.075892	0.407405
18	8	0	4.170047	-0.938148	1.044288
19	8	0	2.682650	-2.301789	0.120981
20	1	0	-0.508054	1.160131	-1.641019
21	1	0	0.705401	3.309703	-1.596472
22	1	0	2.970799	3.442149	-0.566091
23	1	0	4.024455	1.379471	0.356759
24	1	0	-5.002307	0.206681	-0.546713
25	1	0	-4.621048	1.431295	1.608097
26	1	0	-2.373754	1.333051	2.713397
27	1	0	1.835760	-2.233196	-0.395671

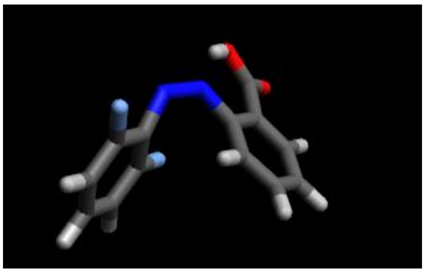

Visualization of calculated geometry of (*Z*)-*o*-**3d**.

• The calculated coordinates of (*E*)-*o*-**3c** (the part of calculated log file) -

Standard orientation:

Center Number	Atomic Number	Atomic Type	X	Y	Z
1	6	0	-1.690258	-0.267964	-0.049518
2	7	0	-0.395147	-0.799547	-0.029601
3	7	0	0.549178	0.037370	-0.034935
4	6	0	1.859993	-0.501075	-0.066024
5	6	0	2.942596	0.410058	0.025539
6	6	0	4.247081	-0.098175	-0.015186
7	6	0	4.486352	-1.462925	-0.142511
8	6	0	3.410782	-2.354372	-0.234071
9	6	0	2.107166	-1.877740	-0.198272
10	6	0	-2.737662	-1.179626	0.190806

11	6	0	-4.070350	-0.795050	0.189668
12	6	0	-4.352316	0.539330	-0.076982
13	6	0	-3.356668	1.483165	-0.340467
14	6	0	-2.033335	1.072126	-0.324687
15	9	0	-5.634579	0.932135	-0.090755
16	9	0	-2.443436	-2.459898	0.446115
17	6	0	2.821573	1.910953	0.170402
18	8	0	3.805742	2.626289	0.217322
19	8	0	1.594281	2.439666	0.251678
20	1	0	5.066757	0.607962	0.055826
21	1	0	5.506970	-1.833390	-0.172651
22	1	0	3.592105	-3.420605	-0.337169
23	1	0	1.260678	-2.550481	-0.273310
24	1	0	-4.858758	-1.512230	0.388110
25	1	0	-3.632727	2.508593	-0.561930
26	1	0	-1.246822	1.782225	-0.552751
27	1	0	0.925796	1.703163	0.183197

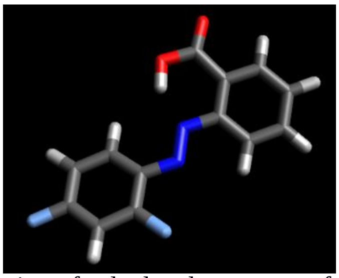

Visualization of calculated geometry of (*E*)-*o*-**3c**

- The calculated coordinates of (*Z*)-*o*-**3c** (the part of calculated log file) -

Standard orientation:

Center Number	Atomic Number	Atomic Type	Coordinates (Angstroms) X	Y	Z
1	6	0	-1.487096	-0.902868	-0.492399
2	7	0	-0.373817	-1.648708	-0.975532
3	7	0	0.811593	-1.239421	-0.959007
4	6	0	1.245647	0.088292	-0.672531
5	6	0	0.585956	1.213556	-1.188811
6	6	0	1.124652	2.483116	-1.002060
7	6	0	2.315101	2.643431	-0.287760
8	6	0	2.984077	1.525021	0.202801
9	6	0	2.485743	0.234444	-0.006745
10	6	0	-1.531281	-0.256672	0.754631
11	6	0	-2.692967	0.316834	1.246901
12	6	0	-3.839072	0.237666	0.461982
13	6	0	-3.858371	-0.415570	-0.767966
14	6	0	-2.685595	-1.010541	-1.217854
15	9	0	-4.969421	0.798422	0.922791
16	9	0	-0.424890	-0.219726	1.518663
17	6	0	3.333080	-0.905075	0.507184
18	8	0	4.272629	-0.713192	1.256101
19	8	0	3.048693	-2.143876	0.081686
20	1	0	-0.327880	1.090269	-1.759113
21	1	0	0.613304	3.347332	-1.416314
22	1	0	2.728429	3.635239	-0.130164
23	1	0	3.917894	1.625918	0.744961
24	1	0	-2.703466	0.802361	2.216512
25	1	0	-4.779089	-0.470141	-1.338621
26	1	0	-2.669659	-1.555868	-2.156610
27	1	0	2.255007	-2.119738	-0.518493

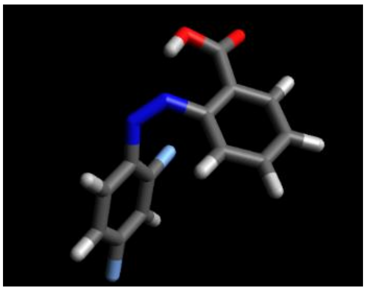

Visualization of calculated geometry of (*Z*)-*o*-**3c**

- The calculated coordinates of (*E*)-*o*-**3b** (the part of calculated log file) -

Standard orientation:

Center Number	Atomic Number	Atomic Type	Coordinates (Angstroms) X	Y	Z
1	6	0	1.839291	-0.485684	0.000312
2	7	0	0.513196	-0.951617	-0.000064
3	7	0	-0.399682	-0.081552	0.000196
4	6	0	-1.731089	-0.574318	0.000256
5	6	0	-2.781845	0.377881	-0.000249
6	6	0	-4.103686	-0.085764	-0.000174
7	6	0	-4.391560	-1.446784	0.000489
8	6	0	-3.347594	-2.379430	0.001040
9	6	0	-2.027906	-1.947455	0.000993
10	6	0	2.812579	-1.500418	-0.002112
11	6	0	4.167268	-1.186815	-0.002312
12	6	0	4.530407	0.155830	0.000306
13	6	0	3.589734	1.188442	0.002770
14	6	0	2.241074	0.865991	0.002848
15	9	0	5.835612	0.476614	-0.000434
16	6	0	-2.609689	1.880352	-0.000769
17	8	0	-3.568747	2.630917	-0.000923
18	8	0	-1.364023	2.369822	-0.001687
19	1	0	-4.897473	0.652762	-0.000579
20	1	0	-5.424568	-1.782416	0.000483
21	1	0	-3.565807	-3.443756	0.001703
22	1	0	-1.206418	-2.654299	0.001445
23	1	0	2.480781	-2.533920	-0.004097
24	1	0	4.933153	-1.955329	-0.004006
25	1	0	3.927205	2.219972	0.004970
26	1	0	1.502863	1.659227	0.005009
27	1	0	-0.724572	1.603527	-0.001463

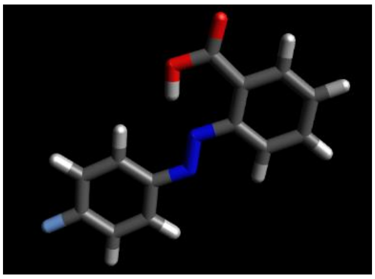

Visualization of calculated geometry of (*E*)-*o*-**3b** :

- The calculated coordinates of (*Z*)-*o*-**3b** (the part of calculated log file) -

Standard orientation:

Center Number	Atomic Number	Atomic Type	Coordinates (Angstroms) X	Y	Z
1	6	0	1.456832	-0.602201	0.855844
2	7	0	0.440692	-1.039750	1.765802

3	7	0	-0.758414	-0.715442	1.641472
4	6	0	-1.216345	0.325517	0.762508
5	6	0	-0.809059	1.643194	1.006996
6	6	0	-1.413583	2.701901	0.331431
7	6	0	-2.433373	2.462327	-0.591741
8	6	0	-2.854019	1.154673	-0.824621
9	6	0	-2.266186	0.077922	-0.146317
10	6	0	2.691856	-0.239321	1.410930
11	6	0	3.752164	0.118463	0.583310
12	6	0	3.572645	0.045785	-0.795121
13	6	0	2.375126	-0.369825	-1.369519
14	6	0	1.305736	-0.689217	-0.536862
15	9	0	4.603740	0.368775	-1.603136
16	6	0	-2.647539	-1.330203	-0.453246
17	8	0	-1.858464	-2.254193	-0.431366
18	8	0	-3.934386	-1.564373	-0.800657
19	1	0	-0.031281	1.831940	1.740515
20	1	0	-1.082838	3.717973	0.527838
21	1	0	-2.895301	3.285314	-1.128221
22	1	0	-3.625037	0.966818	-1.568130
23	1	0	2.804932	-0.223600	2.491007
24	1	0	4.709828	0.429822	0.987968
25	1	0	2.292956	-0.443585	-2.449349
26	1	0	0.373048	-1.051601	-0.955254
27	1	0	-4.498662	-0.791264	-0.614424

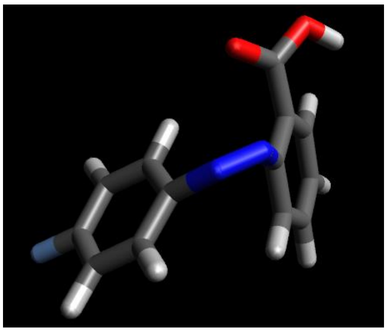

Visualization of calculated geometry of (*Z*)-*o*-**3b**

- The calculated coordinates of (*E*)-*o*-**3a** (the part of calculated log file) -

Standard orientation:

Center Number	Atomic Number	Atomic Type	Coordinates (Angstroms) X	Y	Z
1	6	0	2.243248	-0.414383	0.014171
2	7	0	0.929973	-0.924448	-0.019482
3	7	0	0.000350	-0.074626	0.028491
4	6	0	-1.322112	-0.587498	0.038872
5	6	0	-2.384277	0.348933	-0.027596
6	6	0	-3.699755	-0.131601	-0.006967
7	6	0	-3.968797	-1.494045	0.078702
8	6	0	-2.913008	-2.410747	0.147185
9	6	0	-1.599112	-1.961471	0.129352
10	6	0	3.245188	-1.340710	-0.317804
11	6	0	4.582097	-0.950584	-0.327901
12	6	0	4.925668	0.358810	0.015859
13	6	0	3.929956	1.278778	0.371899
14	6	0	2.592662	0.901941	0.372246
15	6	0	-2.229093	1.850475	-0.126915
16	8	0	-3.197771	2.587616	-0.165914
17	8	0	-0.990345	2.355081	-0.174733
18	1	0	-4.503692	0.593964	-0.059934

19	1	0	-4.997238	-1.843211	0.094785
20	1	0	-3.117681	-3.475401	0.218362
21	1	0	-0.767480	-2.654272	0.187124
22	1	0	2.948986	-2.353502	-0.574072
23	1	0	5.353913	-1.666305	-0.595624
24	1	0	5.968533	0.663720	0.019118
25	1	0	4.203856	2.290817	0.656514
26	1	0	1.824406	1.606484	0.671662
27	1	0	-0.336924	1.602263	-0.122449

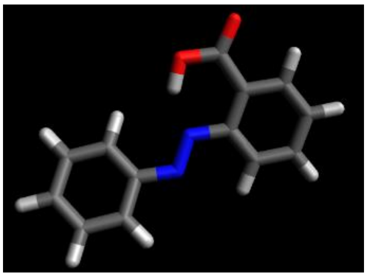

Visualization of calculated geometry of (*E*)-*o*-**3a**

- The calculated coordinates of (*Z*)-*o*-**3a** (the part of calculated log file) -

Standard orientation:

Center Number	Atomic Number	Atomic Type	Coordinates (Angstroms) X	Y	Z
1	6	0	-1.854628	-0.509905	-0.574912
2	7	0	-0.951355	-0.923489	-1.609539
3	7	0	0.260659	-0.625614	-1.602046
4	6	0	0.829576	0.363259	-0.726894
5	6	0	0.416137	1.695779	-0.850433
6	6	0	1.104238	2.708839	-0.185305
7	6	0	2.213524	2.407830	0.607556
8	6	0	2.640240	1.086156	0.717118
9	6	0	1.968736	0.055442	0.044905
10	6	0	-3.119013	-0.057863	-0.974053
11	6	0	-4.066535	0.276987	-0.008967
12	6	0	-3.777090	0.104567	1.347972
13	6	0	-2.533779	-0.400373	1.738047
14	6	0	-1.563938	-0.702963	0.783435
15	6	0	2.369231	-1.370232	0.221984
16	8	0	1.576112	-2.290064	0.245592
17	8	0	3.687499	-1.625720	0.393848
18	1	0	-0.434108	1.932001	-1.482518
19	1	0	0.768550	3.737245	-0.285731
20	1	0	2.741457	3.194725	1.137434
21	1	0	3.485352	0.849541	1.359193
22	1	0	-3.337529	0.041587	-2.033706
23	1	0	-5.037794	0.655180	-0.315881
24	1	0	-4.524845	0.347083	2.098044
25	1	0	-2.317914	-0.562582	2.790685
26	1	0	-0.607568	-1.126324	1.071886
27	1	0	4.230202	-0.843600	0.183461

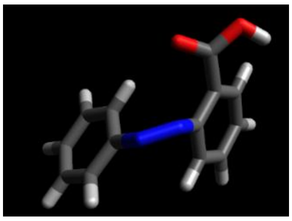

Visualization of calculated geometry of (*Z*)-*o*-**3a**

2.2.2. Molecular Orbitals-

<u>Para</u>

1) Molecular orbitals for the (*E*)-*p*-**3d** model system, HOMO on the left, LUMO on the right.

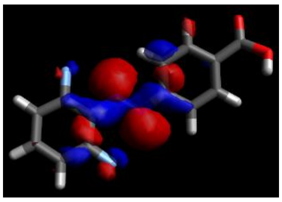

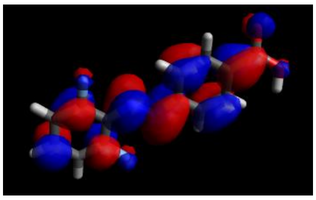

HOMO (-6.474 eV) LUMO (-2.801 eV)

2) Molecular orbitals for the (*Z*)-*p*-**3d** model system**,** HOMO on the left, LUMO on the right.

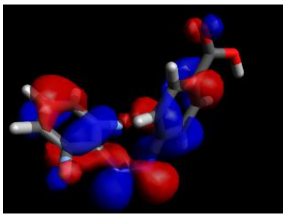

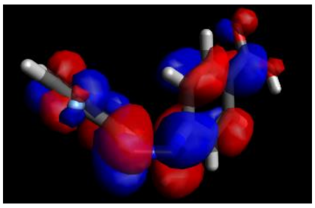

HOMO (- 6.416 eV) LUMO (-2.639 eV)

3) Molecular orbitals for the (*E*)-*p*-**3c** model system**,** HOMO on the left, LUMO on the right.

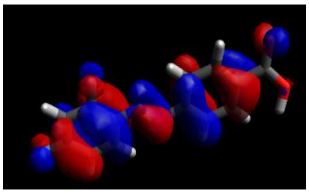

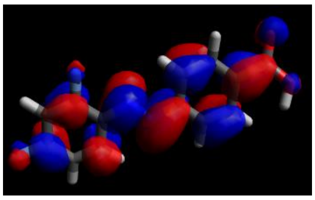

HOMO (-6.540 eV) LUMO (-2.801 eV)

4) Molecular orbitals for the (*Z*)-*p*-**3c** model system**,** HOMO on the left, LUMO on the right.

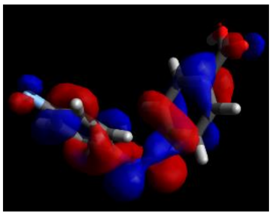

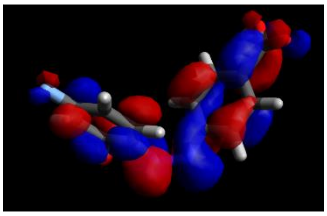

HOMO (-6.283 eV) LUMO (- 2.593 eV)

5) Molecular orbitals for the (*E*)-*p*-**3b** model system**,** HOMO on the left, LUMO on the right.

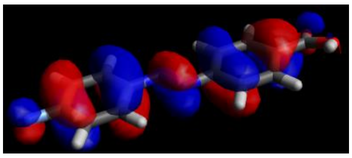

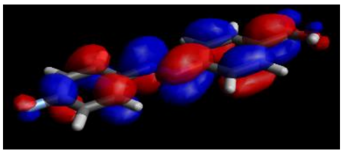

HOMO (-6.472 eV) LUMO (- 2.716 eV)

6) Molecular orbitals for the (*Z*)-*p*-**3b** model system, HOMO on the left, LUMO on the right.

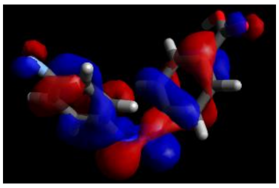

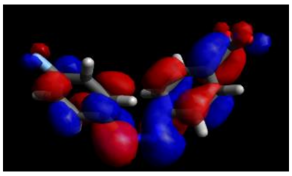

HOMO (-6.178 eV) LUMO (- 2.480 eV)

7) Molecular orbitals for the (*E*)-*p*-**3a** model system**,** HOMO on the left, LUMO on the right.

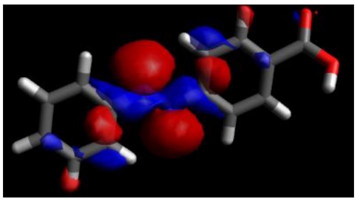

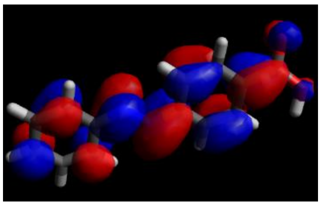

HOMO (- 6.487 eV) LUMO (- 2.705 eV)

8) Molecular orbitals for the (*Z*)-*p*-**3a** model system**,** HOMO on the left, LUMO on the right.

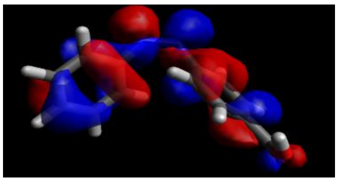

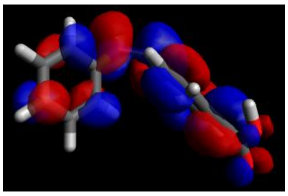

HOMO (-6. 170 eV) LUMO (-2.458 eV)

<u>Meta</u>

1) Molecular orbitals for the (*E*)-*m*-**3d** model system**,** HOMO on the left, LUMO on the right.

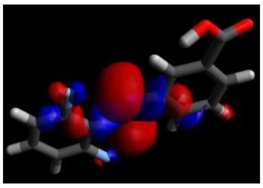

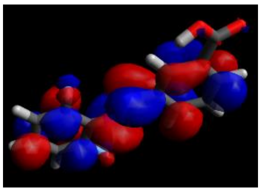

HOMO (-6.473 eV) LUMO (-2.661 eV)

2) Molecular orbitals for the (*Z*)-*m*-**3d** model system**,** HOMO on the left, LUMO on the right.

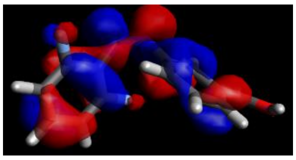

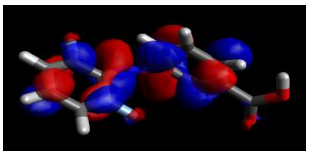

HOMO (- 6.369 eV) LUMO (-2.523 eV)

3) Molecular orbitals for the (*E*)-*m*-**3c** model system**,** HOMO on the left, LUMO on the right.

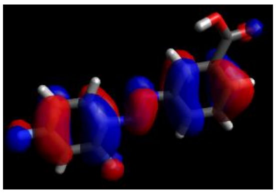

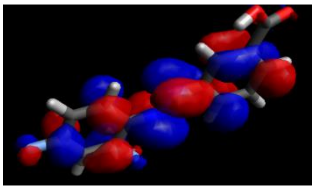

HOMO (- 6.512 eV) LUMO (-2.659 eV)

4) Molecular orbitals for the (*Z*)-*m*-**3c** model system**,** HOMO on the left, LUMO on the right.

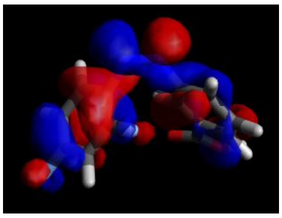

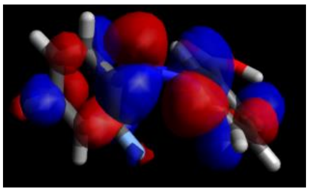

HOMO (-6.262 eV) LUMO (-2.431 eV)

5) Molecular orbitals for the (*E*)-*m*-**3b** model system**,** HOMO on the left, LUMO on the right.

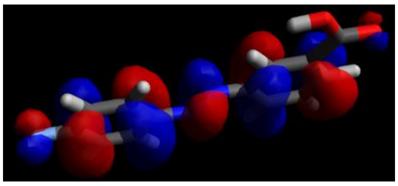

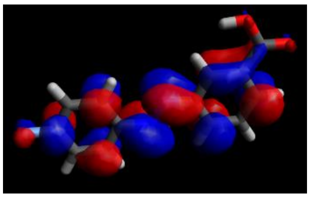

HOMO (-6.447 eV) LUMO (-2.566 eV)

6) Molecular orbitals for the (*Z*)-*m*-**3b** model system**,** HOMO on the left, LUMO on the right.

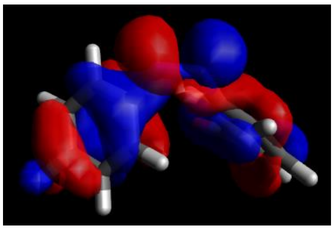

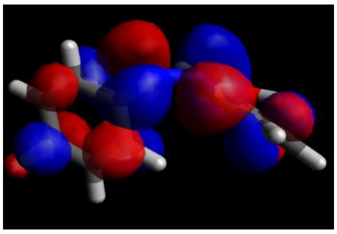

HOMO (- 6.140 eV) LUMO (-2.352 eV)

7) Molecular orbitals for the (*E*)-*m*-**3a** model system**,** HOMO on the left, LUMO on the right.

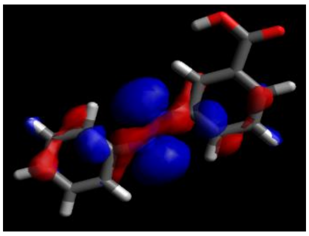

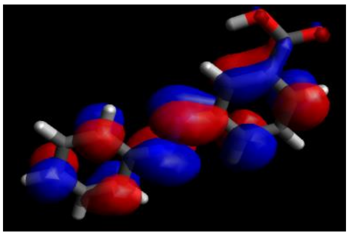

HOMO (-6.490 eV) LUMO (-2.555 eV)

8) Molecular orbitals for the (*Z*)-*m*-**3a** model system**,** HOMO on the left, LUMO on the right.

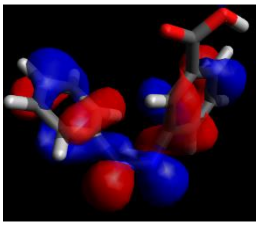

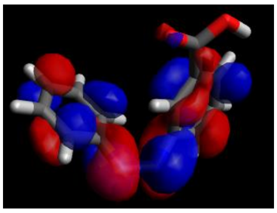

HOMO (-6.129 eV) LUMO (-2.313 eV)

Ortho

1) Molecular orbitals for the (*E*)-*o*-**3d** model system**,** HOMO on the left, LUMO on the right.

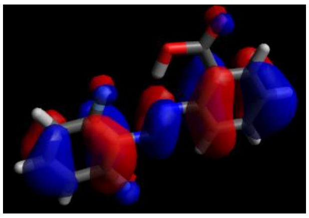

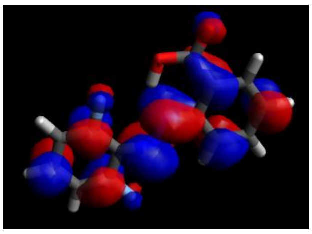

HOMO (-6.777 eV) LUMO (- 3.054 eV)

2) Molecular orbitals for the (*Z*)-*o*-**3d** model system**,** HOMO on the left, LUMO on the right.

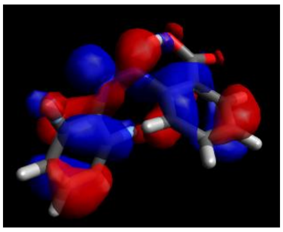

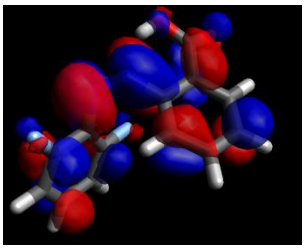

HOMO (-6.592 eV) LUMO (-2.995 eV)

3) Molecular orbitals for the (*E*)-*o*-**3c** model system**,** HOMO on the left, LUMO on the right.

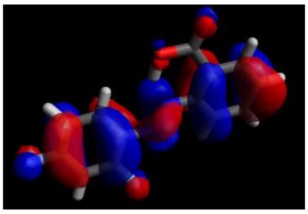

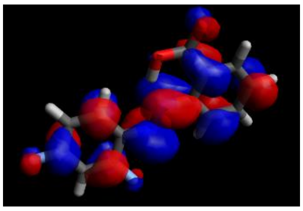

HOMO (-6.659 eV) LUMO (-3.060 eV)

4) Molecular orbitals for the (*Z*)-*o*-**3c** model system**,** HOMO on the left, LUMO on the right.

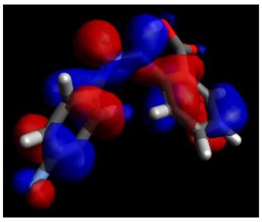

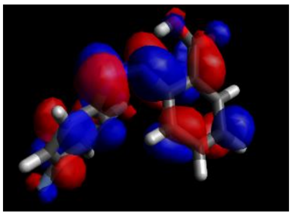

HOMO (-6.478 eV) LUMO (-2.933 eV)

5) Molecular orbitals for the (*E*)-*o*-**3b** model system**,** HOMO on the left, LUMO on the right.

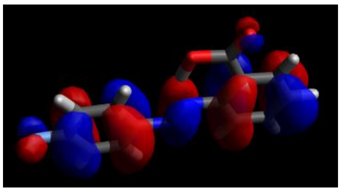

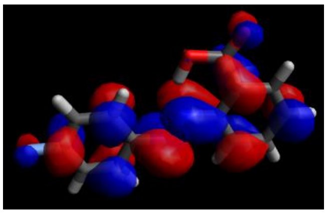

HOMO (-6.625 eV) LUMO (-2.972 eV)

6) Molecular orbitals for the (*Z*)-*o*-**3b** model system**,** HOMO on the left, LUMO on the right.

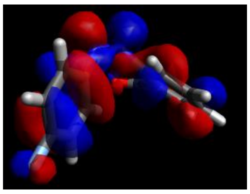

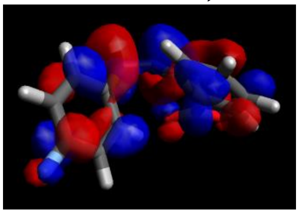

HOMO (-6.121 eV) LUMO (-2.251 eV)

7) Molecular orbitals for (*E*)-*o*-**3a** model system**,** HOMO on the left, LUMO on the right.

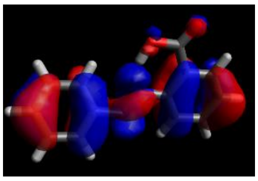

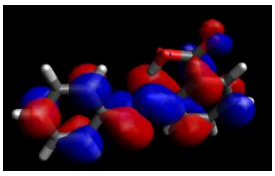

HOMO (-6.650 eV) LUMO (-2.955 eV)

8) Molecular orbitals for the (*Z*)-*o*-**3a** model system**,** HOMO on the left, LUMO on the right.

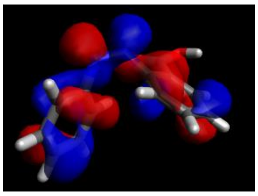

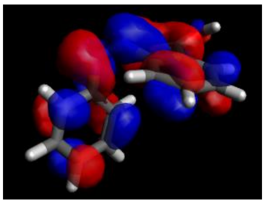

HOMO (-6.114 eV) LUMO (-2.220 eV)

[1] Frisch, M.J.; Trucks, G.W.; Schlegel, H.B.; Scuseria, G.E.; Robb, M.A.; Cheeseman, J.R.; Montgomery, J.A.; Vreven, T.; Kudin, K.N.; Burant, J.C.; Millam, J.M.; Iyengar, S.S.; Tomasi, J.; Barone, V.; Mennucci, B.; Cossi, M.; Scalmani, G.; Rega, N.; Petersson, G.A.; Nakatsuji, H.; Hada, M.; Ehara, M.; Toyota, K.; Fukuda, R.; Hasegawa, J.; Ishida, M.; Nakajima, T.; Honda, Y.; Kitao, O.; Nakai, H.; Klene, M.; Li, X.; Knox, J.E.; Hratchian, H.P.; Cross, J.B.; Bakken, V.; Adamo, C.; Jaramillo, J.; Gomperts, R.; Stratmann, R.E.; Yazyev, O.; Austin, A.J.; Cammi, R.; Pomelli, C.; Ochterski, J.W.; Ayala, P.Y.; Morokuma, K.; Voth, G.A.; Salvador, P.; Dannenberg, J.J.; Zakrzewski, V.G.; Dapprich, S.; Daniels, A.D.; Strain, M.C.; Farkas, O.; Malick, D.K.; Rabuck, A.D.; Raghavachari, K.; Foresman, J.B.; Ortiz, J.V.; Cui, Q.; Baboul, A.G.; Clifford, S.; Cioslowski, J.; Stefanov, B.B.; Liu, G.; Liashenko, A.; Piskorz, P.; Komaromi, I.; Martin, R.L.; Fox, D.J.; Keith, T.; Al-Laham, M.A.; Peng, C.Y.; Nanayakkara, A.; Challacombe, M.; Gill, P.M.W.; Johnson, B.; Chen, W.; Wong, M.W.; Gonzalez, C.; Pople, J.A.; Gaussian Inc., Wallingford, CT, 2004 (Gaussian 03, Revision E.01).

[2] Tomasi, J.; Mennucci, B.; Cammi, R. Quantum Mechanical Continuum Solvation Models. *Chem. Rev.* **2005**, *105*, 2999–3094; doi:10.1021/CR9904009.

[3] Bagnato, J.D.; Eilers, A.L.; Horton, R.A.; Grissom, C.B. Synthesis and Characterization of a Cobalamin-colchicine Conjugate as a Novel Tumor-targeted Cytotoxin. *J. Org. Chem.* **2004**, *69*, 8987–8996; doi:10.1021/JO049953W.

[4] Borowiak, M.; Borowiak, M.; Nahaboo, W.; Reynders, M.; Nekolla, K.; Jalinot, P.; Hasserodt, J.; Rehberg, M.; Delattre, M.; Zahler, S. Photoswitchable Inhibitors of Microtubule Dynamics Optically Control Mitosis and Cell Death. *Cell* **2015**, *162*, 403-411; doi:10.1016/J.CELL.2015.06.049.

[5] Sailer, A.; Ermer, F.; Kraus, Y.; Lutter, F.H.; Donau, C.A.; Bremerich, M.; Ahlfeld, J.; Thorn-Seshold, O. Hemithioindigos for Cellular Photopharmacology: Desymmetrised Molecular Switch Scaffolds Enabling Design Control over the Isomer-dependency of Potent Antimitotic Bioactivity. *ChemBioChem* **2019**, *20*, 1305-1314; doi:10.1002/CBIC.201800752.

[6] Sailer, A.; Meiring, J.C.M.; Heise, C.; Pettersson, L.N.; Akhmanova, A.; Thorn-Seshold, J.; Thorn-Seshold, O. Pyrrole Hemithioindigo Antimitotics with Near-quantitative Bidirectional Photoswitching Photocontrol Cellular Microtubule Dynamics with Single-cell Precision. *Angew. Chem. Int. Ed.* **2021**, *60*, 23695-23704; doi:10.1002/ANIE.202104794.

[7] Gao, L.; Meiring, J.C.M.; Kraus, Y.; Wranik, M.; Weinert, T.; Pritzl, S.D.; Bingham, R.; Ntouliou, E.; Jansen, K.I.; Olieric, N. A Robust, Gfp-orthogonal Photoswitchable Inhibitor Scaffold Extends Optical Control over the Microtubule Cytoskeleton. *Cell Chem. Biol.* **2021**, *28*, 228-241; doi:10.1016/J.CHEMBIOL.2020.11.007.

[8] Gao, L.; Meiring, J.C.; Varady, A.; Ruider, I.E.; Heise, C.; Wranik, M.; Velasco, C.D.; Taylor, J.A.; Terni, B.; Weinert, T.; Standfuss, J.; Cabernard, C.C.; Llobet, A.; Steinmetz, M.O.; Bausch, A.R.; Distel, M.; Thorn-Seshold, J.; Akhmanova, A. Thorn-Seshold, O. In vivo photocontrol of microtubule dynamics and integrity, migration and mitosis, by the potent GFP-imaging-compatible photoswitchable reagents SBTubA4P and SBTub2M. *J. Am. Chem. Soc.* **2022**, *144*, 5614-5628; doi: 10.1021/jacs.2c01020.
